# Structure based discovery of antipsychotic-like TAAR1 agonists

**DOI:** 10.1038/s41467-026-77484-2

**Published:** 2026-09-17

**Authors:** Sijie Huang, Heng Liu, Yujin Wu, Joao M. Braz, Divya Kranthi, Brendan W. Hall, Xinyue Zhang, Dmytro S. Radchenko, Yurii S. Moroz, John J. Irwin, Allan I. Basbaum, H. Eric Xu, William C. Wetsel, Brian K. Shoichet

**Affiliations:** 1 Department of Pharmaceutical Chemistry, University of California, San Francisco, San Francisco, CA USA; 2 State Key Laboratory of Drug Research, Shanghai Institute of Materia Medica, Chinese Academy of Sciences, Shanghai, China; 3 Department of Anatomy, University of California, San Francisco, San Francisco, CA USA; 4 Enamine Ltd., Kyiv, Ukraine; 5 National Taras Shevchenko University of Kyiv, Kyiv, Ukraine; 6 Chemspace LLC, Kyiv, Ukraine; 7 University of Chinese Academy of Sciences, Beijing, China; 8 Departments of Psychiatry and Behavioral Sciences, Cell Biology, and Neurobiology, Mouse Behavioral and Neuroendocrine Analysis Core Facility, Duke University Medical Center, Durham, NC USA

**Keywords:** Drug discovery, Virtual drug screening, Cryoelectron microscopy, Single-molecule biophysics

## Abstract

Schizophrenia is a severe mental illness whose current treatments primarily target dopamine and serotonin receptors. These drugs often cause side effects and vary in effectiveness across patients. The trace amine-associated receptor 1 (TAAR1), which modulates monoamine signaling, has emerged as a promising alternative target. To discover previously unexplored TAAR1 ligands, we computationally dock 65 million molecules against the active state of TAAR1 and experimentally test 55 of those highly ranked. Fourteen TAAR1 agonists emerge with potencies ranging from mid-nanomolar to micromolar. This high functional selectivity may reflect the compact conformation adopted by the activated TAAR1 orthosteric site. While this is favorable for agonist prioritization, simulations suggest that it can be over-optimized for initial hit rates at the expense of subsequent affinity maturation. Hit optimization yields nanomolar agonists whose docking-predicted poses are confirmed by cryo-EM. Three agonists have high brain exposure and potencies, with one compound showing potency comparable to the investigational drug ulotaront and two compounds exhibiting even greater potency, at levels sufficient for behavioral studies. All three potently normalize amphetamine-induced pre-pulse inhibition in mice, a model for schizophrenia, without catalepsy, a common side effect of traditional antipsychotics.

## Abstract (web summary)

In this work, large library docking against the trace amine receptor reveals agonists that are optimized to nanomolar potency. In a mouse model, the molecules reversed schizophrenia-like behavior. Experimental structures of the reported agonists support the docking predictions and template their further development.

## Introduction

The trace amine-associated receptor 1 (TAAR1) is a G protein-coupled receptor (GPCR) that has received growing attention in recent years for its role in modulating monoaminergic neurotransmission^1^. TAAR1 is activated by trace amines such as β-phenylethylamine (β-PEA), p-tyramine, tryptamine, and several synthetic psychostimulants, including amphetamine^2^ and methamphetamine (METH)^3^; its native neurotransmitter remains uncertain. Unlike other members of the TAAR family, which are predominantly expressed in olfactory regions^4^, TAAR1 is widely expressed in the brain, particularly in monoaminergic nuclei such as the ventral tegmental area (VTA) and dorsal raphe nucleus (DRN)^5,6^. This distribution is consistent with its regulation of the dopaminergic^5^ and serotonergic^7^ systems, including reward circuits and mood^8^. These properties make TAAR1 a potential target for psychiatric disorders like schizophrenia, anxiety, depression, and psychostimulant addiction^6,9–11^. Indeed, the drug SEP-363856 (ulotaront), a potent TAAR1 agonist with joint 5-HT_1A_ activity, was developed as a new therapeutic to treat schizophrenia^11,12^. Ulotaront was designed to address both the positive and negative symptoms of the disorder while minimizing the side effects often associated with conventional antipsychotics^13,14^. Despite promising results in early-phase studies, the drug failed to meet the primary endpoints in Phase 3 trials and was not further advanced.

Here we leverage an experimental structure of TAAR1 in its active form to find agonists unrelated to those in the literature using large library docking, which has recently shown promise against multiple targets^9,15–30^. Despite ulotaront’s limitations, TAAR1 remains an interesting target to treat schizophrenia^31–35^, with new chemotypes being explored including from a successful library docking campaign that targeted a homology model of the receptor^33,36^. Docking a library of 65 million fragment-like molecules against the active state of TAAR1 reveals agonists with potencies ranging from 22 nM to 18 µM, with an overall hit-rate (number active/number tested) of 25% (14/55). Three of these are optimized for activity and for pharmacokinetics (PK), reaching TAAR1 potencies from 1.5 to 72 nM. Encouragingly, these molecules are also active at the 5-HT_1A_ receptor, a co-target with TAAR1 for antipsychotic efficacy^37,38^. While potency is lower against 5-HT_1A_, ranging from 377 nM to 3.2 µM, these activities are higher than that of ulotaront and likely relevant given the micromolar brain-free fractions reached by these molecules. With their high potency and favorable PK properties, three compounds are further evaluated for antipsychotic-like efficacy in mouse models.

## Results

### Large library docking identifies TAAR1 agonists

**Fig. 1:**
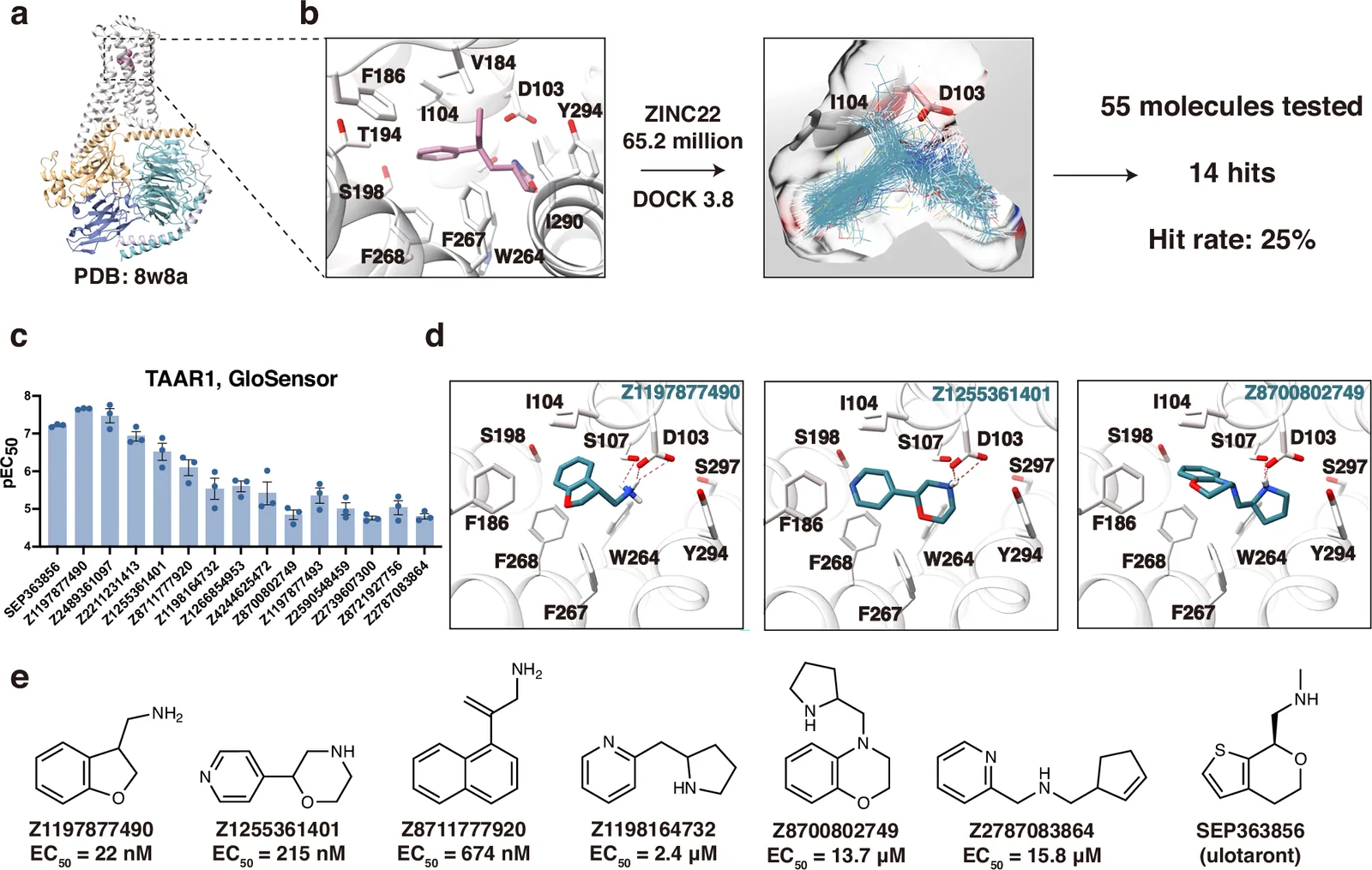
Docking for and in vitro testing of TAAR1 agonists. **a** The cryo-EM structure of the human TAAR1 complex (PDB 8w8a). **b** A library of 65 million compounds was docked to the TAAR1 complex that included G protein. Binding modes of top-ranked compounds from the virtual screening and docked compounds are shown as lines. Hit rates were defined by more than a 30% GloSensor response compared with SEP-363856 at 10 μM. **c** The GloSensor response of the initial hits at 10 μM. Data are mean ± SEM from three independent experiments (*n* = 3). **d** Docked poses of the hits at the orthosteric site are shown. **e** Chemical structures of the exemplar docking hits, with the TAAR1 investigational drug ulotaront (far right) shown for comparison. Source data are provided as a Source data file.

The ligands were docked against the human TAAR1 orthosteric pocket largely as defined by the configuration of the agonist RO5256390^39^ in that site (PDB: 8W8A) (Fig. 1a, b). Using DOCK3.7^15,40^, molecule orientations were sampled using pre-defined hot spots (“matching spheres”) as guides. For each orientation, up to 200 ligand conformations were sampled from precomputed low-energy conformational ensembles^40,41^. Each ligand configuration was scored electrostatic complementarity using a probe-charge model in pre-calculated electrostatic potential grids calculated by the Poisson-Boltzmann method QNIFFT^42^, and for non-polar complementarity using pre-calculated van der Waals terms from the AMBER potential^43^. Context-dependent ligand desolvation was calculated using SOLVMAP^44^.

As in purely experimental studies, control calculations are important sanity checks before launching prospective docking campaigns^45^. We tested our sampling and the energy potential maps used for scoring by asking whether we could highly-rank known TAAR1 agonists against larger sets of property-matched^40^ and extrema decoys^45–47^. Electrostatic and desolvation energies may be adjusted by changing the boundaries between low- and high-dielectric in these calculations; we varied these to reach log-weighted AUC values of 31.7 (Supplementary Fig. 1). This relatively favorable value suggested we could well-distinguish true TAAR1 ligands from decoy molecules.

With these controls in hand, we screened approximately 65 million fragment-like cations (between 11 and 19 non-hydrogen atoms) from the ZINC22 compound library^48^. This calculation took 39,993 core hours, or less than two days on 1000 cores. Seeking novelty, from the top 382,767 scored molecules we excluded compounds that topologically resembled known TAAR1 agonists; any docked molecule with an ECFP4-based Tanimoto coefficient > 0.35 was removed. To ensure direct interaction between ligand cations and the recognition sites D103^3.32^, Y294^7.43^, F267^6.51^, F268^6.52^, I104^3.33^, and F186^ECL2^ within the orthosteric binding pocket of the receptor, interaction fingerprint-based filters were implemented using LUNA. This approach ensured that the cation was not only well-complemented by the electrostatic potential of the receptor in the area of D103^3.32^—which is what was scored in DOCK3.8—but also that a recognizable ion-pair was made. The 95,126 compounds that remained were clustered by topology (see Methods) and the best scoring molecules from the top clusters were visually evaluated using Chimera^49^. From these, 2462 diverse molecules, none more similar to one another than an ECFP4 Tc of 0.25, were inspected for favorable geometry and interactions. Sixty-six were prioritized of which 55 were successfully synthesized by Enamine, an 83% fulfillment rate (Fig. 1b).

To evaluate the ability to activate TAAR1, the 55 docking hits were first screened at 10 μM using functional live-cell GloSensor assays^50^ to evaluate their ability to activate TAAR1 by recruiting Gs and inducing cAMP production (Fig. 1b, c). Fourteen of the 55 compounds had agonist activity with efficacies (Emax) exceeding 30% of ulotaront’s (SEP363856) (Fig. 1b, c, Supplementary Fig. 1a, and Supplementary Table 1). Concentration-response experiments confirmed that these compounds acted as TAAR1 agonists, with the negative logarithm of the half-maximal effective concentration (pEC_50_) values ranging from 4.76 to 7.65 and Emax values ranging between 34% and 108% of the ulotaront E_max_ (Fig. 1c and Supplementary Fig. 1c). In empty-vector (no transfected receptor) controls, no activity was observed for the initial active compounds nor for the optimized leads, below, in both single-point and concentration-response assays, consistent with an on-target effect of the discovered molecules in the cellular assays. Based on their potency and dissimilarity to TAAR1 compounds annotated in ChEMBL (i.e., ECFP4-based Tc of 0.32, 0.18, and 0.26, respectively) (Supplementary Table 2), we prioritized compounds Z1197877490, Z1255361401, and Z8700802749 for structure-based optimization (Fig. 1c–e, Supplementary Figs. 2 and 3, and Supplementary Tables 1 and 3).

### Only agonists found

In principle, docking against an activated or inactivated state of a receptor should bias toward the discovery of agonists or antagonists, respectively. Whereas we have occasionally observed this to be true^19,51–53^, more often we have found a mix of agonists and antagonists^15,22,54–56^. This result is consistent with the idea that docking primarily selects for binding and struggles to distinguish between activating and inactivating ligand interactions. However, in docking against the TAAR1 active state, all 14 actives were agonists and none of the 55 tested were active in antagonist assays (Supplementary Fig. 4). To better understand this result, we modeled an inactive structure using Boltz-2^57^, which is among the fastest of the co-folding methods and has been optimized for interaction energy prediction. We asked whether the 28 agonists (the 14 discovered agonists and 14 known ones) had better fits and ranks when docked to the experimental active-state structure versus the modeled inactive state structure, comparing also the behavior of seven known antagonists^7,58–61^ (Supplementary Table 4). Against the active-state structure of TAAR1, 23 of the 28 agonists ranked among the among the top ~0.5% of the docked library, with DOCK scores ranging from −54 kcal/mol to −33 kcal/mol (382,767 in total). Only one of the seven antagonists fell in this range, with a score of −45 kcal/mol. Docked against the inactive state model, the agonist scores shifted to worse (less negative) values, ranging from −44 kcal/mol to −26 kcal/mol, worsening by an average 8.5 kcal/mol, and their relative ranks suffered even further. Meanwhile, the performance of the antagonists improved, with the best now receiving a score of −46 kcal/mol and two others climbing by between −42 and −35 kcal/mol into the top-ranking molecules (Supplementary Fig. 5).

The unusual ability of the TAAR1 active state docking to prioritize agonists, and of the inactive structure to relatively prioritize antagonists, may reflect the size distinction between agonists and antagonists and between active and inactive receptor conformations (Supplementary Fig. 6). TAAR1 agonists are typically smaller than its antagonists, and the TAAR1 orthosteric site compacts on activation (the site contracts even more in a TAAR1 modeled structure used for docking (Supplementary Fig. 6), a point to which we will return). Another receptor where this is true is the α_2A_-adrenergic receptor, where docking has also prioritized exclusively agonists from active-state docking^19^. It may be that docking’s ability to predict ligand function (agonist, antagonist) will be strongest where there are large differences in the physical properties of the active and inactive states—here site volume. Where differences are subtler, docking may struggle to distinguish among ligand functional classes.

### Initial hit optimization

To enhance the potency of the initial docking-active compounds, we employed two strategies. The first involved searching within Enamine REAL Database (e.g., using the SmallWorld program, https://sw.docking.org/) to find similar molecules. In a second strategy, conservative modifications were introduced into to the initial hits wherever synthetically feasible, a strategy that can be remarkably effective^44^. To the extent that design was used in optimization, it was driven by complementarity to the TAAR1 structure; activity for the 5-HT_1A_ was investigated subsequently.

**Fig. 2:**
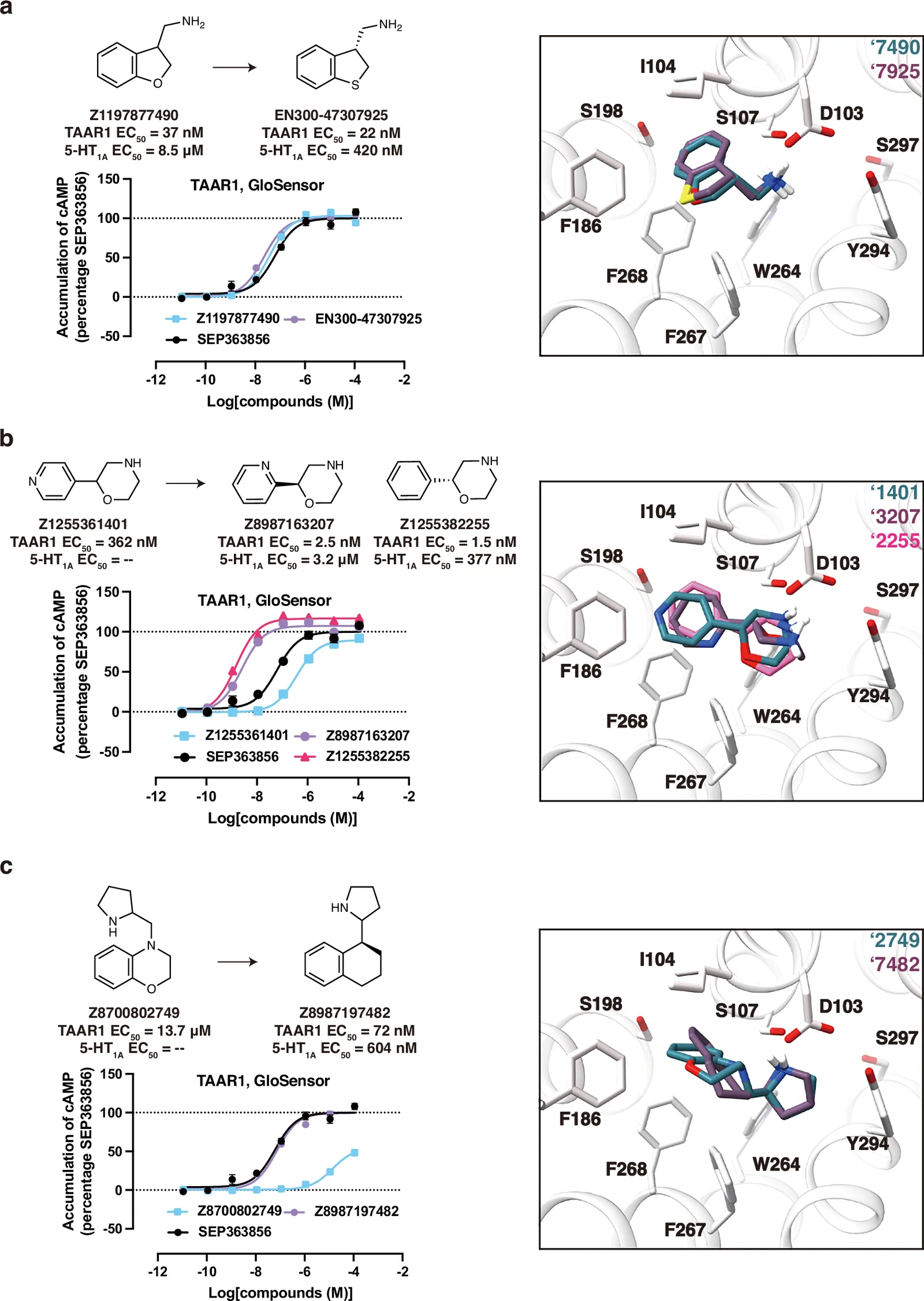
Optimization of ‘7490, ‘1401, and ‘2749. **a** Docking hit ‘7490 (teal) and its optimized analog ‘7925 (rebeccapurple). Superposition of docked poses of ‘7490 and ‘7925 in the TAAR1 orthosteric site. The concentration-response curves shown correspond to TAAR1 activation. Data are mean ± SEM from three independent experiments (*n* = 3). **b** Docking hit ‘1401 (teal) and its optimized analogs ‘3207 (rebeccapurple) and ‘2255 (hotpink). Superposition of docked poses of ‘1401, ‘3207, and ‘2255 in the TAAR1 orthosteric site. The concentration-response curves shown correspond to TAAR1 activation. Data are mean ± SEM from three independent experiments (*n* = 3). **c** Docking hit ‘2749 (teal) and its optimized analog ‘7482 (rebeccapurple). Superposition of docked poses of ‘2749 and ‘7482 in the TAAR1 orthosteric site. The concentration-response curves shown correspond to TAAR1 activation. Data are mean ± SEM from three independent experiments (*n* = 3). “--” indicates that the value could not be determined due to insufficient compound activity. Source data are provided as a Source data file.

We selected 2012 analogs of Z1197877490 using SmallWorld and explored 27 additional small change derivatives. For Z1255361401, we found 1707 analogs and designed 13 small-change derivatives. For Z8700802749, we found 873 analogs from the database. We docked these to TAAR1; five analogs of ‘7490, six of ‘1401, and one of ‘2749 were prioritized for synthesis at Enamine. The parent agonist ‘7490 began with a TAAR1 EC_50_ of 37 nM and further modifications, such as replacing its oxygen with carbon or sulfur atoms only slightly improved potency (Fig. 2a). Stereochemical purification to EN300-47307925 (Fig. 2a and Supplementary Fig. 2a) also had little effect on TAAR1 potency, though it enhanced 5-HT_1A_ EC_50_ by 20-fold to 420 nM, nearly eight-fold higher than ulotaront. Conversely, small modifications to the 362 nM ‘1401 much improved potency (Fig. 2b and Supplementary Fig. 2b). Moving the nitrogen within the pyridine ring or replacing the pyridine with a benzene increased activity by 90- to 164-fold (Fig. 2b and Supplementary Fig. 2b). Enantiomeric resolution led to Z898713207 (EC_50_ 2.5 nM) and Z1255382255 (EC_50_ 1.5 nM), with the latter exhibiting a 5-HT_1A_ EC_50_ of 377 nM (Fig. 2b and Supplementary Fig. 2b). Finally, for the ‘2749 series, moving the cationic nitrogen two carbon atoms away from the benzene ring enhanced activity 39-fold (Fig. 2c and Supplementary Fig. 3a). Enantiomeric separation led to Z8987197482 with a TAAR1 EC_50_ of 72 nM and a 5-HT_1A_ EC_50_ of 604 nM (Fig. 2c and Supplementary Fig. 3a). Docked poses suggested that shortening the linker by one carbon in ‘7482 repositioned the tetralin group closer to the center of the binding pocket, potentially enhancing π-π stacking (Fig. 2c).

To measure selectivity, we tested Z898713207, Z8987197482, and Z1255382255 across related aminergic receptors. All three compounds exhibited high agonist activity at hTAAR1 and mTAAR1, whereas their agonist activities at 5-HT_1A_, DRD2, and 5-HT_2A_ were lower, indicating a clear preference for TAAR1 over other major monoaminergic receptors (Supplementary Fig. 7).

### Structural determination by Cryo-EM

**Fig. 3:**
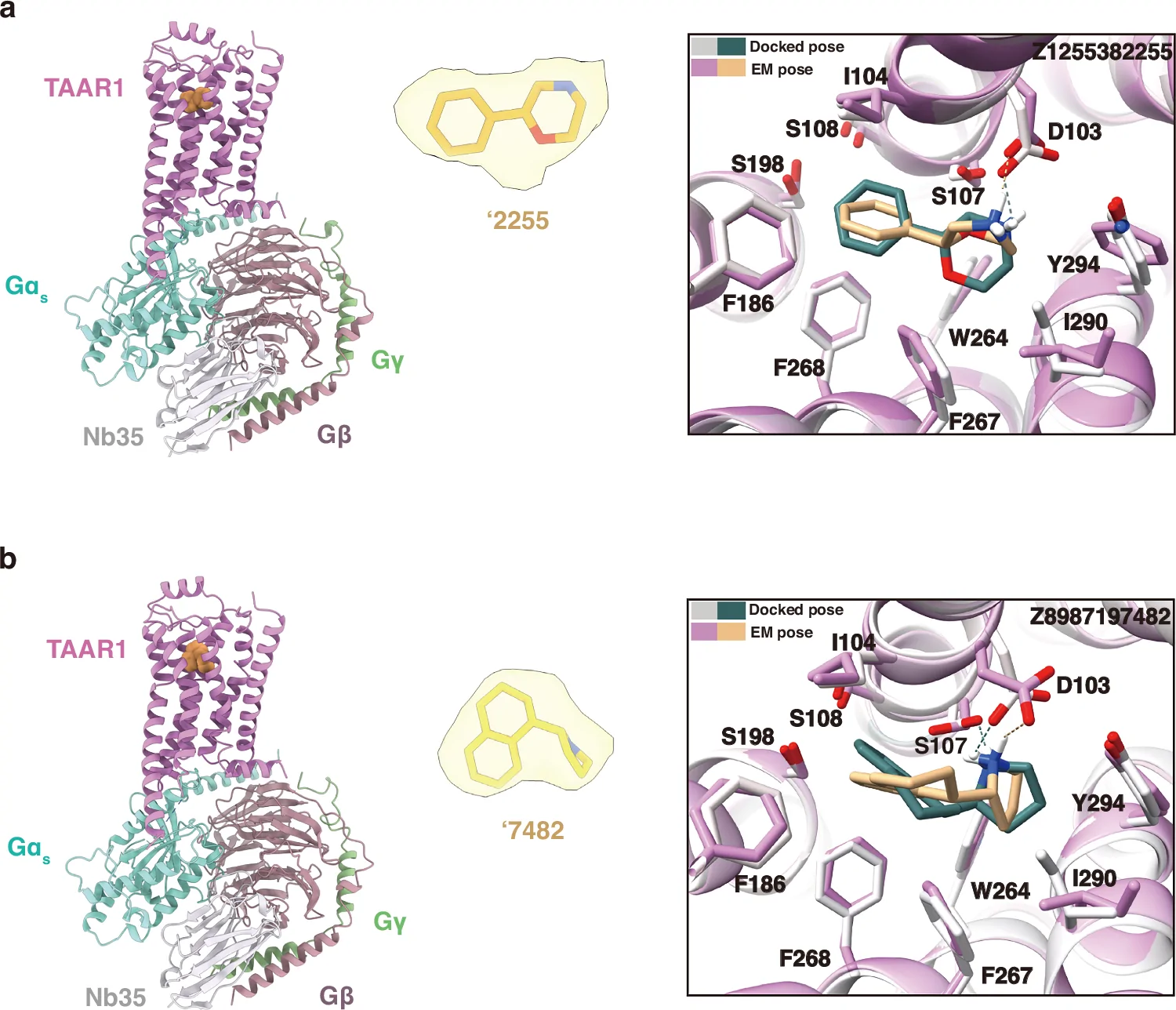
Cryo-EM structures of the ‘2255-TAAR1-Gs and ‘7482-TAAR1-Gs complexes. **a** Cryo-EM model of ‘2255-TAAR1 highlighting the ligand density. Superposition of the docked pose (darkslategrey) and experimentally determined pose (burlywood) of ‘2255 in the orthosteric site. **b** Cryo-EM model of ‘7482-TAAR1 highlighting the ligand density. Superposition of the docked pose (darkslategrey) and experimentally determined pose (burlywood) of ‘7482 in the orthosteric site.

To understand the molecular basis of ligand recognition and provide a template for future optimization, we determined the complex structures of TAAR1 bound to two low-nanomolar agonists: ‘2255 and ‘7482 (Fig. 3, Supplementary Figs. 8 and 9, and Supplementary Table 5). The global nominal resolutions of the corresponding cryo-EM density maps for these complexes were 3.14 Å and 3.12 Å, respectively. The overall structures of the complexes resembled previously reported experimental TAAR1 structures, with root mean square deviation (RMSD) values of 0.492 Å and 0.846 Å, respectively, when compared to the TAAR1 structure 8W8A^39^.

In these two TAAR1 structures, the receptor adopts a canonically activated conformation, with both agonists occupying the orthosteric site and TM6 exhibiting similar degrees of outward displacement. TAAR1 appears to recognize these agonists primarily through two ligand moieties: an aryl group that forms π-π and packing interactions with a sub-pocket formed by F268^6.52^, F186^ECL2^, I104^3.33^, and a cationic amine that ion-pairs with D103^3.32^. These interactions are both predicted by the docking and are captured in the cryo-EM structure. The docked-predicted and cryo-EM observed agonist poses superpose with RMSD values of 2.3 Å and 1.4 Å for ‘2255 and ‘7482, respectively (Fig. 3a, b).

Although the experimental and predicted agonist poses make the same receptor interactions, there were meaningful differences between the structures. In the experimental pose of ‘2255, the ligand undergoes a conformational rotation from its relatively extended and planar docking pose. This reorientation positions the phenyl ring at an inclined angle, facilitating a π-π interaction with F268^6.52^ while at the same time bringing it closer to F186 on ECL2. Similarly, the cationic nitrogen undergoes a shift within the saturated ring system to optimize salt bridge formation with D103^3.32^. As the cationic nitrogen moves closer to D103^3.32^, the residue is slightly displaced outward. Similarly, ‘7482 shifts toward ECL2, bringing its phenyl ring closer to F268^6.52^. Its cationic nitrogen also moves closer to D103^3.32^, resulting in a greater lateral displacement of this residue compared to the ‘2255-bound structure (Fig. 3a, b). More broadly, both the experimental and docking structures of these two compounds help to explain the SAR observed with these two series. For instance, the move from the pyridine of the 2.5 nM ‘3207 to the phenyl of the 1.5 nM ‘2255 is consistent with the placement of the phenyl in the non-polar, “aryl cage” of TAAR1. In this geometry, the pyridine would pay a desolvation cost without compensating polar interactions in the site, while the phenyl would not pay this cost. Similarly, the move from the morpholino moiety of ‘2749 (13.7 µM) to the cyclohexyl moiety of ‘7482 (72 nM), where both are posed in a non-polar sub-pocket, helps to explain the improved potency of the latter (the reduced planarity and better fit of the latter likely also contributes to this >190-fold improvement).

### Comparison to docking against AlphaFold2 models of TAAR1

**Fig. 4:**
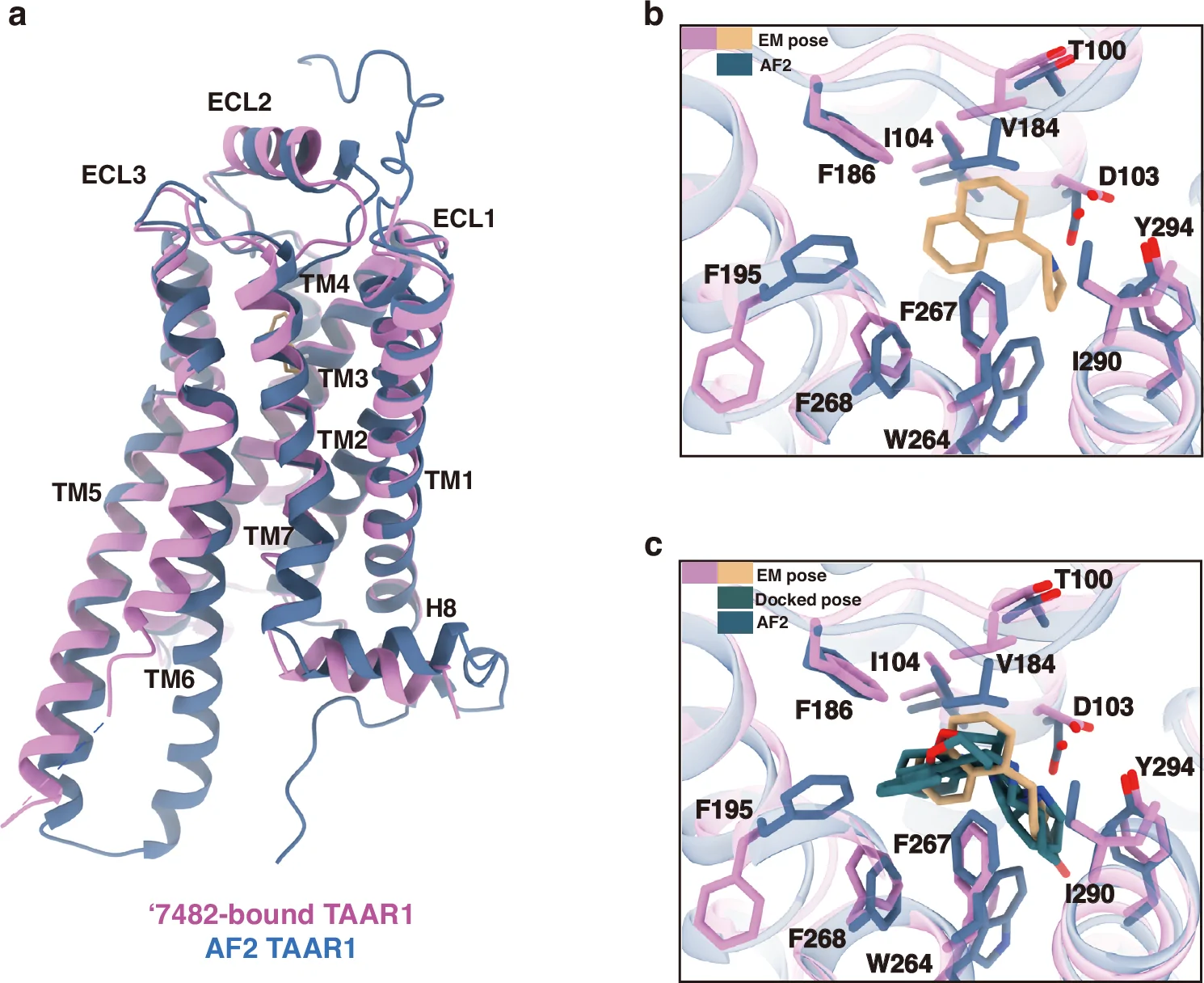
Comparison of the experimental TAAR1 structure and an AlphaFold TAAR1 model^36^, along with the positions of docking-predicted molecules in both structures. **a** Superposition of ‘7482 (burlywood)-bound experimental TAAR1 (violet) and AlphaFold TAAR1 (slategrey) structure. **b** Superposition of the experimentally determined pose of compound ‘7482 (burlywood) with the AlphaFold TAAR1 (slategrey) structure in the orthosteric site. **c** Superposition of the experimental ‘7482-bound TAAR1 orthosteric site with several docking hit poses (teal) and the orthosteric site of the AlphaFold TAAR1 model.

In a fascinating recent study, library docking was used to find agonists targeting an AlphaFold2 (AF2) model of TAAR1^36^. Beginning with 100 different models, the authors selected those that best enriched known TAAR1 ligands versus decoys and used these to template a prospective search of a large library, a strategy they had helped pioneer^62^. On testing of high-ranking molecules, 60% of their predictions were active, with the best of them having an EC_50_ of 35 nM. By hit rate, these results are substantially better than what we found here using essentially the same docking program, while the affinities are similar for the two screens. Despite the fact that the AF2 model was in an inactivate conformation (Fig. 4a and Supplementary Fig. 6), all of their new ligands were functionally agonists. How may these observations be reconciled?

Ordinarily one might expect an experimental structure, especially one that adopts the full activated state, as our cryo-EM structure does, to perform at least as well as an AF2 model, and to be better biased toward agonists. One advantage of the AF2 approach is that multiple models were calculated and those that best-enriched ligands in control calculations were used prospectively. While this likely contributed to the high prospective hit rate, it may also have had unanticipated consequences. One reason that the AF2 model may have been so successful is that it represents an unusually tight orthosteric site, with multiple residues clamping down towards the ligands site versus the experimental structure (Fig. 4b), leading to a binding site whose volume ranges from 513 to 639 Å^3^, depending on the model, substantially smaller than the 746 Å^3^ of the experimental structure used here. This will bias the docking toward the small agonist cations favored by TAAR1. But the AF2 orthosteric site was closed down to such an extent that other plausible ligands, including many of those found in our study, could no longer fit (Fig. 4c). It may have also made it harder to optimize the initial molecules for potency because the ligands had few places to grow. Thus, the potency of the best agonist from the AF2 docking, after optimization, was little changed from the initial docking hit. Meanwhile, the larger orthosteric site of the cryo-EM structure allowed us to optimize compounds into the 1.5 nM range, something that ultimately contributed to their high in vivo potency (below). It may be that the strategy of selecting model structures by ligand enrichment—something widely used in the field, including by ourselves^63–65^—may optimize for initial discovery but lead to challenges for optimization.

### In vivo antipsychotic-like activity of the discovered TAAR1 agonists

To assess the therapeutic potential of compounds ‘3207, ‘2255, and ‘7482, we first measured their pharmacokinetics with a 10 mg/kg intraperitoneal (i.p.) injection in mice (Supplementary Fig. 10 and Supplementary Table 6). All three compounds had high exposure in both brain and cerebrospinal fluid (CSF), the latter of which is a widely used proxy for the fraction unbound (Fu) in the brain. Among them, ‘3207 reached a CSF concentration (C_max_) of 3040 ng/mL (about 18 μM), approximately twice that of ‘2255 and ‘7482. Additionally, the area under the concentration-time curve (AUC) in the CSF was 292,000 ng·min/mL for ‘3207 versus 67,400 ng·min/mL and 123,000 ng·min/mL for ‘2255 and ‘7482, respectively. The half-life (T_1/2_) of ‘3207 in CSF was 56 min, whereas ‘2255 and ‘7482 had T_1/2_ values of 35 and 64 min, respectively. Collectively, ‘3207, ‘2255, and ‘7482 combine high potency at TAAR1 with high brain exposure, leading to receptor coverage (free concentration/EC_50_) between 1000 to 10,000-fold. We therefore evaluated their antipsychotic-like efficacies in pre-pulse inhibition (PPI), a behavioral test that is well-known to be affected by TAAR1 agonists^12,66,67^.

PPI of the acoustic startle reflex assesses the ability of a weak pre-stimulus to suppress the response to a subsequent strong stimulus producing a reduced startle response relative to the acoustic startle stimulus alone. PPI deficits are observed in patients with schizophrenia^68^, and similar disruptions in rodents are used to model psychotic-like states^69^. To induce such deficits, we administered (i.p.) amphetamine (AMPH) to mice—a well-established method for disrupting PPI in rodents. These deficits are known to be reversible by antipsychotic drugs.

**Fig. 5:**
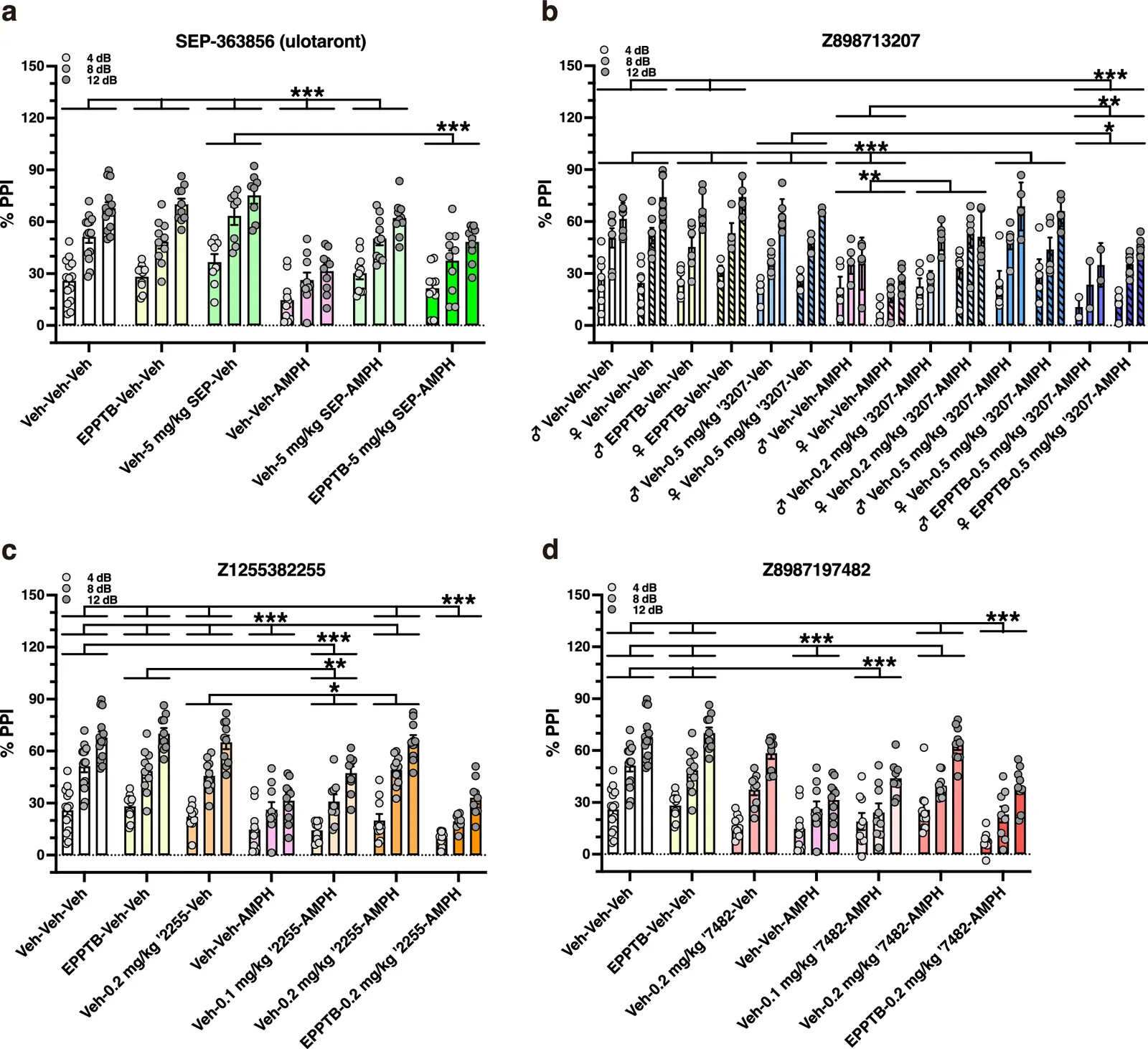
Effects of SEP, ‘3207, ‘2255, and ‘7482 on restoring amphetamine-disrupted prepulse inhibition. Mice were administered the vehicle (Veh) or 0.5 mg/kg EPPTB, followed 10 min later with the Veh, 5 mg/kg SEP, or low or high doses of compounds. After 10 min, mice were given the Veh or 3 mg/kg amphetamine (AMPH). They were placed in the PPI apparatus and tested after 10 min of habituation. Note, to reduce mouse use results from the same mice are shown for the Veh-Veh-Veh, EPPTB-Veh-Veh, and Veh-Veh-AMPH treatments. **a** Effects on PPI following administration of SEP. AMPH-disrupted PPI, 5 mg/kg SEP led to a partial rescue, while EPPTB antagonized this effect; *n* = 8–14 mice/treatment. **b** Effects of ‘3207 on PPI. Overall sex effects were detected, as well as sex effects on individual dB and treatments. Overall treatment effects are shown with AMPH suppressing PPI and this effect was rescued with 0.5 mg/kg ‘3207; the TAAR1 antagonist blocked this response; *n* = 9–14 mice/treatment, **c** PPI responses to ‘2255. A low dose of 0.2 mg/kg ‘2255 restored the AMPH-induced impairment in PPI and this was blocked with EPPTB; *n* = 9–14 mice/treatment. **d** Responses to ‘7482 in PPI. As with ‘2255, 0.2 mg/kg ‘7482 normalized the AMPH-disrupted PPI and this effect was antagonized with EPPTB; *n* = 9–14 mice/treatment. The data are presented as means and standard errors of the mean and were analyzed with Levene’s tests for homogeneity of variance, repeated-measures ANOVA with or without Greenhouse-Geisser corrections, followed by Bonferroni tests; *p < 0.05, **p < 0.01, and ***p < 0.001, *vs*. the indicated group(s). The full statistics are located in Supplementary Table 7 and the numbers of mice per sex and treatment are found in Supplementary Table 8. Source data are provided as a Source data file.

Mice were injected (i.p.) either with vehicle (Veh) or 0.5 mg/kg EPPTB, a TAAR1 antagonist^7^. Ten minutes later they received the Veh, 5 mg/kg SEP-363856 (SEP), or different doses of the compounds. After 10 min they were given the Veh or 3 mg/kg AMPH and tested in PPI after 10 min. PPI data are expressed in terms of responses to 4, 8, and 12 dB prepulse stimuli that were followed by the 120 dB startle stimulus (Fig. 5a–d; see Supplementary Table 7 for all statistics). These data were analyzed by PPI (dB), sex, and treatment. In all cases, PPI and the PPI by treatment interaction were significant. Additionally, analyses of ‘3207 found the PPI by sex by treatment interaction was also significant; no overall sex effects were detected for SEP or any compounds. While these statistical effects are important (see Supplementary Table 7), presenting the data in a format displaying these differences would further complicate the already complex annotations of the significant differences within the Figures and detract from the overall effects important in demonstrating potential antipsychotic efficacies of the compounds. Hence, because main effects of treatment were significant for SEP and all compounds, the results are presented in this manner. In all cases, the Veh-Veh-AMPH treatment impaired PPI relative to the Veh-Veh-Veh and EPPTB-Veh-Veh controls (Fig. 5a–d). 5 mg/kg SEP, 0.5 mg/kg ‘3207, and 0.2 mg/kg ‘2255, and ‘7482 alone did not affect PPI and were similar to controls, also as expected. The 5 mg/kg dose of SEP partially restored the AMPH-disrupted PPI and the TAAR1 antagonist EPPTB partially blocked this response (Fig. 5a). To demonstrate more robust effects of SEP in rescuing the AMPH effect, a 10 mg/kg dose was used and to show TAAR1 specificity higher doses of EPPTB were tested (Supplementary Fig. 11). Ten mg/kg SEP fully restored the AMPH-disrupted PPI; however, 1 and 10 mg/kg EPPTB alone depressed PPI and 10 mg/kg EPPTB could not block the 10 mg/kg SEP plus AMPH effect (Supplementary Fig. 11). Encouragingly, low doses of ‘3207, ‘2255, and ‘7482 partly reversed the AMPH impairment and higher doses completely normalized the AMPH-disrupted PPI (Fig. 5b–d). Here, the TAAR1 antagonist EPPTB blocked the rescuing effects of 0.5 mg/kg ‘3207 and 0.2 mg/kg ‘2255 or ‘7482 by suppressing PPI to the levels of the Veh-Veh-AMPH treatment alone. Collectively, ‘3207, ‘2255, and ‘7482 restored PPI due to the AMPH-induced impairment, with ‘2255 and ‘7482 being more efficacious than ‘3207 in this response, likely reflecting their higher potencies at TAAR1. Regardless, all three compounds normalized PPI in the AMPH mouse model.

Besides PPI, null and startle activities were also evaluated for SEP and the three agonists. Null activity was consistently low in the Veh-Veh-Veh, EPPTB-Veh-Veh, and Veh-SEP/compound-Veh groups compared to the Veh-Veh-AMPH and EPPTB-SEP/compound-AMPH treatments (Supplementary Fig. 12a–d). Activity was enhanced with “2255 and ‘7482, and sex effects were identified with ‘3207. For pulse or startle activities, the patterns of responses were similar among SEP, ‘2255, and ‘7482. Here activities were lower with the Veh-SEP-AMPH, Veh-0.5 mg/kg ‘3207-AMPH, and Veh-0.5 mg/kg ‘7482-Veh treatments compared to Veh-Veh-AMPH (Supplementary Fig. 13). In the ‘2255 and ‘7482 experiments, 0.2 mg/kg of the compounds increased startle activities and the same dose with AMPH reduced these activities below the levels of controls (Supplementary Fig. 13c, d). Overall sex effects were observed with all compounds where startle was higher in males and females. More specific effects were noted with ‘3207 where startle to Veh-0.1 mg/kg ‘3207-AMPH and Veh-0.2 mg/kg ‘3207-AMPH was reduced in females compared to males, as well as to the Veh-Veh-Veh treatment in both sexes and to other treatments within females (Supplementary Fig. 13b).

Besides PPI and associated activities, we also evaluated the potential for inducing catalepsy, a serious adverse drug reaction of many current antipsychotics. At doses that rescue AMPH-disrupted PPI, mice treated (i.p.) with 0.5 mg/kg ‘3207, 0.2 mg/kg ‘2255 or ‘7482 showed no observable catalepsy at 60 or 90 min, unlike those treated with the classic dopamine receptor antagonist haloperidol (1 mg/kg i.p.) (Supplementary Fig. 14). This result suggests that the discovered agonists have a favorable catalepsy-related safety profile versus classic anti-dopaminergics. Based on values for PPI in mice from the literature^66^, these molecules—with substantial activity at doses as low as 0.2 mg/kg—are at least as potent and arguably more potent than TAAR1 drugs like ulotaront.

## Discussion

Four observations from this study merit emphasis. First, library docking and structure-guided optimization found agonists with nanomolar potency on TAAR1 and with sub-µM activity on 5-HT1A, making them more potent both in vitro and apparently in the mouse than ulotaront. Second, only agonists were found. This may reflect the choice of an active-state structure of the TAAR1 for docking, where the orthosteric site contracts around the agonist, selecting for smaller agonist-like molecules and discriminating against larger antagonist-like molecules. Consistent with this view, a previous docking campaign versus AlphaFold2 structures of TAAR1^36^, where the orthosteric site contracted even more, had even higher agonist hit-rates, even though the overall TAAR1 model was in an inactive state. While the compact orthosteric site of the AlphaFold2 model may have improved hit-rates, it may also have restricted the ability to optimize for more potent leads because it was overly compact, something perhaps selected for in optimizing the docking parameters against the AlphaFold2 structure. Third, the docking predictions here were largely confirmed by the subsequent cryo-EM structures, which may template further optimization of these compounds. Fourth, three of the discovered agonists potently restored AMPH-disrupted PPI without the catalepsy that is a key liability for many of the classic dopamine receptor antagonists. These TAAR1 agonists may possess a broader therapeutic index than typical approved antipsychotic drugs for treating acute psychosis.

Several caveats bear mentioning. Although we found agonists with improved potency for both TAAR1 and 5-HT_1A_ receptors, their primary target remains TAAR1, with only moderate potency against 5-HT_1A_. While the discovered agonists are topologically dissimilar to known TAAR1 ligands, they share physical features—and in some cases, core scaffold elements—likely due to the receptor’s small orthosteric site. Finally, the in vivo activities of ‘3207, ‘2255, and ‘7482 were only evaluated in the PPI model of AMPH-disruption for acute psychosis. Their potential effects on other behavioral phenotypes, as well as other adverse drug reactions, remain unexplored.

These limitations should not obscure the core findings from this study. Large library docking against TAAR1 followed by structure-based optimization revealed a series of low-nanomolar TAAR1 agonists with topologies unrelated to known ligands. Targeting the active state of the receptor successfully biased toward agonist discovery. Cryo-EM structures largely supported the docking predictions. The favorable physical properties of the discovered TAAR1 agonists, something insisted upon in the choice of library and docking hits, led to molecules with high CNS exposure and behavioral changes consistent both with improving sensorimotor gating and reducing the side effects of catalepsy. With structures and pharmacology differing from known investigational drugs against TAAR1, these results support the further optimization of these agonists and, more broadly, a structure-based approach to ligand discovery for this interesting receptor^33,36^.

## Methods

### Ethics

Adult male and female C57BL/6J mice (#000664; Jackson Laboratories, Bar Harbor, ME) were used. Mice were housed at 3–4 per cage in a temperature- and humidity-controlled room with a 14:10 h (lights on at 0600 hours) light-dark cycle and provided with food and water ad libitum. All studies were conducted with a protocol (#A069-23-04) approved by the Duke University Institutional Animal Care and Use Committee, and investigations and procedures were performed under guidelines from the NIH for The Care and Use of Laboratory Animals and from ARRIVE.

### Molecular docking against TAAR1

In the docking, we targeted the active-state structure of TAAR1 represented by the cryo-EM structure in PDB 8w8a. The receptor was protonated assuming a pH of 7.4 using Epik and PROPKA in 2022-released Maestro. Energy grids for the different terms of the DOCK3.8 scoring function were pre-generated—the van der Waals term based on the AMBER force fields using CHEMGRID^70^, Poisson-Boltzmann–based electrostatic potentials using QNIFFT^42^, and the context-dependent ligand desolvation using SOLVMAP^40^. The resulting grids were evaluated for their ability to enrich known TAAR1 ligands over property-matched decoys. We chose 14 known TAAR1 agonists and used the DUDE-Z pipeline^46^ to generate 700 decoys. After optimization of the dielectric boundaries for the Poisson-Boltzmann electrostatic and the GB/SA desolvation grids, adjusted logAUC values of 34.1 were achieved. We also docked against an “extrema” set from the DUDE-Z web server (http://tldr.docking.org) to ensure that molecules with extreme physical properties were not enriched^45,46^. We found close to 80% monocations were enriched among the top 1000 ranking molecules, with a logAUC of 15.4. Subsequently, 65 million “fragment-like” molecules (molecular weights ≤250 Da and clogP ≤3.5) from the ZINC22 database (https://cartblanche22.docking.org) were docked against TAAR1 using DOCK3.8. On average, 19,140 orientations and 408 conformations were sampled per molecule demanding 39,993 core hours or less than two days on 1000 cores.

Once the screen was completed, the 382,767 molecules with scores ≤−33 kcal/mol were filtered for key interactions with D103, Y294, F267, F268, I104, and F186 and for dissimilarity to known ligands; no molecules with Tanimoto coefficients (Tcs) > 0.35 to known TAAR1 ligands in ChEMBL, using ECFP4 fingerprints, were accepted. Then the remaining 95,128 molecules were clustered by LUNA 1024-length binary fingerprints with a Tc = 0.25, resulting in 2462 clusters. From these 66 compounds were chosen by human inspection and prioritized for synthesis, of which 55 compounds were successfully made at Enamine.

### Boltz-2 modeled inactive structure

Because no inactive-state experimental structure of TAAR1 state was available, we turned to Boltz-2^57^ to model the inactive-state, as bound to the antagonist, RT-7470-44^58^. MSAs were generated using the mmseqs2 server, and structure prediction was performed with Boltz-2 (v2.0.3) using the default settings (3 recycling steps, 200 sampling steps, 1 diffusion sample, step size of 1.638). The predicted structure was then used to generate docking energy grids following the same procedure as the active-state structure.

### Hit optimization

Potential analogs of the agonists emerging from docking were sought in SmallWorld (NextMove Software, Cambridge, UK) searches of a 48 billion make-on-demand libraries^71^. The resulting analogs were docked to the TAAR1 and evaluated for fit. Compounds were also designed by exploring single atom changes to the parent agonists that were synthetically accessible at Enamine. Compound purities were at least 90% and most active compounds were at least 95% (assessed by LC/MS and NMR).

### GloSensor cAMP accumulation assay

The Promega GloSensor cAMP Assay^50^ was performed to monitor the real-time cAMP variation induced by the TAAR1 or 5-HT_1A_R. Full-length TAAR1 and 5-HT1AR coding sequences were subcloned from PRESTO-Tango plasmids into the pcDNA3.1 expression vector and transfected into HEK293T cells. The cloning primers used were as follows: 5-HT1AR forward, 5′-ttctgtagacaatagatcgataccggtggacgcaccccacccagcc-3′; 5-HT1AR reverse, 5′-gtatcgatctattgtctacagaacttgcacttaattatctttttaaagg-3′; TAAR1 forward, 5′-gaattgtctagctagatcgataccggtggacgcaccccacccagcc-3′; and TAAR1 reverse, 5′-gtatcgatctagctagacaattcgagaaagagtttacaccg-3′. Before transfection, the cells were cultured in DMEM (Gibco) with 10% FBS (Thermo Scientific), 100 IU/ml penicillin, and 100 mg/ml streptomycin at ∼70% confluency in six-well plates. After 16 h, cells were transiently transfected following the Lipofectamine 3000 Reagent transfection system (Thermo Fisher) with 0.5 μg receptor and 1.5 μg GloSensor-22F (Promega). After 24 h, cells were serum-starved in DMEM supplemented with 1% dialyzed FBS (dFBS), 100 IU/ml penicillin, and 100 mg/ml streptomycin. After 16 h, transfected cells were digested and transferred into a 96-well plate with warmed CO_2_–Independent media (Gibco) containing 100 μM Beetle luciferin (Promega) for 1 h. One μL of 100x test compounds at various concentrations were added and incubated for 10 min at room temperature. For the 5-HT_1A_R, an additional 10 μL Forskolin was added to the cells at a final concentration of 5 μM. Luminescence measurements were recorded on a CLARIOstar plate reader and monitored for 12 min. All observations were analyzed with GraphPad Prism v.10. Non-linear curve fits were performed using a three-parameter logistic equation (log (agonist versus response)).

### IP1 accumulation assay

IP1 accumulation was measured using the IP-ONE Gq HTRF kit (Cisbio, 621PAPEJ). Briefly, 24 h after transfection, cells were harvested and resuspended in DMEM containing 0.1% BSA at a density of 4 million per milliliter. 5 ml cells were aliquoted into 384-well plate and stimulated with 5 ml compounds in DMEM (0.1% BSA) supplemented with 50 mM LiCl. After incubation at 37 °C for 30 min, 5 ml IP1 d2 reagent and 5 ml IP1 Tb cryptate antibody were added. After incubation at room temperature for another 1 h, HTRF was read on EnVision multiplate reader (PerkinElmer). EC_50_ values for each curve were calculated by Prism 8.0 software (GraphPad Software).

### Expression, complex formation and purification

The TAAR1 complexes with the discovered agonists were expressed in Hi5 insect cells (Invitrogen). Cells were cultured in SIM HF Expression Medium (Sino Biological, MHF1) to a density of 3 × 10^6^ per mL with virus preparations for TAAR1-miniGα_s_ and Gβ_1_γ_2_ at a ratio of 1:1.2. The plasmids were generated as previously described^39^. The infected cells were cultured at 27 °C for 48 h before collection by centrifugation and the cell pellets were stored at −80 °C for future use.

Protein purification proceeded as previously described^39^. Briefly, cell pellets from 2 L culture were thawed at room temperature and resuspended in low salt buffer containing 20 mM HEPES pH 7.4, 100 mM NaCl, 5 mM CaCl_2_, 5 mM MgCl_2_, 10% glycerol, protease inhibitor cocktail (Thermo Fisher). Cell membranes were collected by ultra-centrifugation at 30,000 rpm for 30 min. The membranes were resuspended and then incubated with 1 μM Z1255382255 or 5 μM Z8987197482, and 20 mU mL^−1^ apyrase (NEB) at 4 °C for 1 h. The protein was extracted from the membrane by 20 mM HEPES, pH 7.4, 100 mM NaCl, 5 mM CaCl_2_, 5 mM MgCl_2_, 10% glycerol, 1%(w/v) lauryl maltose neopentyl glycol (LMNG, Anatrace), 0.2% (w/v) cholesteryl hemisuccinate Tris salt (CHS, Anatrace), and stirred for 2.5 h at 4 °C. The supernatant was isolated by centrifugation at 30,000 rpm for 30 min and then incubated overnight at 4 °C with pre-equilibrated TALON IMAC resin (Clontech). After batch binding, the TALON IMAC resin was washed with 10 column volumes of 20 mM HEPES, pH 7.4, 100 mM NaCl, 5 mM CaCl2, 5 mM MgCl2, 30 mM imidazole, 10% glycerol, 0.1% LMNG (w/v), 0.02% CHS (w/v), 1 μM Z1255382255 or 5 μM Z8987197482 and eluted with the same buffer plus 300 mM imidazole. The eluted protein was incubated with 20 μg/ml of Nb35 at 4 °C for another 2 h. The mixture was then purified by SEC using a Superose 6 10/300 GL column (GE Healthcare) in 20 mM HEPES, pH 7.4, 100 mM NaCl, 0.00075% (w/v) LMNG, 0.00025% (w/v) CHS and 100 nM Z1255382255 or 500 nM Z8987197482. The fractions of monomeric complex were collected and concentrated to 5.5–8.0 mg mL^−1^ for electron microscopy experiments.

### Cryo-EM data collection and processing

For cryo-EM grid preparation, samples for Z1255382255 or Z8987197482 were applied individually to EM grids (Quantifoil, 300 mesh Au R1.2/1.3) that were glow-discharged for 45 s and then blotted for 4 s under 100% humidity at 4 °C before being plunged into liquid ethane cooled by liquid nitrogen using a Mark IV Vitrobot (FEI). Cryo-EM imaging was collected on a Titan Krios equipped with a Falcon 4 direct electron detection device at 300 kV at the Shanghai Advanced Electron Microscope Center, Shanghai Institute of Material Medica. Images were taken with a pixel size of 0.73 Å, a defocus ranging from −1.0 to −2.0 μm using the EPU software (FEI Eindhoven, Netherlands). Each micrograph was dose-fractionated to 36 frames with a total dose of 50 e^-^ Å^−2^ on each EER format movie.

All dose-fractioned images were motion-corrected and dose-weighted by MotionCorr2^72^ software, and their contrast transfer functions were estimated by patch CTF estimation in cryoSPARC v3.2.0^73^. For the Z8987197482-TAAR1- Gs complex, particle selection and 2D, 3D classifications were performed using cryoSPARC. The auto-picking process and Topaz extract produced 1,344,129 particles, which were subjected to interactively 2D and 3D classifications, producing a well-defined subset with 137,014 particles. Subsequent non-uniform refinement and local refinement generated a map with an indicated global resolution of 3.12 Å. For the Z1255382255-TAAR1-Gs complex, particle selection and 2D, 3D classifications were performed using cryoSPARC v3.2.0. The Topaz extract process produced 853,711 particles, which were subjected to interactively 2D and 3D classifications, producing 72,449 particles with complete complex and good quality. The particles were further subjected to non-uniform refinement and local refinement generated a map with an indicated global resolution of 3.14 Å.

### Model building and refinement

The active state of TAAR1 from a previous structure (PDB ID: 8W8A) was used as the starting model for model building the TAAR-Gs complexes. Restraints for Z1255382255 or Z8987197482 were generated using Phenix elbow. The cryo-EM model was docked into the electron microscopy density map using Chimera-1.14^49^ followed by iterative manual adjustment and rebuilding in COOT-0.9.6^74^ and ISOLDE-1.2^75^. Restraints for Z1255382255 or Z8987197482 were generated using Phenix.elbow^76^. Real space and reciprocal space refinements were performed using Phenix programs with secondary structure and geometry restraints. The final refinement statistics are provided in Supplementary Table 5. UCSF Chimera-1.14, Chimera X^77^, and PyMOL-2.0 (https://pymol.org/2/) were used to prepare the structural figures in the paper.

### Pharmacokinetics

The pharmacokinetics (PK) of ‘3207, ‘2255, and ‘7482 were investigated by Bienta Enamine Biology Services (Kyiv, Ukraine) in accordance with the study protocols P081724a, P100824a, and P100824b. Compound ‘3207 was formulated in Captisol-Water for injection (40:60, w/v), while compounds ‘2255, and ‘7482 were formulated in saline. For all three studies, a single 10 mg/kg intraperitoneal (i.p.) dose was administered and samples collected at nine time points: 5, 15, 30, 60, 120, 240, 360, 480, and 1440 min. Each of the time-point treatment groups included 3 animals. Mice were injected (i.p.) with 2,2,2-tribromoethanol at the dose of 150 mg/kg prior to drawing the blood. CSF was collected under a stereomicroscope from *cisterna magna* using 1 mL syringes. Blood collection was performed from the orbital sinus in microtainers containing K3EDTA. Animals were euthanized by cervical dislocation after the blood samples collection. Each blood sample was centrifuged for 10 min at 3000 rpm. Brain samples were collected and weighed. The samples were immediately processed, flash-frozen, and stored at −70 °C until subsequent analysis.

Plasma samples (40 μL) were mixed with 200 μL of internal standard solution [IS(90)]. After mixing by pipetting and centrifuging for 4 min at 6000 rpm, 0.25–2 μL of each supernatant was injected into LC-MS/MS system. The solution with compound Phenibut (200 ng/ml in water-methanol mixture 1:9, v/v) was used as the internal standard [IS(90)] for quantification of ‘3207, the standard CLK-IN-T3 (400 ng/ml in water-methanol mixture, 1:9, v/v) was used for ‘2255, and Ondansetron (50 ng/mL in water- acetonitrile mixture 1:9, v/v) was used for ‘7482. Brain samples were homogenized with 5 volumes of IS(80) solution using glass beads (115 mg ± 5 mg) in the Bullet Blender® homogenizer for 30 s at speed 8. After this, the samples were centrifuged for 4 min at 14,000 rpm, and 0.25–2 μL of each supernatant was injected into LC-MS/MS system. The solution of compound Phenibut (200 ng/ml in water-methanol mixture 1:4, v/v) was used as the internal standard [IS(80)] for quantification of ‘3207, CLK-IN-T3 (400 ng/ml in water-methanol mixture, 1:4, v/v) for ‘2255, and Ondansetron (50 ng/mL in water- acetonitrile mixture 1:4, v/v) for ‘7482 were used as the IS for the quantifications in the brain and CSF samples. CSF samples (3 μL) were mixed with 75 μL of IS(80) solution. After mixing by pipetting and centrifuging for 4 min at 6000 rpm, 0.5–6 μL of each supernatant was injected into LC-MS/MS system. Analyses of plasma, brain and CSF samples were conducted by the Bioanalytical Laboratory personnel at Enamine/Bienta. The concentration of test compounds in samples was determined using high performance liquid chromatography/tandem mass spectrometry (HPLC-MS/MS). The HPLC consisted of a Shimadzu HPLC system comprised 2 isocratic pumps LC-20AD, an autosampler SIL-20AC HT, a sub-controller FCV-14H and a degasser DGU-20As. Mass spectrometric analysis was performed using an API 5000 (triple-quadrupole) instrument from AB Sciex (Canada) with an electro-spray (ESI) interface. The data acquisition and system control were performed using Analyst 1.6.3 software from AB Sciex. The concentrations of the test compounds below the lower limit of quantitation (LLOQ = 5 ng/ml for plasma; 5 ng/g for brain, and 10 ng/ml for CSF samples) were designated as zero. Results were analyzed using noncompartmental, bolus injection or extravascular input analysis models in WinNonlin 5.2 (PharSight). Data below the LLOQ were presented as missing values to improve the validity of T_1⁄2_ calculations. For each treatment condition, the final concentration values at each time-point were analyzed for outliers using Grubbs’ test with the level of significance set at *p* < 0.05.

### In vivo behavioral studies

Adult male and female C57BL/6J mice (90 days old; #000664; The Jackson Laboratory, Bar Harbor, ME) were used. Mice were housed at 3–4 per cage in a temperature- and humidity-controlled room with a 14:10 h (lights on at 0600 hours) light-dark cycle and provided with food and water ad libitum. Sex was included as a biological variable in the study design and statistical analyses. Sample sizes for all experiments are provided in Supplementary Table 8 and source data files. All PPI studies were conducted under protocols approved by the Duke University Institutional Animal Care and Use Committee, and investigations and procedures were performed as ARRIVE guidelines.

The drugs consisted of *D*-amphetamine hemisulfate salt (AMPH, #A5880; Sigma-Aldrich, St. Louis, MO), EPPTB (SML-1350; Sigma-Aldrich), and SEP-363856 (T12899L; Target Mol, Wellesley Hills, MA), and the compounds included Z8987163207 (‘3207), Z1255382255 (‘2255), and Z8987197482 (‘7482). In the experiments, the groups consisted of three separate administrations (i.p.) separated by 10 min each consisting of the vehicle (Veh) or 0.5 mg/kg EPPTB; followed by the Veh, 5 mg/kg SEP, or two different doses of each of the compounds, followed with the Veh or 3 mg/kg AMPH. The injection series consisted of the following groups: Veh-Veh-Veh and EPPTB-Veh-Veh controls; Veh-SEP-Veh, and Veh-compounds-Veh (“high” dose) also controls; the Veh-Veh-AMPH positive control; and the Veh-SEP/compound-AMPH and EPPTB-SEP/compound-AMPH test groups. The first and second Veh consisted of *N,N*-dimethyllacetamide (final volume 0.5%; Sigma-Aldrich) that was brought to volume with 5% 2-hydroxypropoyl-β-cyclodextrin (Sigma-Aldrich) in water (Mediatech Inc., Manassas, VA), while the third Veh was water. The AMPH was reconstituted in water. All drugs were administered (i.p.) in a 5 mL/kg volume.

### Prepulse inhibition (PPI)

PPI of the acoustic startle response was conducted using SR-LAB chambers (San Diego Instruments, San Diego, CA)^78^. Mice were injected with Veh or 0.5 mg/kg EPPTB and returned to their home-cages. Ten minutes later they were given Veh; 5 mg/kg SEP; 0.1 or 0.2 ‘2255 or ‘7482; or 0.2 or 0.5 mg/kg ‘3207 and returned to their home-cages. Finally, 10 min later the mice received the Veh or 3 mg/kg AMPH and were placed into a Plexiglas holding tube and put into the test apparatus. After 10 min of habituation to a white noise background (64 dB), testing began. A test session consisted of 42 trials with 6 null trials, 18 pulse-alone trials, and 18 prepulse-pulse trials. Null trials were composed of exposure to the white noise background, startle or pulse trials contained of 40 ms bursts of 120 dB white-noise, and prepulse-pulse trials were composed of 20 ms prepulse stimuli that were 4, 8, or 12 dB above the white-noise background (6 trials/dB), and were followed by the 120 dB pulse stimulus 100 ms later. Testing began with 10 pulse-alone trials followed by combinations of the prepulse-pulse and null trials and was terminated with 10 pulse-alone trials. PPI responses were calculated as %PPI = [1–(pre-pulse trials/startle-only trials)]*100.

All statistical analyses were performed with IBM SPSS Statistics 29 programs (IBM, Chicago, IL). The data are displayed as means and standard errors of the mean. Note, to reduce the numbers of mice used, the same raw data for the Veh-Veh-Veh, EPPTB-Veh-Veh, and the Veh-Veh-AMPH groups were used within comparisons with the SEP, ‘3207, ‘2255, and ‘7482 treatments. The PPI data were analyzed with three- and two-way repeated-measures ANOVA (RMANOVA), with or without Greenhouse-Geisser corrections (due to violation of homogeneity of variance in Levene’s test), followed by Bonferroni corrected pair-wise comparisons. Male and female mice were analyzed together with sex incorporated as a factor in the analyses. No consistent sex-specific effects were observed across PPI-related measures. The null and pulse activities were analyzed by two- and one-way ANOVA followed with Bonferroni corrections. If Levene’s test for homogeneity of variance was violated, the data were analyzed by Mann-Whitney (sex) and/or one-way Kruskal–Wallis (treatment) tests followed with Bonferroni post-hoc tests. A *p* < 0.05 was considered significant. The statistics are located in Supplementary Table 7, all results were plotted using GraphPad Prism 10.4.1, and numbers of mice per sex and treatment are displayed in Supplementary Table 8.

### Catalepsy test

Thirty minutes after an i.p. injection of the compounds or haloperidol, mice were placed onto a vertical wire mesh and the latency to move all four paws was recorded^56^.

### Chemical synthesis and analysis

#### General considerations

All compounds active or tested for activity against TAAR1 in this study are commercially available from Enamine Ltd (www.enamine.net) and are identified by their registry numbers throughout (“Z”-numbers), including make-on-demand molecules. For the latter, all solvents were treated according to standard methods. ^1^H NMR spectra were recorded at 400 or 500 MHz (Varian or Bruker spectrometers). ^1^H chemical shifts are calibrated using residual nondeuterated solvent DMSO or CHCl_3_: δ = 2.50 ppm or 7.29 ppm, respectively. Coupling constants are given in Hz. LC/MS analysis was performed utilizing Agilent 1200 Series LC/MSD system with DAD/ELSD (column Zorbax SB-C18 1.8 µm 4.6 × 15mm; solvent A (water, 0.1% formic acid) and solvent B (acetonitrile, 0.1% formic acid); gradient 0–100% solvent B, run time, 1.8 min; flow rate, 3 mL/min) and Agilent LC/MSD SL (G6130A), SL (G6140A) mass-spectrometer (APCI mode). All the LC/MS data were obtained using positive/negative mode switching. Analytical results for active compounds may be found in the Supplementary Information.

### Reporting summary

Further information on research design is available in the Nature Portfolio Reporting Summary linked to this article.

## Supplementary information

Supplementary Information

Reporting Summary

Transparent Peer Review file

## Source data

Source Data

## Data Availability

The ZINC22 compound library is available to all at (https://cartblanche22.docking.org/), accessed in July 2023. Compounds can be ordered directly on the Enamine store website at (https://next.enaminestore.com/) or by contacting Enamine and providing the Z-number or the compound SMILES. The PDB entry for the TAAR1 cryo-EM structure used for docking calculations is 8W8A. The ‘2255-TAAR1-Gs model was deposited to the Protein Data Bank under accession code 9VZ3 and the map was deposited to the EMDB under accession code EMD-65477. The ‘7482-TAAR1-Gs model was deposited to the Protein Data Bank under accession code 9VZ2 the map was deposited to the EMDB under accession code EMD-65476. A list of all de novo compounds and their 2D structure can be found in Supplementary Tables 1 and 3. All compounds may be ordered from Enamine. Source data are provided with this paper as a Source data file. Source data are provided with this paper.

## Code availability

DOCK3.8 has been previously published and is available to all academics without charge (https://dock.compbio.ucsf.edu/DOCK3.8/).
