# Supplementary material for: Structure based discovery of antipsychotic-like TAAR1 agonists
Source: Nat Commun. 2026 Sep 17;17:10902. doi: 10.1038/s41467-026-77484-2 (PMC13677718; doi:10.1038/s41467-026-77484-2)
Supplement: Supplementary file 1 [file 41467_2026_77484_MOESM1_ESM.pdf]

Supplementary Information for:

Structure based discovery of antipsychotic-like TAAR1 agonists

Sijie Huang[1#], Heng Liu[2#], Yujin Wu[1#], Joao M. Braz[3], Divya Kranthi[1], Brendan W. Hall[1], Xinyue Zhang[2], Dmytro S. Radchenko[4], Yurii S. Moroz[4,5,6], John J. Irwin[1], Allan I. Basbaum[3], H. Eric Xu[2,7*], William C. Wetsel[8*] & Brian K. Shoichet[1*]

# These authors contributed equally to this work.

* Corresponding authors: bshoichet@gmail.com (Brian K. Shoichet), william.wetsel@duke.edu (William C. Wetsel), eric.xu@simm.ac.cn (H. Eric Xu)

This file includes:

Supplementary figures 1 to 14

Supplementary Tables 1 to 8

Supplementary Methods

Supplementary Figures

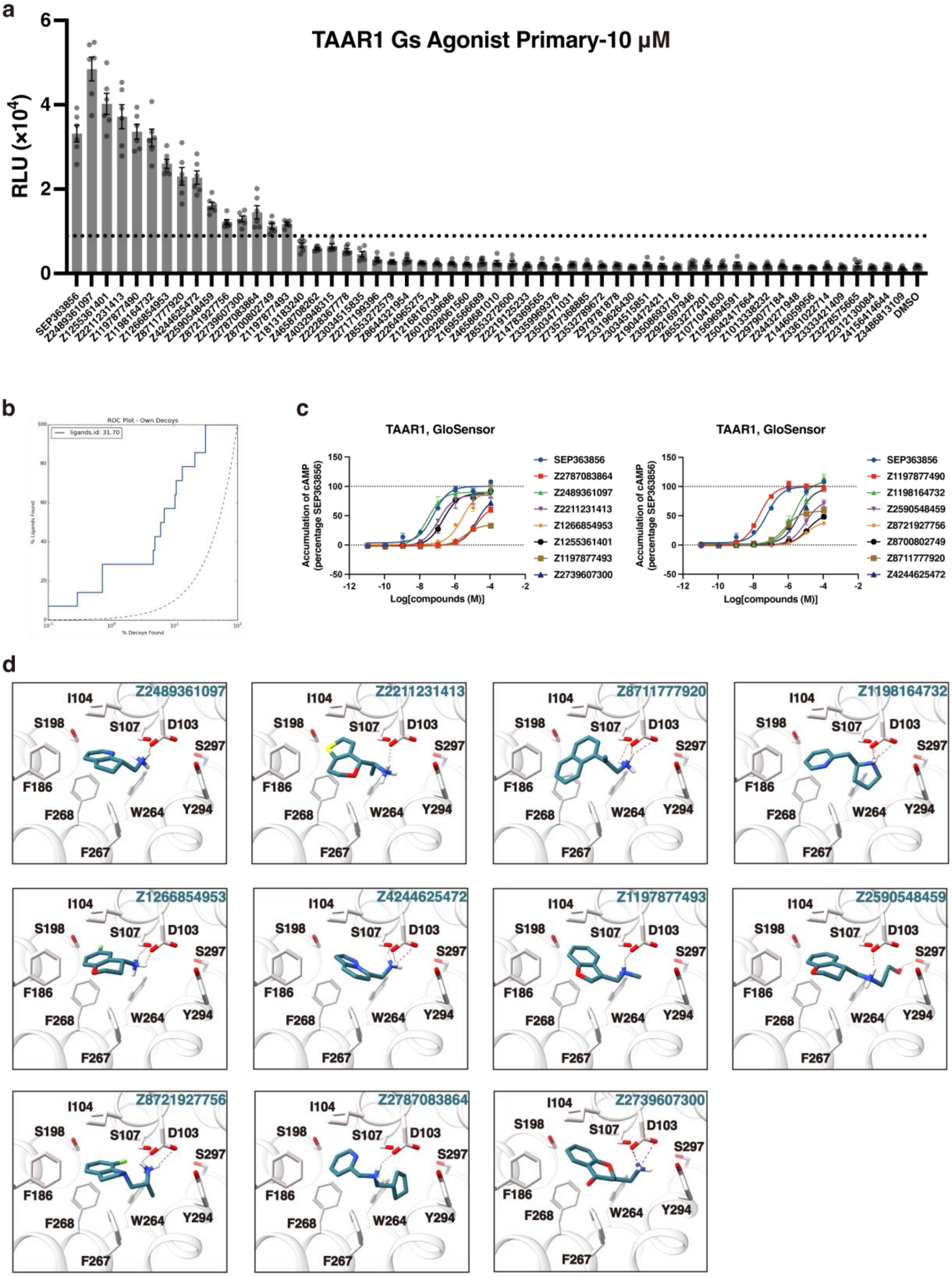

Supplementary Fig. 1. Functional screening of the predicted molecules against the TAAR1 and the docked poses of the initial hits from the larger screen. a, GloSensor responses of 55 predicted molecules at 10 μM. Hit rates were defined as having more than a 30% GloSensor response compared with SEP-363856 at 10 μM. **b,** Log-transformed ROC plots that compare the rate of ligand identification versus property-matched decoys for the TAAR1 receptor. **c,** GloSensor response of the initial hits at 10 μM. Data are mean ± SEM. from three independent experiments (n = 3). **d,** Docked poses of the initial hits from the 65-million screen of compounds. Source data are provided as a Source Data file.

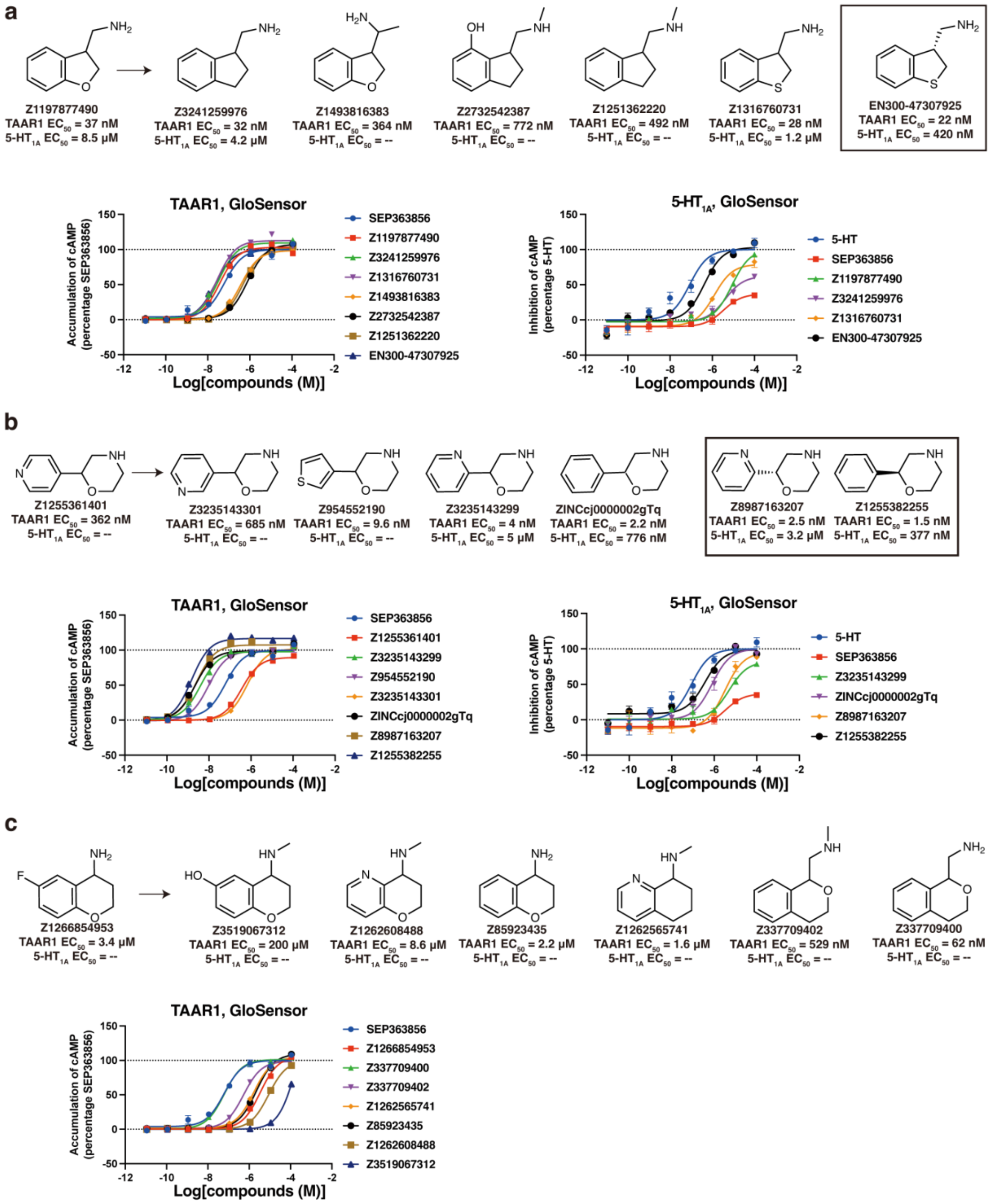

Supplementary Fig. 2. Additional structure-activity relationships around the initial hits from a large-scale docking campaign. a, Additional SAR for optimization of compound ‘7490. Data are mean ± SEM from three independent experiments (n = 3). **b,** Additional SAR for optimization of compound ‘1401. Data are mean ± SEM from three independent experiments (n = 3). **c,** SAR for optimization of compound ‘4953. Data are mean ± SEM from three independent experiments (n = 3). “--” indicates that the value could not be determined due to insufficient compound activity. Source data are provided as a Source Data file.

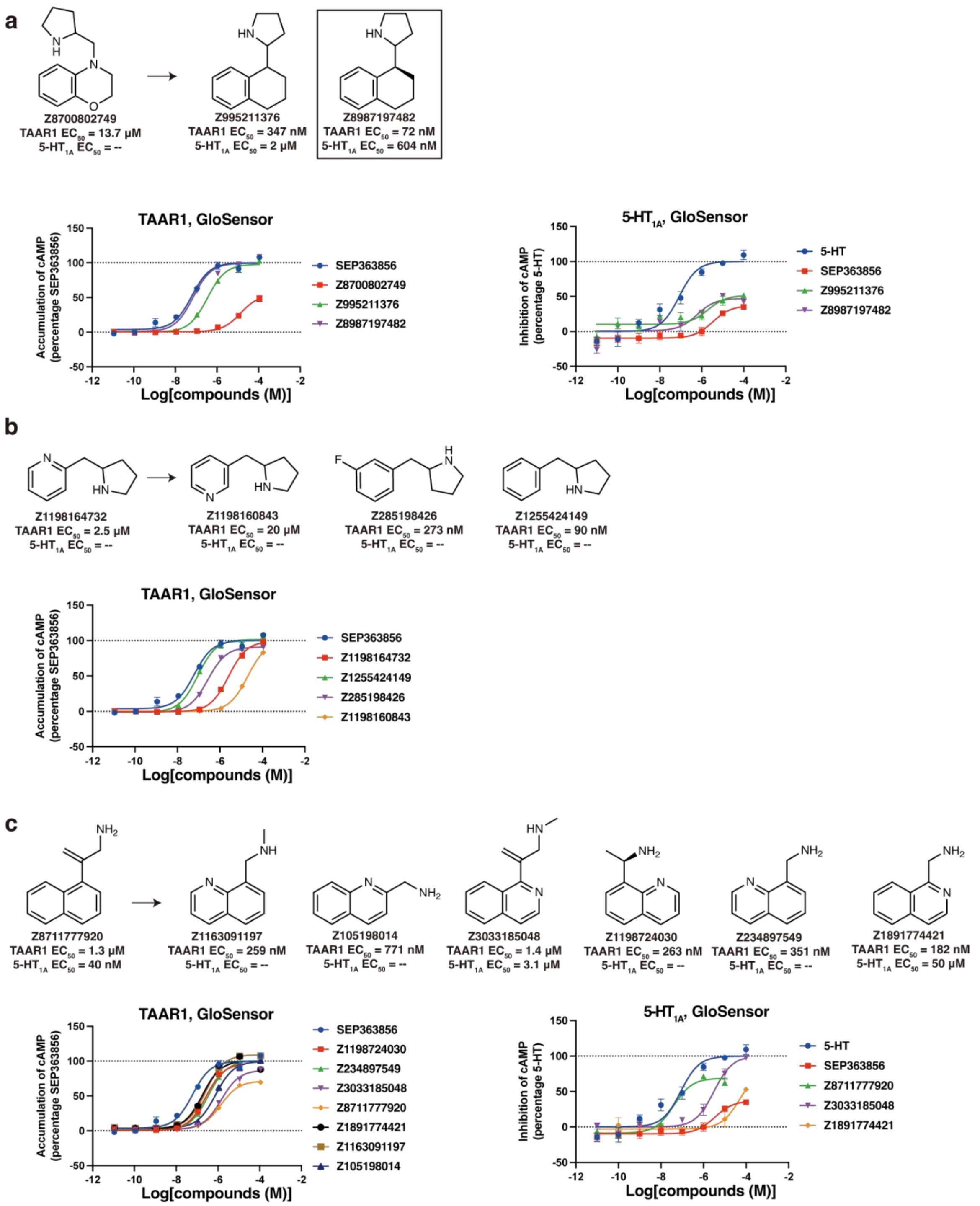

Supplementary Fig. 3. Additional structure-activity relationships around the initial hits from a large-scale docking campaign. a, Additional SAR for optimization of compound ‘2749. Data are mean ± SEM from three independent experiments (n = 3). **b,** SAR for optimization of compound ‘4732. Data are mean ± SEM from three independent experiments (n = 3). **c,** SAR for optimization of compound ‘7920. Data are mean ± SEM from three independent experiments (n = 3). “--” indicates that the value could not be determined due to insufficient compound activity. Source data are provided as a Source Data file.

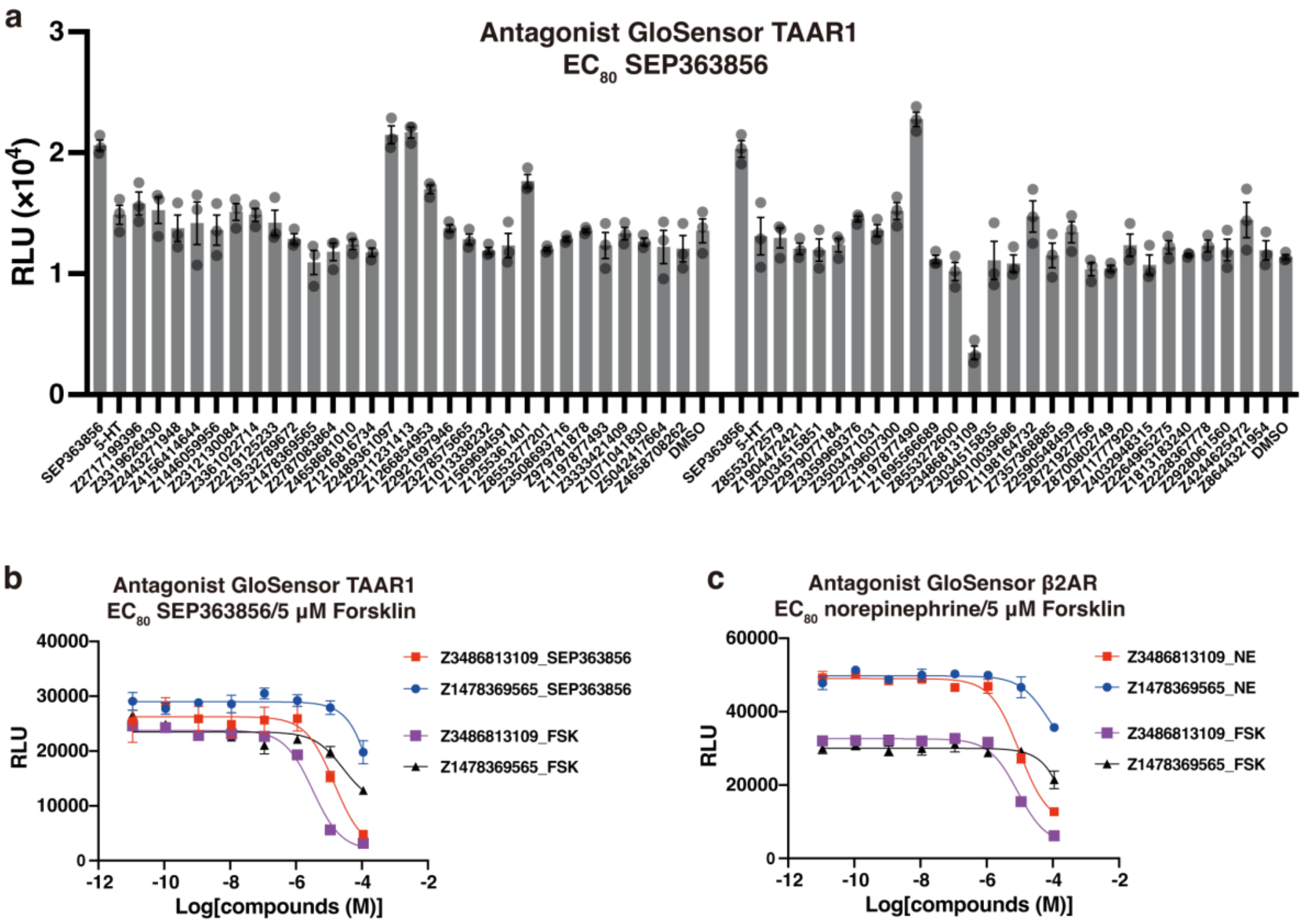

Supplementary Fig. 4. Functional screening of predicted molecules against the TAAR1 in antagonist mode. a, GloSensor responses of 55 predicted molecules at 10 μM in antagonist mode (cells were treated with EC_{80} concentrations of agonist SEP363856). Data are mean ± SEM from three independent experiments (n = 3). **b,** GloSensor assay in antagonist mode with cells expressing TAAR1 and treated with EC80 concentrations of agonist SEP363856 or 5 μM forskolin. Data are mean ± SEM from three independent experiments (n = 3). **c,** GloSensor assay in antagonist mode with cells expressing β2AR and treated with EC80 concentrations of the agonist norepinephrine or 5 μM forskolin. Data are mean ± SEM from three independent experiments (n = 3). Source data are provided as a Source Data file.

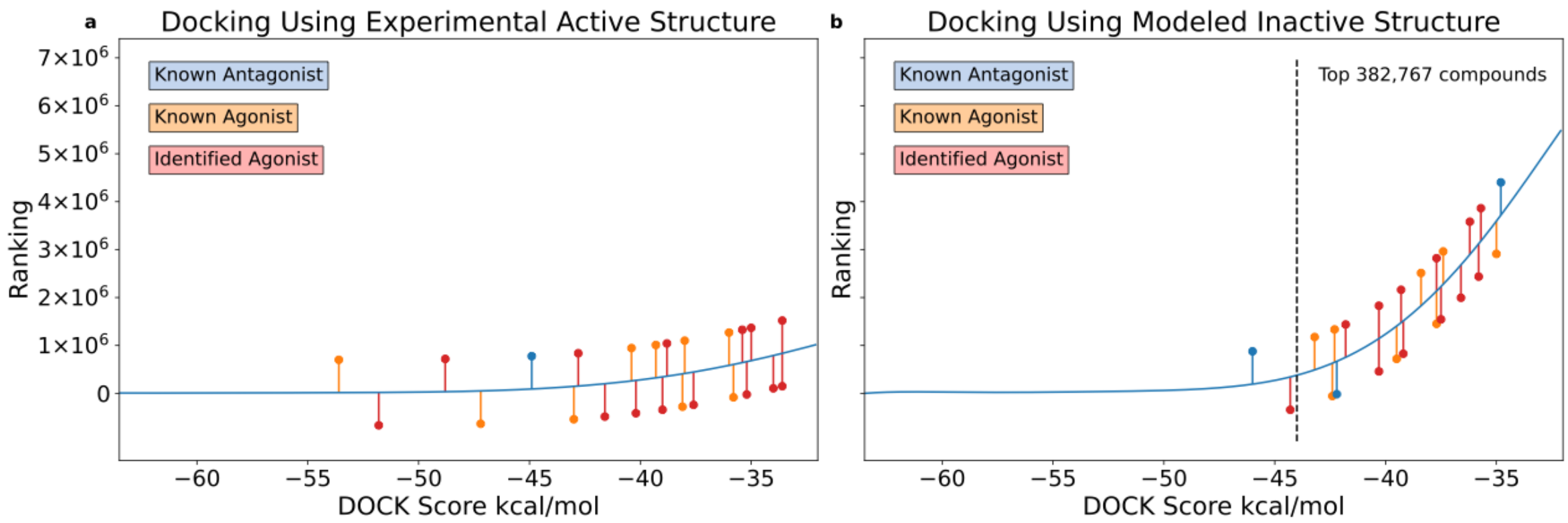

Supplementary Fig. 5. Comparison of large-scale docking results using the experimentally active and modeled inactive TAAR1 structures. a, Docking results using the experimental active structure, showing the rankings and docking scores of known agonists, antagonists and identified agonists. **b,** Docking results using the modeled inactive structure (generated by Boltz-2), showing the rankings and docking scores of agonists and antagonists. The known antagonist, known agonist and identified agonist are shown in blue, orange and red respectively. The top 382,767 ranking position is labelled **(b)**.

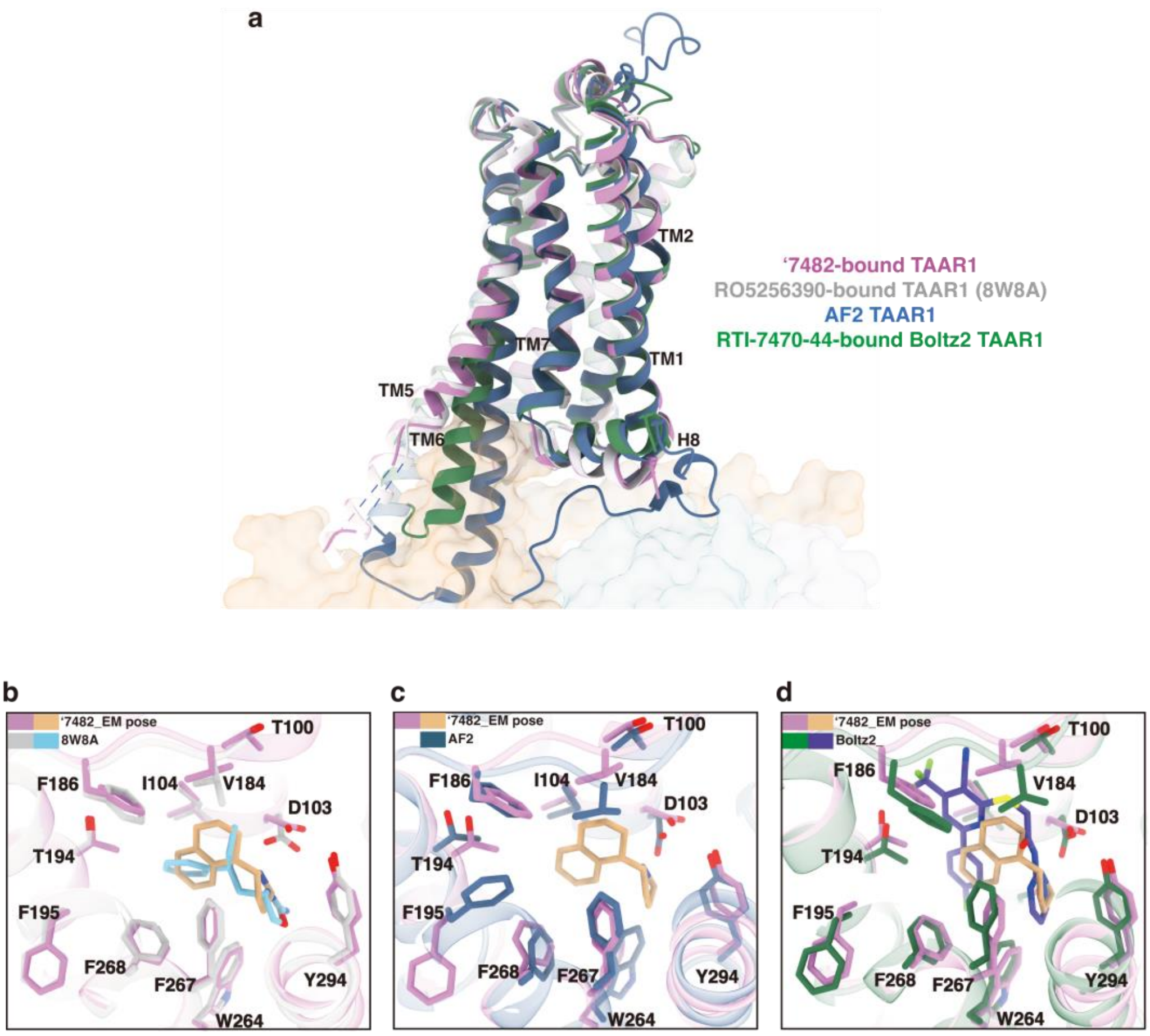

Supplementary Fig. 6. Structural comparison of experimental TAAR1 agonist-bound conformations, an AlphaFold2 model, and a Boltz-2 antagonist co-folding inactive model. **a,** Superposition of the experimental '7482-bound TAAR1 structure (plum), the previously reported RO5256390-bound TAAR1 structure (light gray, PDB 8W8A), an AlphaFold2 (AF2) TAAR1 model (slategrey), and the antagonist RTI-7470-44 co-folding TAAR1 Boltz-2 inactive model (green). The Gs protein shown corresponds to the '7482-bound structure. When displayed with the Gs surface, both the AF2 and Boltz-2 TAAR1 models exhibit steric clashes at the cytoplasmic end of TM6, reflecting their inactive-like conformations. **b,** Superposition of the experimentally determined '7482 pose (burlywood) with the RO5256390 (skyblue)-bound TAAR1 (8W8A) structure in the orthosteric site. The pocket residues in both structures adopt highly similar orientations. **c,** Superposition of the experimentally determined '7482 pose (burlywood) with the AF2-predicted TAAR1 model in the orthosteric site. In the AF2 structure, several pocket-shaping residues, including F195 and V184, rotate inward toward the binding cavity, resulting in an over-collapsed and smaller orthosteric site compared with experimental structures. **d,** Superposition of the '7482 (burlywood)-bound TAAR1 structure with the RTI-7470 (slateblue)-bound Boltz-2 inactive TAAR1 model. In the Boltz-2 model, residue F186 adopts a shifted, more outward orientation relative to the experimental active state, expanding the orthosteric pocket and accommodating the larger antagonist used during co-folding.

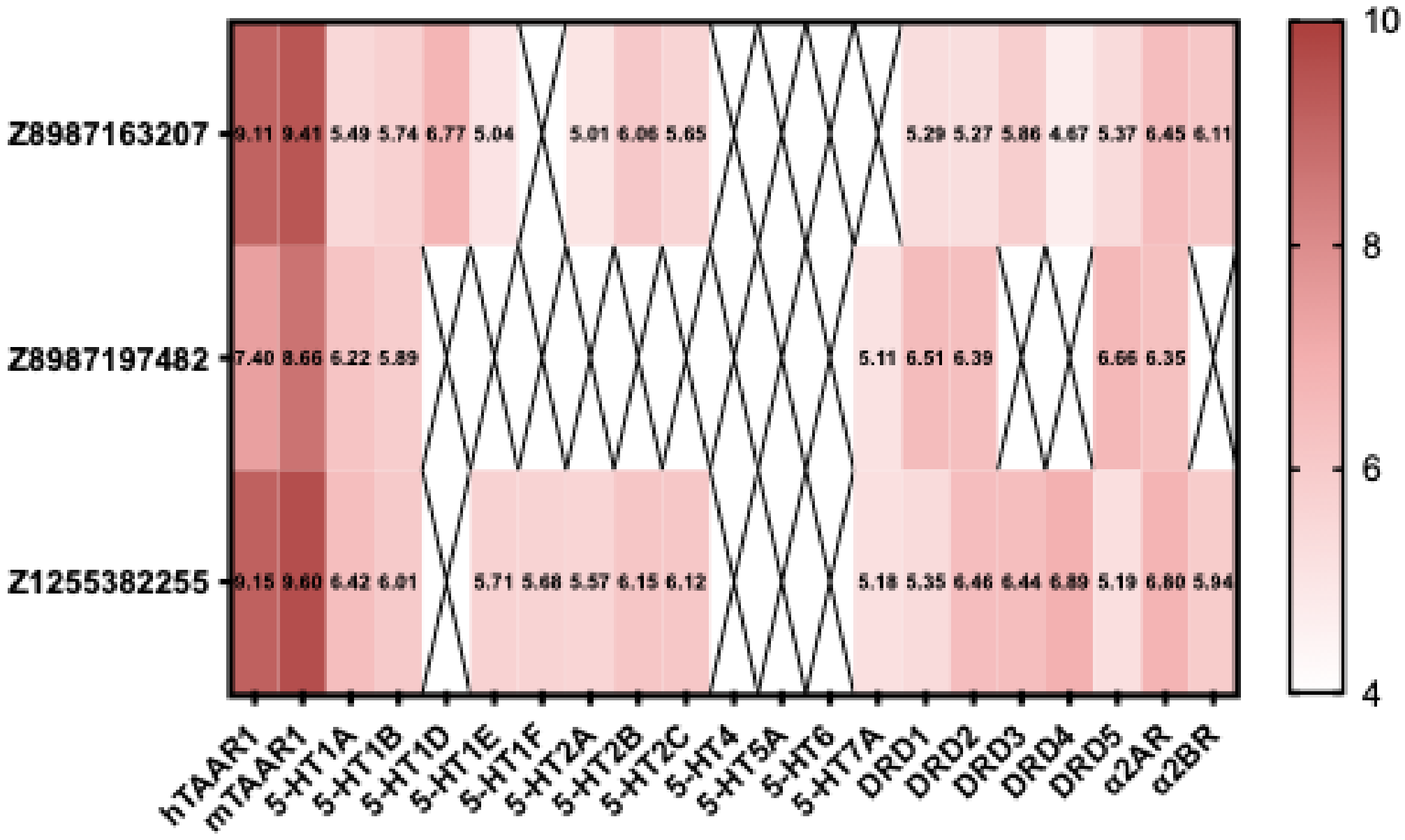

Supplementary Fig. 7. Heatmap of compound activity across selected aminergic receptors. Heatmap showing the activity of ‘3207, ‘7482, and ‘2255 across a panel of related aminergic receptors, displayed as pEC_{50} values obtained from IP1 accumulation or GloSensor cAMP functional assays. “X” denotes either no detectable response or responses for which reliable pEC_{50} values could not be determined within the tested concentration range. Data represent mean values from three independent experiments (n = 3). Source data are provided as a Source Data file.

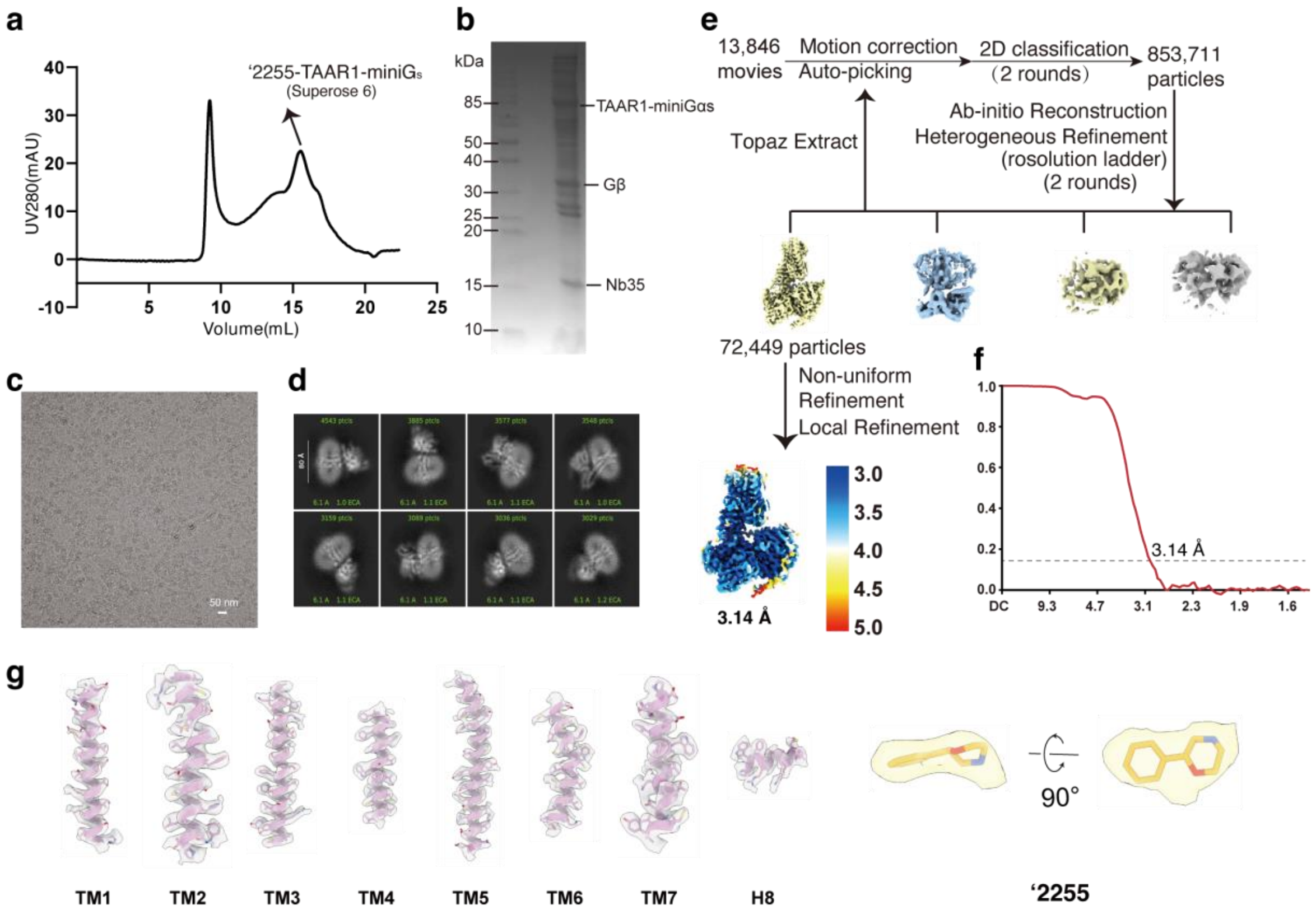

Supplementary Fig. 8. Purification and structural determination of '2255 bound TAAR1-Gs complexes. a, Representative size-exclusion chromatography (SEC) profiles. **b,** SDS-PAGE analysis of the TAAR1-Gs complex activated by '2255. **c-d,** Representative cryo-EM image from 13,846 movies **(c)** and 2D classification averages **(d)** of '2255-TAAR1-Gs. **e,** Cryo-EM data-processing flowcharts of '2255-TAAR1-Gs by cyroSPARC 3.2 and the global density map of '2255-TAAR1-Gs colored by local resolutions. **f,** The Fourier shell correlation (FSC) curves of '2255-TAAR1-Gs. Global resolution of the final processed density map estimated at the FSC = 0.143 is 3.14 Å. **g,** The density maps of helices TM1-TM7 of the transmembrane domain and extracellular loop ECL2 of TAAR1, as well as the α5 helix of Gαs with '2255.

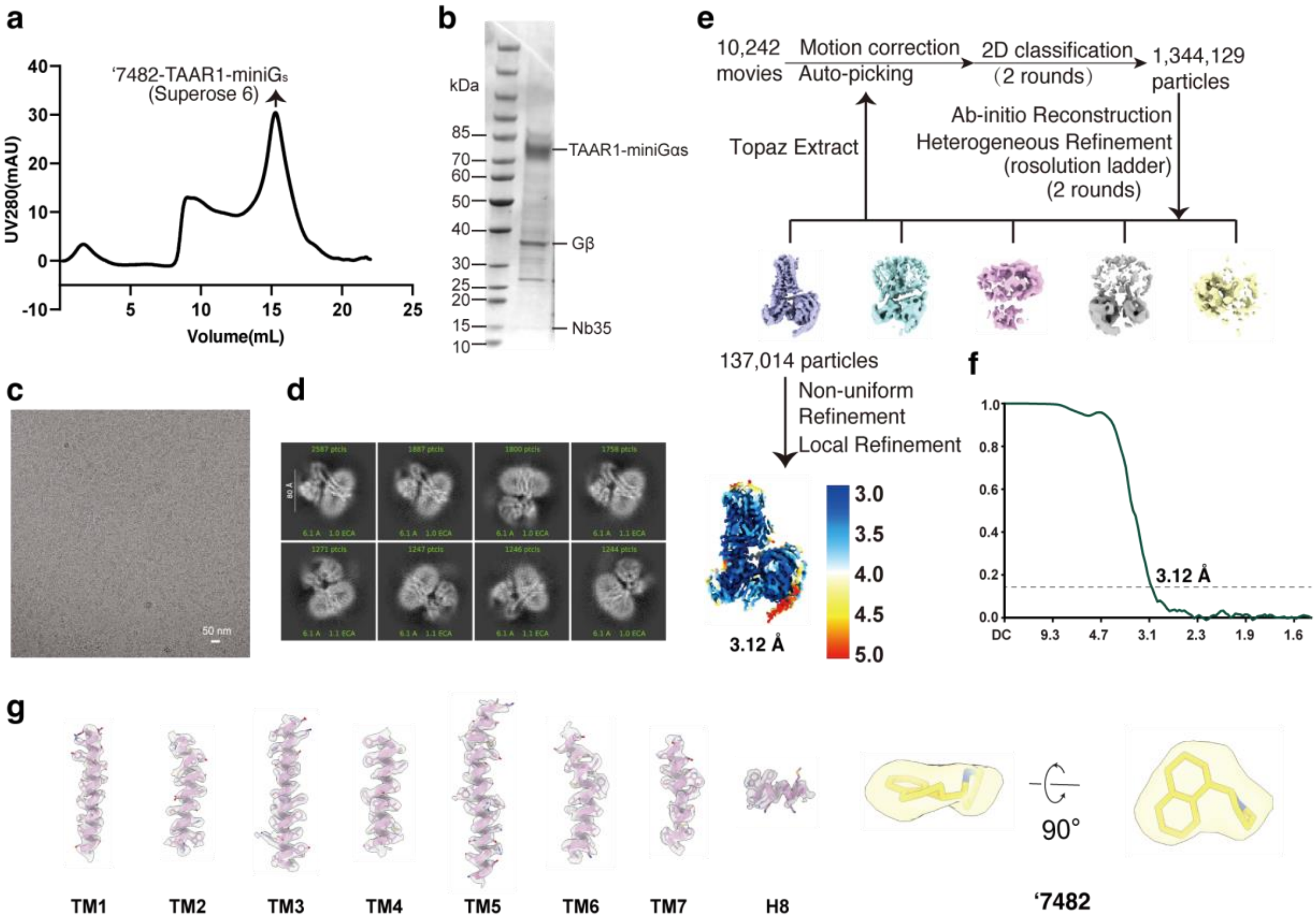

Supplementary Fig. 9. Purification and structural determination of '7482 bound TAAR1-Gs complexes. a, Representative size-exclusion chromatography (SEC) profiles. **b,** SDS-PAGE analysis of TAAR1-Gs complex activated by '7482. **c-d,** Representative cryo-EM image from 10,242 movies **(c)** and 2D classification averages **(d)** of '7482-TAAR1-Gs. **e,** Cryo-EM data-processing flowcharts of '7482-TAAR1-Gs by cyroSPARC 3.2 and the global density map of '7482-TAAR1-Gs colored by local resolutions. **f,** The Fourier shell correlation (FSC) curves for '7482-TAAR1-Gs. Global resolution of the final processed density map estimated at the FSC = 0.143 is 3.12 Å. **g,** The density maps of helices TM1-TM7 of the transmembrane domain and extracellular loop ECL2 of TAAR1, as well as the α5 helix of Gαs with '7482.

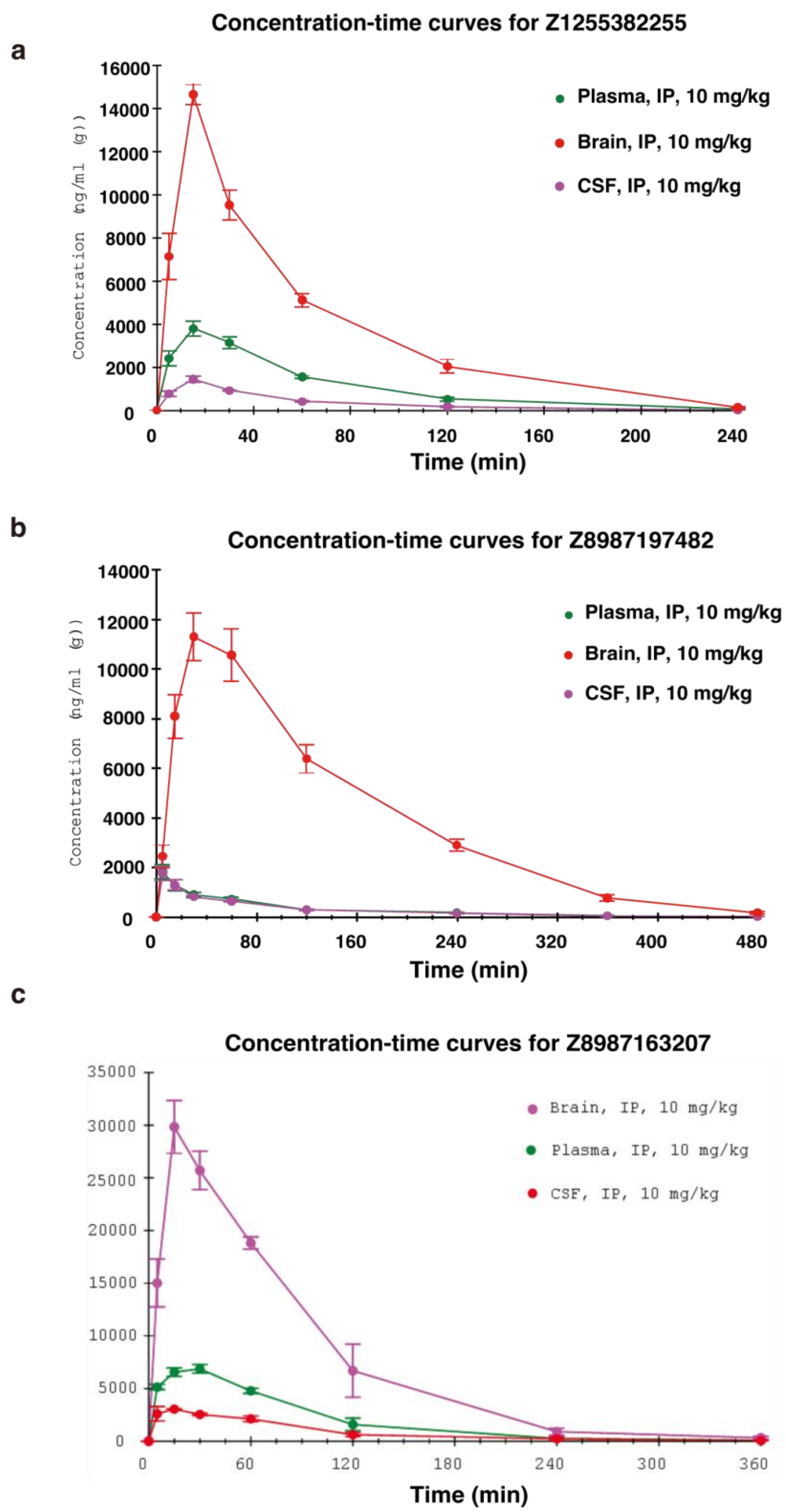

Supplementary Fig. 10. Pharmacokinetic profile of compounds in mice. a, Concentration-time curves of **'2255** in the blood plasma, brain, and cerebrospinal fluid (CSF) after i.p. administration at 10 mg/kg to mice. **b,** Concentration-time curves of **'7482** in the blood plasma, brain, and cerebrospinal fluid (CSF) after i.p. administration at 10 mg/kg to mice. **c,** Concentration-time curves of **'3207** in the blood plasma, brain, and cerebrospinal fluid (CSF) after i.p. administration at 10 mg/kg to mice.

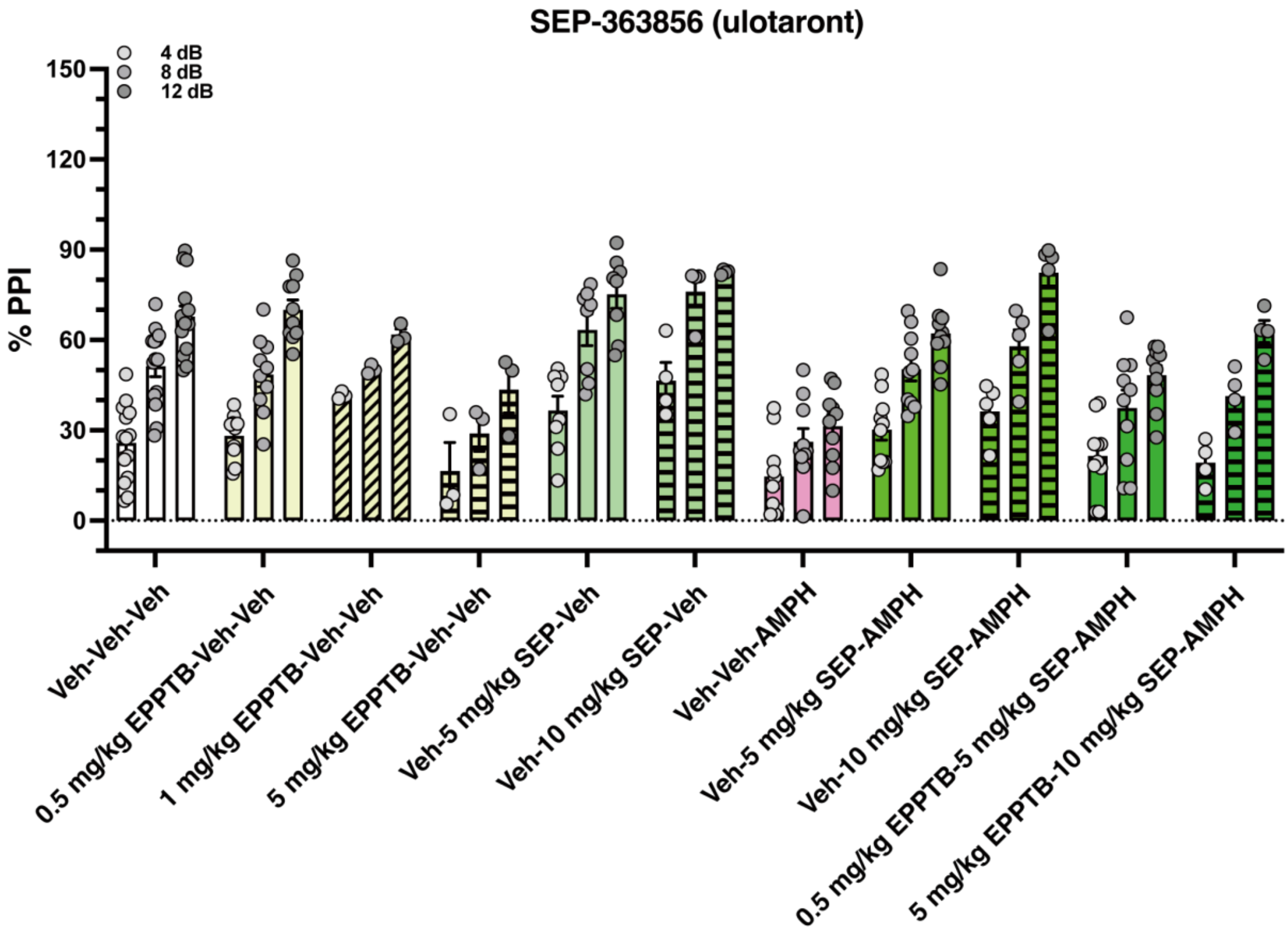

Supplementary Fig. 11. Effects of different concentrations of EPPTB and SEP in prepulse inhibition related to Figure 5a. Effects of additional doses of EPPTB and SEP on PPI. The cross-hatched and diagonal lines represent results from a pilot study with these higher doses; the other bars are duplicated from Figure 5a. The data are presented as means and SEMs.

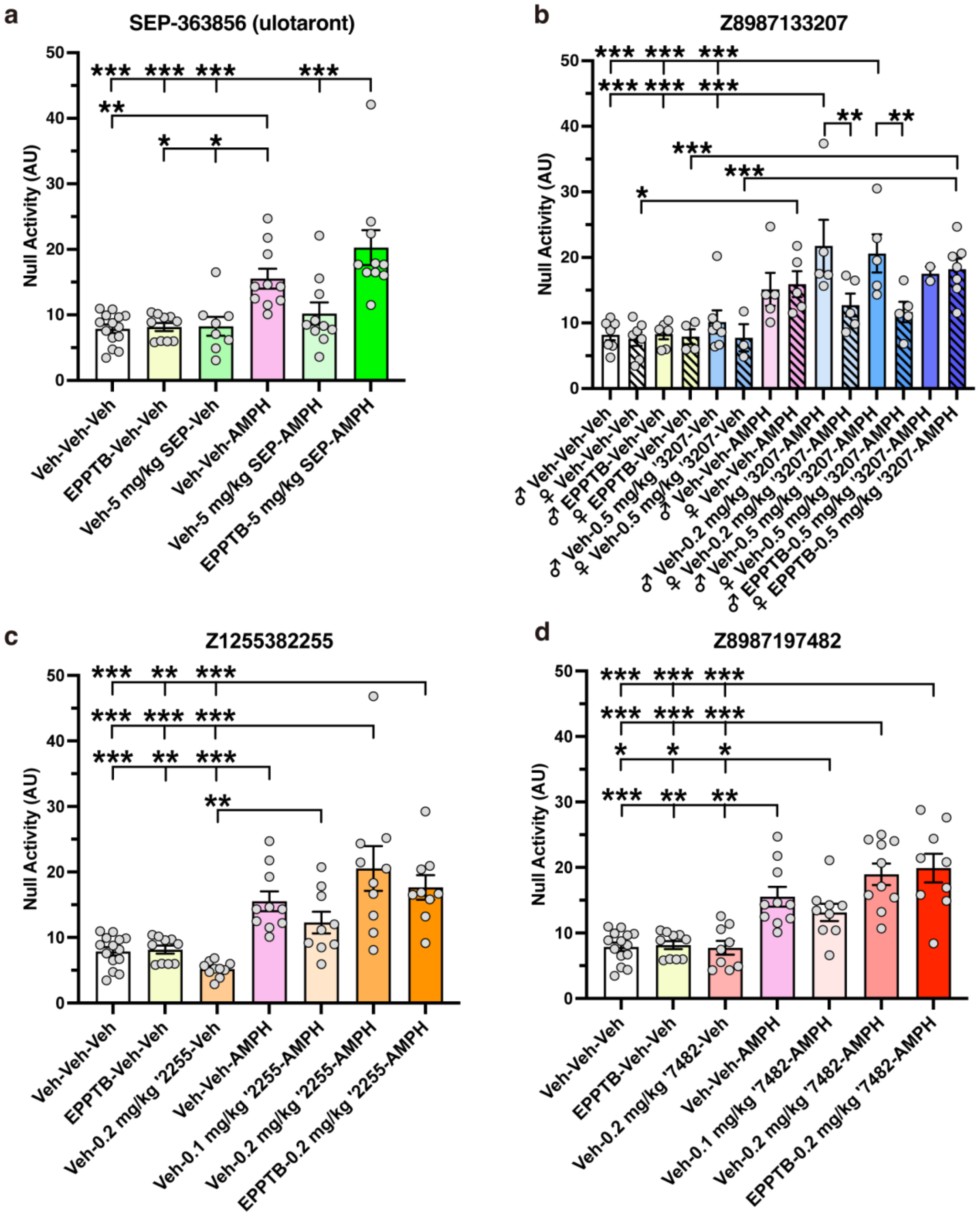

a
SEP-363856 (ulotaront)
Null Activity (AU)
Veh-Veh-Veh
EPPTB-Veh-Veh
Veh-5 mg/kg SEP-Veh
Veh-Veh-AMPH
Veh-5 mg/kg SEP-AMPH
EPPTB-5 mg/kg SEP-AMPH
b
Z8987133207
Null Activity (AU)
♂ Veh-Veh-Veh
♀ Veh-Veh-Veh
♂ EPPTB-Veh-Veh
♀ EPPTB-Veh-Veh
♂ Veh-0.5 mg/kg '3207-Veh
♀ Veh-0.5 mg/kg '3207-Veh
♂ Veh-Veh-AMPH
♀ Veh-Veh-AMPH
♂ Veh-0.2 mg/kg '3207-AMPH
♀ Veh-0.2 mg/kg '3207-AMPH
♂ Veh-0.5 mg/kg '3207-AMPH
♀ Veh-0.5 mg/kg '3207-AMPH
♂ EPPTB-0.5 mg/kg '3207-AMPH
♀ EPPTB-0.5 mg/kg '3207-AMPH
c
Z1255382255
Null Activity (AU)
Veh-Veh-Veh
EPPTB-Veh-Veh
Veh-0.2 mg/kg '2255-Veh
Veh-Veh-AMPH
Veh-0.1 mg/kg '2255-AMPH
Veh-0.2 mg/kg '2255-AMPH
EPPTB-0.2 mg/kg '2255-AMPH
d
Z8987197482
Null Activity (AU)
Veh-Veh-Veh
EPPTB-Veh-Veh
Veh-0.2 mg/kg '7482-Veh
Veh-Veh-AMPH
Veh-0.1 mg/kg '7482-AMPH
Veh-0.2 mg/kg '7482-AMPH
EPPTB-0.2 mg/kg '7482-AMPH

Supplementary Fig. 12. Effects of SEP, ‘3207, ‘2255, and ‘7482 on null activities related to prepulse inhibition in Figure 5. Effects of vehicle (Veh), 0.5 mg/kg EPPTB, 5 mg/kg SEP, 0.2 and 0.5 mg/kg ‘3207, 0.1 and 0.2 mg/kg “2255 and ‘7482, and 3 mg/kg amphetamine (AMPH) on null activities. Note, to reduce mouse use results from the same mice are shown for the Veh-Veh-Veh, EPPTB-Veh-Veh, and Veh-Veh-AMPH treatments. **a,** Null responses to these treatments with SEP. Responses were increased with Veh-Veh-AMPH and EPPTB-5 mg/kg SEP-AMPH; n=8-14 mice/treatment. **b,** Null activities to these treatments with ‘3207. Sex effects were found with Veh-0.2 mg/kg ‘3207-AMPH and Veh-0.5 mg/kg ‘3207-AMPH, as well as within sex. Null responses to Veh-Veh-AMPH and EPPTB-0.5 mg/kg ‘3207-AMPH were augmented in both sexes; n=9-14 mice/treatment. **c,** Effects of these treatments with ‘2255 on null activities. Responses to Veh-Veh-AMPH, Veh-0.1 mg/kg ‘2255-AMPH, Veh-0.2 mg/kg ‘2255-AMPH, and EPPTB-0.2 mg/kg ‘2255-AMPH were increased relative to the other groups; n=9-14 mice/treatment. **d,** The effects of the treatments with ’7482 on null activities were similar to those for ‘2255; n=9-14 mice/treatment. The data are presented as means and SEMs and were analyzed by two- and one-way ANOVA followed with Bonferroni corrections. If Levene’s test for homogeneity of variance was violated, the data were analyzed by Mann-Whitney (sex) and/or one-way Kruskal-Wallis (treatment) tests followed with Bonferroni *post-hoc* tests; *$p<0.05$, **$p<0.01$, and ***$p<0.001$, *vs.* the indicated group(s). The primary statistics are found in Supplementary Table 7 and the numbers of mice per sex and treatment are located in Supplementary Table 8.

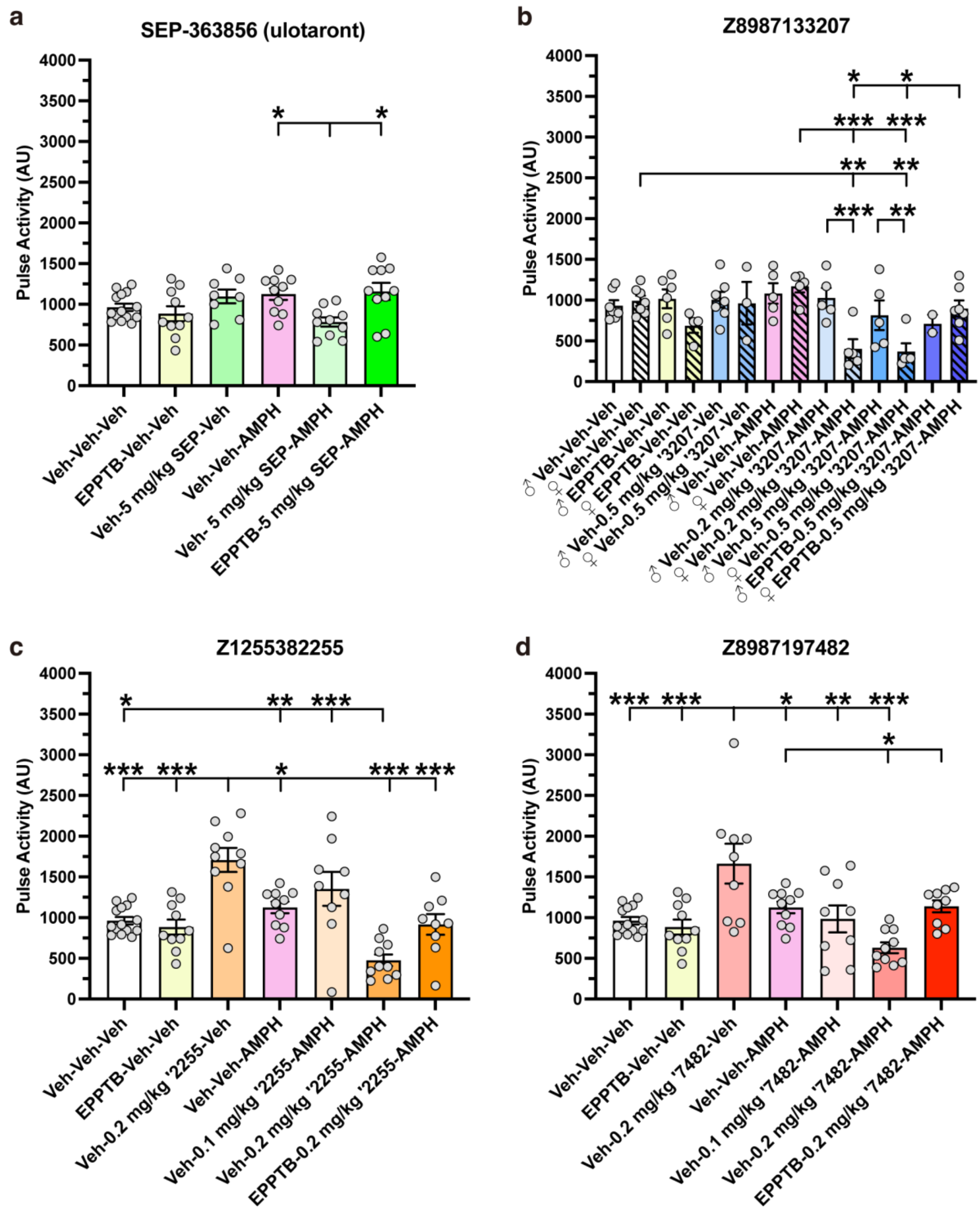
a
SEP-363856 (ulotaront)
Pulse Activity (AU)
Veh-Veh-Veh
EPPTB-Veh-Veh
Veh-5 mg/kg SEP-Veh
Veh-Veh-AMPH
Veh- 5 mg/kg SEP-AMPH
EPPTB-5 mg/kg SEP-AMPH
b
Z8987133207
Pulse Activity (AU)
♂ Veh-Veh-Veh
♀ Veh-Veh-Veh
♂ EPPTB-Veh-Veh
♀ EPPTB-Veh-Veh
♂ Veh-0.5 mg/kg '3207-Veh
♀ Veh-0.5 mg/kg '3207-Veh
♂ Veh-Veh-AMPH
♀ Veh-Veh-AMPH
♂ Veh-0.2 mg/kg '3207-AMPH
♀ Veh-0.2 mg/kg '3207-AMPH
♂ Veh-0.5 mg/kg '3207-AMPH
♀ Veh-0.5 mg/kg '3207-AMPH
♂ EPPTB-0.5 mg/kg '3207-AMPH
♀ EPPTB-0.5 mg/kg '3207-AMPH
c
Z1255382255
Pulse Activity (AU)
Veh-Veh-Veh
EPPTB-Veh-Veh
Veh-0.2 mg/kg '2255-Veh
Veh-Veh-AMPH
Veh-0.1 mg/kg '2255-AMPH
Veh-0.2 mg/kg '2255-AMPH
EPPTB-0.2 mg/kg '2255-AMPH
d
Z8987197482
Pulse Activity (AU)
Veh-Veh-Veh
EPPTB-Veh-Veh
Veh-0.2 mg/kg '7482-Veh
Veh-Veh-AMPH
Veh-0.1 mg/kg '7482-AMPH
Veh-0.2 mg/kg '7482-AMPH
EPPTB-0.2 mg/kg '7482-AMPH

Supplementary Fig. 13. Effects of SEP, '3207, '2255, and '7482 on startle activities related to prepulse inhibition in Figure 5. Effects of vehicle (Veh), 0.5 mg/kg EPPTB, 5 mg/kg SEP, 0.2 and 0.5 mg/kg '3207, 0.1 and 0.2 mg/kg "2255 and '7482, and 3 mg/kg amphetamine (AMPH) on startle activities. Note, to reduce mouse use results from the same mice are shown for the Veh-Veh-Veh, EPPTB-Veh-Veh, and Veh-Veh-AMPH treatments. **a,** Startle responses to these treatments with SEP. Startle to Veh-5 mg/kg SEP-AMPH was reduced relative to Veh-Veh-AMPH and EPPTB-5 mg.kg SEP-AMPH; n=8-14 mice/treatment. **b,** Pulse activities to these treatments with '3207. As with null activities, sex effects were found with Veh-0.2 mg/kg '3207-AMPH and Veh-0.5 mg/kg '3207-AMPH, as well as within females; n=9-14 mice/treatment. **c,** Effects of these treatments with '2255 on pulse activities. Startle was increased with Veh-0.2 mg/kg '2255-Veh and Veh-0.1 mg/kg '2255-AMPH, while it was decreased with Veh-0.2 mg/kg '2255 compared to most other treatments; n=9-14 mice/treatment. **d,** Effects of the treatments with '7482 on startle responses. As with '2255, pulse responses were augmented with Veh-0.2 mg/kg '7482-Veh and reduced with Veh-0.2 mg/kg '7482-AMPH relative to some additional treatments; n=9-14 mice/treatment. The data are presented as means and SEMs and were analyzed by two- and one-way ANOVA followed with Bonferroni corrections. If Levene's test for homogeneity of variance was violated, the data were analyzed by Mann-Whitney (sex) and/or one-way Kruskal-Wallis (treatment) tests followed with Bonferroni *post-hoc* tests; *$p<0.05$, **$p<0.01$, and ***$p<0.001$, *vs.* the indicated group(s). The primary statistics are in Supplementary Table 7 and the numbers of mice per sex and treatment are located in Supplementary Table 8.

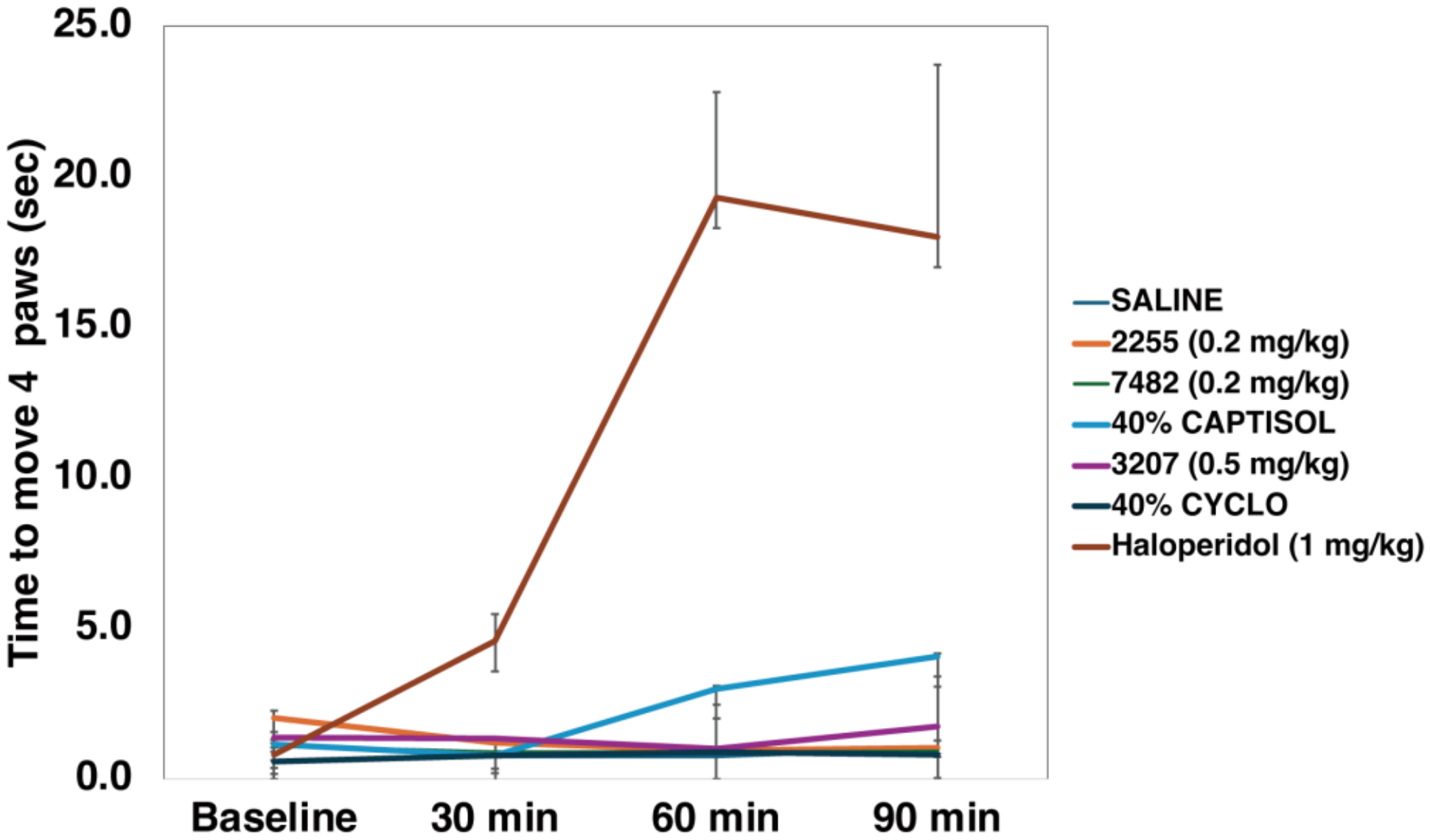

Supplementary Fig. 14. Catalepsy is low for the three optimized agonists versus haloperidol. Baseline catalepsy was assessed and this response was evaluated 30, 60, and 90 min later using the most efficacious dose of each compound in the PPI experiment. The data are presented as means and standard errors of the mean; n=5 for the groups.

Supplementary Table 1. Initial actives from the docking screen against TAAR1.

ID	Structure	Rank	pEC_{50}
Z1197877490	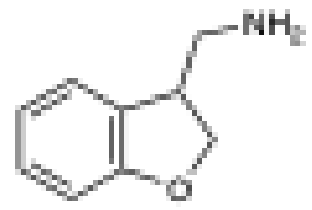	146807	7.66 ± 0.01
Z2489361097	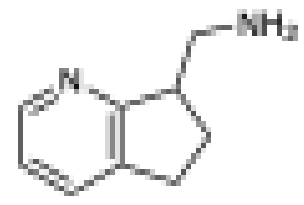	143284	7.47 ± 0.19
Z2211231413	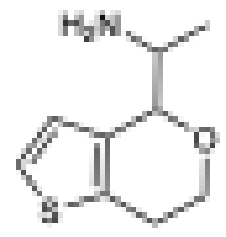	349943	6.93 ± 0.13
Z1255361401	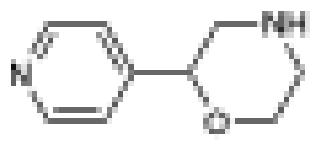	348940	6.52 ± 0.23
Z8711777920	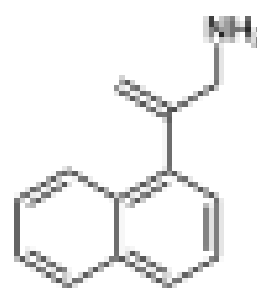	285048	6.09 ± 0.21
Z1198164732	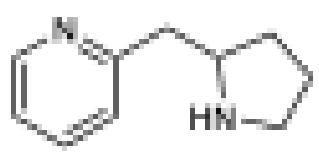	6186	5.53 ± 0.28
Z1266854953	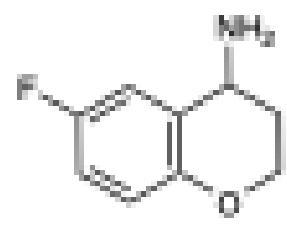	267272	5.60 ± 0.14
Z4244625472	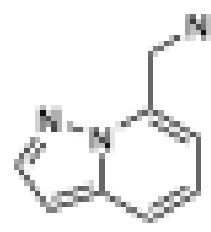	84791	5.41 ± 0.3
Z8700802749	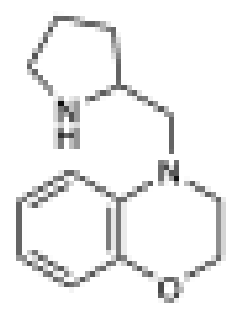	14217	4.85 ± 0.13
Z1197877493	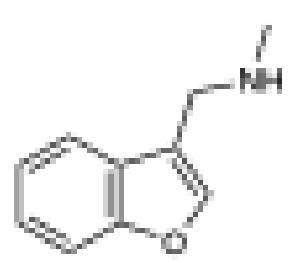	64456	5.36 ± 0.2
Z2590548459	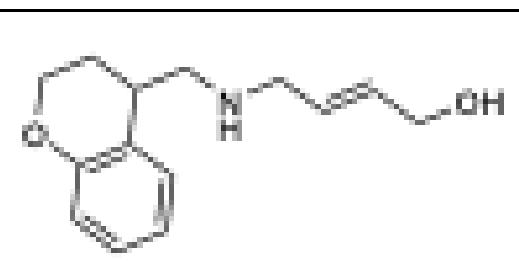	185741	5.01 ± 0.16
Z2739607300	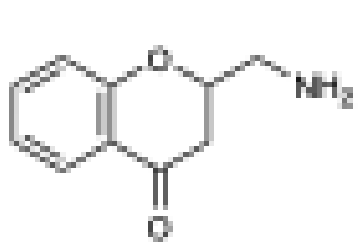	332574	4.76 ± 0.05

Z8721927756	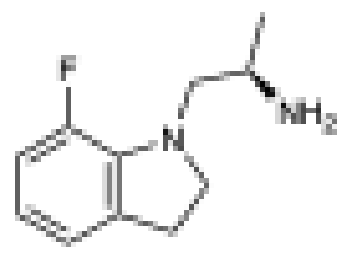	111801	5.03 ± 0.18
Z2787083864	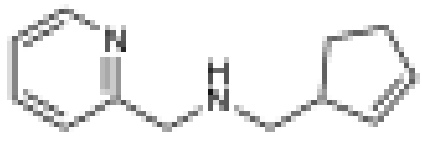	277264	4.8 ± 0.07

Supplementary Table 2. Structural similarity of hits and lead compounds to reference TAAR1 ligands.

ID	Structure	Tc[a]	ChEMBL ligands
Z1197877490	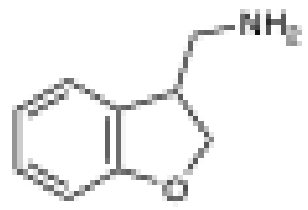	0.32	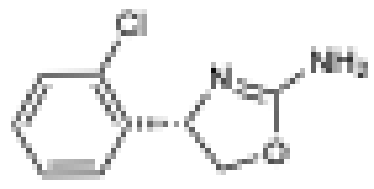
Z2489361097	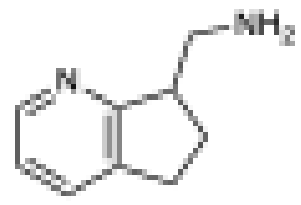	0.27	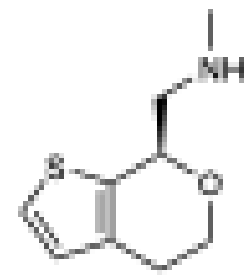
Z2211231413	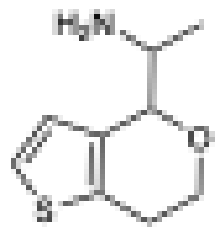	0.31	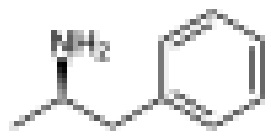
Z1255361401	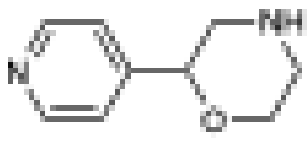	0.18	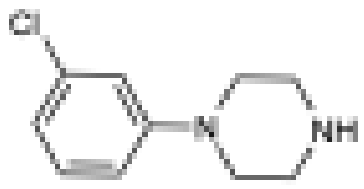
Z8711777920	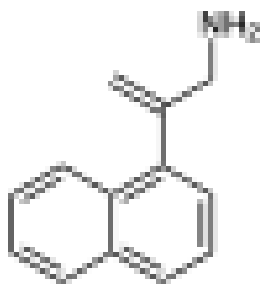	0.26	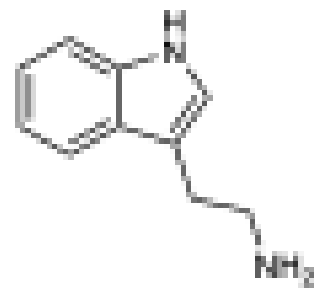
Z1198164732	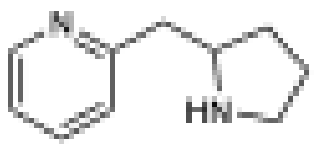	0.33	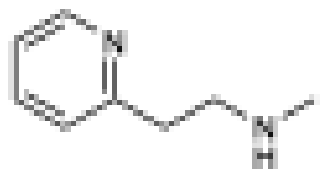
Z1266854953	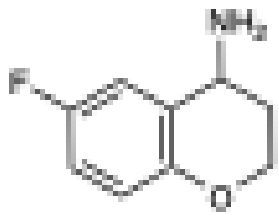	0.28	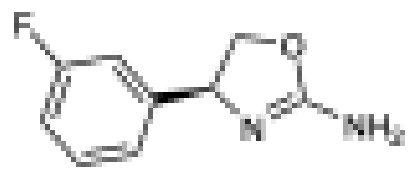
Z4244625472	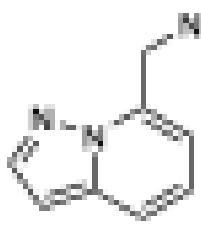	0.26	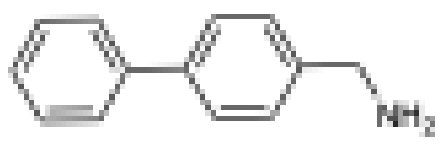
Z8700802749	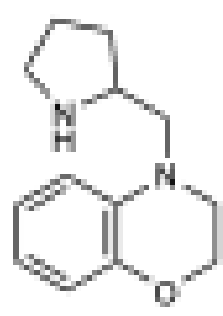	0.26	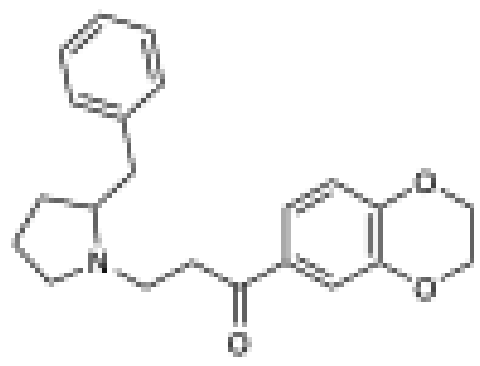
Z1197877493	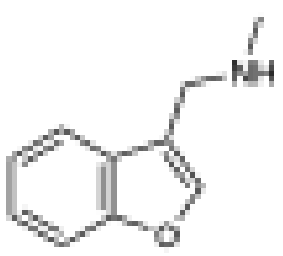	0.31	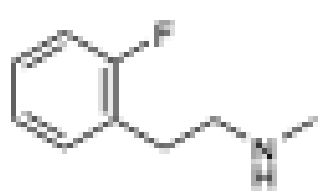
Z2590548459	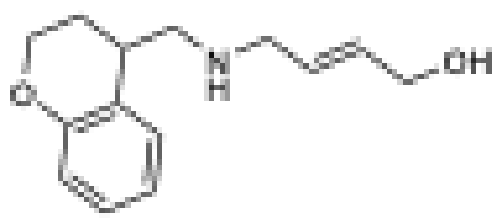	0.25	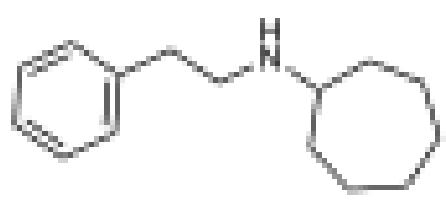

Z2739607300	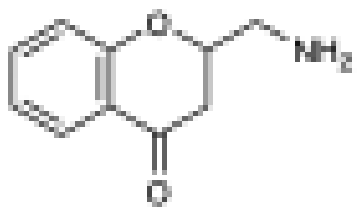	0.27	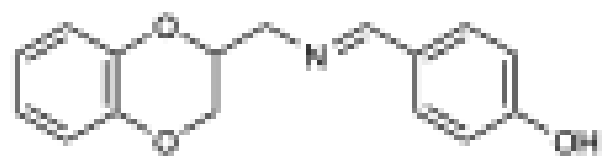
Z8721927756	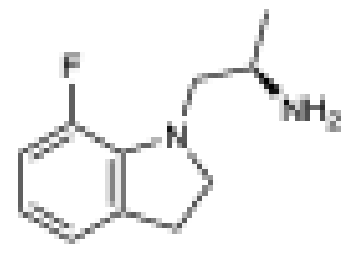	0.31	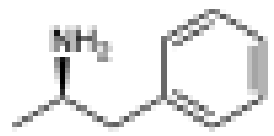
Z2787083864	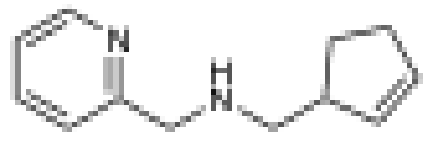	0.33	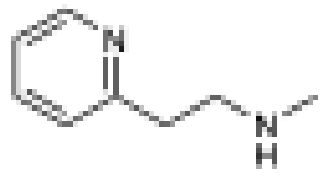
Z3241259976	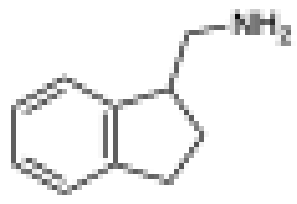	0.32	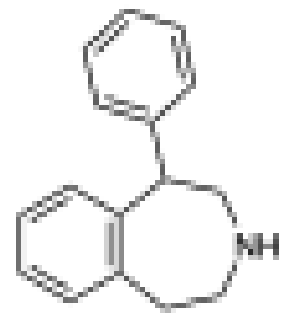
EN300-47307925	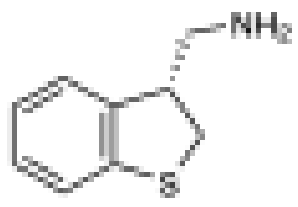	0.25	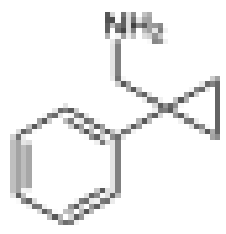
Z8987163207	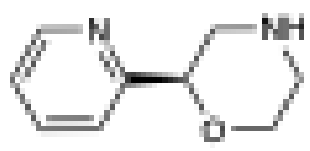	0.17	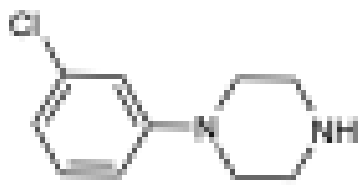
Z1255382255	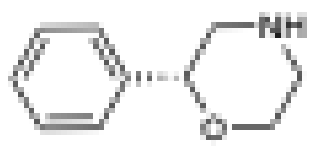	0.19	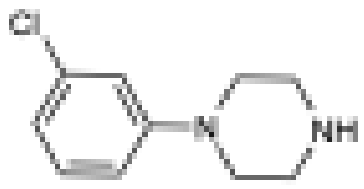
Z8987197482	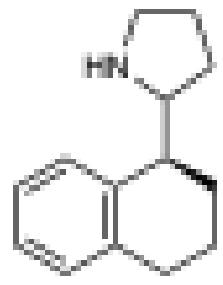	0.17	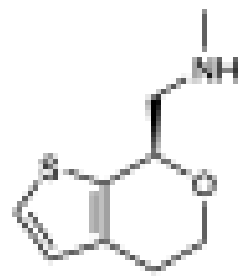
Z337709400	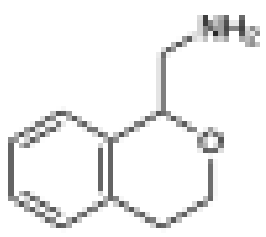	0.34	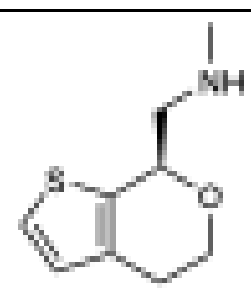
Z1255424149	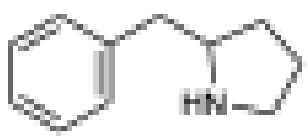	0.33	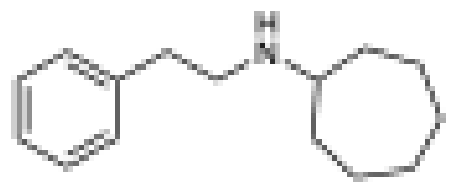
Z1891774421	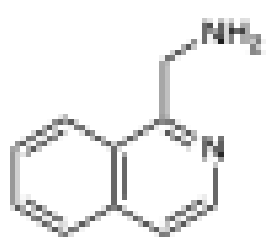	0.26	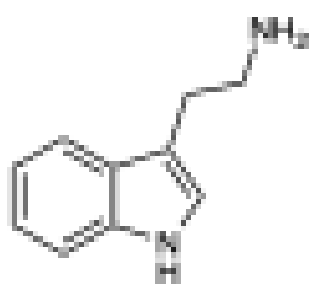

[a] Maximal Tanimoto similarity coefficient (Tc) between the compound and all ChEMBL ligands of TAAR1 (Standard Value ≤ 10000 nM).

Supplementary Table 3. Optimized agonists from initial hits.

ID	Structure	pEC$_{50}$ for TAAR1	pEC$_{50}$ for 5-HT$_{1A}$
Z3241259976	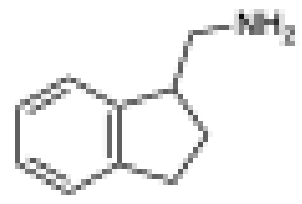	7.49 ± 0.1	5.38 ± 0.53
Z1493816383	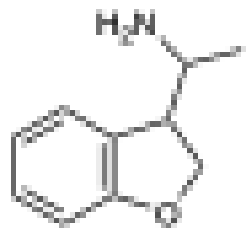	6.44 ± 0.08	--
Z2732542387	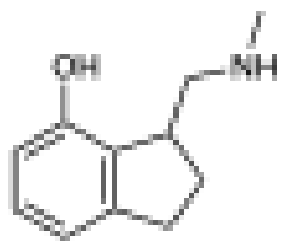	6.11 ± 0.07	--
Z1251362220	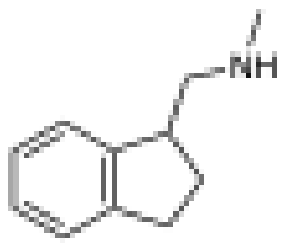	6.31 ± 0.04	--
Z1316760731	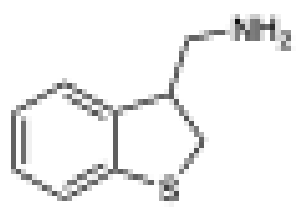	7.56 ± 0.09	5.91 ± 0.3
EN300-47307925	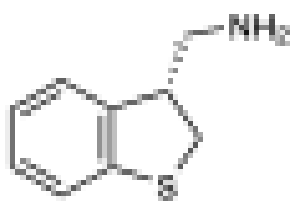	7.66 ± 0.13	6.38 ± 0.3
Z3235143301	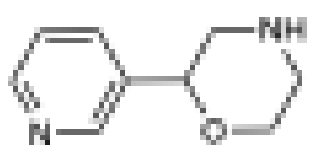	6.16 ± 0.07	--
Z954552190	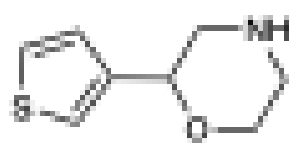	8.02 ± 0.09	--
Z3235143299	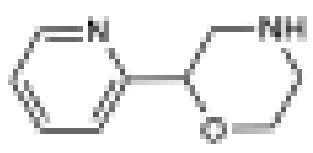	8.4 ± 0.1	5.3 ± 0.47
ZINCcj0000002gTq	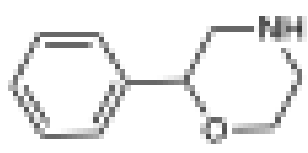	8.65 ± 0.12	6.11 ± 0.31
Z8987163207	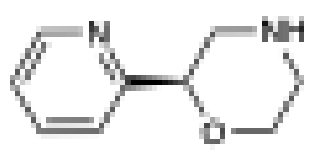	8.6 ± 0.12	5.49 ± 0.28
Z1255382255	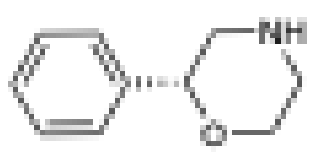	8.82 ± 0.08	6.42 ± 0.32
Z3519067312	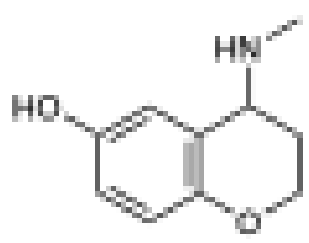	3.7 ± 0.21	--
Z1262608488	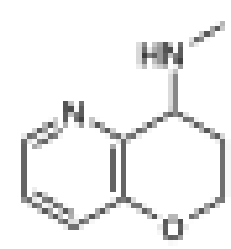	5.06 ± 0.05	--

Z85923435	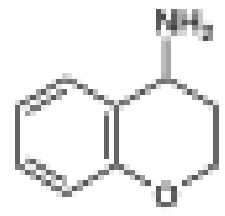	5.66 ± 0.04	--
Z1262565741	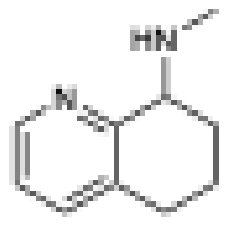	5.78 ± 0.03	--
Z337709402	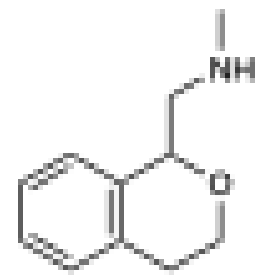	6.28 ± 0.05	--
Z337709400	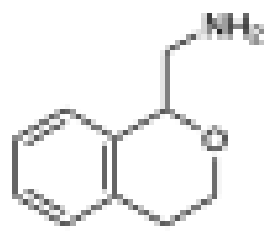	7.21 ± 0.05	--
Z995211376	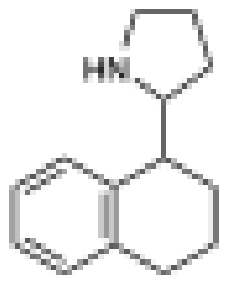	6.46 ± 0.06	5.7 ± 0.9
Z8987197482	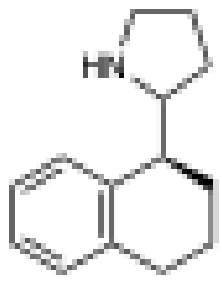	7.15 ± 0.12	6.22 ± 0.69
Z1198160843	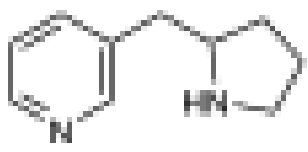	4.7 ± 0.04	--
Z285198426	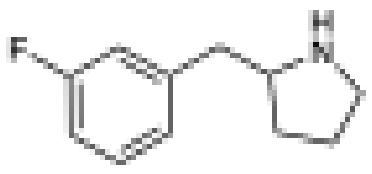	6.56 ± 0.06	--
Z1255424149	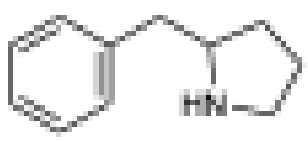	7.04 ± 0.04	--
Z1163091197	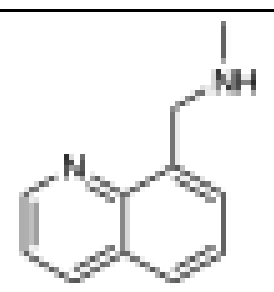	6.59 ± 0.04	--
Z105198014	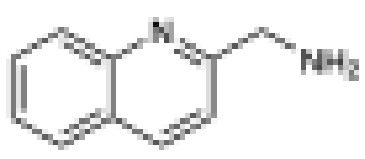	6.11 ± 0.07	--
Z3033185048	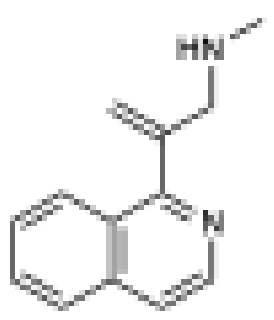	5.84 ± 0.05	5.52 ± 0.26
Z1198724030	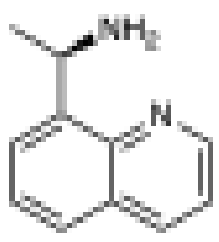	6.58 ± 0.04	--

Z234897549	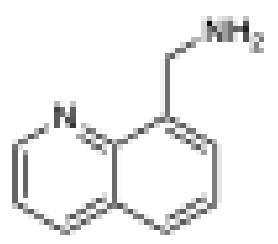	6.46 ± 0.03	--
Z1891774421	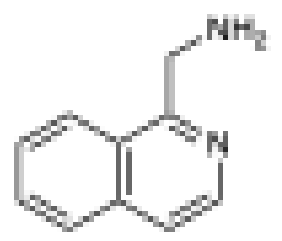	6.74 ± 0.08	--

"--" indicates that the value could not be determined due to insufficient compound activity.

Supplementary Table 4. Antagonists docking against the Boltz-2 inactive-state structure.

ID	Structure	Rank	Docking score
RTI-7470-44	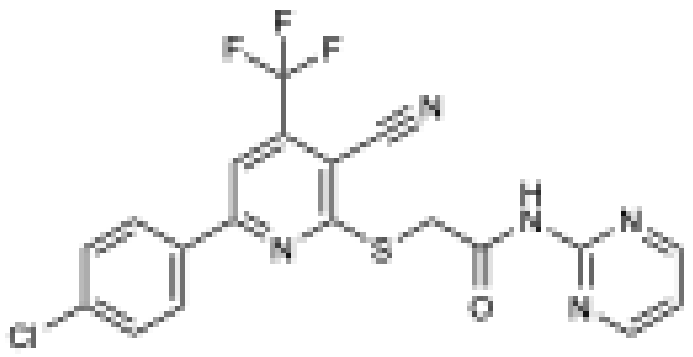	657,123	-42.21
EPPTB	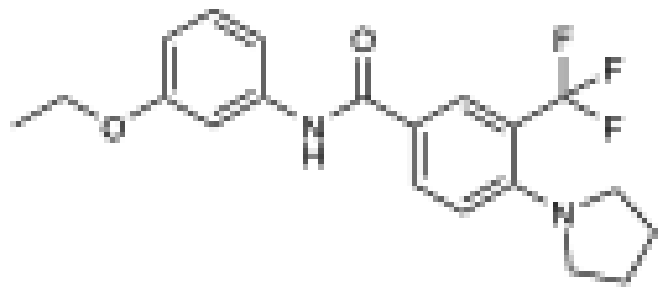	NA	NA
Compound 3	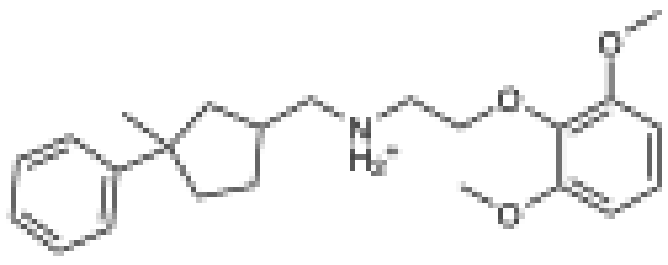	NA	NA
Compound 9	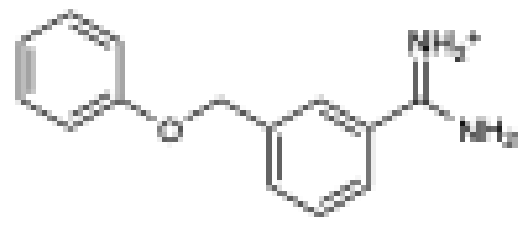	3,709,173	-34.81
Compound 16	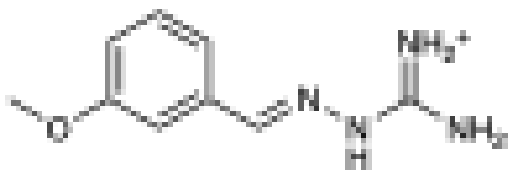	174,773	-45.95
Compound 22	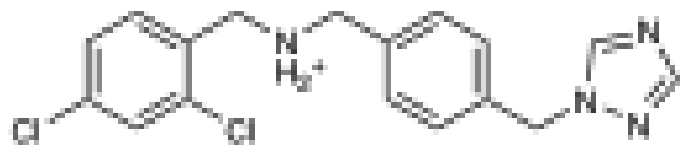	NA	NA
Compound 24	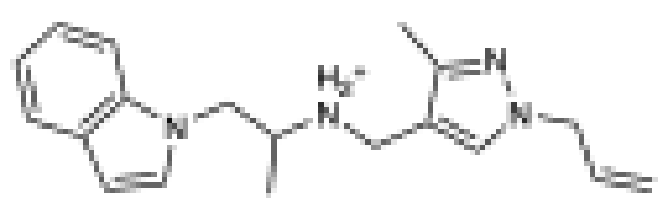	NA	NA

An NA result means the compound has a docking score higher than (worse than) -10 kal/mol and thus are not recorded in the final docking result.

Supplementary Table 5. Cryo-EM data collection, refinement and validation statistics.

**Data Collection**		
**Protein**	TAAR1-‘7482	TAAR1-‘2255
**Voltage (kV)**	300	300
**Detector**	Falcon 4	Falcon 4
**Pixel size (Å)**	0.73	0.73
**Defocus range (μm)**	1.0-2.0	1.0-2.0
**Electron dose (e^-/Å^2)**	50	50
**Dose per image**	1.38	1.38
**3D reconstruction**		
**Final Particle number**	137,014	72,449
**Symmetry**	C1	C1
**Overall resolution (Å)**	3.12	3.14
**Model refinement**		
**Model composition**		
**Chains**	6	6
**Ligands**	1	1
**Non-hydrogen atoms**	8231	8232
**Protein residues**	1040	1040
**Bonds (RMSD)**		
**Length (Å)**	0.003	0.004
**Angles (°)**	0.567	0.571
**Ramachandran plot (%)**		
**Outliers**	0.00	0.00
**Allowed**	2.24	2.63
**Favored**	97.76	97.37
**Rotamer outliers (%)**	0.11	0.00
**MolProbity score**	1.33	1.46
**Clash score**	5.21	6.07

Supplementary Table 6. Pharmacokinetic properties of docking agonists.

**Compound**	**Sample**	**Administration**	**Dose, mg/kg**	**Pharmacokinetic Parameters**					
				Tmax, min	Cmax, ng/ml	$AUC_{0\to t\ min}$ (AUClast) ng*min/ml	$AUC_{0\to\infty}$ (AUCINF_obs) ng*min/ml	$T_{1/2}$ (HL_Lambda_z), min	K_{el} (Lambda_z), min^{-1}
**‘3027**	Plasma	IP	10	30.0	6860	671000	673000	65.8	0.0105
	Brain			15.0	29800	2660000	2670000	66.9	0.0104
	CSF			15.0	3040	292000	295000	55.8	0.0124
**‘2255**	Plasma	IP	10	15.0	3780	259000	260000	42.4	0.0164
	Brain			15.0	14600	882000	884000	38.9	0.0178
	CSF			15.0	1430	67400	75800	34.7	0.020
**‘7482**	Plasma	IP	10	5.00	1810	134000	135000	65.0	0.0107
	Brain			30.0	11300	1870000	1880000	58.6	0.0118
	CSF			5.00	1750	123000	124000	64.0	0.0108

Supplementary Table 7. Statistics for the prepulse inhibition studies.

Figure[a] Notes	Statistical Model[b]	Variable[c]	Degrees of Freedom	F-, H-, statistic[d]	*p*-value[e]	# Mice per Group
Fig. 5a<br>Sex, Treat	Levene's test (Sex. Treat)	Equality of Error Variances<br>4 dB	11,50	0.657	0.771	8-14
**SEP**-363856		8 dB	11,50	0.908	0.539	
(ulotront)		12 dB	11,50	0.950	0.503	
PPI	RMANOVA	PPI	2,100	269.726	<0.001	
	(within subjects)	PPI x Sex	2,100	0.009	0.991	
		PPI x Treat	10,100	4.550	<0.001	
		PPI x Sex x Treat	10,100	1.400	0.191	
	(between subjects)	Sex	1,50	0.035	0.852	
		Treat	5,50	11.888	<0.001	
		Sex x Treat	5,50	1.295	0.281	
Treat	Levene's test (Treat)	Equality of Error Variances<br>4 dB	5,56	0.676	0.643	
PPI		8 dB	5,56	0.865	0.511	
		12 dB	5,56	0.454	0.809	
	RMANOVA	PPI	2,112	267.424	<0.001	
	(within subjects)	PPI x Treat	10,112	4.510	<0.001	
	(between subjects)	Treat	5,56	11.718	<0.001	
	Bonferroni	Veh+Veh+Veh *vs.* EPPTB+Veh+Veh			1.000	
		Veh+Veh+Veh *vs.* Veh+0.5mg/kg SEP+Veh			0.613	
		Veh+Veh+Veh *vs.* Veh+Veh+AMPH			<0.001	
		Veh+Veh+Veh *vs.* Veh+0.5mg/kg SEP+AMPH			1.000	
		Veh+Veh+Veh *vs.* EPPTB+0.5mg/kg SEP+AMPH			0.107	
		Veh+0.5mg/kg SEP+Veh *vs.*			<0.001	

		Veh+Veh+AMPH				
		Veh+0.5mg/kg SEP+Veh *vs.* EPPTB+0.5mg/kg SEP+AMPH			<0.001	
		Veh+Veh+AMPH *vs.* EPPTB+Veh+Veh			<0.001	
		Veh+Veh+AMPH *vs.* Veh+0.5mg/kg SEP+AMPH			<0.001	
		Veh+Veh+AMPH *vs.* EPPTB+0.5mg/kg SEP+AMPH			0.300	
Fig. 5b Sex, Treat	Levene's test (Sex, Treat)	Equality of Error Variances 4 dB	13,59	1.372	0.200	9-14
Z898713**3207**		8 dB	13,59	1.586	0.115	
		12 dB	13,59	1.776	0.069	
PPI	RMANOVA	PPI	2,118	270.022	<0.001	
		PPI x Sex	2,118	0.498	0.609	
		PPI x Treat	12,118	4.385	<0.001	
		PPI x Sex x Treat	12,118	2.000	0.030	
		Sex	1,59	2.091	0.153	
		Treat	6,59	12.109	<0.001	
		Sex x Treat	6,59	2.034	0.075	
	Bonferroni	Veh+Veh+Veh – 4dB ♂ *vs.* ♀			0.655	
	Treat x dB x Sex	Veh+Veh+Veh – 8dB ♂ *vs.* ♀			0.833	
		Veh+Veh+Veh – 12dB ♂ *vs.* ♀			0.030	
		EPPTB+Veh+Veh – 4dB ♂ *vs.* ♀			0.439	
		EPPTB+Veh+Veh – 8dB ♂ *vs.* ♀			0.297	
		EPPTB+Veh+Veh – 12dB ♂ *vs.* ♀			0.315	
		Veh+0.5 '3207+AMPH – 4dB ♂ *vs.* ♀			0.488	
		Veh+0.5 '3207+AMPH – 8dB ♂ *vs.* ♀			0.213	
		Veh+0.5 '3207+AMPH – 12dB ♂ *vs.* ♀			0.735	
		Veh+Veh+AMPH – 4dB ♂ *vs.* ♀			0.046	
		Veh+Veh+AMPH – 8dB ♂ *vs.* ♀			0.023	
		Veh+Veh+AMPH – 12dB ♂ *vs.* ♀			0.200	

		Veh+0.2 ‘3207+AMPH – 4dB ♂ *vs.* ♀			0.133	
		Veh+0.2 ‘3207+AMPH – 8dB ♂ *vs.* ♀			0.002	
		Veh+0.2 ‘3207+AMPH – 12dB ♂ *vs.* ♀			0.953	
		Veh+0.5 ‘3207+AMPH – 4dB ♂ *vs.* ♀			0.349	
		Veh+0.5 ‘3207+AMPH – 8dB ♂ *vs.* ♀			0.522	
		Veh+0.5 ‘3207+AMPH – 12dB ♂ *vs.* ♀			0.686	
		EPPTB+0.5 ‘3207+AMPH – 4dB ♂ *vs.* ♀			0.775	
		EPPTB+0.5 ‘3207+AMPH – 8dB ♂ *vs.* ♀			0.218	
		EPPTB+0.5 ‘3207+AMPH – 12dB ♂ *vs.* ♀			0.230	
	Sex x dB x Treat	♂ 8dB: Veh+Veh+Veh *vs.* Veh+0.2 ‘3207+AMPH			0.050	
		♂ 12dB: Veh+Veh+AMPH *vs.* Veh+Veh+Veh			0.002	
		♂ 12dB: Veh+Veh+AMPH *vs.* EPPTB+Veh+Veh			<0.001	
		♂ 12dB: Veh+Veh+AMPH *vs.* Veh+0.5 ‘3207+Veh			<0.001	
		♂ 12dB: Veh+Veh+AMPH *vs.* Veh+0.5 ‘3207+AMPH			<0.001	
		♂ 12dB: EPPTB+0.5 ‘3207+ AMPH *vs.* Veh+Veh+Veh			0.061	
		♂ 12dB: EPPTB+0.5 ‘3207+ AMPH *vs.* EPPTB+Veh+Veh			0.009	
		♂ 12dB: EPPTB+0.5 ‘3207+ AMPH *vs.* Veh+0.5 ‘3207+Veh			0.029	
		♂ 12dB: EPPTB+0.5 ‘3207+ AMPH *vs.* Veh+0.5 ‘3207+AMPH			0.007	
		♀ 4dB: Veh+Veh+AMPH *vs.* EPPTB+Veh+Veh			0.046	

		♀ 4dB: Veh+Veh+AMPH *vs.* Veh+0.2 ‘3207+AMPH			0.012	
		♀ 4dB: Veh+Veh+AMPH *vs.* Veh+0.5 ‘3207+AMPH			0.033	
		♀ 4dB: EPPTB+0.5 ‘3207+AMPH *vs.* Veh+0.2 ‘3207+AMPH			0.069	
		♀ 8dB: Veh+Veh+AMPH *vs.* Veh+Veh+Veh			<0.001	
		♀ 8dB: Veh+Veh+AMPH *vs.* EPPTB+Veh+Veh			<0.001	
		♀ 8dB: Veh+Veh+AMPH *vs.* Veh+0.2 ‘3207+AMPH			<0.001	
		♀ 8dB: Veh+Veh+AMPH *vs.* Veh+0.5 ‘3207+AMPH			0.018	
		♀ 12dB: Veh+Veh+AMPH *vs.* Veh+Veh+Veh			<0.001	
		♀ 12dB: Veh+Veh+AMPH *vs.* EPPTB+Veh+Veh			<0.001	
		♀ 12dB: Veh+Veh+AMPH *vs.* Veh+0.2 ‘3207+Veh			0.014	
		♀ 12dB: Veh+Veh+AMPH *vs.* Veh+0.5 ‘3207+Veh			<0.001	
		♀ 12dB: Veh+Veh+AMPH *vs.* EPPTB+0.5 ‘3207+AMPH			0.100	
		♀ 12dB: Veh+0.2’3207+AMPH *vs.* Veh+Veh+Veh			0.011	
		♀ 12dB: Veh+0.2 ’3207+AMPH *vs.* EPPTB+Veh+Veh			0.044	
		12dB: Veh+0.5 ’3207+AMPH *vs.* EPPTB+0.5 ‘3207+AMPH			0.032	
		♀ 12dB: EPPTB+0.5 ’3207+ AMPH *vs.* Veh+Veh+Veh			<0.001	
		♀ 12dB: EPPTB+0.5 ’3207+ AMPH *vs.* EPPTB+Veh+Veh			0.001	
	Sex x Treat x dB	Only groups that are not shown are significant 4 *vs* 8, 8 *vs.* 12, or 4 *vs.* 12 dB				
		♂ Veh+Veh+AMPH: 8 *vs.* 12dB			1.000	

		♂ Veh+0.2 ‘3207+AMPH: 4 *vs.* 8dB			0.695	
		♂ EPPTB-0.5 ‘3207-AMPH: 4 *vs.* 8dB			0.331	
		♂ EPPTB-0.5 ‘3207-AMPH: 8 *vs.* 12dB			0.426	
		♀ Veh+Veh+AMPH: 4 *vs.* 8dB			0.155	
		♀ Veh+Veh+AMPH: 8 *vs.* 12dB			0.164	
		♀ Veh+0.2 ‘3207+AMPH: 8 *vs.* 12dB			1.000	
		♀ EPPTB+0.5 ‘3207+AMPH: 8 *vs.* 12dB			0.056	
	Treat	Veh+Veh+Veh *vs.* EPPTB+Veh+Veh			1.000	
		Veh+Veh+Veh *vs.* Veh+0.5 ‘3207+Veh			1.000	
		Veh+Veh+Veh *vs.* Veh+Veh+AMPH			<0.001	
		Veh+Veh+Veh *vs.* Veh+0.2 ‘3207+AMPH			0.643	
		Veh+Veh+Veh *vs.* Veh+0.5 ‘3207+AMPH			1.000	
		Veh+Veh+Veh *vs.* EPPTB+0.5 ‘3207+AMPH			<0.001	
		EPPTB+Veh+Veh *vs.* Veh+Veh+AMPH			<0.001	
		EPPTB+Veh+Veh *vs.* EPPTB+0.5 ‘3207+AMPH			<0.001	
		Veh+Veh+AMPH *vs.* Veh+0.5 ‘3207+Veh			<0.001	
		Veh+Veh+AMPH *vs.* Veh+0.2 ‘3207+AMPH			0.005	
		Veh+Veh +AMPH *vs.* Veh+0.5 ‘3207+AMPH			<0.001	
		EPPTB+0.5 ‘3207+AMPH *vs.* Veh+0.5 ‘3207+Veh			0.020	
		EPPTB+0.5 ‘3207+AMPH *vs.* Veh+0.5 ‘3207+AMPH			0.001	

Fig. 5c<br>Sex, Treat	Levene’s test (Sex, Treat)	Equality of Error Variances<br>4 dB	13,58	2.210	0.020	9-14
Z1255382**2255**		8 dB	13,58	1.564	0.123	
		12 dB	13,58	0.656	0.796	
PPI	RMANOVA	PPI	1.877,108.878	385.356	<0.001	
	(within subjects)	PPI x Sex	1.877,108.878	0.974	0.376	
	Greenhouse-Geisser Corrections	PPI X Treat	11.263,108.878	6.288	<0.001	
		PPI X Sex X Treat	11.263,108.878	1.515	0.135	
	(between subjects)	Sex	1,58	0.037	0.849	
		Treat	6,58	18.993	<0.001	
		Sex x Treat	6,58	1.743	0.127	
Treat	Levene’s test (Treat)	Equality of Error Variances<br>4 dB	6,65	2.900	0.014	
		8 dB	6,65	1.701	0.135	
		12 dB	6,65	0.188	0.979	
PPI	RMANOVA	PPI	1.884,122.487	377.623	<0.001	
	(within subjects) Geenhouse-Geisser correction	PPI x Treat	11.306,122.487	5.947	<0.001	
	(between subjects)	Treat	6.65	17.549	<0.001	
	Bonferroni	Veh+Veh+Veh *vs.* EPPTB+Veh+Veh			1.000	
		Veh+Veh+Veh *vs.* Veh+0.2mg/kg ‘2255+Veh			1.000	
		Veh+Veh+Veh *vs.* Veh+Veh+AMPH			<0.001	
		Veh+Veh+Veh *vs.* Veh+0.1mg/kg ‘2255+AMPH			<0.001	
		Veh+Veh+Veh *vs.* Veh+0.2mg/kg ‘2255+AMPH			1.000	
		Veh+Veh+Veh *vs.*			<0.001	

		EPPTB+0.2mg/kg ‘2255+AMPH				
		EPPTB+Veh+Veh *vs.* Veh+Veh+AMPH			<0.001	
		EPPTB+Veh+Veh *vs.* Veh+0.1mg/kg ‘2255+AMPH			0.001	
		EPPTB+Veh+Veh *vs.* EPPTB+0.2mg/kg ‘2255+AMPH			<0.001	
		Veh+0.2mg/kg ‘2255+Veh *vs.* Veh+Veh+AMPH			<0.001	
		Veh+0.2mg/kg ‘2255+Veh *vs.* Veh+0.1mg/kg ‘2255+AMPH			0.040	
		Veh+0.2mg/kg ‘2255+Veh *vs.* EPPTB+0.2mg/kg ‘2255+AMPH			<0.001	
		Veh+Veh+AMPH *vs.* Veh+0.2mg/kg ‘2255+AMPH			<0.001	
		Veh+0.2mg/kg ‘2255+AMPH *vs.* Veh+0.1mg/kg ‘2255+AMPH			0.024	
		Veh+0.2mg/kg ‘2255+AMPH *vs.* EPPTB+0.2mg/kg ‘2255+AMPH			<0.001	
Fig. 5d Sex, Treat	Levene’s test (Sex, Treat)	Equality of Error Variances 4 dB	13,57	2.446	0.010	9-14
Z898719**7482**		8 dB	13,57	2.706	0.005	
		12 dB	13,57	0.893	0.565	
PPI	RMANOVA	PPI	1.819,103.668	297.984	<0.001	
	(within subjects)	PPI x Sex	1.819,103.668	0.150	0.841	
	(Greenhouse-Geisser corrections)	PPI x Treat	10.912,103.668	5.157	<0.001	
		PPI x Sex x Treat	10.912,103.668	1.516	0.137	
	(between subjects)	Sex	1,57	0.452	0.504	
		Treat	6,57	12.957	<0.001	
		Sex x Treat	6,57	1.489	0.198	
Treat	Levene’s test (Treat)	Equality of Error Variances 4 dB	6,64	2.005	0.078	
		8 dB	6,64	1.290	0.275	
		12 dB	6,64	0.193	0.978	

PPI	RMANOVA	PPI	2,128	315.372	<0.001	
	(within subjects)	PPI x Treat	12,128	4.932	<0.001	
	(between subjects)	Treat	6,64	13.258	<0.001	
	Bonferroni	Veh+Veh+Veh *vs.* EPPTB+Veh+Veh			1.000	
		Veh+Veh+Veh *vs.* Veh+0.2mg/kg ‘7482+Veh			0.242	
		Veh+Veh+Veh *vs.* Veh+Veh+AMPH			<0.001	
		Veh+Veh+Veh *vs.* Veh+0.1mg/kg ‘7482+AMPH			<0.001	
		Veh+Veh+Veh *vs.* Veh+0.2mg/kg ‘7482+AMPH			1.000	
		Veh+Veh+Veh *vs.* EPPTB+0.2mg/kg ‘7482+AMPH			<0.001	
		EPPTB+Veh+Veh *vs.* Veh+Veh+AMPH			<0.001	
		EPPTB+Veh+Veh *vs.* Veh+0.1mg/kg ‘7482+AMPH			<0.001	
		EPPTB+Veh+Veh *vs.* EPPTB+0.2mg/kg ‘7482+AMPH			<0.001	
		Veh+0.2mg/kg ‘7482+Veh *vs.* Veh+Veh+AMPH			0.081	
		Veh+0.2mg/kg ‘7482+Veh *vs.* EPPTB+0.2mg/kg ‘7482+AMPH			0.054	
		Veh+0.1mg/kg ‘7482+AMPH *vs.* EPPTB+0.2mg/kg ‘7482+AMPH			1.000	
		Veh+0.2mg/kg ‘7482+AMPH *vs.* Veh+Veh+AMPH			0.001	
		Veh+0.2mg/kg ‘7482+AMPH *vs.* Veh+0.1mg/kg ‘7482+AMPH			0.060	
		Veh+0.2mg/kg ‘7482+AMPH *vs.* EPPTB+0.2mg/kg ‘7482+AMPH			<0.001	
Suppl. Fig. 3a Sex, Treat	Levene’s Test (Sex, Treat)	Equality of Error Variances	11,50	2.165	0.032	8-14

**SEP**-363856	Two-Way ANOVA	Sex	1,50	0.510	0.479	
(ulotront)		Trest	5,50	10.573	<0.001	
Null Activity		Sex x Treat	5,50	1.631	0.169	
Treat	Levene's Test (Treat)	Equality of Error Variances	5,56	2.223	0.065	
	One-Way ANOVA	Treat	5,56	11.126	<0.001	
	Bonferroni	Veh+Veh+Veh *vs.* EPPTB+Veh+Veh			1.000	
		Veh+Veh+Veh *vs.* Veh+5mg/kg SEP+Veh			1.000	
		Veh+Veh+Veh *vs.* Veh+Veh+AMPH			0.006	
		Veh+Veh+Veh *vs.* Veh+5mg/kg SEP+AMPH			1.000	
		Veh+Veh+Veh *vs.* EPPTB+5mg/kg SEP+AMPH			<0.001	
		EPPTB+Veh+Veh *vs.* Veh+Veh+AMPH			0.020	
		EPPTB+Veh+Veh *vs.* EPPTB+5 mg/kg SEP+AMPH			<0.001	
		Veh+5mg/kg SEP+Veh *vs.* Veh+Veh+AMPH			0.041	
		Veh+5mg/kg SEP+Veh *vs.* EPPTB+5 mg/kg SEP+AMPH			<0.001	
		Veh+5mg/kg SEP+AMPH *vs.* EPPTB+5mg/kg SEP+AMPH			<0.001	
Suppl. Fig. 3b Sex, Treat	Levene's Test (Sex, Treat)	Equality of Error Variances	13,59	1.348	0.213	9-14
Z898713**3207**	Two-Way ANOVA	Sex	1,59	6.570	0.013	
Null Activity		Treat	6,59	10.141	<0.001	
		Sex x Treat	6,59	2.269	0.049	
	Bonferroni	Veh-Veh-Veh: ♂ *vs.* ♀			0.783	
		EPPTB-Veh-Veh: ♂ *vs.* ♀			0.897	
		Veh+0.5mg/kg '3207+Veh: ♂ *vs.* ♀			0.435	

		Veh+Veh+AMPH: ♂ *vs.* ♀			0.780	
		Veh+0.2mg/kg '3207+AMPH: ♂ *vs.* ♀			0.002	
		Veh+0.5mg/kg '3207+AMPH: ♂ *vs.* ♀			0.003	
		EPPTB+0.5mg.kg '3207+AMPH: ♂ *vs.* ♀			0.845	
		♂ Veh+Veh+Veh *vs.* ♂ EPPTB+Veh+Veh			1.000	
		♂ Veh+Veh+Veh *vs.* ♂ Veh+0.5mg/kg '3207+Veh			1.000	
		♂ Veh+Veh+Veh *vs.* ♂ Veh+Veh+AMPH			0.221	
		♂ Veh+Veh+Veh *vs.* ♂ Veh+0.2mg/kg '3207+AMPH			<0.001	
		♂ Veh+Veh+Veh *vs.* ♂ Veh+0.5mg/kg '3207+AMPH			<0.001	
		♂ Veh+Veh+Veh *vs.* ♂ EPPTB+0.5mg/kg '3207+ AMPH			0.251	
		♂ Veh+0.2mg/kg '3207+AMPH *vs.* ♂ EPPTB+Veh+Veh			<0.001	
		♂ Veh+0.2mg/kg '3207+AMPH *vs.* ♂ Veh+0.5mg/kg '3207+Veh			<0.001	
		♂ Veh+0.5mg/kg '3207+AMPH *vs.* ♂ EPPTB+Veh+Veh			<0.001	
		♂ Veh+0.5mg/kg '3207+AMPH *vs.* ♂ Veh+0.5mg/kg '3207+Veh			<0.001	
		♀ Veh+Veh+Veh *vs.* ♀ EPPTB+Veh+Veh			1.000	
		♀ Veh+Veh+Veh *vs.* ♀ Veh+0.5mg/kg '3207+Veh			1.000	
		♀ Veh+Veh+Veh *vs.* ♀ Veh+Veh+AMPH			0.047	
		♀ Veh+Veh+Veh *vs.* ♀ Veh+0.2mg/kg '3207+AMPH			1.000	
		♀ Veh+Veh+Veh *vs.* ♀ Veh+0.5mg/kg '3207+AMPH			1.000	

		♀ Veh+Veh+Veh *vs.* ♀ EPPTB+0.5mg/kg '3207+ AMPH			<0.001	
		♀ Veh-0.5 '3207+Veh *vs.* ♀ EPPTB+Veh+Veh			0.026	
Suppl. Fig. 3c Sex, Treat	Levene's Test (Sex, Treat)	Equality of Error Variances	13,58	2.158	0.023	9-14
Z125538**2255**	Two-Way ANOVA	Sex	1,58	3.273	0.076	
Null Activity		Treat	6,58	14.314	<0.001	
		Sex x Treat	6,58	3.084	0.011	
Sex	Mann-Whitney	Sex		598.000	0.577	
Treat	Levene's Test (Treat)	Equality of Error Variances	6,65	3.606	0.004	
	One-Way ANOVA	Treat	6,65	11.804	<0.001	
	One-Way Kruskal-Wallis	Treat	6	47.452	<0.001	
	Bonferroni	Veh+Veh+Veh *vs.* EPPTB+Veh+Veh			0.844	
		Veh+Veh+Veh *vs.* Veh+0.2mg/kg '2255+Veh			0.101	
		Veh+Veh+Veh *vs.* Veh+Veh+AMPH			<0.001	
		Veh+Veh+Veh *vs.* Veh+0.1mg/kg '2255+AMPH			0.067	
		Veh+Veh+Veh *vs.* Veh+0.2mg/kg '2255+AMPH			<0.001	
		Veh+Veh+Veh *vs.* EPPTB+0.2mg/kg '2255+AMPH			<0.001	
		EPPTB+Veh+Veh *vs.* Veh+Veh+AMPH			0.004	
		EPPTB+Veh+Veh *vs.* Veh+0.2mg/kg '2255+AMPH			<0.001	
		EPPTB+Veh+Veh *vs.*			0.002	

		EPPTB+0.2mg/kg '2255+AMPH				
		Veh+0.2mg/kg '2255+Veh *vs.* Veh+Veh+AMPH			<0.001	
		Veh+0.2mg/kg '2255+Veh *vs.* Veh+0.1mg/kg '2255+AMPH			0.001	
		Veh+0.2mg/kg '2255+Veh *vs.* Veh+0.2mg/kg '2255+AMPH			<0.001	
		Veh+0.2mg/kg '2255+Veh *vs.* EPPTB+0.2mg/kg '2255+AMPH			<0.001	
		Veh+0.2mg/kg '2255+AMPH *vs.* Veh+0.1mg/kg '2255+AMPH			0.084	
Suppl. Fig. 3d Sex, Treat	Levene's Test	Equality of Error Variances	13,57	2.213	0.020	9-14
Z898719**7482**	Two-Way ANOVA	Sex	1,57	0.021	0.885	
Null Activity		Treat	6,57	13.304	<0.001	
		Sex x Treat	6,57	0.574	0.749	
Sex	Mann-Whitney	Sex		728.000	0.254	
Treat	Levene's Test	Equality of Error Variances	6,64	3.776	0.003	
	One-Way ANOVA	Treat	6,64	16.246	<001	
	One-Way Kruskal-Wallis	Treat	6	45.922	<0.001	
	Bonferroni	Veh+Veh+Veh *vs.* EPPTB+Veh+Veh			0.946	
		Veh+Veh+Veh *vs.* Veh+0.2mg/kg '7482+Veh			0.966	
		Veh+Veh+Veh *vs.* Veh+Veh+AMPH			<0.001	
		Veh+Veh+Veh *vs.* Veh+0.1mg/kg '7482+AMPH			0.011	
		Veh+Veh+Veh *vs.* Veh+0.2mg/kg '7482+AMPH			<0.001	
		Veh+Veh+Veh *vs.* EPPTB+0.2mg/kg '7482+AMPH			<0.001	

		EPPTB+Veh+Veh *vs.* Veh+Veh+AMPH			0.002	
		EPPTB+Veh+Veh *vs.* Veh+0.1mg/kg ‘7482+AMPH			0.021	
		EPPTB+Veh+Veh *vs.* EPPTB+0.2mg/kg ‘7482+AMPH			<0.001	
		Veh+0.2mg/kg ‘7482+Veh *vs.* Veh+Veh+AMPH			0.002	
		Veh+0.2mg/kg ‘7482+Veh *vs.* Veh+0.1mg/kg ‘7482+AMPH			0.019	
		Veh+0.2mg/kg ‘7482+Veh *vs.* Veh+0.2mg/kg ‘7482+AMPH			<0.001	
		Veh+0.2mg/kg ‘7482+Veh *vs.* EPPTB+0.2mg/kg ‘7482+AMPH			<0.001	
Suppl. Fig. 4a Sex, Treat	Levene’s Test (Sex, Treat)	Equality of Error Variances	11,50	0.981	0.476	8-14
**SEP**-363856	Two-Way ANOVA	Sex	1,50	0.007	0.931	
(ulotront)		Trest	5,50	3.901	0.005	
Startle Activity		Sex x Treat	5,50	1.216	0.316	
Treat	Levene’s Test (Treat)	Equality of Error Variances	5,56	1.331	0.265	
	One-Way ANOVA	Treat	5,56	3.834	0.005	
	Bonferroni	Veh+Veh+Veh *vs.* EPPTB+Veh+Veh			1.000	
		Veh+Veh+Veh *vs.* Veh+5mg/kg SEP+Veh			1.000	
		Veh+Veh+Veh *vs.* Veh+Veh+AMPH			1.000	
		Veh+Veh+Veh *vs.* Veh+5mg/kg SEP+AMPH			1.000	
		Veh+Veh+Veh *vs.* EPPTB+5mg/kg SEP+AMPH			0.811	
		Veh+Veh+AMPH *vs.* Veh+5mg/kg SEP+AMPH			0.035	
		Veh+5mg/kg SEP+AMPH *vs.*			0.014	

		EPPTB+5mg/kg SEP+AMPH				
Suppl. Fig. 4b Sex, Treat	Levene's Test (Sex, Treat)	Equality of Error Variances	13,59	0.993	0.470	9-14
Z89871**3207**	Two-Way ANOVA	Sex	1,59	6.074	0.017	
Startle Activity		Trest	6,59	4.782	<0.001	
		Sex x Treat	6,59	3.363	0.006	
	Bonferroni	Veh-Veh-Veh: ♂ *vs.* ♀			0.663	
		0.5 EPPTB-Veh-Veh: ♂ *vs.* ♀			0.053	
		Veh+0.5mg/kg '3207+Veh: ♂ *vs.* ♀			0.803	
		Veh+Veh+AMPH: ♂ *vs.* ♀			0.591	
		Veh+0.2mg/kg '3207+AMPH: ♂ *vs.* ♀			<0.001	
		Veh+0.5mg/kg '3207+AMPH: ♂ *vs.* ♀			0.009	
		EPPTB+0.5mg.kg '3207+AMPH: ♂ *vs.* ♀			0.371	
		♂ Veh+Veh+Veh *vs.* ♂ EPPTB+Veh+Veh			1.000	
		♂ Veh+Veh+Veh *vs.* ♂ Veh+0.5mg/kg '3207+Veh			1.000	
		♂ Veh+Veh+Veh *vs.* ♂ Veh+Veh+AMPH			1.000	
		♂ Veh+Veh+Veh *vs.* ♂ Veh+0.2mg/kg '3207+AMPH			1.000	
		♂ Veh+Veh+Veh *vs.* ♂ Veh+0.5mg/kg '3207+AMPH			1.000	
		♂ Veh+Veh+Veh *vs.* ♂ EPPTB+0.5mg/kg '3207+ AMPH			1.000	
		♀ Veh+Veh+Veh *vs.* ♀ EPPTB+Veh+Veh			1.000	
		♀ Veh+Veh+Veh *vs.* ♀ Veh+0.5mg/kg '3207+Veh			1.000	
		♀ Veh+Veh+Veh *vs.* ♀ Veh+Veh+AMPH			1.000	
		♀ Veh+Veh+Veh *vs.*			0.005	

		♀ Veh+0.2mg/kg ‘3207+AMPH				
		♀ Veh+Veh+Veh *vs.* ♀ Veh+0.5mg/kg ‘3207+AMPH			0.003	
		♀ Veh+Veh+Veh *vs.* ♀ EPPTB+0.5mg/kg ‘3207+ AMPH			1.000	
		♀ Veh-0.2 ‘3207+AMPH *vs.* ♀ Veh+Veh+AMPH			<0.001	
		♀ Veh-0.2 ‘3207+AMPH *vs.* ♀ Veh+0.5 ‘3207+Veh			0.096	
		♀ Veh-0.2 ‘3207+AMPH *vs.* ♀ EPPTB+0.5mg/kg ‘3207+ AMPH			0.039	
		♀ Veh-0.5 ‘3207+AMPH *vs.* ♀ Veh+Veh+AMPH			<0.001	
		♀ Veh-0.5 ‘3207+AMPH *vs.* ♀ Veh+0.5 ‘3207+Veh			0.057	
		♀ Veh-0.5 ‘3207+AMPH *vs.* ♀ EPPTB+0.5mg/kg ‘3207+ AMPH			0.020	
Suppl. Fig. 4c Sex, Treat	Levene’s Test (Sex, Treat)	Equality of Error Variances	13,58	2.820	0.003	9-14
Z125538**2255**	Two-Way ANOVA	Sex	1,58	5.453	0.023	
Startle Activity		Treat	6,58	12.775	<0.001	
		Sex x Treat	6,58	1.177	0.331	
Sex	Mann-Whitney	Sex		550.000	0.272	
Treat	Levene’s Test (Treat)	Equality of Error Variances	6,65	2.050	0.071	
	One-Way ANOVA	Treat	6,65	11.882	<0.001	
	Bonferroni	Veh+Veh+Veh *vs.* EPPTB+Veh+Veh			1.000	
		Veh+Veh+Veh *vs.* Veh+0.2mg/kg ‘2255+Veh			<0.001	
		Veh+Veh+Veh *vs.*			1.000	

		Veh+Veh+AMPH				
		Veh+Veh+Veh *vs.* Veh+0.1mg/kg '2255+AMPH			0.264	
		Veh+Veh+Veh *vs.* Veh+0.2mg.kg '2255+AMPH			0.034	
		Veh+Veh+Veh *vs.* EPPTB+0.2mg/kg '2255+AMPH			1.000	
		Veh+0.2mg/kg '2255+Veh *vs.* EPPTB+Veh+Veh			<0.001	
		Veh+0.2mg/kg '2255+Veh *vs.* Veh+Veh+AMPH			0.011	
		Veh+0.2mg/kg '2255+Veh *vs.* Veh+0.2mg/kg '2255+AMPH			<0.001	
		Veh+0.2mg/kg '2255+Veh *vs.* EPPTB+0.2mg/kg '2255+AMPH			<0.001	
		Veh+Veh+AMPH *vs.* Veh+0.2mg/kg '2255+AMPH			0.003	
		Veh+0.1mg/kg '2255+AMPH *vs.* Veh+0.2mg/kg '2255+AMPH			<0.001	
Suppl. Fig. 4d (Sex, Treat)	Levene's Test	Equality of Error Variances	13.57	1.709	0.084	9-14
Z898719**7482**	Two-Way ANOVA	Sex	1,57	8.484	0.005	
Startle Activity		Treat	6,57	7.881	<0.001	
		Sex x Treat	6,57	2.125	0.064	
	Bonferroni	Veh+Veh+Veh *vs.* EPPTB+Veh+Veh			1.000	
		Veh+Veh+Veh *vs.* Veh+0.2mg/kg '7482+Veh			<0.001	
		Veh+Veh+Veh *vs.* Veh+Veh+AMPH			1.000	
		Veh+Veh+Veh *vs.* Veh+0.1mg/kg '7482+AMPH			1.000	
		Veh+Veh+Veh *vs.* Veh+0.2mg/kg '7482+AMPH			0.417	
		Veh+Veh+Veh *vs.* EPPTB+0.2mg/kg '7482+AMPH			1,000	
		EPPTB+Veh+Veh *vs.*			<0.001	

		Veh+0.2mg/kg ‘7482+Veh				
		Veh+0.2mg/kg ‘7482+Veh *vs.* EPPTB+Veh+Veh			<0.001	
		Veh+0.2mg/kg ‘7482+Veh *vs.* Veh+Veh+AMPH			0.048	
		Veh+0.2mg/kg ‘7482+Veh *vs.* Veh+0.1mg/kg ‘7482+AMPH			0.007	
		Veh+0.2mg/kg ‘7482+Veh *vs.* Veh+0.2mg/kg ‘7482+AMPH			<0.001	
		Veh+Veh+AMPH *vs.* Veh+0.2mg.kg ‘7482+AMPH			0.034	
		Veh+0.2mg/kg ‘7482+AMPH *vs.* EPPTB+0.2mg/kg ‘7482+AMPH			0.040	

[a]Abbreviations: PPI, prepulse inhibition; SEP, SEP-363856 (ulotaront) is a trace amine 1 receptor agonist with serotonin 1A receptor agonist activity.

[b]Abbreviations: ANOVA, analysis of variance; RMANOVA, repeated measures analyses of variance.

[c]Abbreviations: dB, decibel; PPI, prepulse inhibition; Treat, treatment; Veh, Vehicle; SEP, SEP-363856 (ulotaront); EPPTB, trace amine 1 receptor/antagonist/inverse agonist; ‘3207, Z898713207; ‘2255, Z1255382255; ‘7482, Z8987197482; novel trace amine 1 receptor agonists; AMPH amphetamine.

[d]F, statistic for ANOVA, RMANOVA, and Levene’s tests; H, statistic for Kruskal-Wallis test.

[e]Abbreviation: *p*-value, probability value from given statistical test.

Supplementary Table 8. Numbers of mice in the prepulse inhibition studies.

Treatment[a]	SEP-363856			Z898713207			Z1255382255			Z898717482		
	♂[b]	♀[b]	Total[b]	♂[b]	♀[b]	Total[b]	♂[b]	♀[b]	Total[b]	♂[b]	♀[b]	Total[b]
Veh-Veh-Veh	7	7	14	7	7	14	7	7	14	7	7	14
EPPTB-Veh-Veh	6	4	10	6	4	10	6	4	10	6	4	10
Veh-Hi Dose Comp-Veh	4	4	8	7	3	10	5	5	10	5	4	9
Veh-Veh-AMPH	5	5	10	5	5	10	5	5	10	5	5	10
Veh-Lo Dose Comp-Veh	---	---	---	5	5	10	3	6	9	4	5	9
Veh-Hi Dose Comp-Veh	5	5	10	5	5	10	5	5	10	5	5	10
EPPTB-Hi Dose Comp-AMPH	4	6	10	2	7	9	4	5	9	2	7	9

[a]Abbreviations: Veh, Vehicle; EPPTB, N-(3-ethoxy-phenyl)-4-pyrrolidin-1-yl-3-trifluoromethyl-benzamide (SML1350), a TAAR1 antagonist; Hi, high; Comp, compound; AMPH, amphetamine; and Lo, low.

[b]♂, male; ♀, female; Total, total numbers of mice in a given treatment that were tested.

Supplementary Methods

Synthetic procedures

Method 1. An aryl halide (100 mg), boronic derivative (1 mol equiv to the aryl halide), sodium carbonate (1.5 mol equiv to the aryl halide), 1,1′- [bis(diphenylphosphino)ferrocene]-dichloropalladium(II) (0.05 mol equiv to the aryl halide), and a water-dioxane mixture 1:3 (1 mL) were placed in a vial. The vial was then placed into a thermostat and stirred for 24 h at 95 °C. After cooling down the mixture, the solvents were evaporated under reduced pressure. The residue was dissolved in chloroform (3 mL) and washed with water (3×1 mL). The catalyst was then filtered, and solvents were evaporated under reduced pressure. The sample was dissolved in 0.6 mL of TFA, placed in an ultrasonic bath for 1 h, and then kept overnight at room temperature. TFA was further removed under reduced pressure, and the crude sample was purified by preparative HPLC.

Method 2. An amine (100 mg), DIPEA (1.2 mol equiv to the amine), and DMSO (0.5 mL) were placed into a 4 mL capped glass vial and stirred for 30 min. After the addition of an alkyl halide (1.2 mol equiv to the amine), the vial was stirred for 12 h at room temperature. Then the vial was placed into a thermostat (set to 100°C) for 9 h. After cooling down, the mixture was filtered; the solvent and volatile components were evaporated under reduced pressure. After the addition of 0.6 mL of TFA, the sample was placed in an ultrasonic bath for 1 h, and then kept overnight at room temperature. TFA was further removed under reduced pressure, and the crude sample was dissolved in 0.5 mL of DMSO and purified by preparative HPLC.

Method 3. An amine (100 mg) was dissolved in 0.5 mL of DMF and shaken for 20 min at room temperature. Then, an aldehyde (1.1 mol equiv to the amine) was added. The resulting mixture was heated for 5 h at 100°C. After cooling down to room temperature, methanol (1 mL) was added, followed by NaBH4 (2 mol equiv to the amine). The reaction vial was left shaken overnight. Then, CHCl3 (3 mL) was added, and the mixture was washed with water (2 mL). The organic phase was separated, washed with Na2SO4, and evaporated. The crude sample was purified utilizing preparative HPLC.

Method 4. An amine (100 mg), DIPEA (1.2 mol equivalent to the amine), and DMSO (0.5 mL) were placed into a 4 mL capped glass vial and stirred for 30 min. After the addition of an alkyl halide (1.2 mol equiv to the amine), the vial was stirred for 12 h at room temperature. Then the vial was placed into a thermostat (set to 100°C) for 9 h. After cooling down, the mixture was filtered; the solvent and volatile components were evaporated under reduced pressure to give the crude product. The product was further purified by HPLC.

Spectral Description

1-(2,3-dihydro-1-benzofuran-3-yl)methanamine – ZINCbh0000002DSB, Z1197877490. EN300-112172 is a catalog building block, available from Enamine's stock.

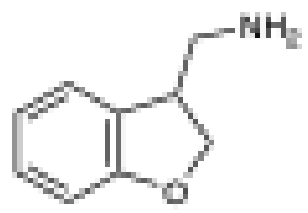

Purity, >95% (assessed by LC/MS).

^{1}H NMR (400 MHz, $CDCl_3$) δ 7.23 (d, J = 7.3 Hz, 1H), 7.16 (t, J = 7.8 Hz, 1H), 6.88 (t, J = 7.4 Hz, 1H), 6.82 (d, J = 8.0 Hz, 1H), 4.66 (t, J = 9.0 Hz, 1H), 4.43 (dd, J = 9.0, 5.2 Hz, 1H), 3.51 (p, J = 6.1 Hz, 1H), 2.97 (qd, J = 12.5, 5.9 Hz, 2H), 1.24 (s, 2H).

LC/MS (APSI) m/z [M] calculated for C9H11NO: 149.0; found: 149.1.

1-{4H,6H,7H-thieno[3,2-c]pyran-4-yl}ethan-1-amine hydrochloride – ZINCcn0000002ry9, Z2211231413. EN300-221284 is a catalog building block, available from Enamine's stock.

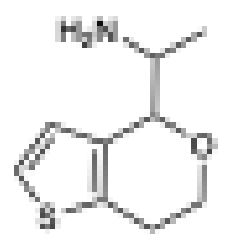

Purity, >95% (assessed by LC/MS).

^{1}H NMR (400 MHz, DMSO-d_6) δ 8.11 (d, J = 183.0 Hz, 3H), 7.40 (d, J = 5.2 Hz, 1H), 6.99 (d, J = 5.3 Hz, 1H), 4.85 (d, J = 103.7 Hz, 1H), 4.36 – 4.04 (m, 1H), 3.90 – 3.59 (m, 2H), 2.91 (d, J = 17.3 Hz, 1H), 2.77 (t, J = 15.2 Hz, 1H), 1.13 (dd, J = 179.8, 6.6 Hz, 3H).

LC/MS (APSI) m/z [M+H] calculated for C9H14NOS: 184.1; found: 184.1.

1-{5H,6H,7H-cyclopenta[b]pyridin-7-yl}methanamine dihydrochloride – ZINCbg0000002DEe, Z2489361097. EN300-270332 is a catalog building block, available from Enamine's stock.

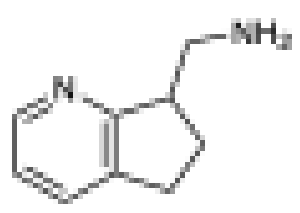

Purity, >95% (assessed by LC/MS).

^{1}H NMR (400 MHz, DMSO-d_6) δ 8.59 (d, J = 5.5 Hz, 1H), 8.48 (s, 3H), 8.24 (d, J = 7.7 Hz, 1H), 7.70 (dd, J = 7.7, 5.6 Hz, 1H), 3.85 (p, J = 7.4 Hz, 1H), 3.56 (dt, J = 11.9, 5.7 Hz, 1H), 3.07 (dddd, J = 44.2, 24.8, 15.6, 7.3 Hz, 3H), 2.42 (dtd, J = 13.6, 8.6, 5.2 Hz, 1H), 2.20 – 2.07 (m, 1H).

LC/MS (APSI) m/z [M+H] calculated for C9H13N2: 149.4; found: 149.1.

2-(naphthalen-1-yl)prop-2-en-1-amine; trifluoroacetic acid – ZINCey000000C7Tw,

Z8711777920. This compound was obtained using Method 1 from EN300-247414 and EN300-19782.

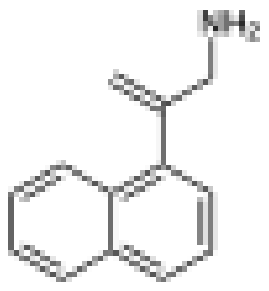

Yield: 12%; Purity, >95% (assessed by LC/MS).

^{1}H NMR (500 MHz, DMSO-d_6) δ 8.34 (s, 3H), 8.03 - 7.94 (m, 2H), 7.92 (d, J = 8.2 Hz, 1H), 7.57 - 7.53 (m, 2H), 7.52 (d, J = 7.9 Hz, 1H), 7.41 (d, J = 6.7 Hz, 1H), 5.73 (s, 1H), 5.34 (s, 1H), 3.86 (d, J = 2.7 Hz, 2H).

LC/MS (APSI) m/z [M+H] calculated for C13H14N: 184.2; found: 184.1.

2-[(pyrrolidin-2-yl)methyl]pyridine dihydrochloride – ZINCcj0000002gSF, Z3214214254.

EN300-6501865 is a catalog building block, available from Enamine's stock.

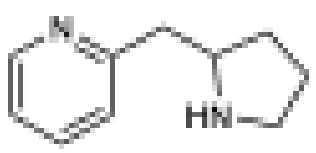

Purity, >95% (assessed by LC/MS).

^{1}H NMR (400 MHz, DMSO-d_6) δ 9.68 (s, 2H), 8.81 (d, J = 5.6 Hz, 1H), 8.40 (t, J = 7.9 Hz, 1H), 8.01 (d, J = 7.9 Hz, 1H), 7.83 (t, J = 6.7 Hz, 1H), 3.94 (t, J = 7.5 Hz, 1H), 3.56 (dd, J = 14.6, 8.2 Hz, 1H), 3.47 (dd, J = 14.6, 6.2 Hz, 1H), 3.23 (s, 1H), 2.05 (ddd, J = 25.3, 12.6, 4.7 Hz, 2H), 1.85 (dt, J = 20.7, 7.8 Hz, 1H), 1.76 – 1.62 (m, 1H).

LC/MS (APSI) m/z [M+H] calculated for C10H15N2: 163.2; found: 163.1.

6-fluoro-3,4-dihydro-2H-1-benzopyran-4-amine hydrochloride – ZINCcm0000002EAW, Z1266854953. EN300-73698 is a catalog building block, available from Enamine's stock.

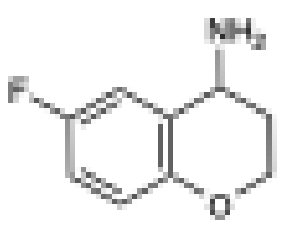

Purity, >95% (assessed by LC/MS).

^{1}H NMR (400 MHz, DMSO-d_6) δ 8.76 (s, 3H), 7.47 (dd, J = 9.4, 3.2 Hz, 1H), 7.13 (td, J = 8.6, 3.1 Hz, 1H), 6.88 (dd, J = 9.1, 4.8 Hz, 1H), 4.50 (t, J = 5.7 Hz, 1H), 4.23 (dddd, J = 17.7, 11.3, 8.8, 3.3 Hz, 2H), 2.24 (tt, J = 9.2, 5.0 Hz, 1H), 2.11 (ddt, J = 11.7, 6.1, 3.0 Hz, 1H).

LC/MS (APSI) m/z [M+H] calculated for C9H11FNO: 168.0; found: 168.1.

1-{pyrazolo[1,5-a]pyridin-7-yl}methanamine dihydrochloride – ZINCbd0000002Cxp, Z4244625472. EN300-8156861 is a catalog building block, available from Enamine's stock.

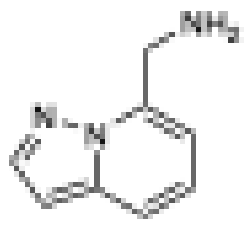

Purity, >95% (assessed by LC/MS).

^{1}H NMR (400 MHz, DMSO-d_6) δ 8.83 (s, 3H), 8.12 (d, *J* = 2.2 Hz, 1H), 7.79 (d, *J* = 8.9 Hz, 1H), 7.30 (dd, *J* = 8.8, 6.9 Hz, 1H), 7.12 (d, *J* = 6.9 Hz, 1H), 6.76 (d, *J* = 2.3 Hz, 1H), 4.51 (d, *J* = 5.6 Hz, 2H).

LC/MS (APSI) m/z [M+H] calculated for C8H10N: 148.2; found: 148.1.

4-[(pyrrolidin-2-yl)methyl]-3,4-dihydro-2H-1,4-benzoxazine dihydrochloride – ZINCgm000000M8UF, Z8700802749. This compound was obtained using Method 2 from EN300-35485 and EN300-86375.

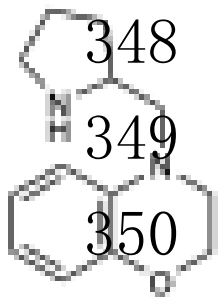

Yield: 48%; Purity, >95% (assessed by LC/MS).

^{1}H NMR (500 MHz, DMSO-d_6) δ 9.35 (s, 1H), 9.14 (s, 1H), 6.81 (dd, *J* = 8.1, 1.5 Hz, 1H), 6.75 (td, *J* = 7.6, 1.6 Hz, 1H), 6.68 (dd, *J* = 7.9, 1.5 Hz, 1H), 6.60 – 6.53 (m, 1H), 4.18 (dtdd, *J* = 10.6, 8.3, 5.5, 3.0 Hz, 2H), 3.71 (t, *J* = 7.5 Hz, 1H), 3.58 (dd, *J* = 14.9, 8.4 Hz, 1H), 3.49 – 3.40 (m, 2H), 3.23 (ddt, *J* = 11.9, 7.6, 4.0 Hz, 1H), 3.10 (ddt, *J* = 15.3, 11.9, 6.2 Hz, 1H), 2.11 (dtd, *J* = 11.8, 7.4, 3.9 Hz, 1H), 1.97 (dq, *J* = 12.8, 4.3 Hz, 1H), 1.89 (tt, *J* = 16.1, 6.1 Hz, 1H), 1.60 (dq, *J* = 12.9, 8.9 Hz, 1H).

LC/MS (APSI) m/z [M+H] calculated for C13H19N2O: 219.2; found: 219.1.

[(1-benzofuran-3-yl)methyl](methyl)amine – ZINCcr0000000JH8, Z1197877493. This compound was obtained using Method 3 from EN300-30027 and EN300-108926.

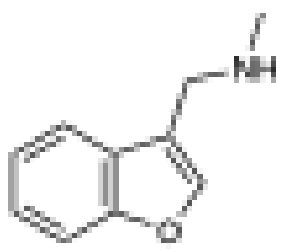

Yield: 21%; Purity, >95% (assessed by LC/MS).

^{1}H NMR (400 MHz, DMSO-d_6) δ 7.82 (s, 1H), 7.71 (dd, *J* = 7.8, 1.4 Hz, 1H), 7.54 (dd, *J* = 8.1, 1.0 Hz, 1H), 7.27 (dtd, *J* = 24.8, 7.2, 1.2 Hz, 2H), 3.76 (s, 2H), 3.30 (s, 1H), 2.31 (s, 3H).

LC/MS (APSI) m/z [M+H] calculated for C10H12NO: 162.0; found: 162.1.

4-{[(3,4-dihydro-2H-1-benzopyran-4-yl)methyl]amino}but-2-en-1-ol – ZINChm000000Gu6n, Z2590548459. This compound was obtained using Method 3 from EN300-157158 and EN300-102599.

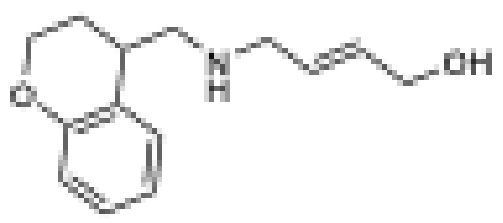

Yield: 12%; Purity, >95% (assessed by LC/MS).
^{1}H NMR (600 MHz, DMSO-d6) δ 7.18 (d, J = 7.5 Hz, 1H), 7.05 (t, J = 7.7 Hz, 1H), 6.81 (t, J = 7.3 Hz, 1H), 6.71 (d, J = 8.1 Hz, 1H), 5.60 – 5.51 (m, 1H), 5.51 – 5.40 (m, 1H), 4.16 – 4.09 (m, 1H), 4.09 – 4.03 (m, 1H), 4.00 (d, J = 6.1 Hz, 2H), 3.30 – 3.27 (m, 1H), 3.24 – 3.14 (m, 2H), 2.87 – 2.81 (m, 1H), 2.78 (dd, J = 12.0, 4.8 Hz, 1H), 2.61 (dd, J = 11.5, 9.8 Hz, 1H), 2.02 – 1.94 (m, 1H), 1.94 – 1.85 (m, 1H).
LC/MS (APSI) m/z [M+H] calculated for C14H20NO2: 234.4; found: 234.1.

2-(aminomethyl)-3,4-dihydro-2H-1-benzopyran-4-one hydrochloride – ZINCdf000001oqrA, Z2739607300. EN300-349414 is a catalog building block, available from Enamine's stock.

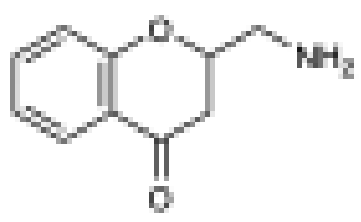

Purity, >95% (assessed by LC/MS).
^{1}H NMR (400 MHz, DMSO-d_6) δ 8.47 (s, 3H), 7.78 (d, *J* = 7.8 Hz, 1H), 7.62 (t, *J* = 7.8 Hz, 1H), 7.16 – 7.05 (m, 2H), 4.87 – 4.78 (m, 1H), 3.35 (s, 1H), 2.95 (ddd, *J* = 16.7, 13.1, 3.2 Hz, 1H), 2.77 (dt, *J* = 17.0, 3.3 Hz, 1H).
LC/MS (APSI) m/z [M+H] calculated for C10H12NO2: 178.2; found: 178.1.

(2R)-1-(7-fluoro-2,3-dihydro-1H-indol-1-yl)propan-2-amine dihydrochloride – ZINCel000000ZhUM, Z8721927756. This compound was obtained using Method 2 from EN300-98048 and EN300-1588161.

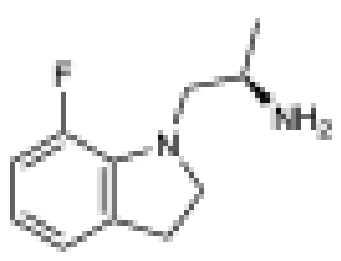

Yield: 53%; Purity, >95% (assessed by LC/MS).
^{1}H NMR (500 MHz, DMSO-d_6) δ 8.03 – 7.99 (m, 3H), 6.93 – 6.83 (m, 2H), 6.63 (td, *J* = 7.7, 4.2 Hz, 1H), 3.45 (q, *J* = 8.7 Hz, 1H), 3.40 – 3.32 (m, 2H), 3.24 (dd, *J* = 13.0, 5.0 Hz, 1H), 2.97 (td, *J* = 8.6, 2.9 Hz, 2H), 1.22 (d, *J* = 6.1 Hz, 3H).
LC/MS (APSI) m/z [M+H] calculated for C11H16FN2: 195.2; found: 195.1.

[(cyclopent-2-en-1-yl)methyl][(pyridin-2-yl)methyl]amine – ZINCer0000002Srj, Z2787083864. This compound was obtained using Method 3 from EN300-373377 and EN300-18999.

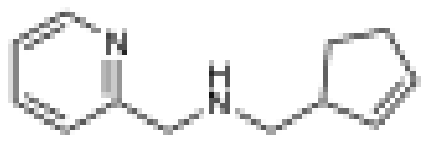

Yield: 32%; Purity, >95% (assessed by LC/MS).

^{1}H NMR (500 MHz, DMSO-d_6) δ 9.53 (s, 1H), 8.67 (d, J = 4.6 Hz, 1H), 7.99 (t, J = 7.6 Hz, 1H), 7.7 (d, J = 7.9 Hz, 1H), 7.51 (t, J = 6.3 Hz, 1H), 5.87 - 5.75 (m, 2H), 4.39 - 4.29 (m, 2H), 3.15 - 3.03 (m, 1H), 2.99 - 2.9 (m, 1H), 2.9 - 2.81 (m, 1H), 2.39 - 2.18 (m, 2H), 2.09 - 1.95 (m, 1H), 1.63 - 1.53 (m, 1H).

LC/MS (APSI) m/z [M+H] calculated for C12H17N2: 189.1; found: 189.1.

1-(2,3-dihydro-1H-inden-1-yl)methanamine hydrochloride – ZINCbm0000002DE2, Z3241259976. EN300-7831773 is a catalog building block, available from Enamine's stock.

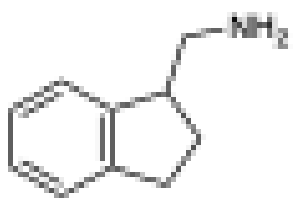

Purity, >95% (assessed by LC/MS).

^{1}H NMR (400 MHz, $CDCl_3$) δ 7.21 (ddt, *J* = 22.8, 5.6, 3.4 Hz, 4H), 3.22 (p, *J* = 6.7 Hz, 1H), 3.06 – 2.81 (m, 5H), 2.29 (dtd, *J* = 12.6, 8.3, 5.7 Hz, 1H), 1.85 (ddt, *J* = 12.7, 8.7, 6.4 Hz, 1H), 1.15 (s, 2H).

LC/MS (APSI) m/z [M+H] calculated for C10H14N: 148.0; found: 148.1.

1-(2,3-dihydro-1-benzofuran-3-yl)ethan-1-amine – 7490_27, Z1493816383. EN300-116047 is a catalog building block, available from Enamine's stock.

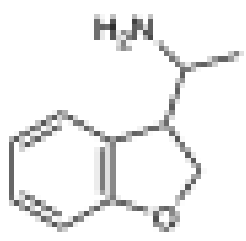

Purity, >95% (assessed by LC/MS).

^{1}H NMR (500 MHz, DMSO-d_6) δ 7.28 – 7.22 (m, 1H), 7.07 (t, *J* = 7.6 Hz, 1H), 6.79 (td, *J* = 7.4, 3.7 Hz, 1H), 6.70 (d, *J* = 7.9 Hz, 1H), 4.53 – 4.36 (m, 2H), 3.63 – 2.86 (m, 4H), 0.87 (dd, *J* = 72.6, 6.4 Hz, 3H).

LC/MS (APSI) m/z [M+H] calculated for C10H14NO: 164.1; found: 164.1.

1-(2,3-dihydro-1-benzothiophen-3-yl)methanamine – 7490_13, Z1316760731. EN300-226087 is a catalog building block, available from Enamine's stock.

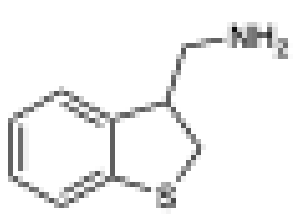

Purity, >95% (assessed by LC/MS).

^{1}H NMR (500 MHz, DMSO-d_6) δ 7.19 (dd, *J* = 10.2, 7.6 Hz, 2H), 7.10 (t, *J* = 7.5 Hz, 1H), 7.05 – 6.96 (m, 1H), 3.45 (dd, *J* = 10.6, 7.5 Hz, 1H), 3.41 – 3.27 (m, 2H), 2.78 (dd, *J* = 12.6, 4.8 Hz, 1H), 2.67 (dd, *J* = 12.6, 8.5 Hz, 1H), 1.72 (s, 2H).

LC/MS (APSI) m/z [M+H] calculated for C9H11NS: 166.0; found: 166.1.

2-(pyridin-3-yl)morpholine dihydrochloride – ZINCcd0000002hfn, Z3235143301. EN300-1605748 is a catalog building block, available from Enamine's stock.

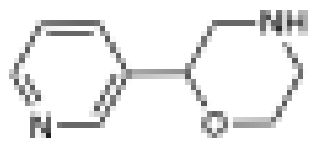

Purity, >95% (assessed by LC/MS).

^{1}H NMR (400 MHz, DMSO-d_6) δ 10.11 (s, 2H), 8.87 (dd, *J* = 12.4, 3.8 Hz, 2H), 8.46 (d, *J* = 8.1 Hz, 1H), 7.97 (dd, *J* = 8.1, 5.5 Hz, 1H), 5.10 (dd, *J* = 11.4, 2.6 Hz, 1H), 4.16 (dd, *J* = 12.6, 3.8 Hz, 1H), 4.04 (td, *J* = 12.3, 2.5 Hz, 1H), 3.58 (d, *J* = 12.6 Hz, 1H), 3.25 (d, *J* = 12.8 Hz, 1H), 3.09 (d, *J* = 11.3 Hz, 2H).

LC/MS (APSI) m/z [M+H] calculated for C9H13N2O: 165.2; found: 165.1.

2-(thiophen-3-yl)morpholine – ZINCbk0000002gVO, Z954552190. EN300-106628 is a catalog building block, available from Enamine's stock.

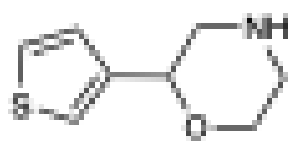

Purity, >95% (assessed by LC/MS).

^{1}H NMR (400 MHz, $CDCl_3$) δ 7.37 – 7.22 (m, 2H), 7.08 (d, *J* = 5.0 Hz, 1H), 4.60 (dd, *J* = 10.2, 2.5 Hz, 1H), 4.01 (dd, *J* = 11.6, 3.3 Hz, 1H), 3.79 (td, *J* = 11.6, 2.8 Hz, 1H), 3.18 – 3.10 (m, 1H), 3.00 (td, *J* = 11.9, 3.4 Hz, 1H), 2.98 – 2.83 (m, 2H), 2.25 (s, 1H).

LC/MS (APSI) m/z [M+H] calculated for C8H12NOS: 170.2; found: 170.1.

2-(pyridin-2-yl)morpholine dihydrochloride – ZINCcd0000002gTu, Z3235143299. EN300-19242522 is a catalog building block, available from Enamine's stock.

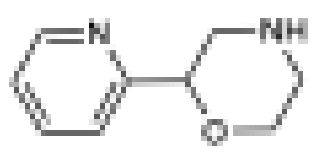

Purity, >95% (assessed by LC/MS).

^{1}H NMR (400 MHz, DMSO-d_6) δ 9.99 (s, 1H), 9.76 (s, 1H), 8.63 (d, *J* = 5.1 Hz, 1H), 8.05 (t, *J* = 7.8 Hz, 1H), 7.65 (d, *J* = 7.9 Hz, 1H), 7.54 (dd, *J* = 7.6, 5.0 Hz, 1H), 5.03 (dd, *J* = 11.1, 2.6 Hz, 1H), 4.15 (dd, *J* = 12.5, 3.8 Hz, 1H), 4.03 (td, *J* = 12.3, 2.5 Hz, 1H), 3.60 (d, *J* = 12.6 Hz, 1H), 3.26 (d, *J* = 12.8 Hz, 1H), 3.19 – 2.99 (m, 2H).

LC/MS (APSI) m/z [M+H] calculated for C9H13N2O: 165.0; found: 165.1.

(4S)-4-(methylamino)-3,4-dihydro-2H-1-benzopyran-6-ol – ZINCdk000002Tr9f,

Z3519067312. This compound was obtained using Method 4 from EN300-2008844 and EN300-1238092.

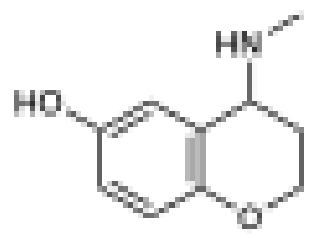

Yield: 12%; Purity, >95% (assessed by LC/MS).

^{1}H NMR (500 MHz, DMSO-d_6) δ 8.71 (s, 1H), 6.67 (d, *J* = 2.1 Hz, 1H), 6.50 (d, *J* = 1.9 Hz, 2H), 4.11 (ddd, *J* = 10.8, 8.2, 4.1 Hz, 1H), 4.03 – 3.96 (m, 1H), 3.47 (t, *J* = 4.8 Hz, 1H), 2.30 (s, 3H), 1.93 (s, 1H), 1.89 – 1.76 (m, 2H).

LC/MS (APSI) m/z [M+H] calculated for C10H14NO2: 180.0; found: 180.1.

N-methyl-2H,3H,4H-pyrano[3,2-b]pyridin-4-amine – 4953_6, Z1262608488. This compound was obtained using Method 4 from EN300-107975 and methyl amine.

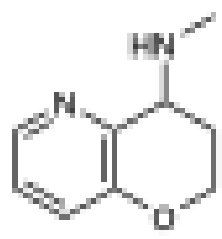

Yield: 56%; Purity, >95% (assessed by LC/MS).

^{1}H NMR (600 MHz, DMSO-d6) δ 8.10 (s, 1H), 7.18 (s, 2H), 4.30 – 4.21 (m, 1H), 4.20 – 4.15 (m, 1H), 3.61 (t, J = 4.9 Hz, 1H), 2.40 (s, 3H), 2.11 – 2.01 (m, 1H), 1.98 – 1.85 (m, 1H).

LC/MS (APSI) m/z [M+H] calculated for C9H13N2O: 165.0; found: 165.1.

3,4-dihydro-2H-1-benzopyran-4-amine – 4953_4, Z85923435. EN300-12531 is a catalog building block, available from Enamine's stock.

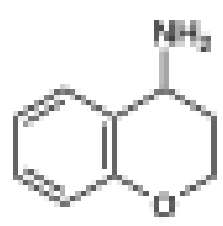

Purity, >95% (assessed by LC/MS).

^{1}H NMR (400 MHz, $CDCl_3$) δ 7.37 – 7.10 (m, 2H), 6.92 (td, *J* = 7.4, 1.2 Hz, 1H), 6.83 (dd, *J* = 8.2, 1.2 Hz, 1H), 4.36 – 4.19 (m, 2H), 4.07 (t, *J* = 5.3 Hz, 1H), 2.24 – 2.12 (m, 1H), 1.87 (dtd, *J* = 14.2, 5.8, 2.9 Hz, 1H), 1.71 (d, *J* = 3.7 Hz, 2H).

LC/MS (APSI) m/z [M] calculated for C9H11NO: 149.0; found: 149.1.

N-methyl-5,6,7,8-tetrahydroquinolin-8-amine – ZINCcm0000002wLB, Z1262565741. EN300-123794 is a catalog building block, available from Enamine's stock.

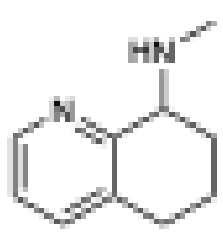

Yield: 84%; Purity, >95% (assessed by LC/MS).
^{1}H NMR (400 MHz, $CDCl_3$) δ 8.37 (d, *J* = 4.8 Hz, 1H), 7.35 (d, *J* = 7.7 Hz, 1H), 7.04 (dd, *J* = 7.7, 4.7 Hz, 1H), 3.64 (t, *J* = 6.2 Hz, 1H), 2.86 – 2.67 (m, 2H), 2.59 (s, 1H), 2.52 (s, 3H), 2.12 (d, *J* = 15.8 Hz, 1H), 1.97 (dt, *J* = 12.7, 5.6 Hz, 1H), 1.82 – 1.67 (m, 2H).
LC/MS (APSI) m/z [M+H] calculated for C10H15N2: 163.2; found: 163.1.

3-[(pyrrolidin-2-yl)methyl]pyridine – ZINCcj0000002hf5, Z1198160843. EN300-1238584 is a catalog building block, available from Enamine's stock.

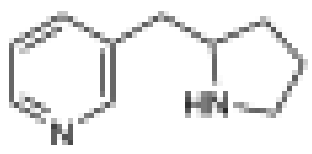

Purity, >95% (assessed by LC/MS).
^{1}H NMR (400 MHz, $CDCl_3$) δ 8.46 – 8.39 (m, 2H), 7.51 (dt, *J* = 7.8, 2.1 Hz, 1H), 7.17 (dd, *J* = 7.8, 4.8 Hz, 1H), 3.19 (p, *J* = 7.0 Hz, 1H), 2.99 (ddd, *J* = 10.3, 7.6, 5.1 Hz, 1H), 2.80 (ddd, *J* = 10.4, 8.2, 6.6 Hz, 1H), 2.70 (d, *J* = 6.9 Hz, 2H), 1.88 – 1.61 (m, 4H), 1.41 – 1.28 (m, 1H).
LC/MS (APSI) m/z [M+H] calculated for C10H15N2: 163.4; found: 163.1.

2-[(3-fluorophenyl)methyl]pyrrolidine hydrochloride – 4732_2, Z449369104. EN300-43804 is a catalog building block, available from Enamine's stock.

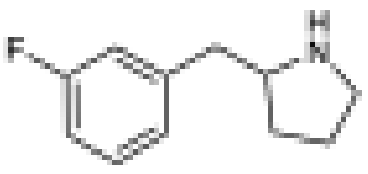

Purity, >95% (assessed by LC/MS).
^{1}H NMR (400 MHz, DMSO-d_6) δ 9.27 (s, 2H), 7.38 (td, *J* = 7.9, 6.2 Hz, 1H), 7.26 – 7.14 (m, 2H), 7.09 (td, *J* = 8.7, 2.7 Hz, 1H), 3.66 (p, *J* = 7.0 Hz, 1H), 3.34 (s, 1H), 3.21 (s, 1H), 3.12 (dd, *J* = 13.9, 7.4 Hz, 1H), 2.96 (dd, *J* = 13.8, 7.3 Hz, 1H), 2.04 – 1.76 (m, 2H), 1.60 (dq, *J* = 12.5, 8.2 Hz, 1H).
LC/MS (APSI) m/z [M+H] calculated for C11H15FN: 180.2; found: 180.1.

(2S)-2-benzylpyrrolidine hydrochloride – ZINCcp0000002gSD, Z3244742843. EN300-7420991 is a catalog building block, available from Enamine's stock.

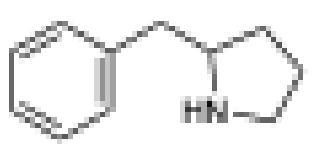

Purity, >95% (assessed by LC/MS).
^{1}H NMR (400 MHz, DMSO-d_6) δ 9.34 (d, *J* = 30.0 Hz, 2H), 7.32 (s, 3H), 7.38 – 7.21 (m, 2H), 3.63 (s, 1H), 3.35 (s, 1H), 3.11 (dt, *J* = 18.7, 9.3 Hz, 2H), 2.91 (dd, *J* = 13.6, 7.9 Hz, 1H), 1.95 (s, 1H), 1.96 – 1.81 (m, 1H), 1.84 (s, 1H), 1.60 (dq, *J* = 12.0, 8.9 Hz, 1H).
LC/MS (APSI) m/z [M+H] calculated for C11H16N: 162.2; found: 162.1.

N-methyl-1-(quinolin-8-yl)methanamine hydrochloride – ZINCdp000000Y2uG,

Z4710975379 (Method 3). EN300-27193650 is a catalog building block, available from Enamine's stock.

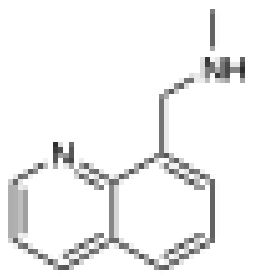

Yield: 62%; Purity, >95% (assessed by LC/MS).

^{1}H NMR (400 MHz, DMSO-d_6) δ 9.48 (s, 2H), 9.06 (dd, J = 4.5, 1.7 Hz, 1H), 8.63 (dq, J = 6.6, 2.1 Hz, 1H), 8.15 (dd, J = 8.3, 1.4 Hz, 1H), 8.07 (d, J = 7.1 Hz, 1H), 7.79 – 7.69 (m, 2H), 4.74 (t, J = 5.9 Hz, 2H), 2.61 (td, J = 5.6, 2.0 Hz, 3H).

LC/MS (APSI) m/z [M+H] calculated for C11H13N2: 173.2; found: 173.1.

1-(quinolin-2-yl)methanamine dihydrochloride – ZINCcm0000002CuJ, Z2106596235. EN300-1166953 is a catalog building block, available from Enamine's stock.

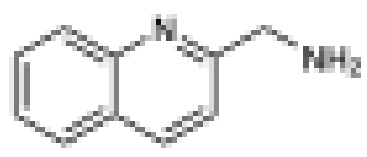

Yield: 53%; Purity, >95% (assessed by LC/MS).

^{1}H NMR (400 MHz, DMSO-d_6) δ 8.80 (s, 3H), 8.56 (d, J = 8.5 Hz, 1H), 8.10 (dd, J = 14.7, 8.3 Hz, 2H), 7.88 (ddd, J = 8.5, 6.8, 1.4 Hz, 1H), 7.76 (d, J = 8.5 Hz, 1H), 7.70 (t, J = 7.5 Hz, 1H), 4.44 (q, J = 5.8 Hz, 2H).

LC/MS (APSI) m/z [M+H] calculated for C10H11N2: 159.3; found: 159.1.

[2-(isoquinolin-1-yl)prop-2-en-1-yl](methyl)amine triflate – 7920_1, Z8843458836. Z8843458836 is a catalog screening compound, available from Enamine's stock.

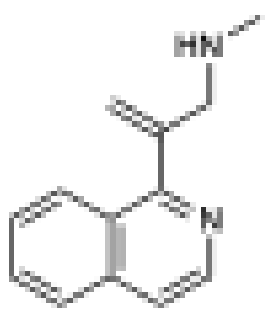

Yield: 13%; Purity, >95% (assessed by LC/MS).

^{1}H NMR (600 MHz, DMSO-d6) δ 8.80 (s, 1H), 8.52 (d, J = 5.7 Hz, 1H), 8.33 (d, J = 8.4 Hz, 1H), 8.06 (d, J = 8.1 Hz, 1H), 7.88 (d, J = 5.5 Hz, 1H), 7.85 (t, J = 7.4 Hz, 1H), 7.73 (t, J = 7.4 Hz, 1H), 6.15 (s, 1H), 5.82 (s, 1H), 4.16 (s, 2H), 2.65 (s, 3H).

LC/MS (APSI) m/z [M+H] calculated for C13H15N2: 199.0; found: 199.1.

1-(quinolin-8-yl)ethan-1-amine – ZINCds000000IYAl, Z1198724030. EN300-1272606 is a catalog building block, available from Enamine's stock.

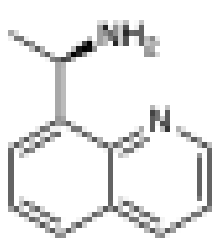

Purity, >95% (assessed by LC/MS).

^{1}H NMR (400 MHz, $CDCl_3$) δ 8.92 (dd, J = 4.2, 1.9 Hz, 1H), 8.15 (dd, J = 8.2, 1.9 Hz, 1H), 7.78 – 7.67 (m, 2H), 7.52 (t, J = 7.6 Hz, 1H), 7.40 (dd, J = 8.3, 4.2 Hz, 1H), 5.18 (q, J = 6.7 Hz, 1H), 1.97 (s, 3H), 1.61 (d, J = 6.6 Hz, 3H).

LC/MS (APSI) m/z [M+H] calculated for C11H13N2: 173.2; found: 173.1.

1-(quinolin-8-yl)methanamine – ZINCcm0000002Cxl, Z234897549. EN300-59246 is a catalog building block, available from Enamine's stock.

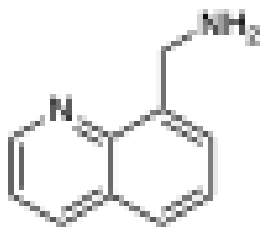

Purity, >95% (assessed by LC/MS).

^{1}H NMR (400 MHz, $CDCl_3$) δ 8.91 (dd, J = 4.2, 1.8 Hz, 1H), 8.14 (dd, J = 8.3, 1.8 Hz, 1H), 7.70 (dd, J = 8.2, 1.5 Hz, 1H), 7.62 (d, J = 6.9 Hz, 1H), 7.47 (dd, J = 8.2, 7.0 Hz, 1H), 7.39 (dd, J = 8.3, 4.2 Hz, 1H), 4.40 (s, 2H), 1.92 (d, J = 8.4 Hz, 2H).

LC/MS (APSI) m/z [M+H] calculated for C10H11N2: 159.2; found: 159.1.

1-(isoquinolin-1-yl)methanamine dihydrochloride – ZINCcm0000002D5q, Z220439492. EN300-26037 is a catalog building block, available from Enamine's stock.

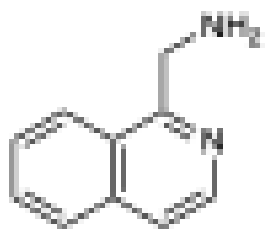

Purity, >95% (assessed by LC/MS).

^{1}H NMR (400 MHz, DMSO-d_6) δ 8.72 (s, 3H), 8.54 (d, J = 5.8 Hz, 1H), 8.29 (d, J = 8.4 Hz, 1H), 8.09 (d, J = 8.2 Hz, 1H), 7.95 (d, J = 5.8 Hz, 1H), 7.90 (t, J = 7.5 Hz, 1H), 7.79 (t, J = 7.6 Hz, 1H), 4.80 (q, J = 5.8 Hz, 2H).

LC/MS (APSI) m/z [M+H] calculated for C10H11N2: 159.2; found: 159.1.

2-(pyridin-2-yl)morpholine dihydrochloride – Z8987163207, Z8987163207. This compound was obtained by chiral separation of the racemic building block EN300-19242522 available from stock.

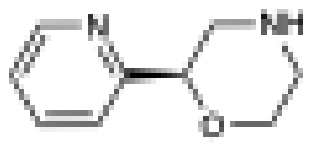

Purity, ≥98% (assessed by LC/MS).

^{1}H NMR (400 MHz, DMSO-d_6) δ 10.45 – 9.51 (m, 2H), 8.63 (d, J = 5.2 Hz, 1H), 8.09 – 8.01 (m, 1H), 7.65 (d, J = 8.0 Hz, 1H), 7.54 (dd, J = 7.6, 5.1 Hz, 1H), 5.03 (dd, J = 11.1, 2.5 Hz, 1H), 4.15 (dd, J = 12.5, 3.8 Hz, 1H), 4.03 (td, J = 12.3, 2.6 Hz, 1H), 3.60 (d, J = 12.6 Hz, 1H), 3.25 (s, 1H), 3.11 (dd, J = 16.1, 6.9 Hz, 2H).

LC/MS (APSI) m/z [M+H] calculated for C9H13N2O: 165.0; found: 165.1.

3-[(methylamino)methyl]-2,3-dihydro-1H-inden-4-ol hydrochloride – 7490_22, Z2732542387 (free base), Z8894458314 (hydrochloride).

Z8894458314 is a catalog screening compound, available from Enamine's stock.

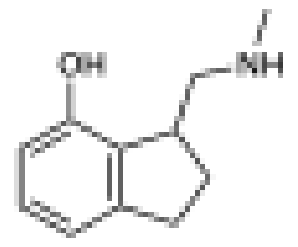

Yield: 16%; Purity, >95% (assessed by LC/MS).

^{1}H NMR (500 MHz, DMSO-d_6) δ 9.70 (s, 1H), 8.79 (s, 2H), 6.99 (t, *J* = 7.7 Hz, 1H), 6.65 (dd, *J* = 13.7, 7.7 Hz, 2H), 3.52 (tt, *J* = 8.9, 4.4 Hz, 1H), 3.27 – 3.21 (m, 1H), 2.90 (dt, *J* = 15.9, 7.9 Hz, 1H), 2.84 (d, *J* = 11.3 Hz, 1H), 2.75 (ddd, *J* = 16.0, 8.8, 4.7 Hz, 1H), 2.56 (s, 3H), 2.12 (dq, *J* = 13.1, 8.1 Hz, 1H), 2.02 (ddt, *J* = 13.0, 8.7, 4.5 Hz, 1H).

LC/MS (APSI) m/z [M+H] calculated for C11H16NO: 178.2; found: 178.1.

[(3,4-dihydro-1H-2-benzopyran-1-yl)methyl](methyl)amine hydrochloride – ZINCdl000001of0A, Z337709402 (free base), Z1269123570 (hydrochloride).

EN300-63883 is a catalog building block, available from Enamine's stock.

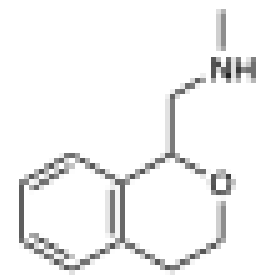

Yield: 23%; Purity, >95% (assessed by LC/MS).

^{1}H NMR (400 MHz, DMSO-d_6) δ 9.32 (s, 1H), 8.77 (s, 1H), 7.21 (q, *J* = 5.5 Hz, 4H), 5.14 – 5.07 (m, 1H), 4.07 (dt, *J* = 10.7, 5.0 Hz, 1H), 3.78 (ddd, *J* = 11.8, 7.8, 4.3 Hz, 1H), 3.48 (d, *J* = 13.0 Hz, 1H), 3.19 (t, *J* = 11.6 Hz, 1H), 2.92 – 2.80 (m, 1H), 2.75 (dt, *J* = 16.5, 4.8 Hz, 1H), 2.58 (s, 3H).

LC/MS (APSI) m/z [M+H] calculated for C11H16NO: 178.1; found: 178.1.

1-(3,4-dihydro-1H-2-benzopyran-1-yl)methanamine hydrochloride – ZINCci0000002DUp, Z337709400 (free amine), Z1695797895 (hydrochloride).

EN300-76182 is a catalog building block, available from Enamine's stock.

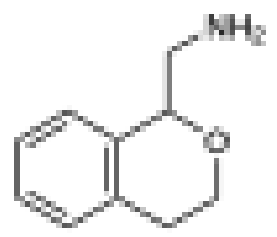

Purity, >95% (assessed by LC/MS).

^{1}H NMR (400 MHz, DMSO-d_6) δ 8.19 (s, 3H), 7.28 – 7.15 (m, 4H), 4.98 (dd, *J* = 10.0, 2.8 Hz, 1H), 4.08 (dt, *J* = 11.3, 5.0 Hz, 1H), 3.77 (ddd, *J* = 11.8, 8.1, 4.3 Hz, 1H), 3.42 – 3.30 (m, 1H), 3.05 (dd, *J* = 13.2, 9.8 Hz, 1H), 2.87 (ddd, *J* = 16.6, 8.1, 5.1 Hz, 1H), 2.74 (dt, *J* = 16.5,

4.7 Hz, 1H).
LC/MS (APSI) m/z [M+H] calculated for C10H14NO: 164.0; found: 164.1.

2-(1,2,3,4-tetrahydronaphthalen-1-yl)pyrrolidine hydrochloride – ZINCfy000000Jliy, Z995211376 (free base), Z8157041786 (hydrochloride).
EN300-99804 is a catalog building block, available from Enamine's stock.

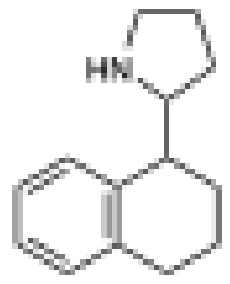

Purity, >95% (assessed by LC/MS).
^{1}H NMR (500 MHz, DMSO-d_6) δ 9.48 (s, 1H), 8.98 (s, 1H), 7.39 – 6.88 (m, 4H), 3.58 (s, 1H), 3.20 – 3.08 (m, 3H), 2.85 – 2.76 (m, 1H), 2.75 – 2.65 (m, 1H), 2.06 (d, *J* = 1.7 Hz, 2H), 1.98 – 1.89 (m, 2H), 1.88 – 1.71 (m, 2H), 1.72 (s, 2H).
LC/MS (APSI) m/z [M+H] calculated for C14H20N: 202.2; found: 202.2.

rel-(R)-(2,3-dihydrobenzo[b]thiophen-3-yl)methanamine hydrochloride – Z8987183750, EN300-47307925. This compound was obtained by chiral separation of the racemic building block EN300-51666382, available from stock.

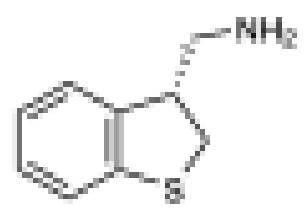

Purity, >95% (assessed by LC/MS).
^{1}H NMR (500 MHz, DMSO-d_6) δ 8.28 (s, 3H), 7.30 (d, *J* = 7.5 Hz, 1H), 7.25 (d, *J* = 7.7 Hz, 1H), 7.18 (td, *J* = 7.5, 1.2 Hz, 1H), 7.07 (td, *J* = 7.5, 1.2 Hz, 1H), 3.78 (dq, *J* = 14.4, 5.2 Hz, 1H), 3.54 (dd, *J* = 11.6, 7.5 Hz, 1H), 3.37 (dd, *J* = 11.5, 5.5 Hz, 1H), 3.10 (dd, *J* = 12.8, 4.3 Hz, 1H), 2.96 (dd, *J* = 12.8, 10.0 Hz, 1H).
LC/MS (APSI) m/z [M+H] calculated for C9H12NS: 166.0; found: 166.1.

(2R)-2-phenylmorpholine hydrochloride – Z1255382255. EN300-7547245 is a catalog building block, available from Enamine's stock.

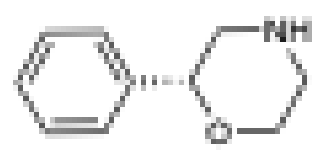

Purity, ≥98% (assessed by LC/MS).
^{1}H NMR (400 MHz, DMSO-d_6) δ 9.72 (s, 2H), 7.38 (s, 3H), 7.44 – 7.31 (m, 2H), 4.80 (dt, *J* = 11.3, 2.2 Hz, 1H), 4.14 – 4.06 (m, 1H), 4.02 – 3.90 (m, 1H), 3.38 (d, *J* = 12.6 Hz, 1H), 3.22 (s, 1H), 3.09 (tt, *J* = 11.9, 3.3 Hz, 1H), 3.02 – 2.91 (m, 1H).
LC/MS (APSI) m/z [M+H] calculated for C10H14NO: 164.0; found: 164.1.

rel-2-[(1R)-1,2,3,4-tetrahydronaphthalen-1-yl]pyrrolidine hydrochloride – Z8987197482. This compound was obtained by chiral separation of the racemic building block EN300-44829671, available from stock.

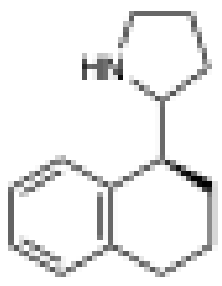

Purity, ≥95% (assessed by LC/MS).

^{1}H NMR (500 MHz, DMSO-d_6) δ 9.33 (s, 2H), 7.16 (dd, *J* = 23.9, 7.4 Hz, 2H), 7.09 (dd, *J* = 10.2, 7.5 Hz, 2H), 3.57 (td, *J* = 9.8, 5.0 Hz, 1H), 3.15 (qd, *J* = 5.6, 3.4 Hz, 2H), 3.11 (dd, *J* = 9.6, 4.8 Hz, 1H), 2.80 (ddd, *J* = 17.0, 6.8, 3.7 Hz, 1H), 2.69 (dt, *J* = 16.7, 8.2 Hz, 1H), 2.01 – 1.64 (m, 7H).

LC/MS (APSI) m/z [M+H] calculated for C14H20N: 202.2; found: 202.2.

2-phenylmorpholine – ZINCcj0000002gTq, Z283821656. EN300-29447 is a catalog building block, available from Enamine's stock.

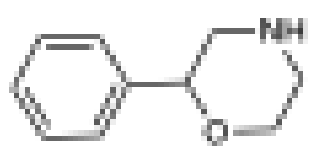

Purity, >95% (assessed by LC/MS).

^{1}H NMR (400 MHz, $CDCl_3$) δ 7.41 – 7.16 (m, 5H), 4.47 (dd, *J* = 10.3, 2.6 Hz, 1H), 4.02 (dd, *J* = 11.5, 3.4 Hz, 1H), 3.76 (td, *J* = 11.5, 2.7 Hz, 1H), 3.08 – 2.92 (m, 2H), 2.91 – 2.84 (m, 1H), 2.78 (dd, *J* = 12.4, 10.2 Hz, 1H), 1.93 (s, 1H).

LC/MS (APSI) m/z [M+H] calculated for C10H14NO: 164.0; found: 164.1.

2-(pyridin-4-yl)morpholine dihydrochloride – ZINCcd0000002gVM, Z3235143302 is a catalog building block, available from Enamine's stock.

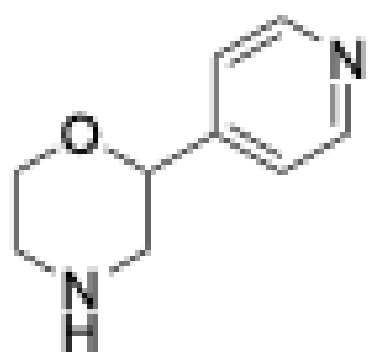

Purity, >95% (assessed by LC/MS).

^{1}H NMR (500 MHz, DMSO-d_6) δ 9.92 (s, 1H), 8.89 – 8.84 (m, 2H), 7.89 (d, J = 5.9 Hz, 2H), 5.13 – 5.07 (m, 1H), 4.18 (dd, J = 12.8, 3.8 Hz, 1H), 4.01 (t, J = 11.9 Hz, 1H), 3.65 (d, J = 12.5 Hz, 1H), 3.26 (d, J = 12.8 Hz, 1H), 2.95 (d, J = 9.7 Hz, 1H).

LC/MS (APSI) m/z [M+H] calculated for C9H13N2O: 165.2; found: 165.1.

[(2,3-dihydro-1H-inden-1-yl)methyl](methyl)amine – ZINCcp0000002wxn, Z1251362220 is a

catalog building block, available from Enamine's stock.

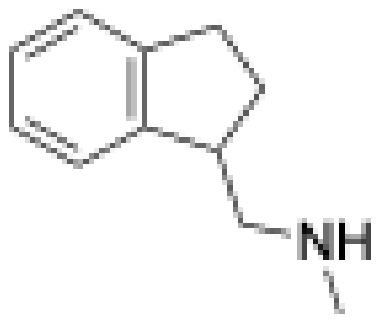

Purity, >95% (assessed by LC/MS).

^{1}H NMR (500 MHz, DMSO-d_6) δ 9.07 (s, 1H), 8.99 (s, 1H), 7.31 – 7.21 (m, 2H), 7.21 – 7.13 (m, 2H), 3.49 (dtd, J = 11.1, 7.3, 4.0 Hz, 1H), 3.31 (dd, J = 8.4, 4.1 Hz, 1H), 2.91 (ddt, J = 14.2, 10.8, 4.6 Hz, 2H), 2.81 (dt, J = 15.8, 7.7 Hz, 1H), 2.57 (t, J = 5.2 Hz, 3H), 2.27 (dtd, J = 13.1, 8.1, 5.2 Hz, 1H), 1.91 (ddd, J = 15.4, 13.1, 7.4 Hz, 1H).

LC/MS (APSI) m/z [M+H] calculated for C11H16N: 162.2; found: 162.1.

Spectral Data

Library Search Report

```
Data File : C:\MSDCHEM\1\DATA\G04_06\R420462.D          Vial: 91
Acq On    :  6 Apr 2015  11:46                          Operator:
Sample    : R420462                                     Inst     : Instrumen
Misc      : CH3OH                                       Multiplr: 1.00
                                                   Sample Amount: 0.00
MS Integration Params: autoint1.e
Method    : C:\MSDCHEM\1\METHODS\UNIVERS.M (Chemstation Integrator)
Title     :
```

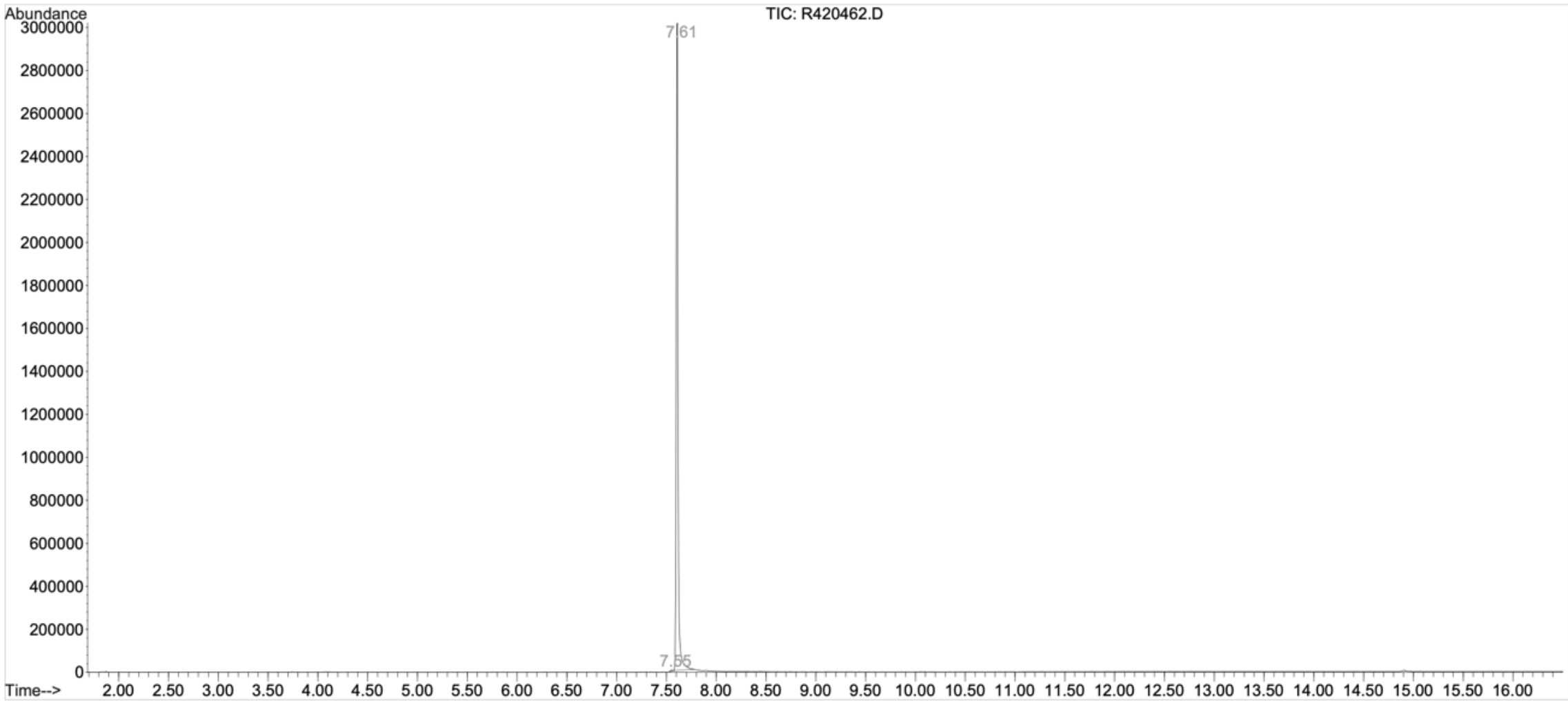

Library Search Report - Chemstation Integrator

Unknown Spectrum based on Apex

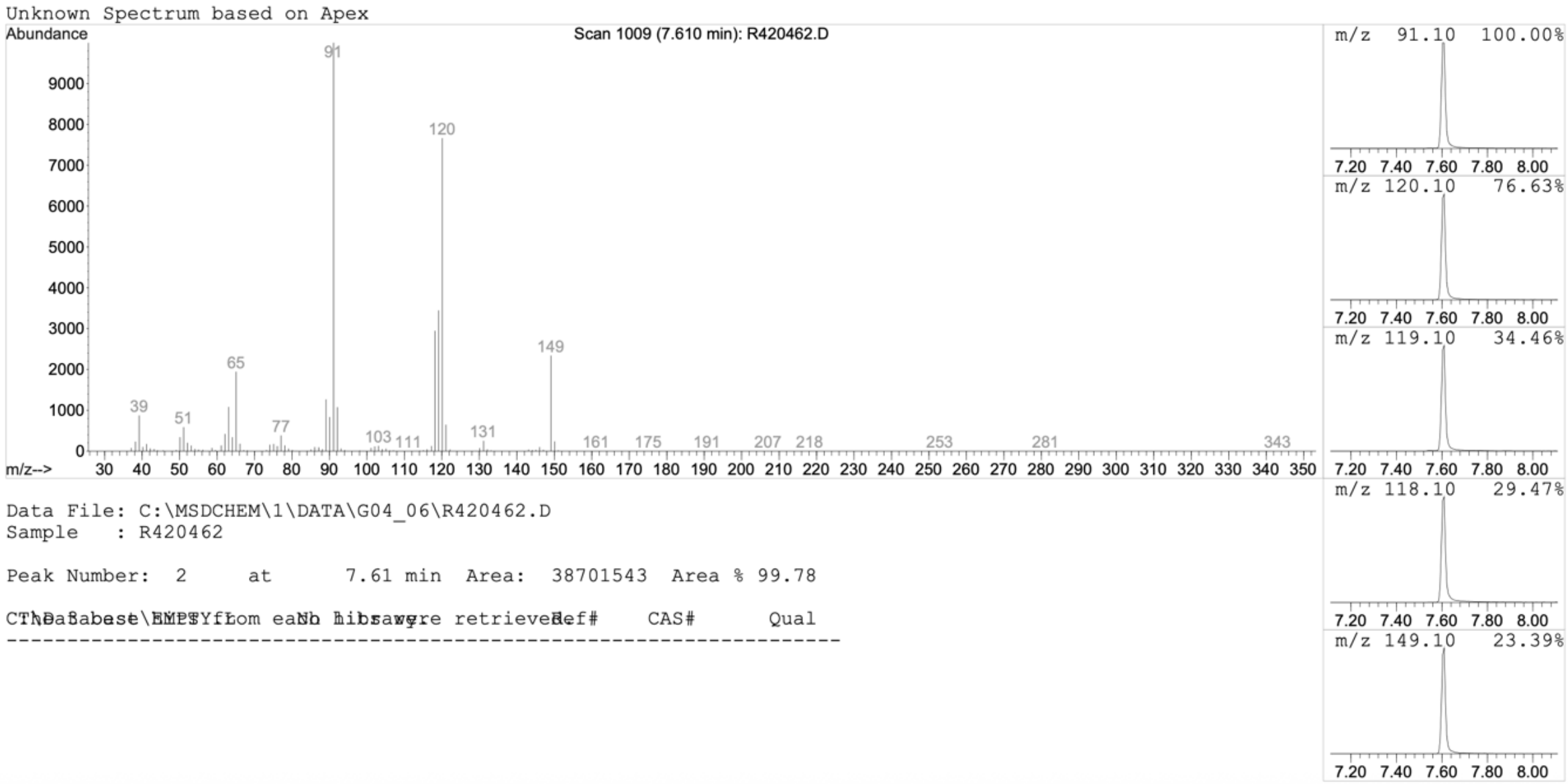


```
Data File: C:\MSDCHEM\1\DATA\G04_06\R420462.D
Sample   : R420462

Peak Number:  2     at      7.61 min   Area:   38701543  Area % 99.78

CThDaSabese\EMPTYfLom eaNo librawyre retrieveRef#    CAS#      Qual
----------------------------------------------------------------------
```

Z1197877490

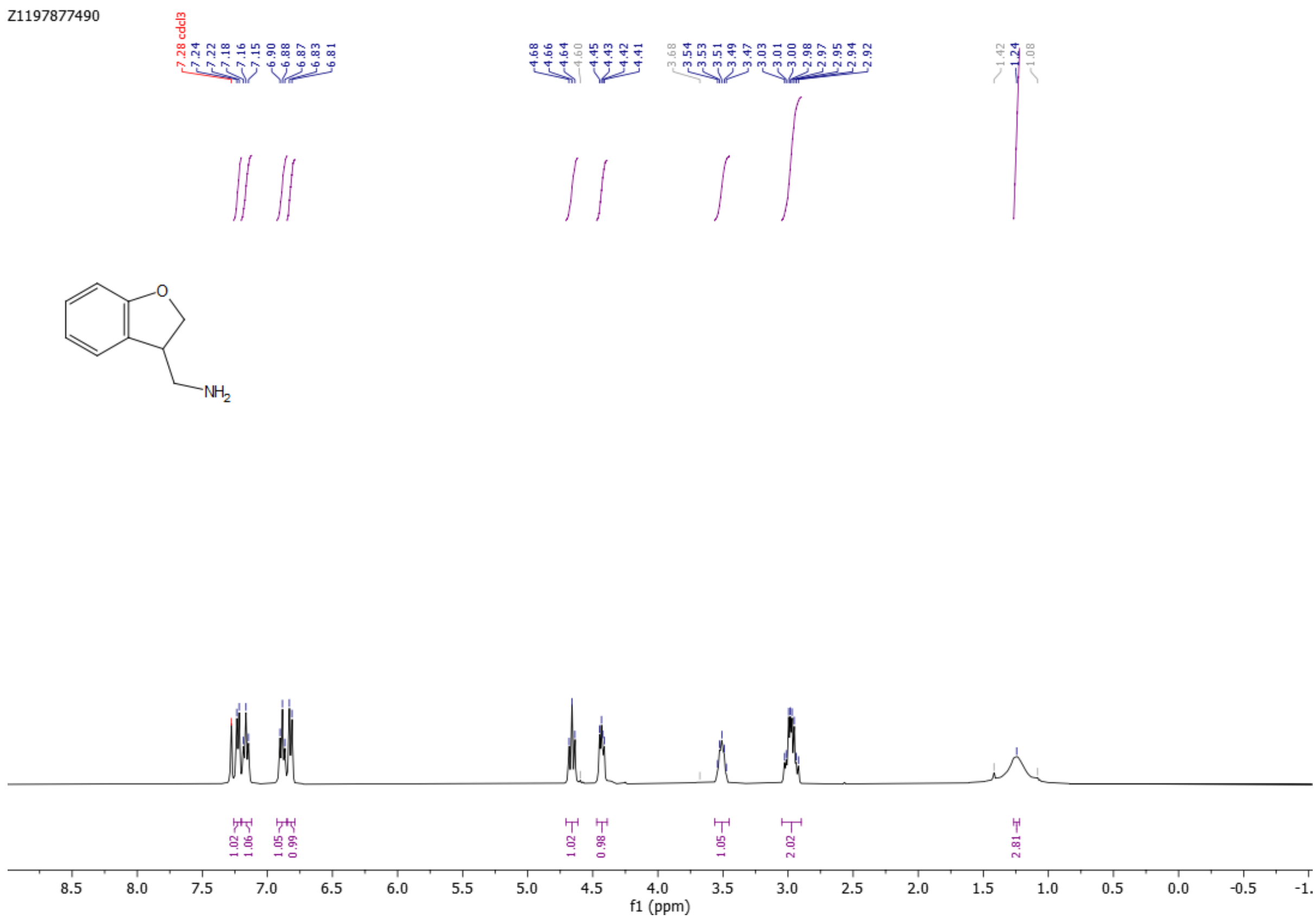

Z1197877490

MaxPeak: 51.60%
Ret_Time: 0.695 min

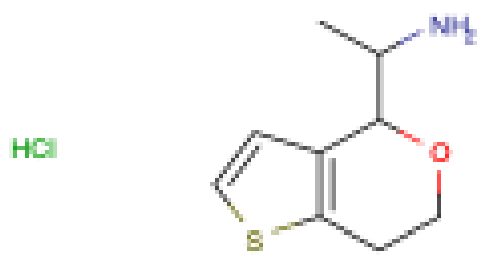

Mol Wt 219.73
Exact Mass 183.09

#	Time	Area%
1	0.646	48.40
2	0.695	51.60

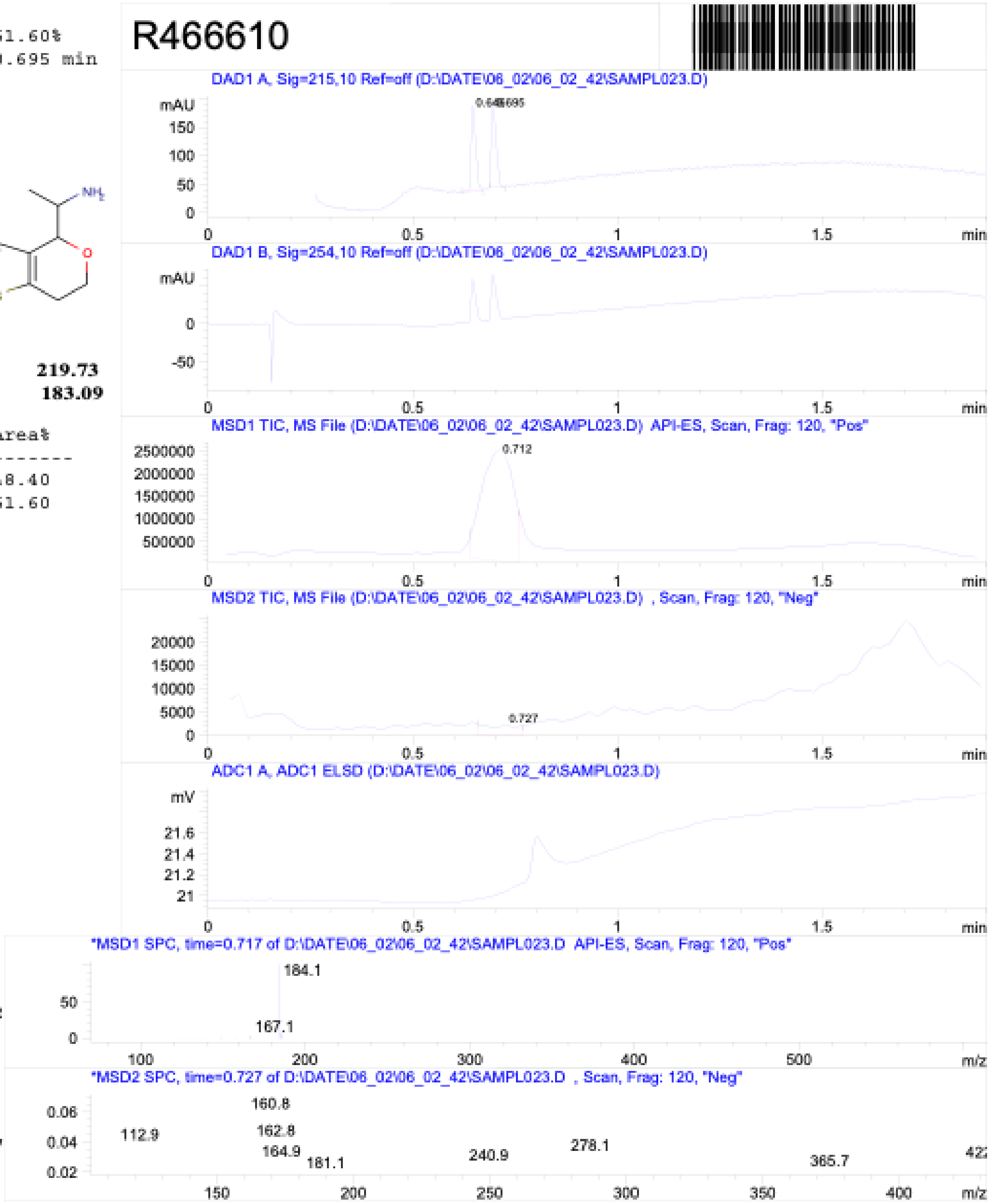

RT 0.712

RT 0.727

Inj.Date 6/3/2015 L P2-C-06 SL Acq. Method C:\HPCHEM\-> ->

Z2211231413

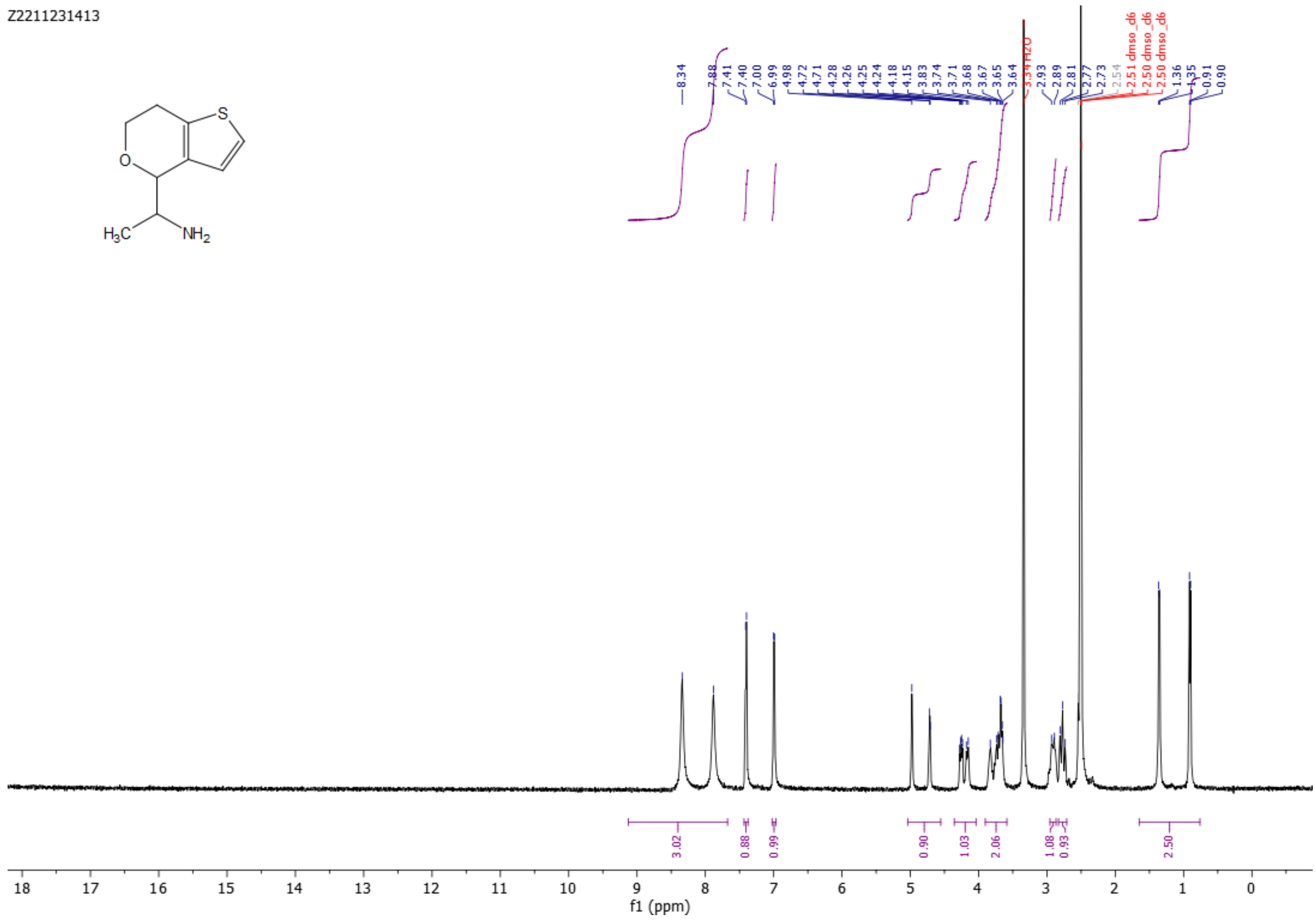

Z2211231413

Error: Peaks not found!

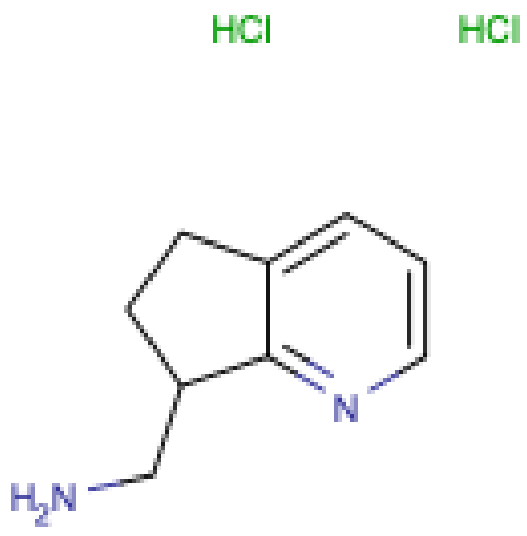

Mol Wt 221.13

Exact Mass 148.12

Error: Peaks not found!

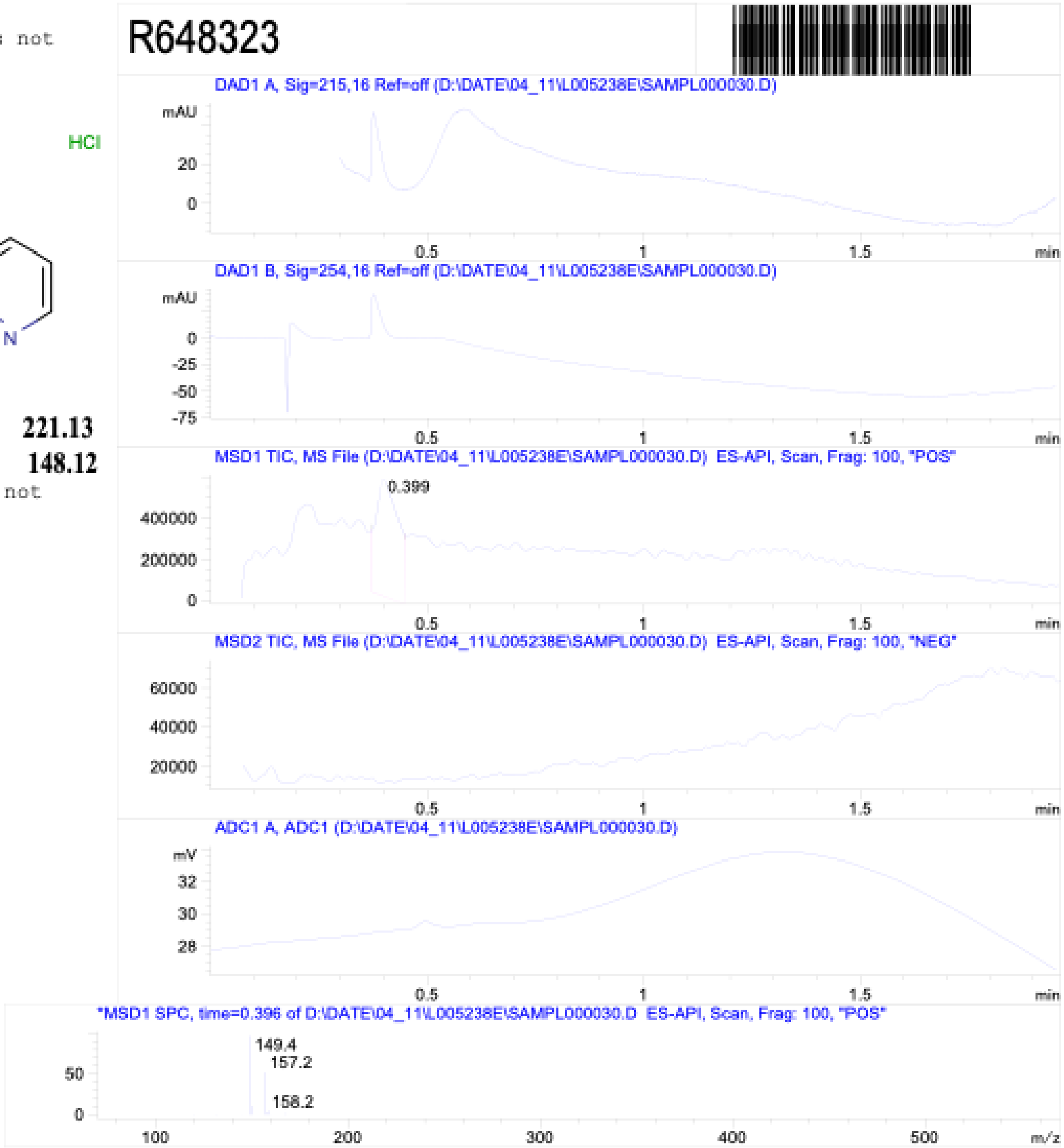

RT 0.399

Inj.Date 4/10/2016 E P2-D-06 - 4 - Acq. Method C:\CHEM32\-> ->

Z2489361097

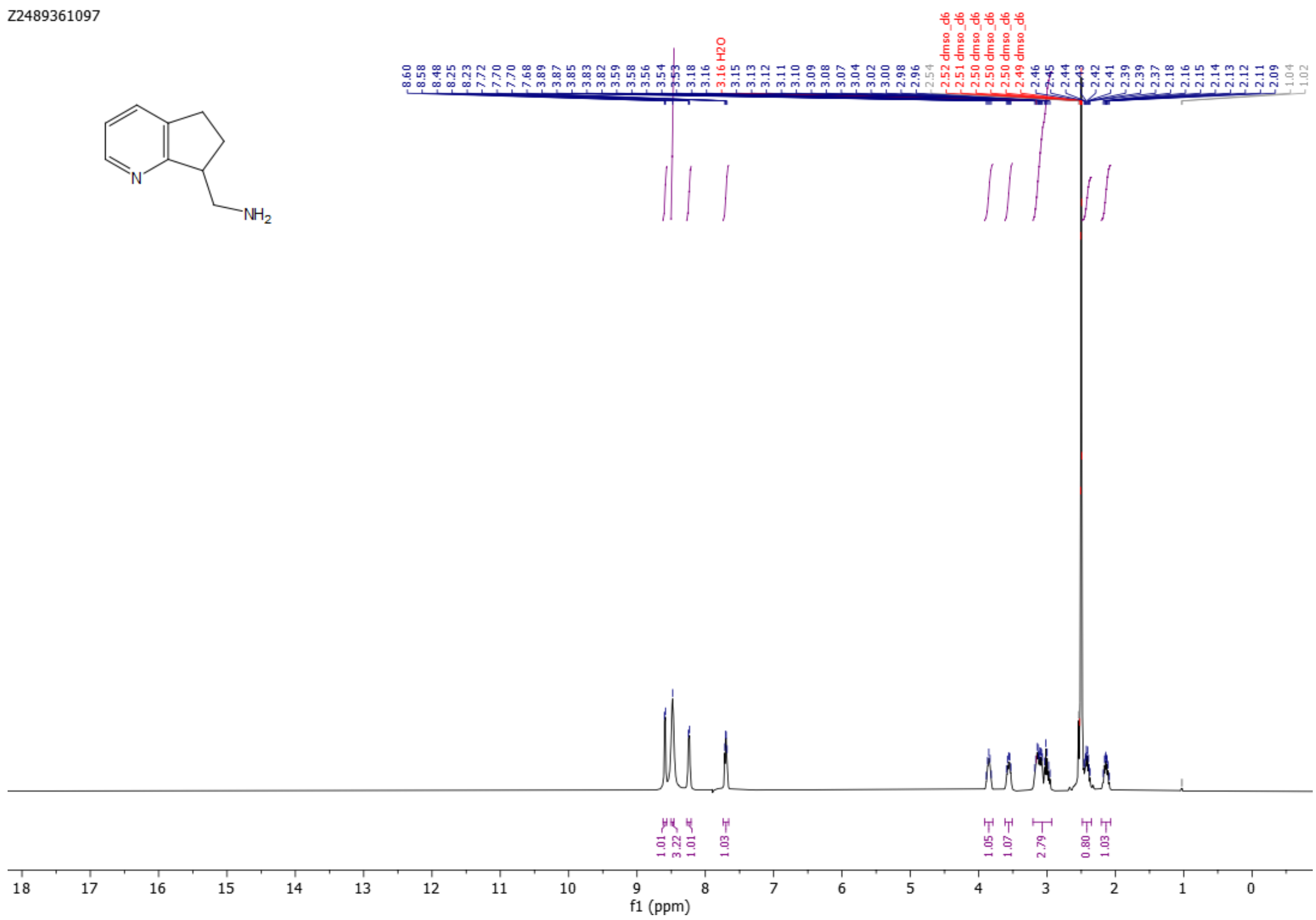

Z2489361097

MaxPeak: 100.00%
Ret_Time: 0.142 min

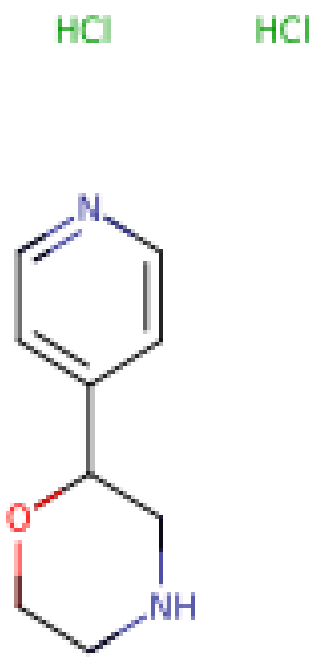

Mol Wt 237.13
Exact Mass 164.11

#	Time	Area%
1	0.142	100.00

RT 0.152

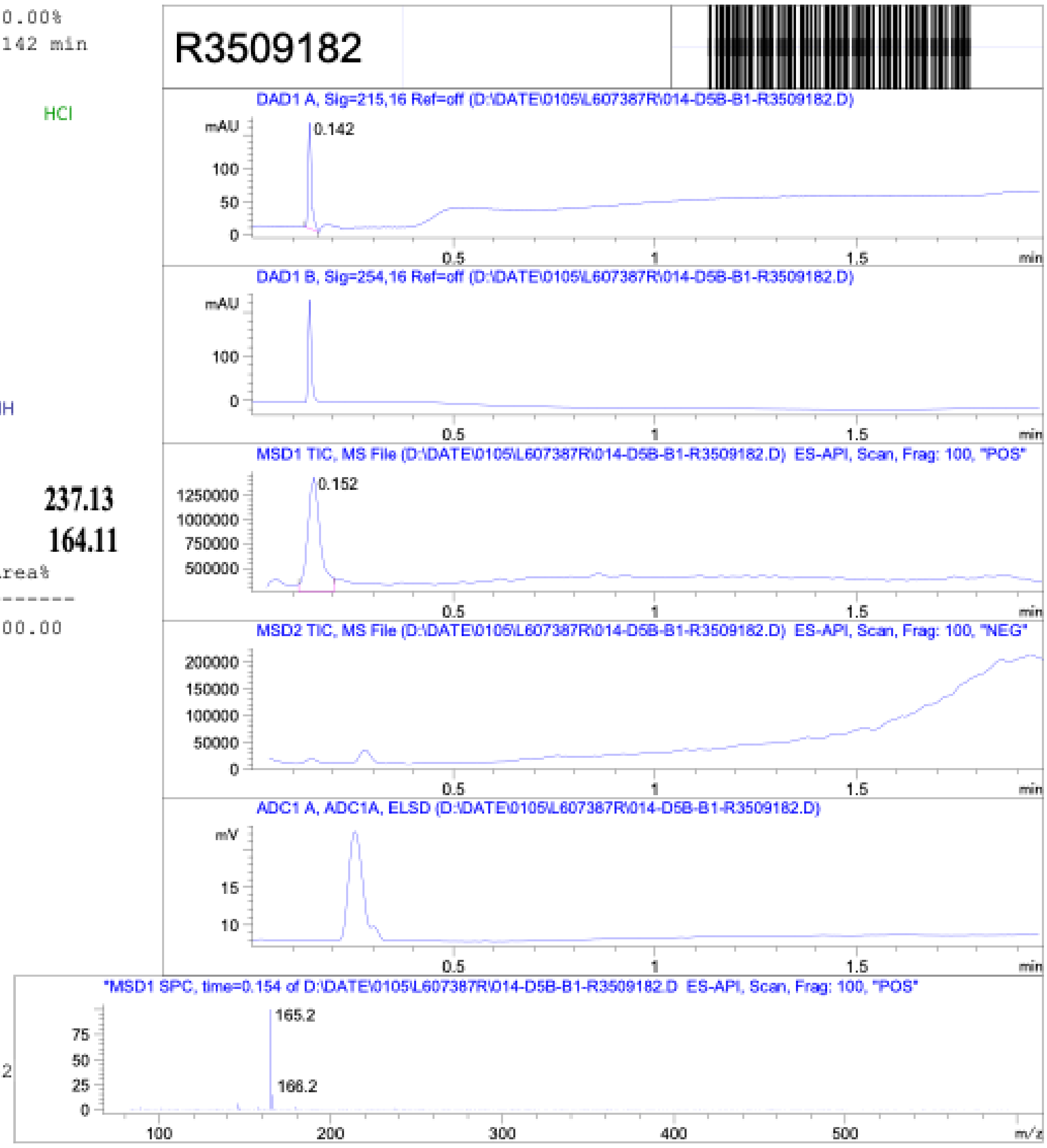

Inj.Date 5/1/2023 CH -5- Acq. Method C:\Users\ -> ->

Z3235143302

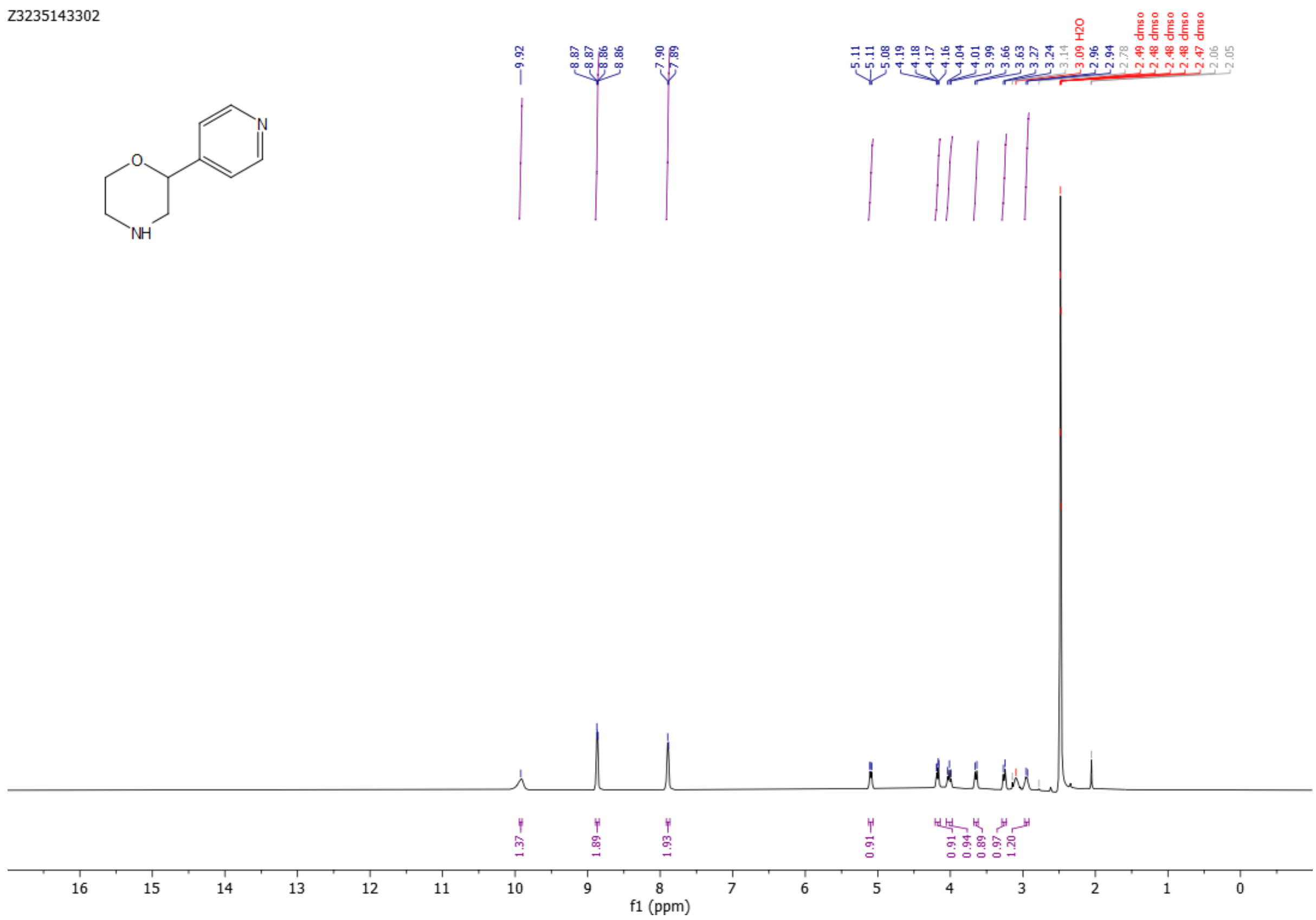

Z3235143302

MaxPeak: 100.00%
Ret_Time: 0.917 min

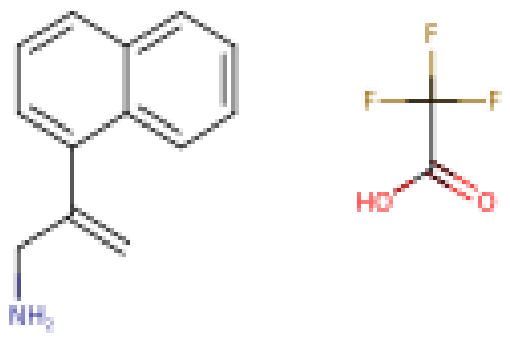

Mol Wt 297.27
Exact Mass 183.13

#	Time	Area%
1	0.917	100.00

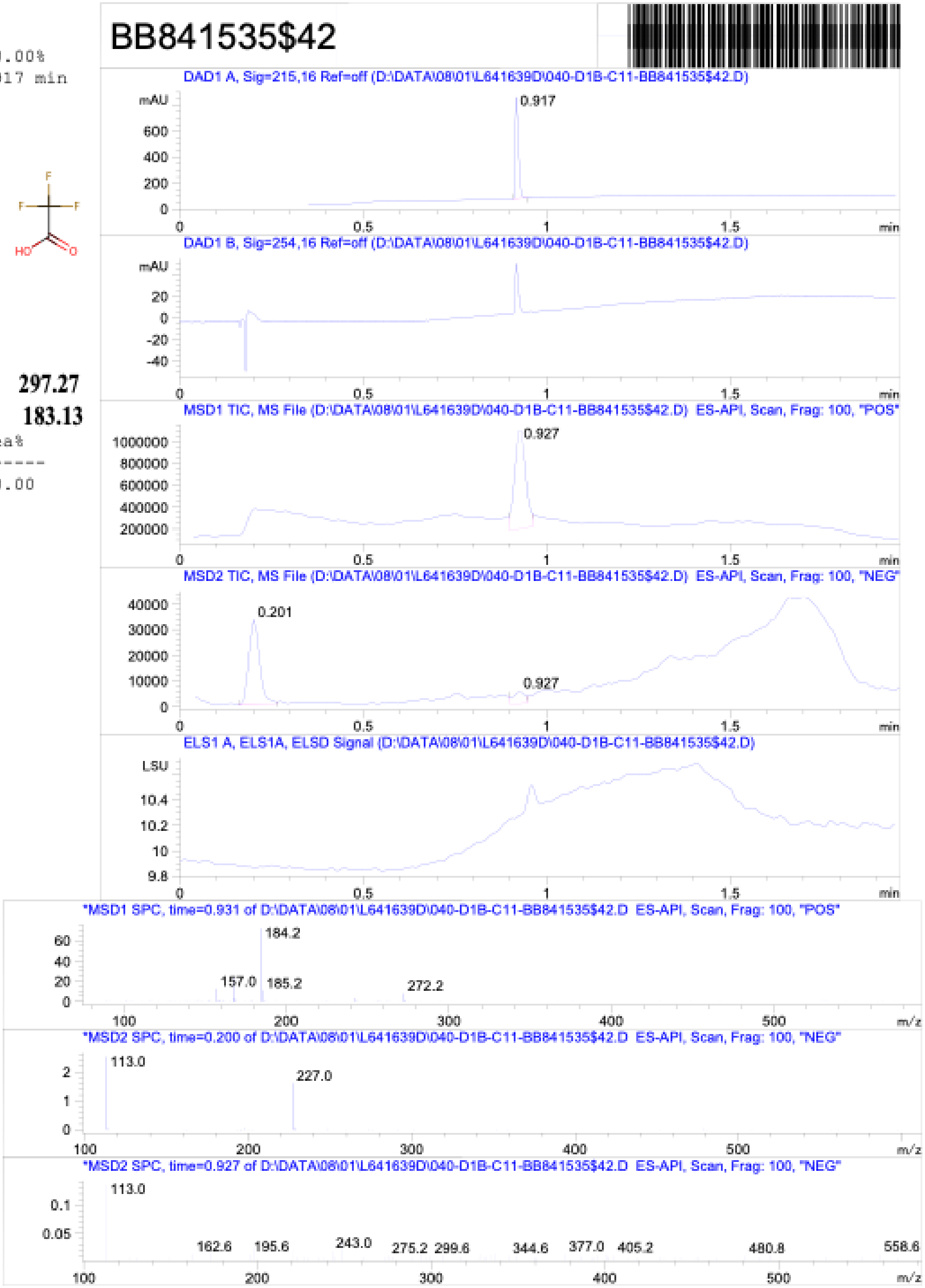

RT 0.927

RT 0.201

RT 0.927

Inj.Date 8/1/2023 CH <invalid> -26- Acq. Method C:\Users\ -> ->

Z8711777920

Error: Peaks not found!

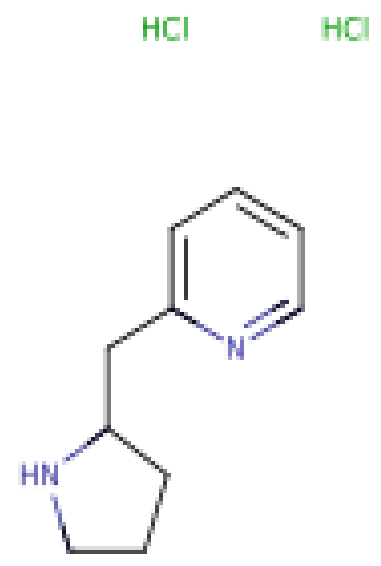

Mol Wt 235.15

Exact Mass 162.14

Error: Peaks not found!

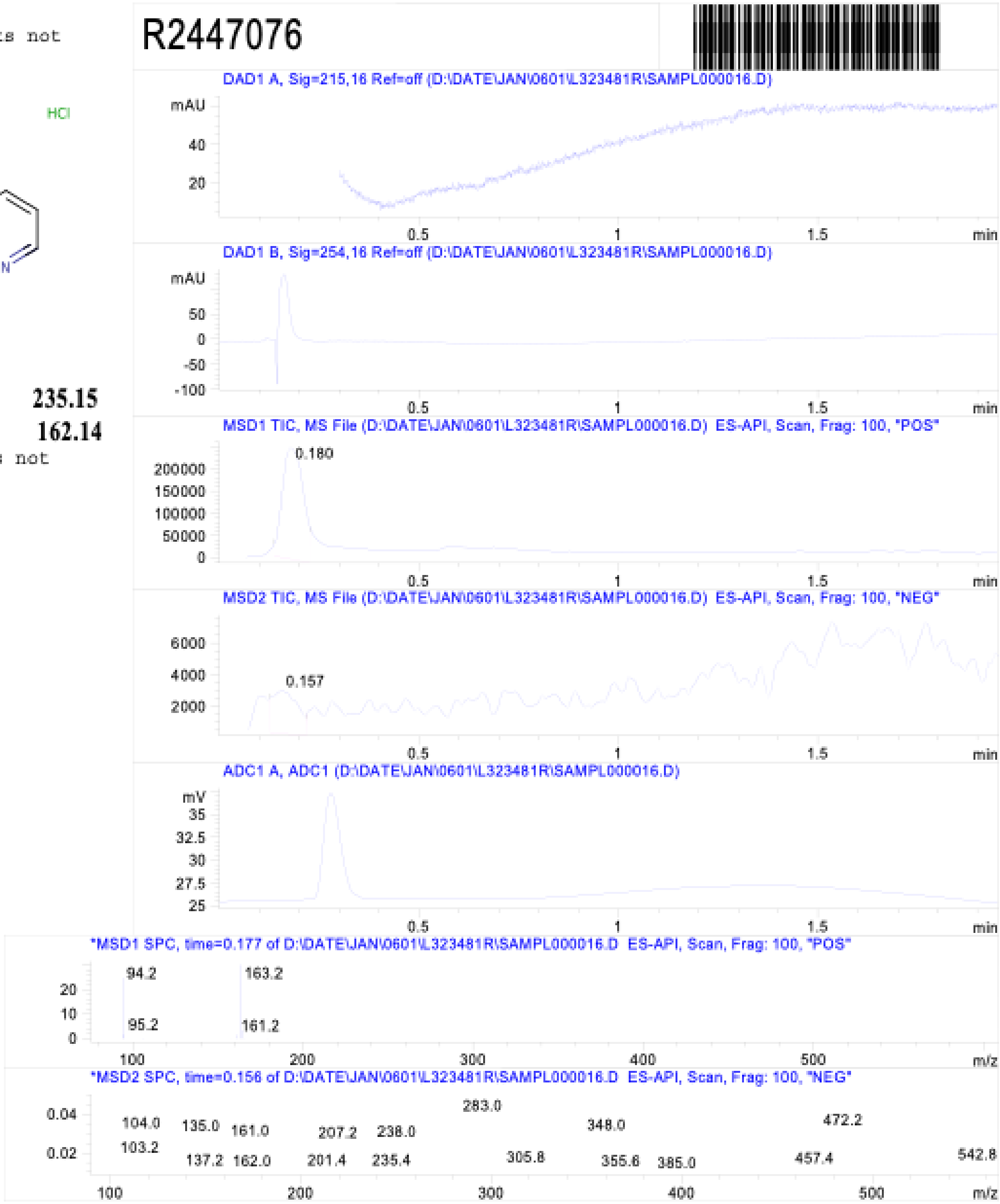

RT 0.180

RT 0.157

Inj.Date 1/6/2021 Y - 4 - Acq. Method C:\CHEM32\-> ->

Z3214214254

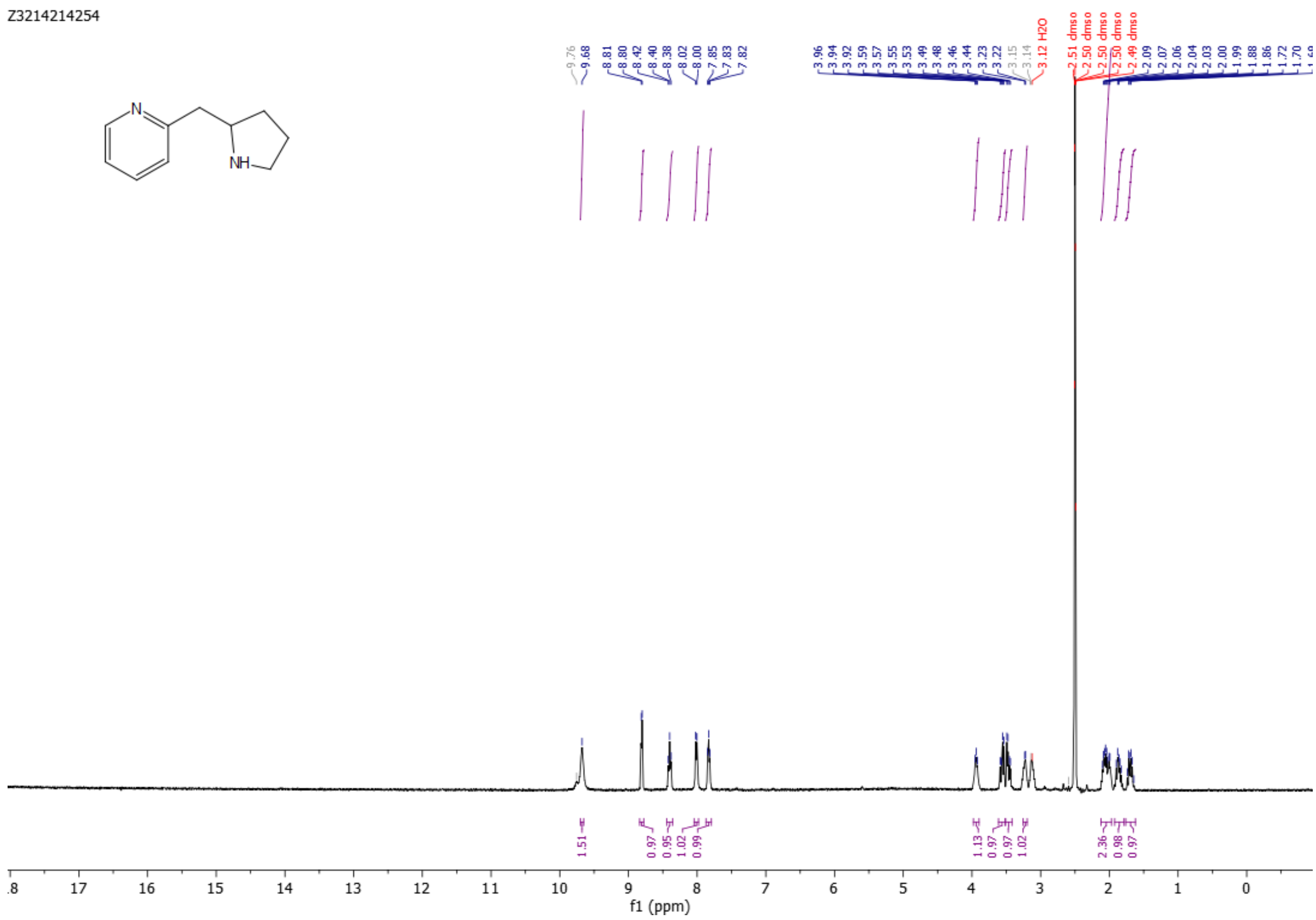

Z3214214254

MaxPeak: 100.00%
Ret_Time: 0.586 min

R2573207

HCl

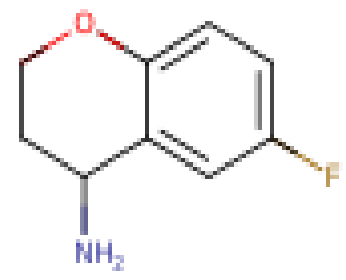

Mol Wt 203.64
Exact Mass 167.09

#	Time	Area%
1	0.586	100.00

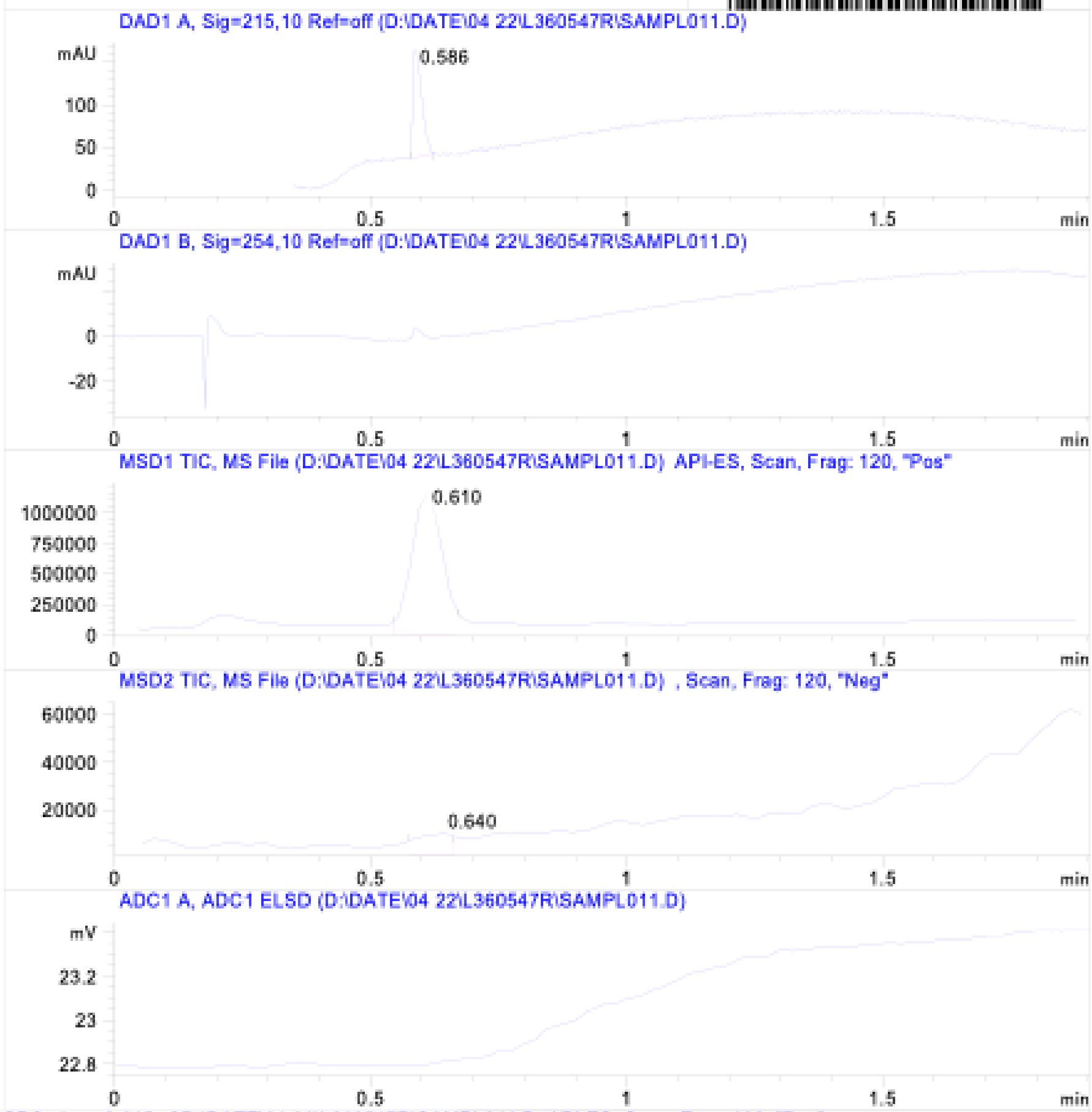

RT 0.610

RT 0.640

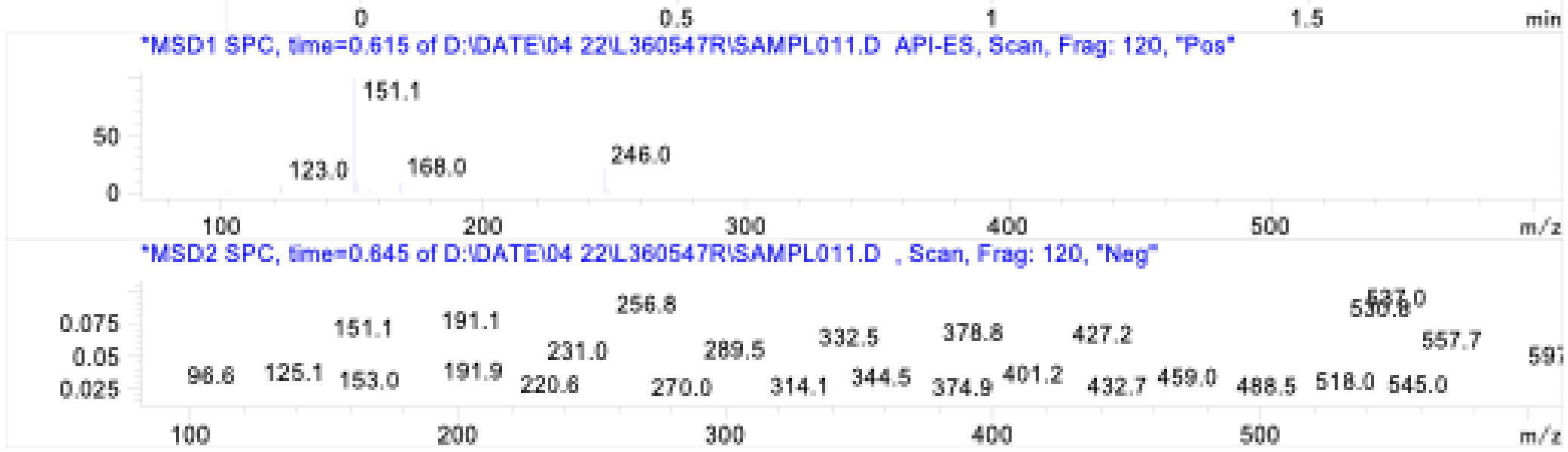

Inj.Date 4/23/2021 E -VL- Acq. Method C:\HPCHEM\-> ->

Z1266854953

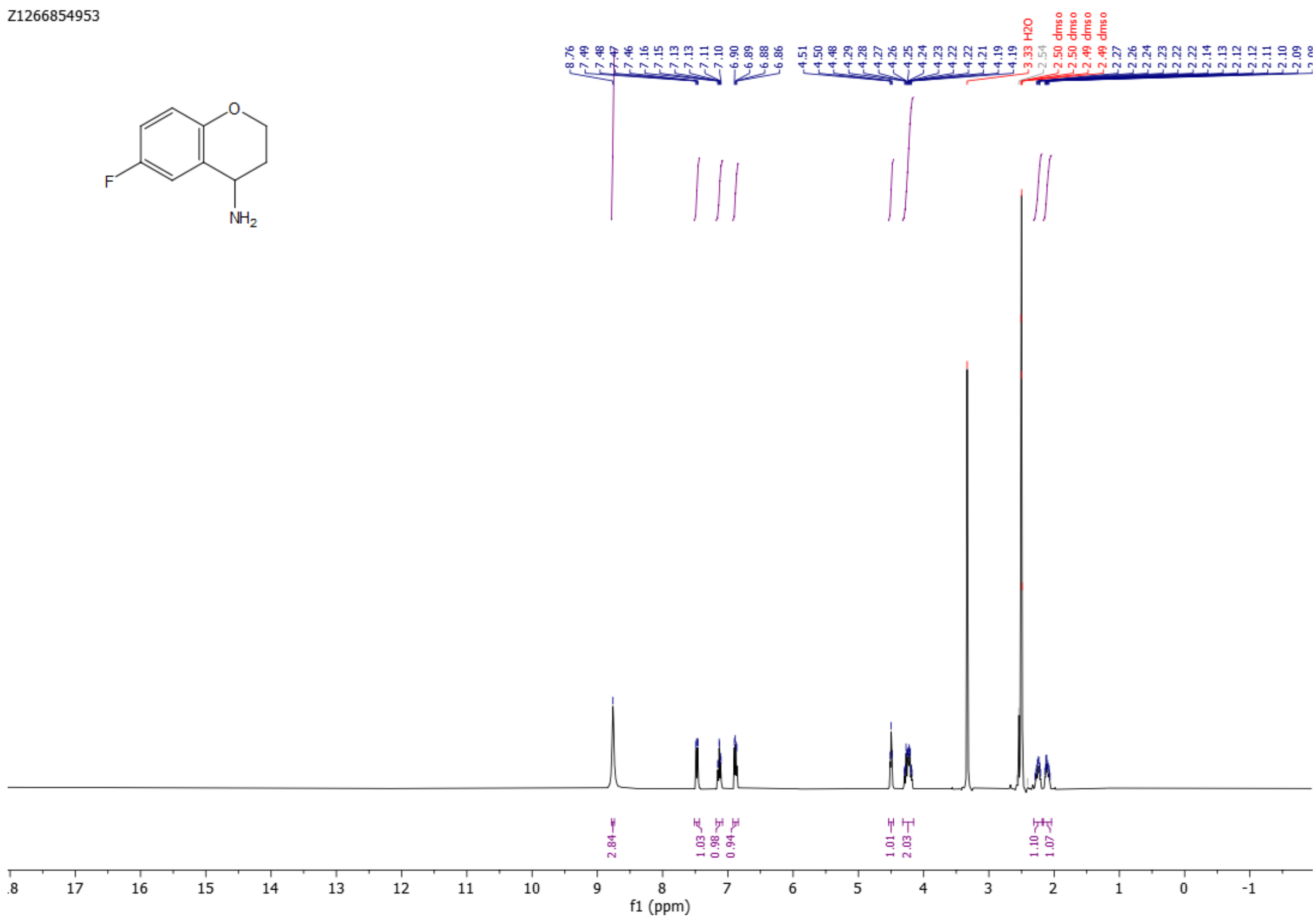

Z1266854953

MaxPeak: 99.10%
Ret_Time: 0.539 min

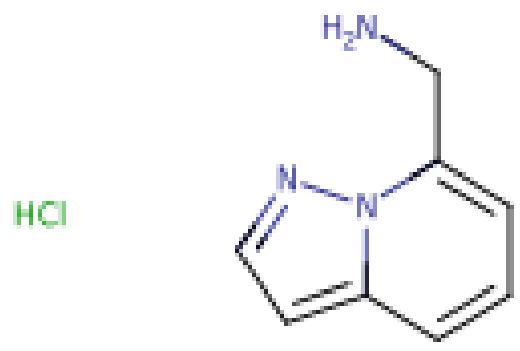

Mol Wt 183.64
Exact Mass 147.09

#	Time	Area%
1	0.539	99.10
2	0.766	0.90

R2384029

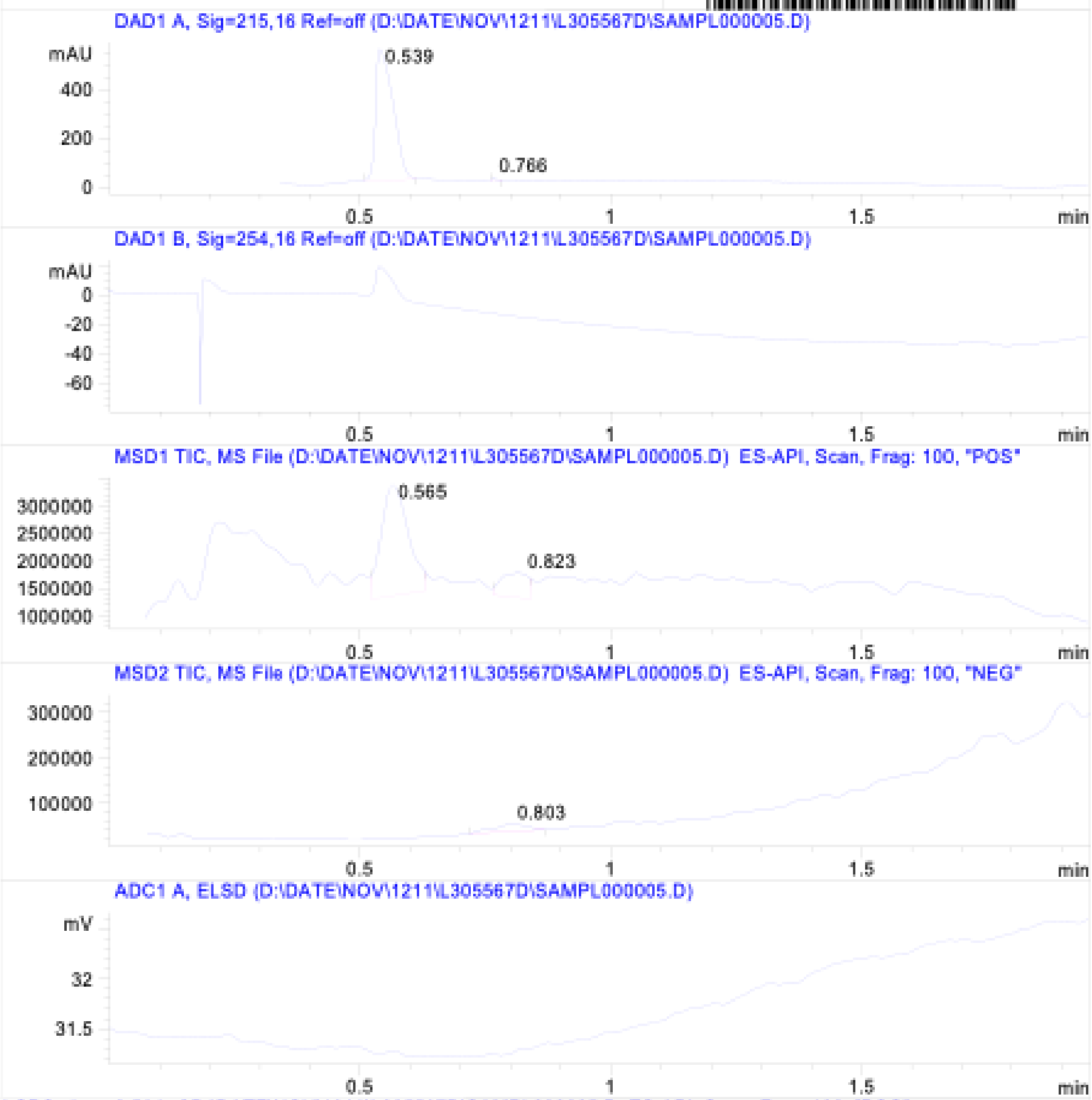

RT 0.565

RT 0.823

RT 0.803

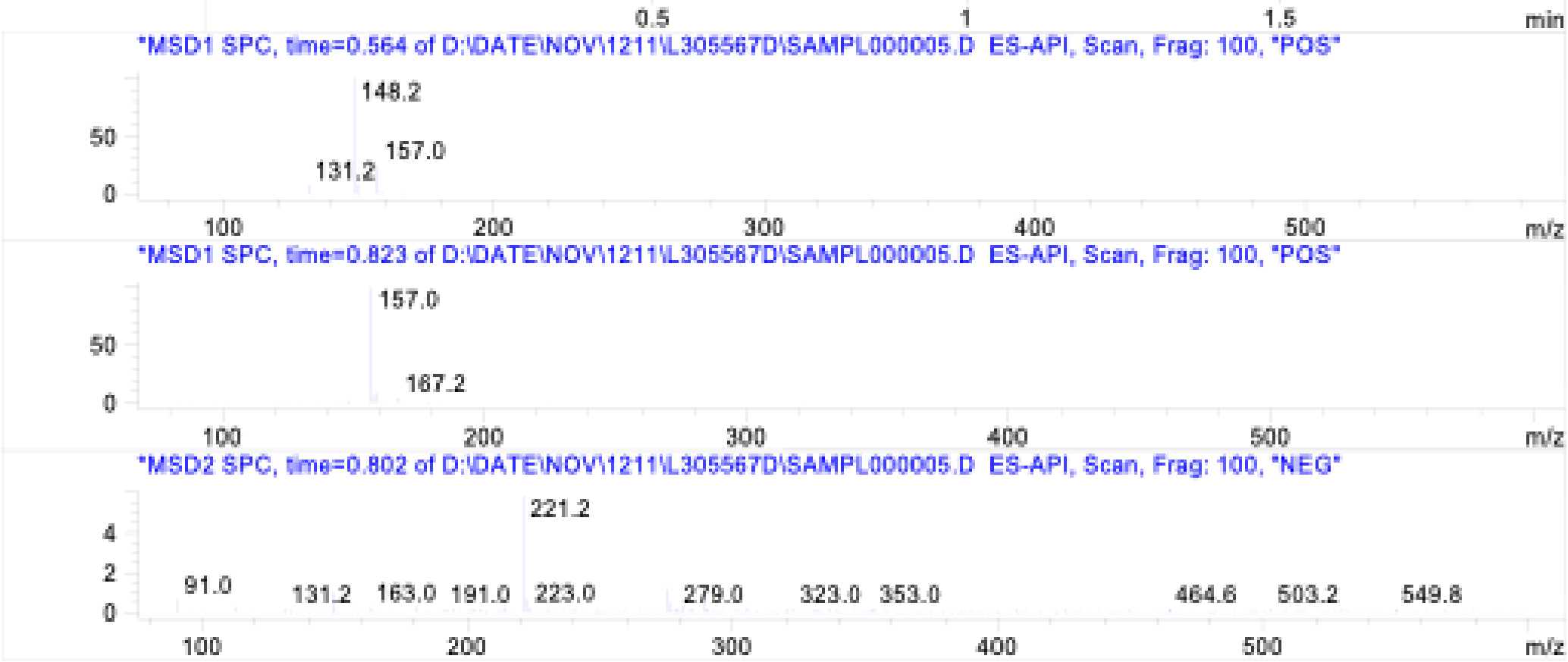

Inj.Date 11/12/2020 Y -3- Acq. Method C:\CHEM32\-> ->

Z4244625472

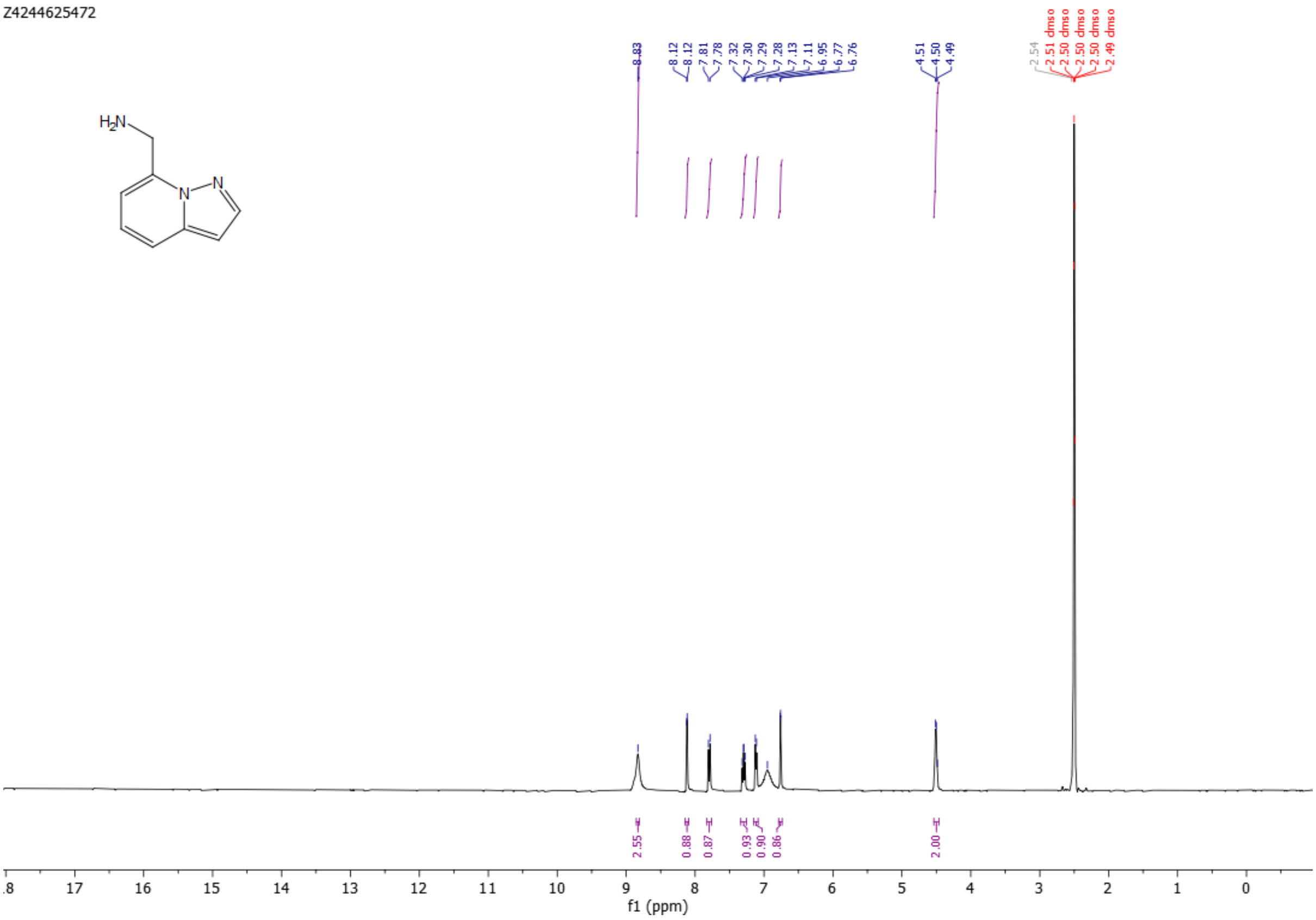

Z4244625472

MaxPeak: 98.06%
Ret_Time: 0.827 min

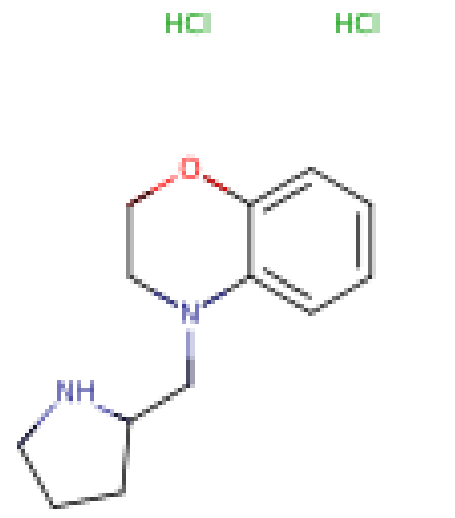

Mol Wt 291.22
Exact Mass 218.17

#	Time	Area%
1	0.827	98.06
2	0.849	1.94

RT 0.838

BB947953$1

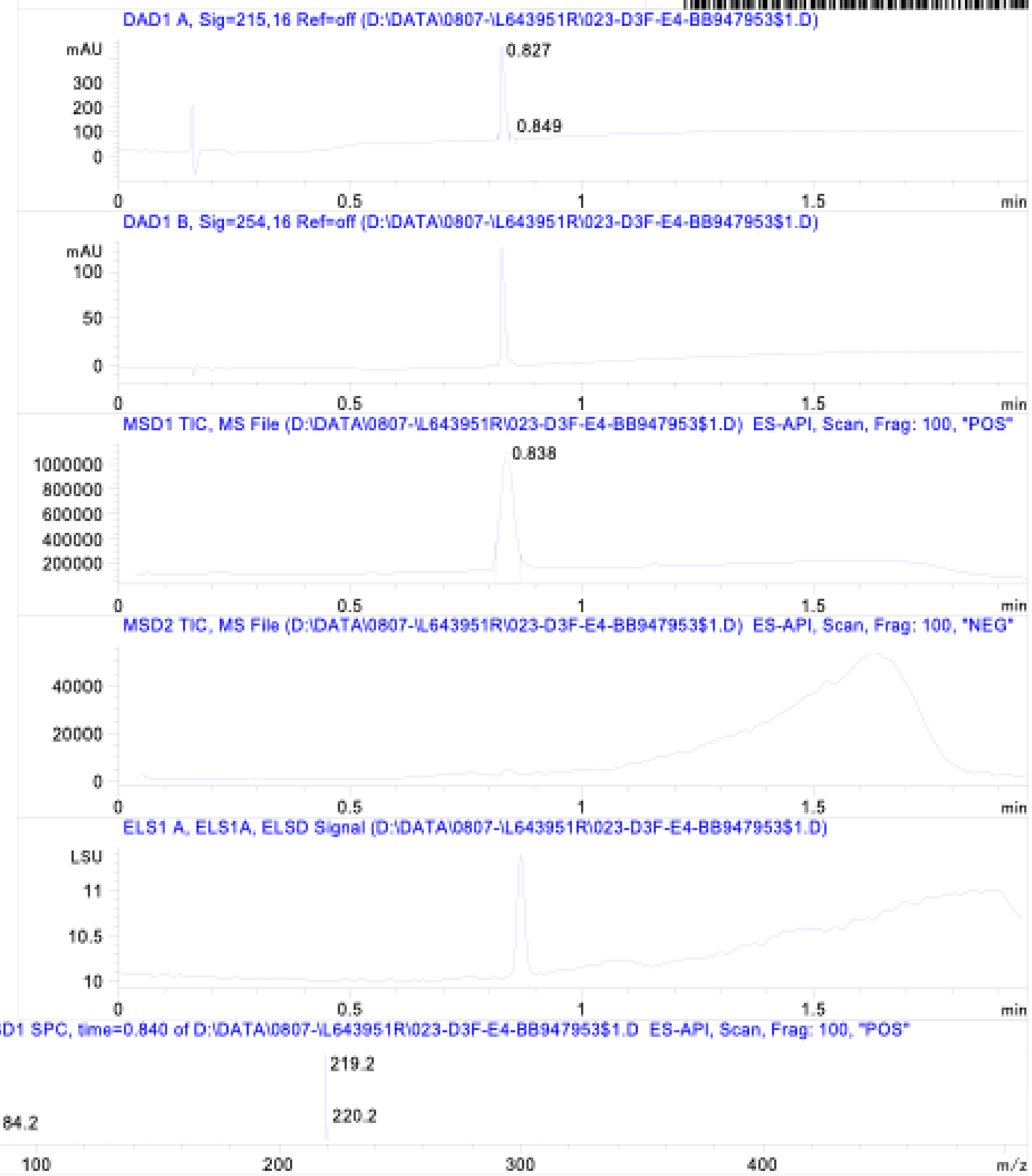

Inj.Date 8/7/2023 H -26- Acq. Method C:\Users\ -> ->

Z8700802749

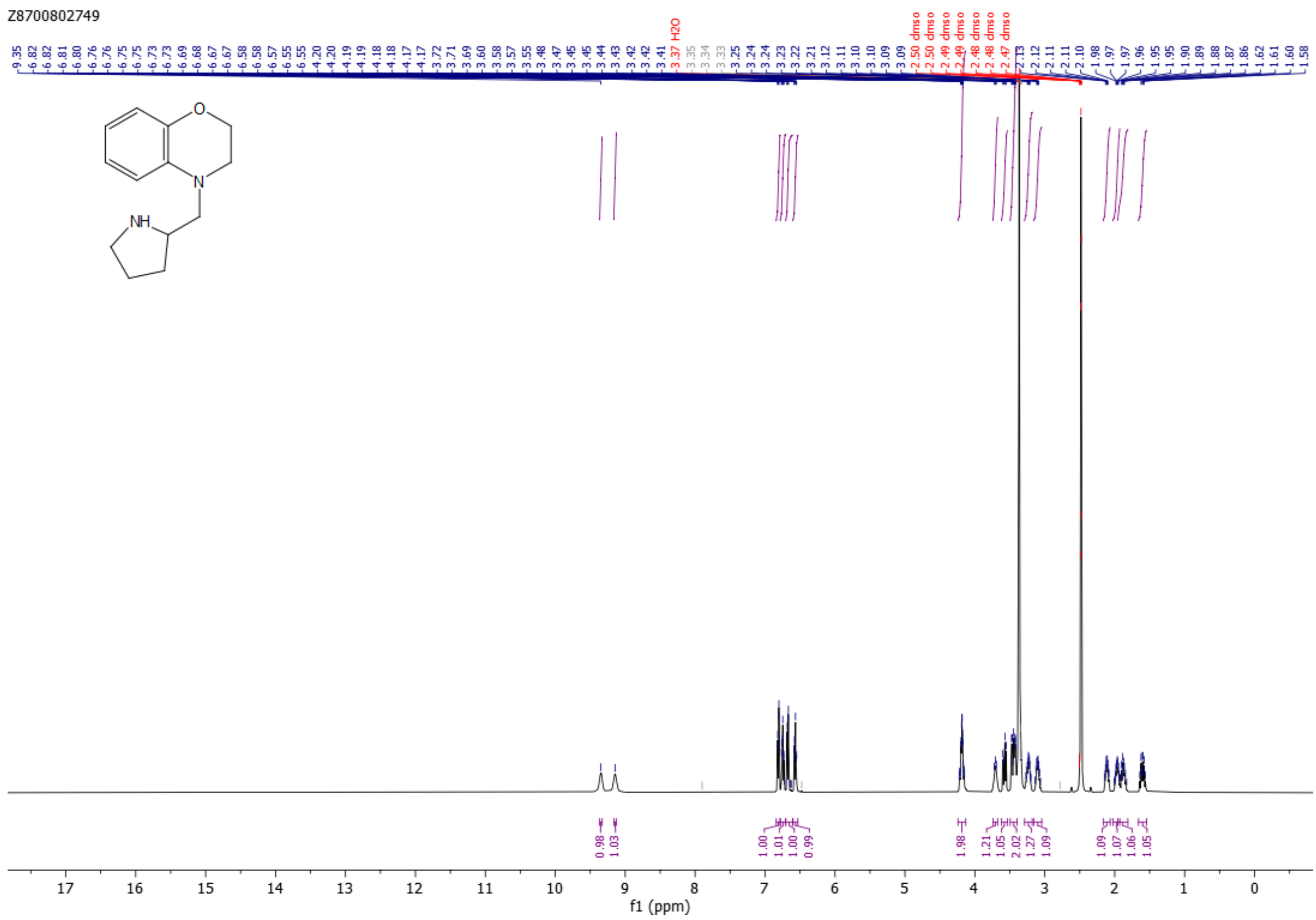

Z8700802749

MaxPeak: 100.00%
Ret_Time: 0.616 min

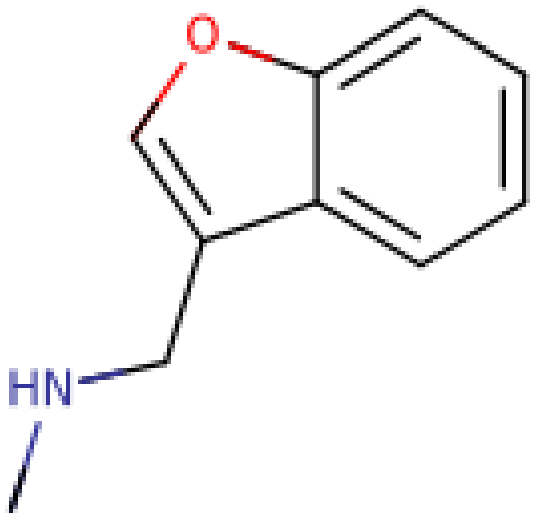

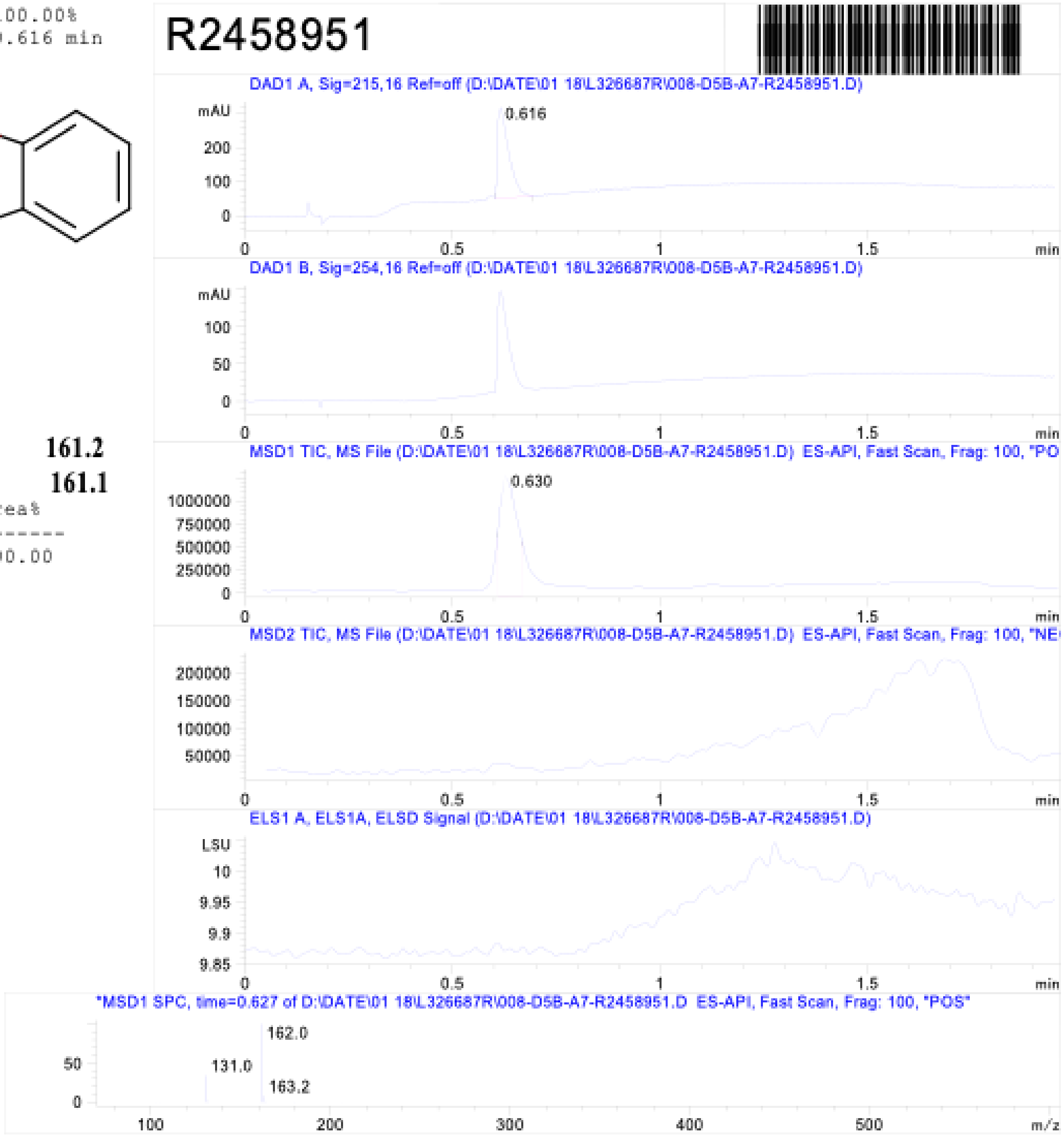

Mol Wt 161.2
Exact Mass 161.1

#	Time	Area%
1	0.616	100.00

RT 0.630

Inj.Date 1/18/2021 E Acq. Method C:\Users\ -> ->

Z1197877493

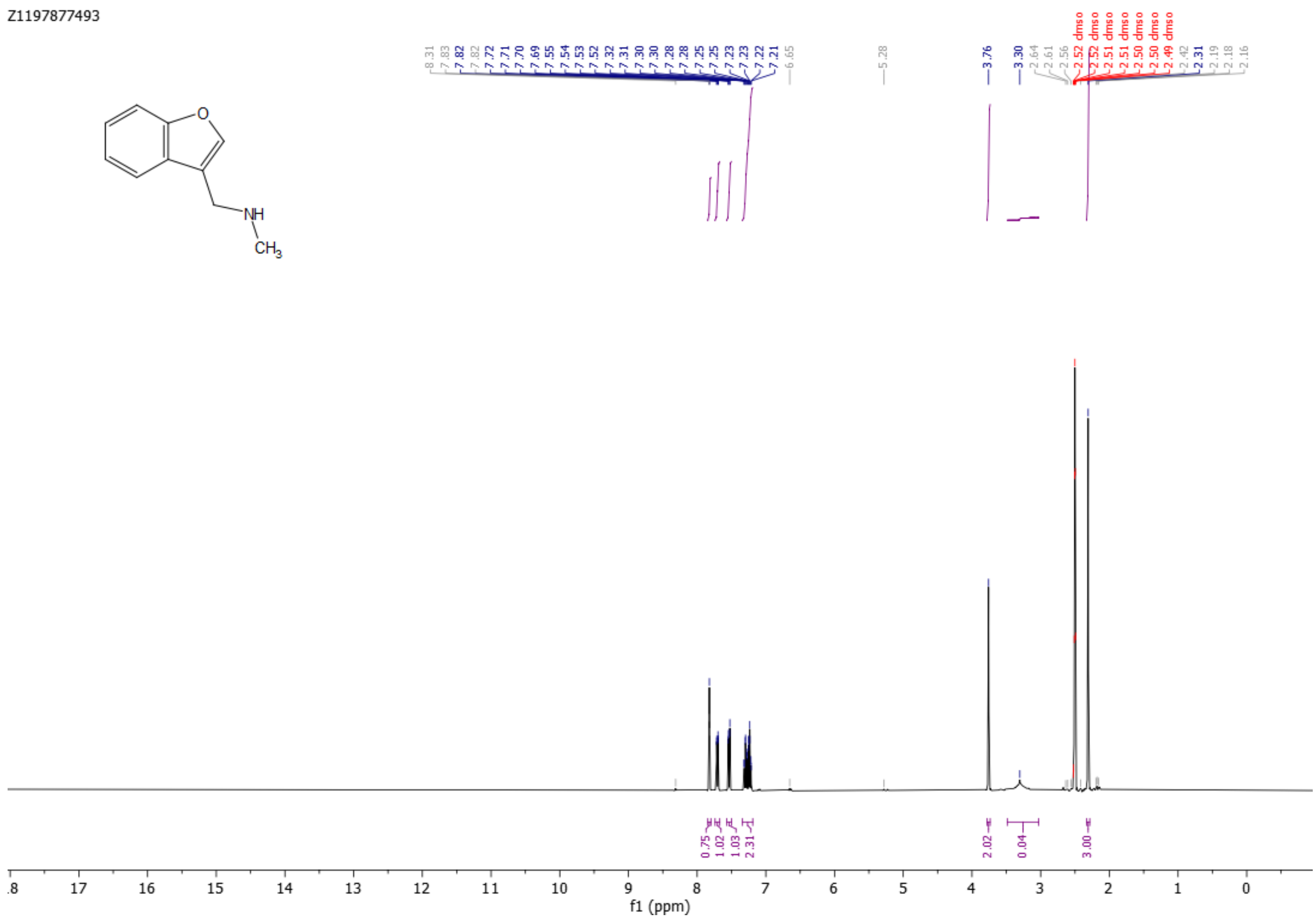

Z1197877493

MaxPeak: 100.00%
Ret_Time: 0.572 min

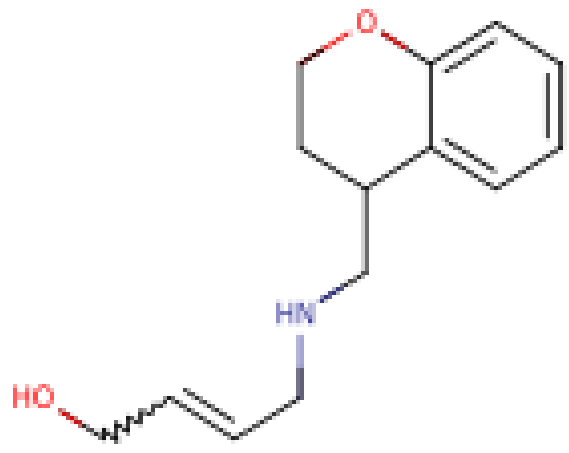

Mol Wt 233.31
Exact Mass 233.17

#	Time	Area%
1	0.572	100.00

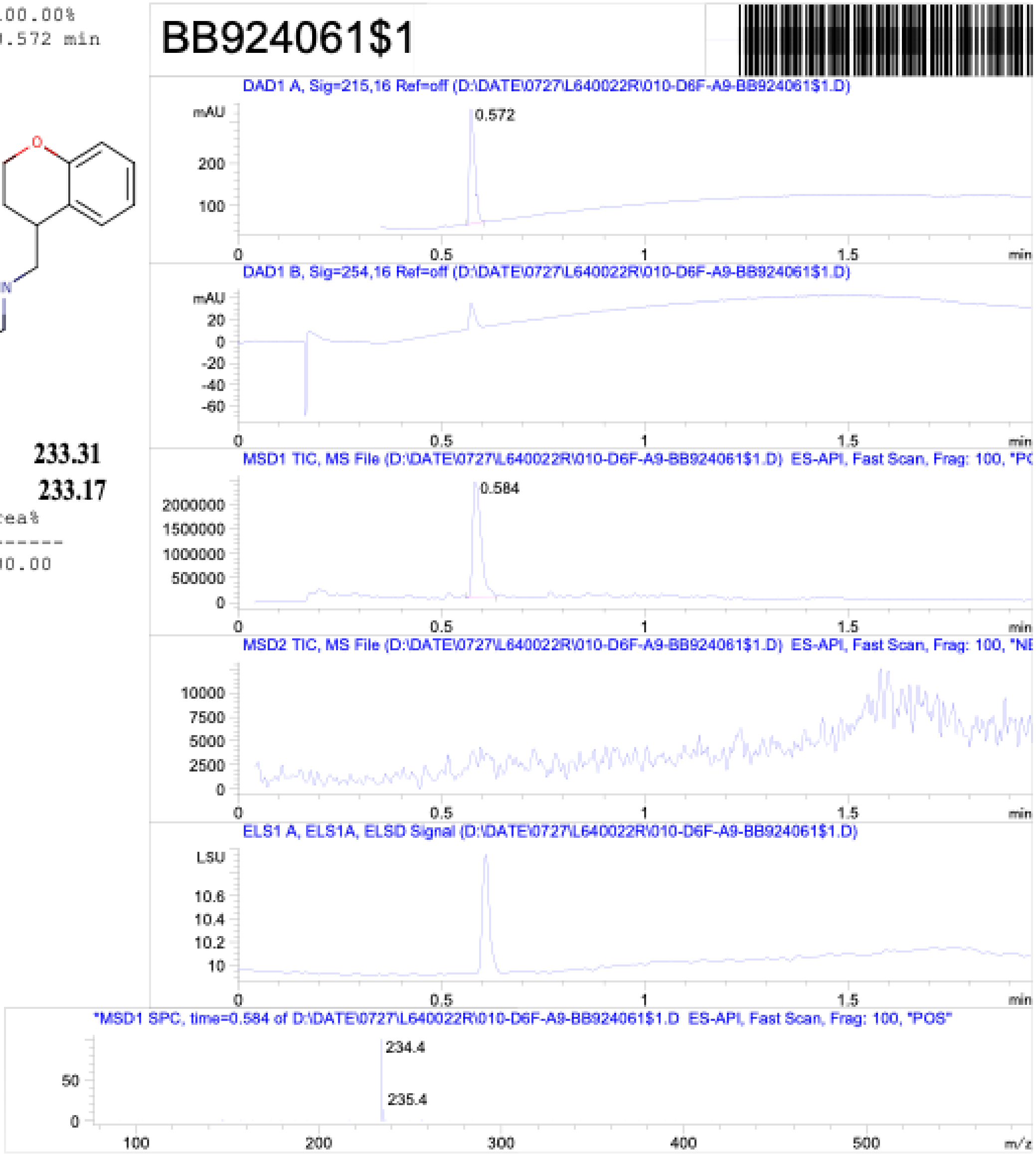

RT 0.584

Inj.Date 7/27/2023 E 28 Acq. Method C:\Users\ -> ->

Z2590548459

MaxPeak: 97.81%
Ret_Time: 0.620 min

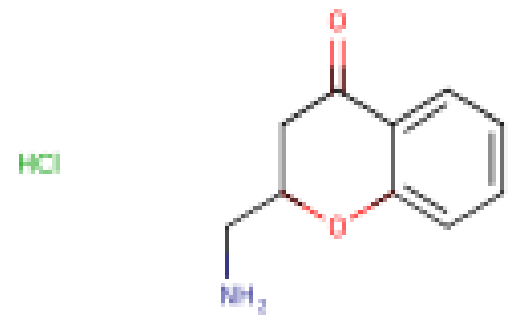

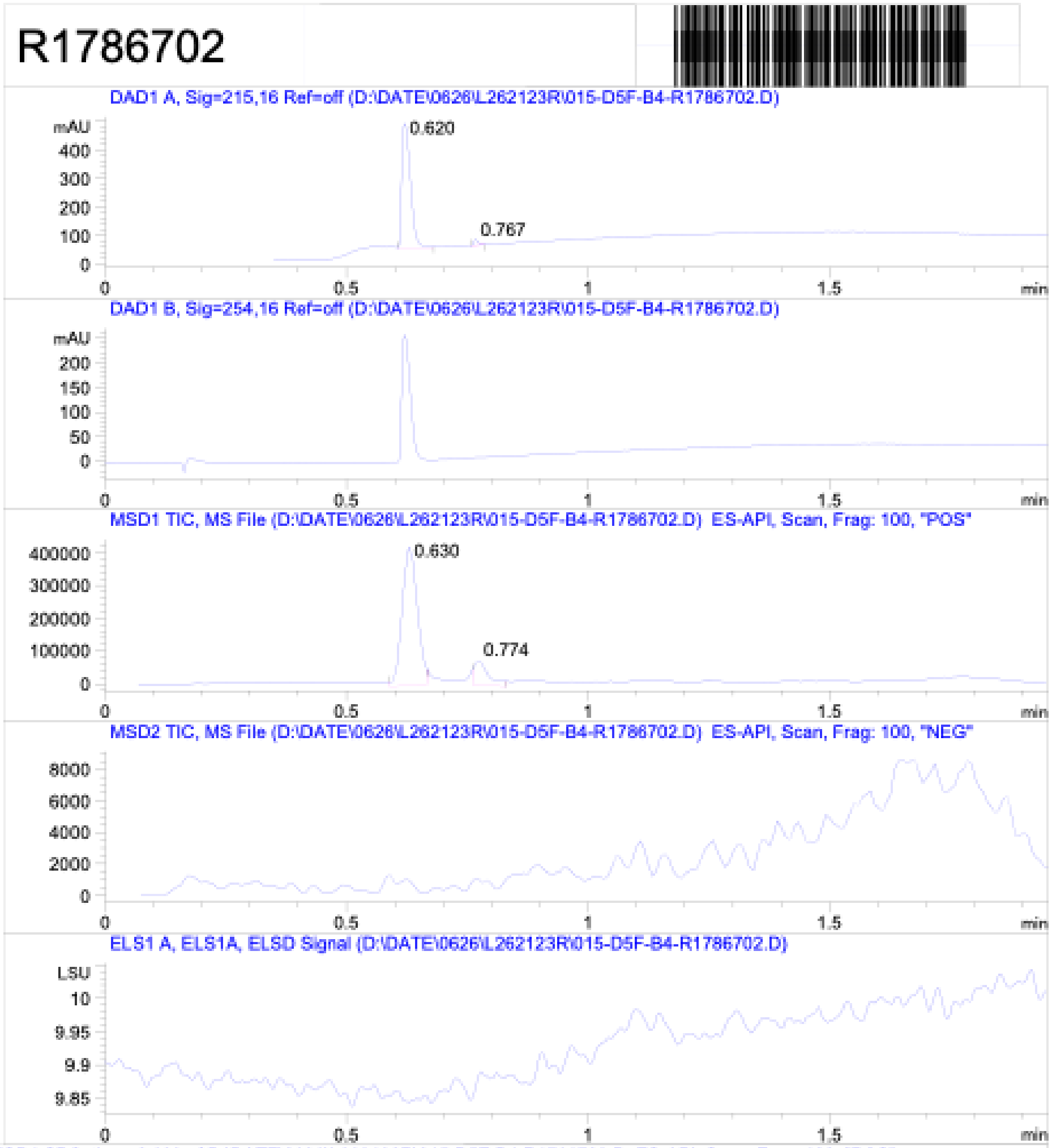

Mol Wt 213.66
Exact Mass 177.09

#	Time	Area%
1	0.620	97.81
2	0.767	2.19

RT 0.630

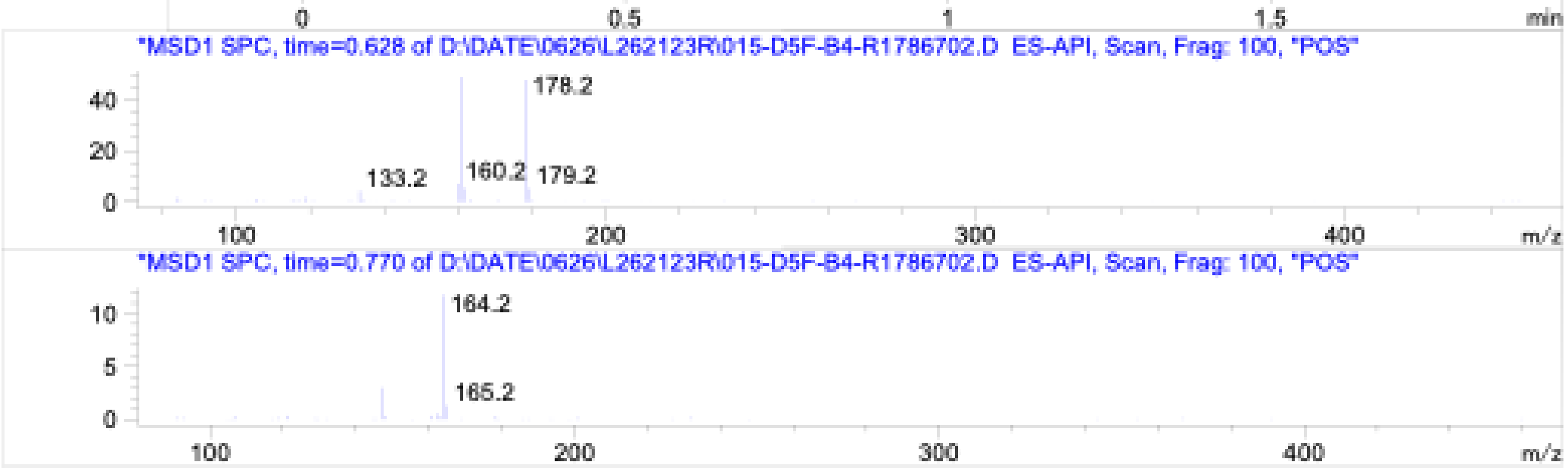

RT 0.774

Inj.Date 6/26/2020 O -15- Acq. Method C:\Chem32\-> ->

Z2739607300

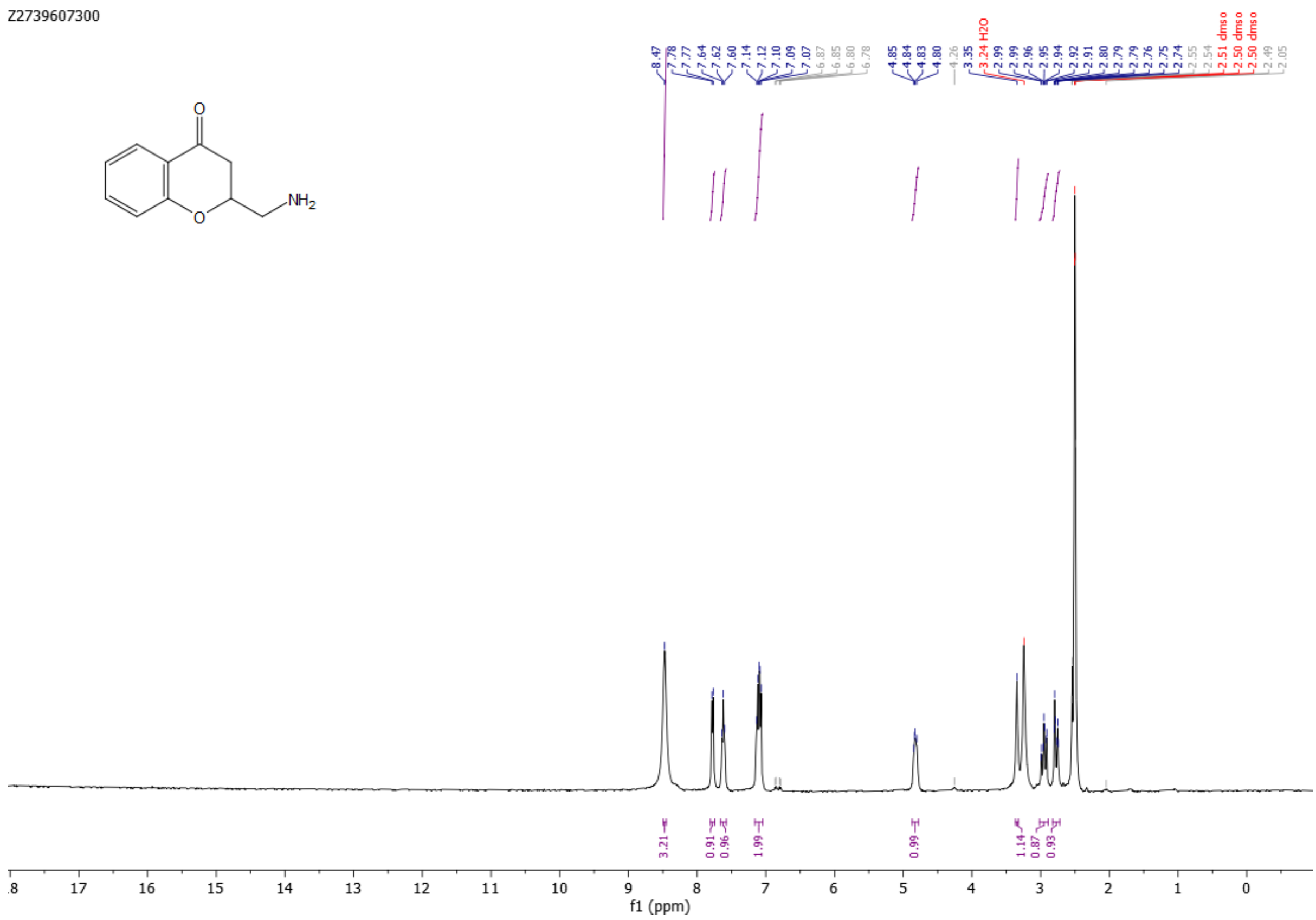

Z2739607300

MaxPeak: 100.00%
Ret_Time: 0.862 min

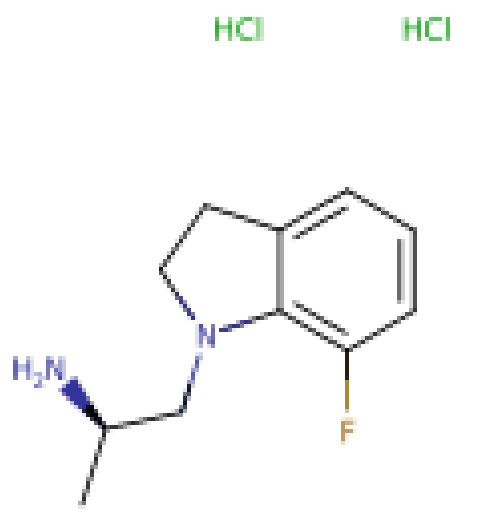

Mol Wt 267.17
Exact Mass 194.15

#	Time	Area%
1	0.862	100.00

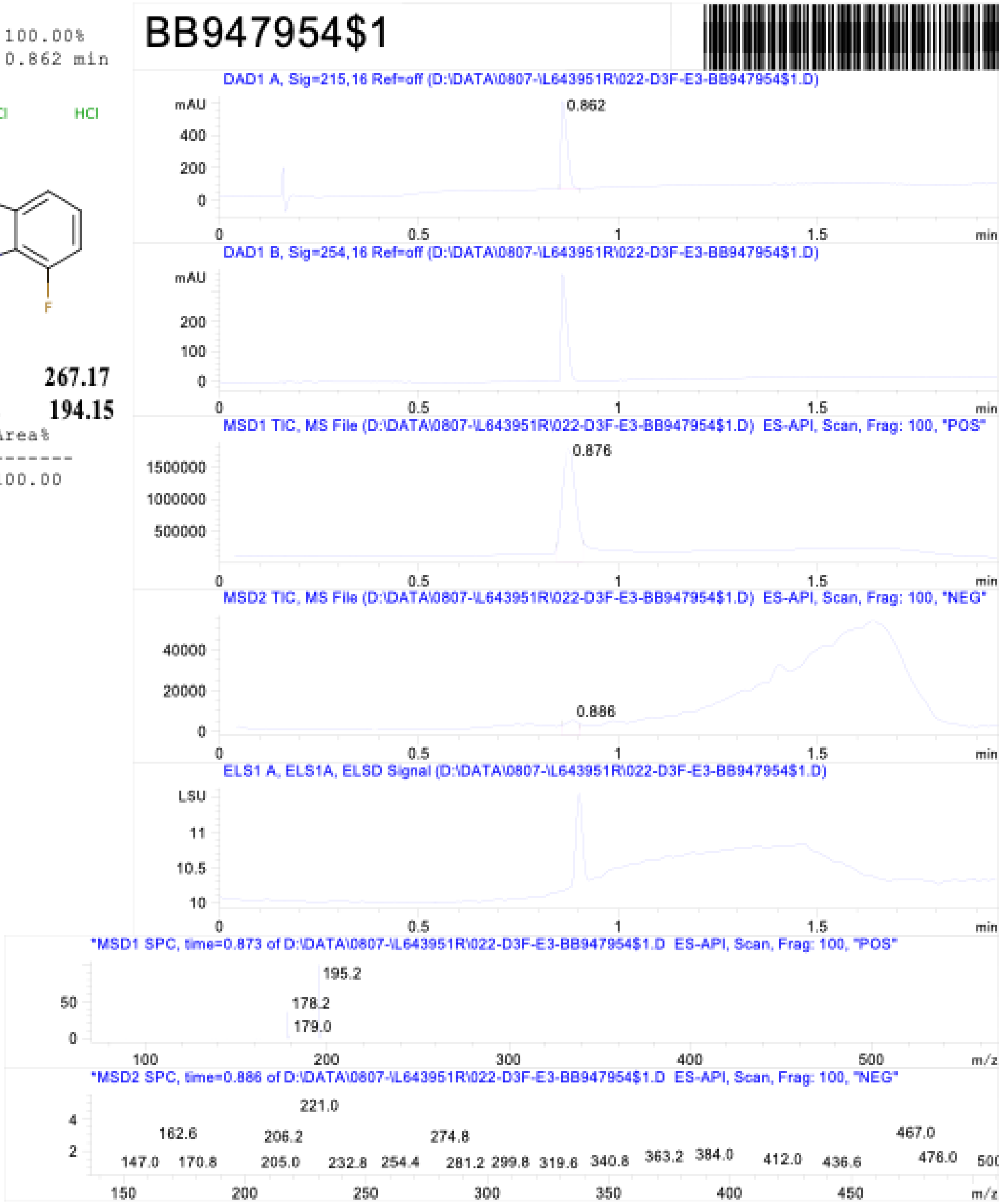

RT 0.876

RT 0.886

Inj.Date 8/7/2023 H -26- Acq. Method C:\Users\ -> ->

Z8721927756

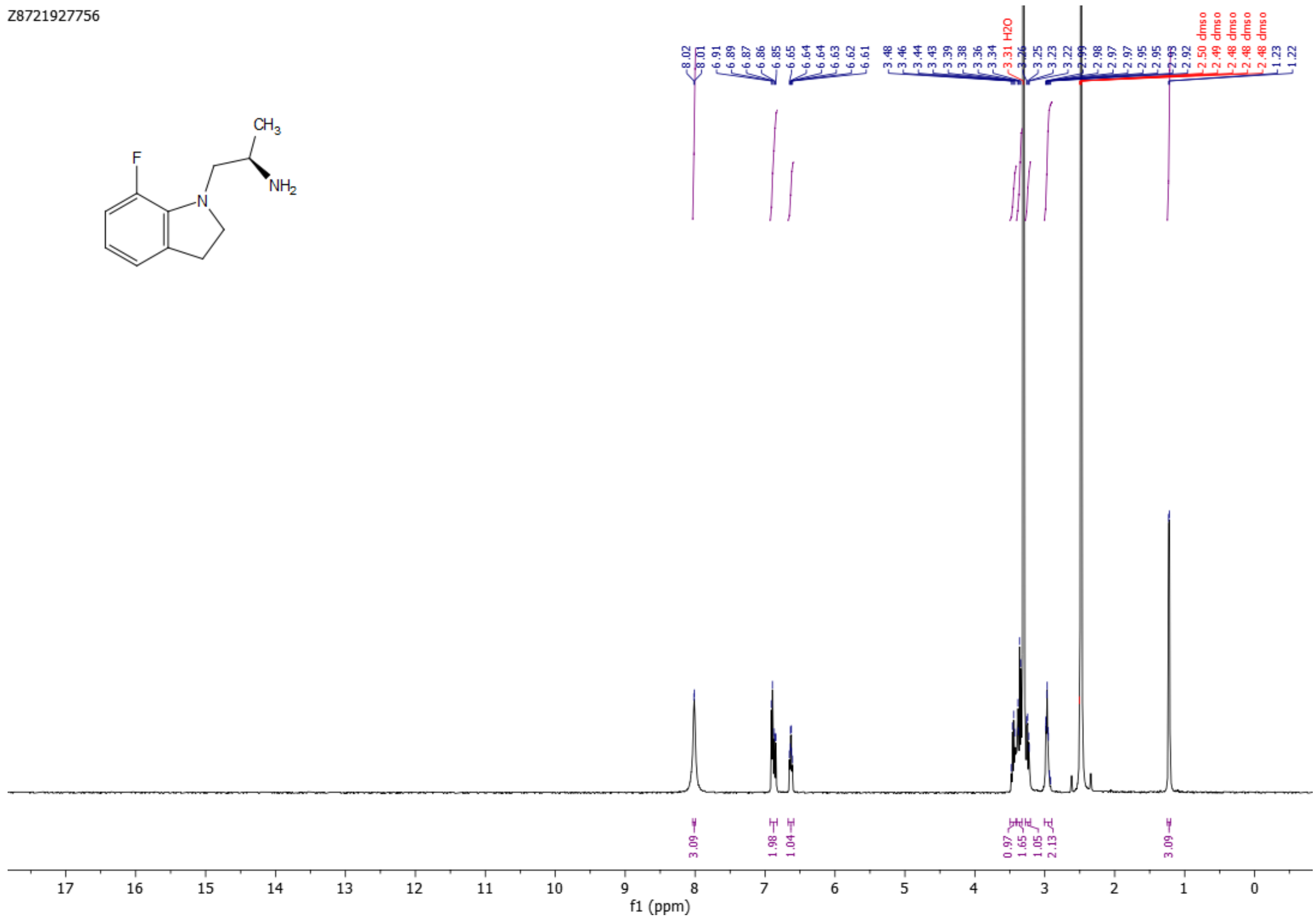

Z8721927756

```
MaxPeak:  95.42%
Ret_Time: 0.762 min
```

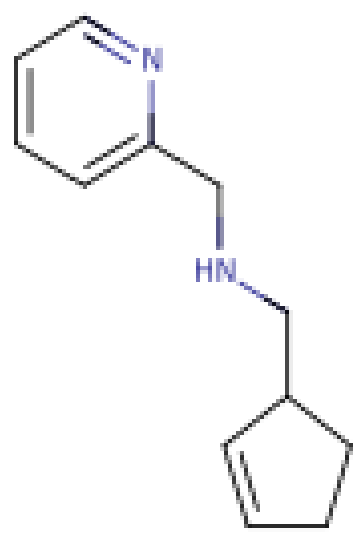

Mol Wt 188.27

Exact Mass 188.16

#	Time	Area%
1	0.713	4.58
2	0.762	95.42

RT 0.784

RT 0.787

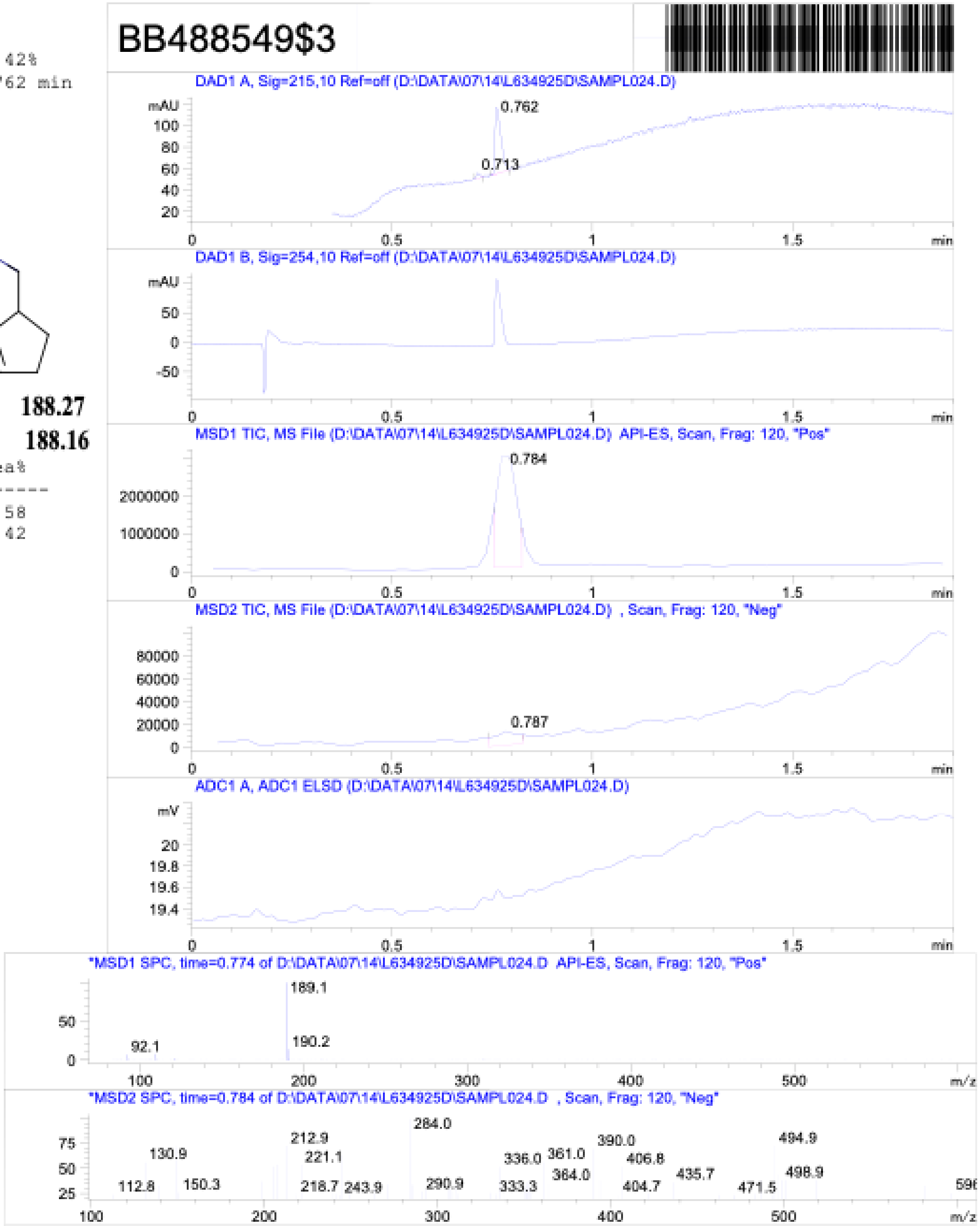


```
Inj.Date 7/14/2023          CH     P2-C-06    -SL-     Acq. Method C:\HPCHEM\->    ->
```

Z2787083864

MaxPeak: 100.00%
Ret_Time: 0.426 min

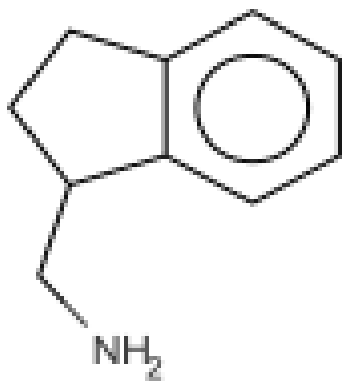

Mol Wt 147.217
Exact Mass 147.13

#	Time	Area%
1	0.426	100.00

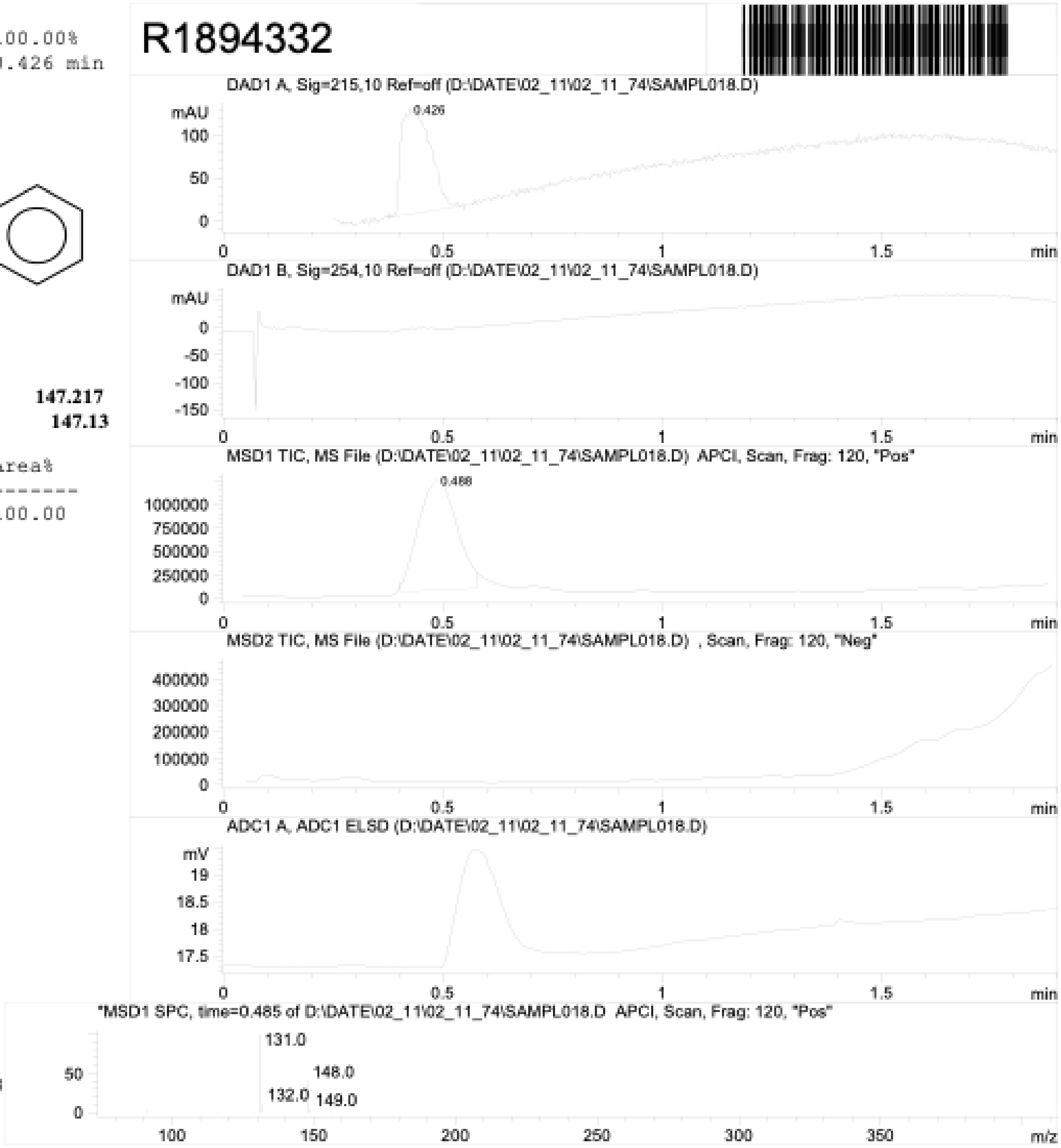

RT 0.488

Inj.Date 2/12/2011 O P2-B-06 SL Acq. Method C:\HPCHEM\-> ->

Z3241259976

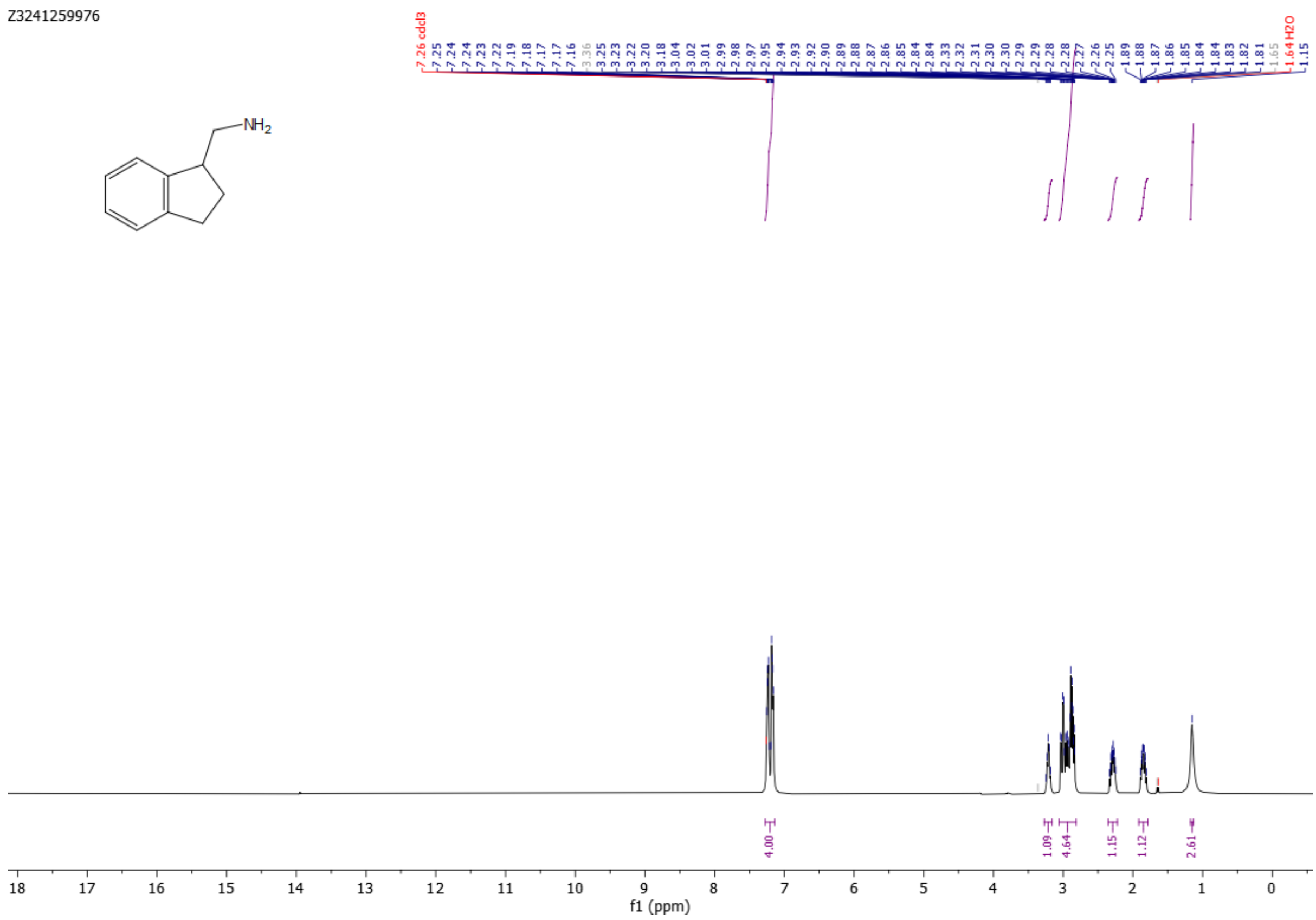

Z3241259976

MaxPeak: 100.00%
Ret_Time: 0.463 min

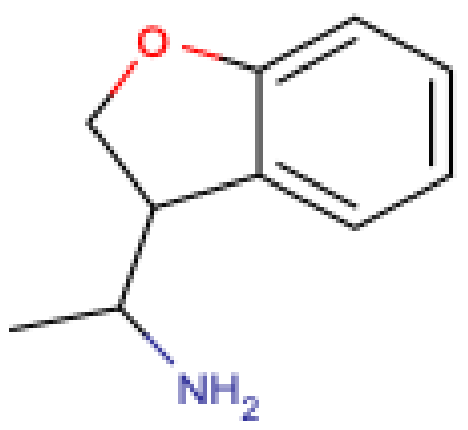

Mol Wt 163.22
Exact Mass 163.12

#	Time	Area%
1	0.463	100.00

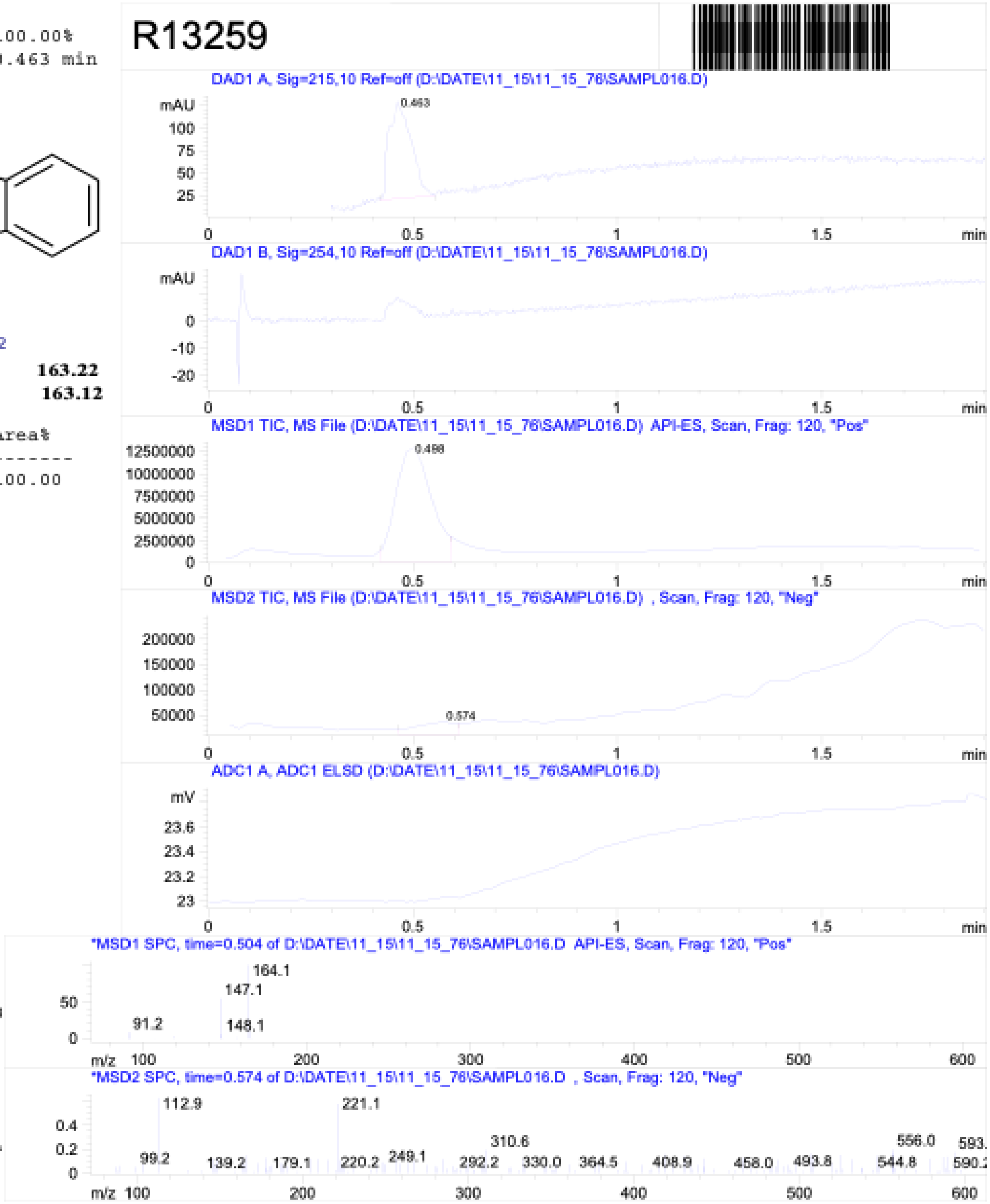

RT 0.498

RT 0.574

Inj.Date 11/15/2012 L P2-B-05 VL Acq. Method C:\HPCHEM\-> ->

Z1493816383

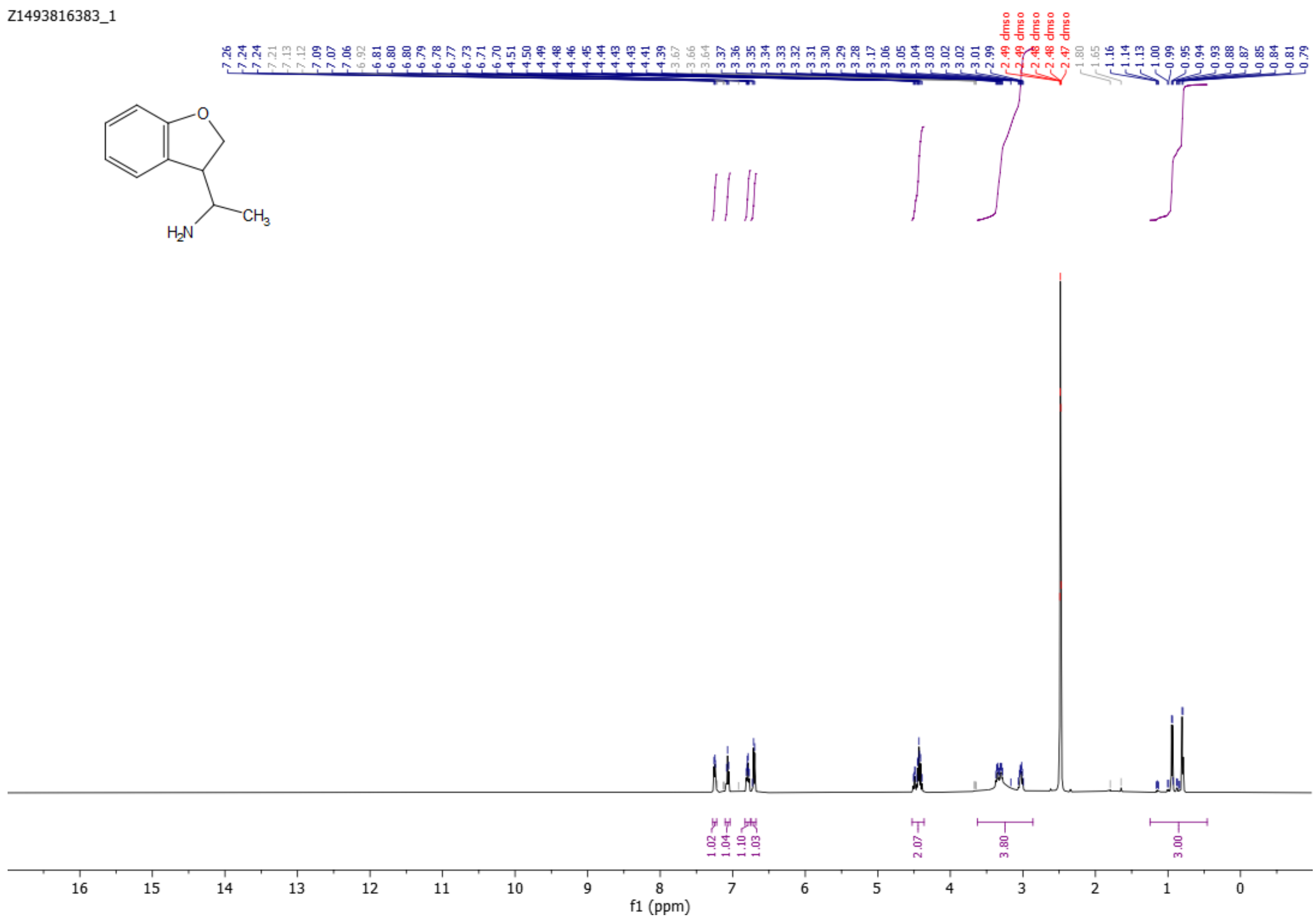

Z1493816383

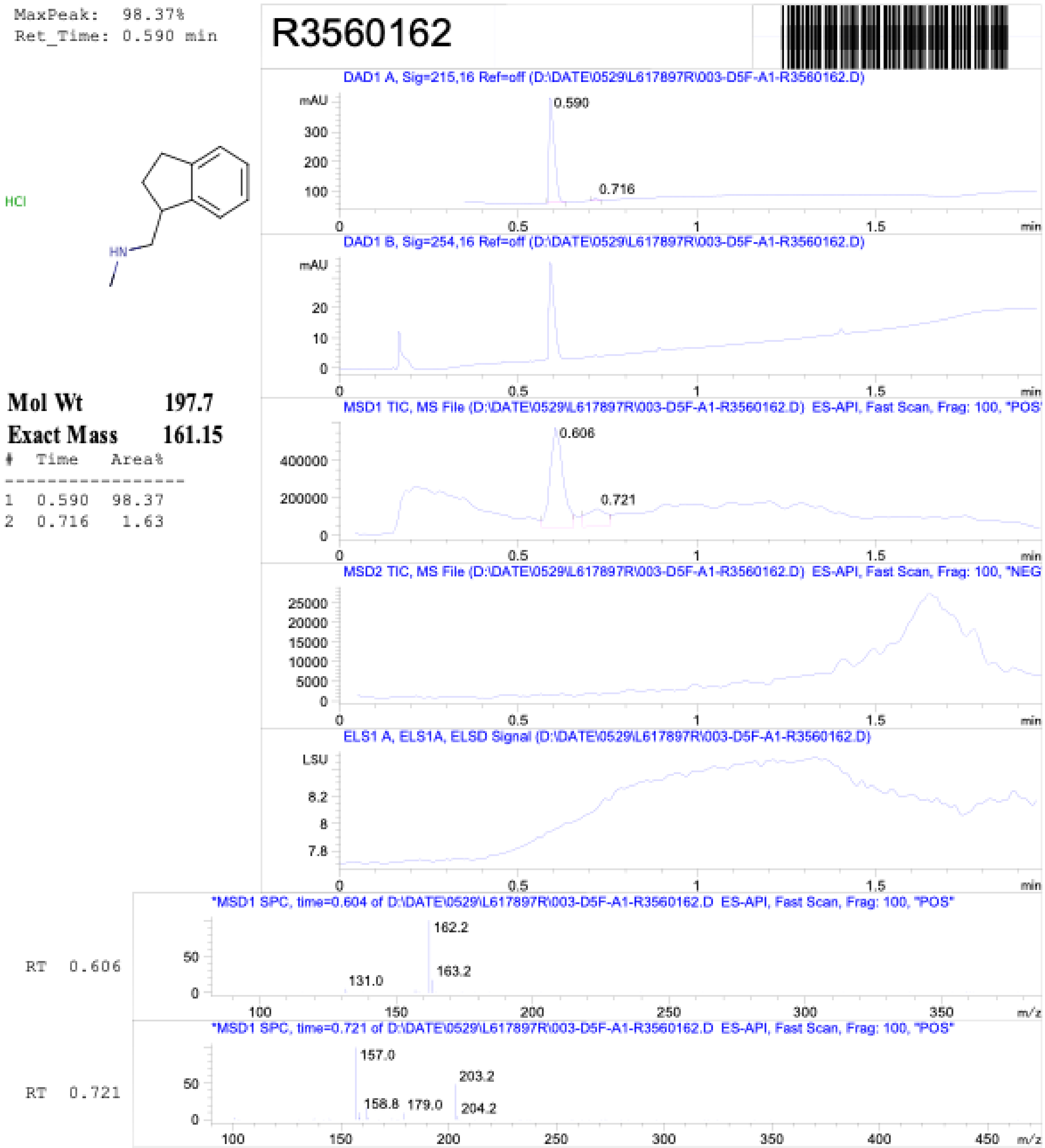

Inj.Date 5/29/2023 E Acq. Method C:\Users\ -> ->

Z1251362220

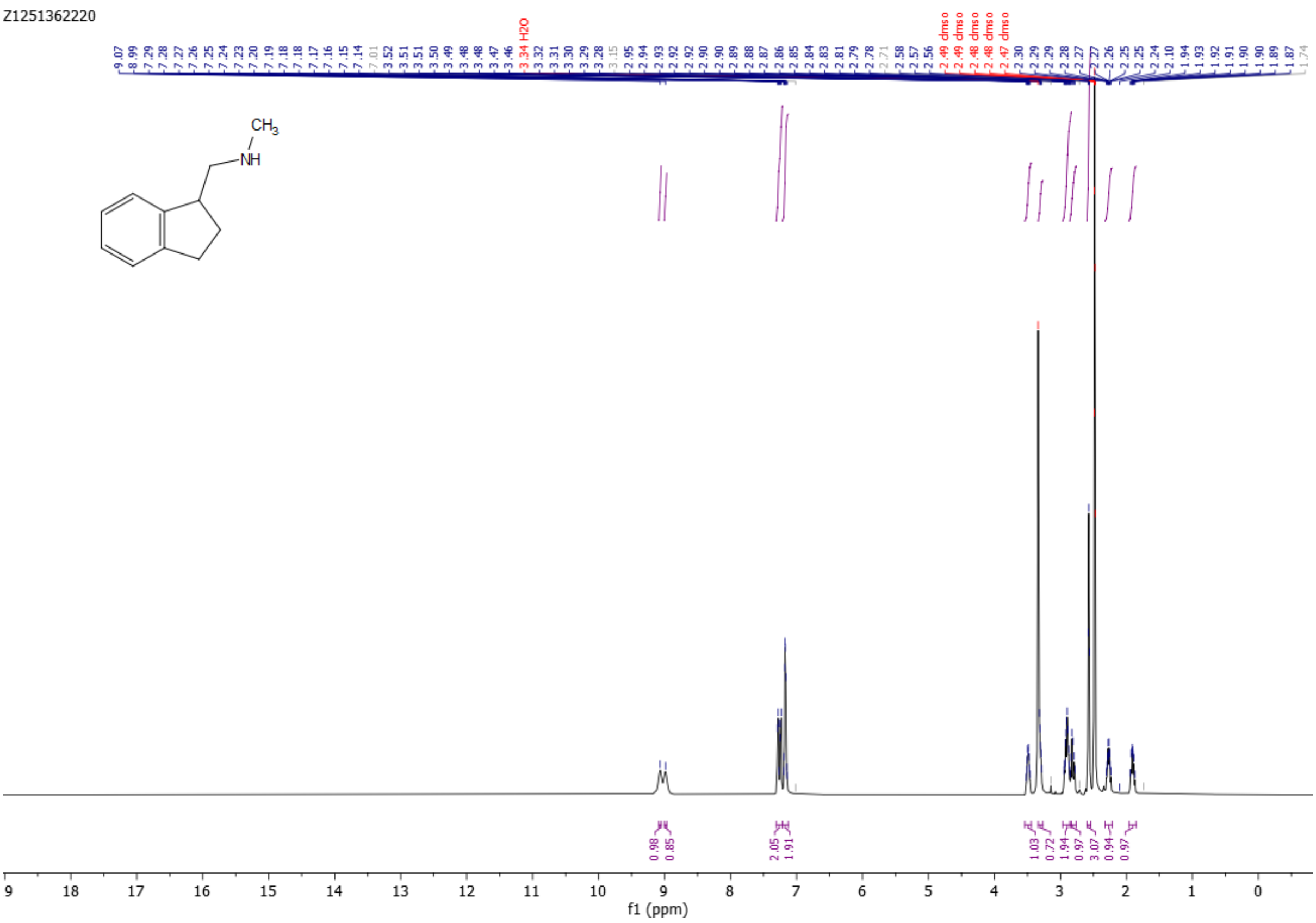

Z1251362220

MaxPeak: 97.39%
Ret_Time: 0.515 min

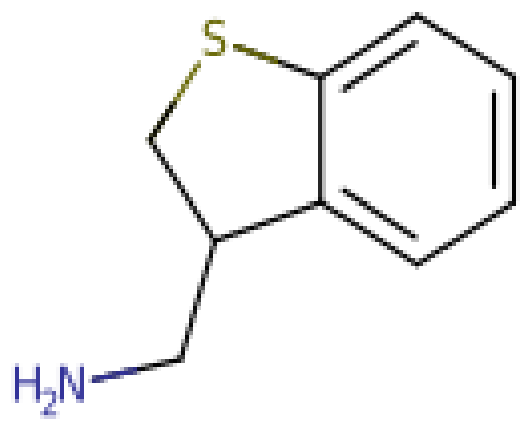

Mol Wt 165.26

Exact Mass 165.08

#	Time	Area%
1	0.515	97.39
2	1.574	1.23
3	2.316	1.38

RT 0.540

RT 2.324

RT 0.561

RT 2.328

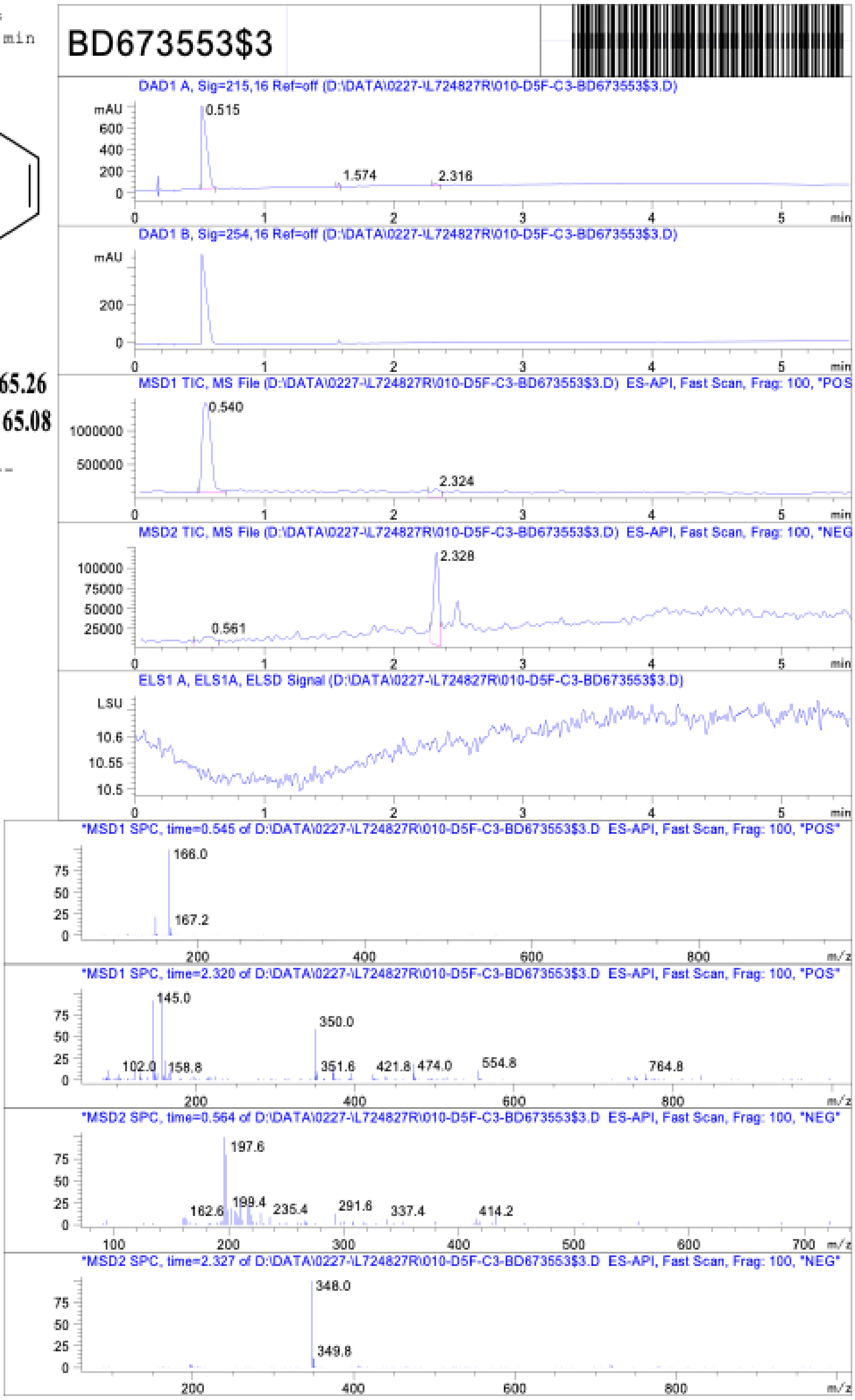

Inj.Date 2/27/2024 LB

C:\Users\Public\Documents\ChemStation\1\Data\02_27\L724827R\UZRX45_3-5V.M

Z1316760731

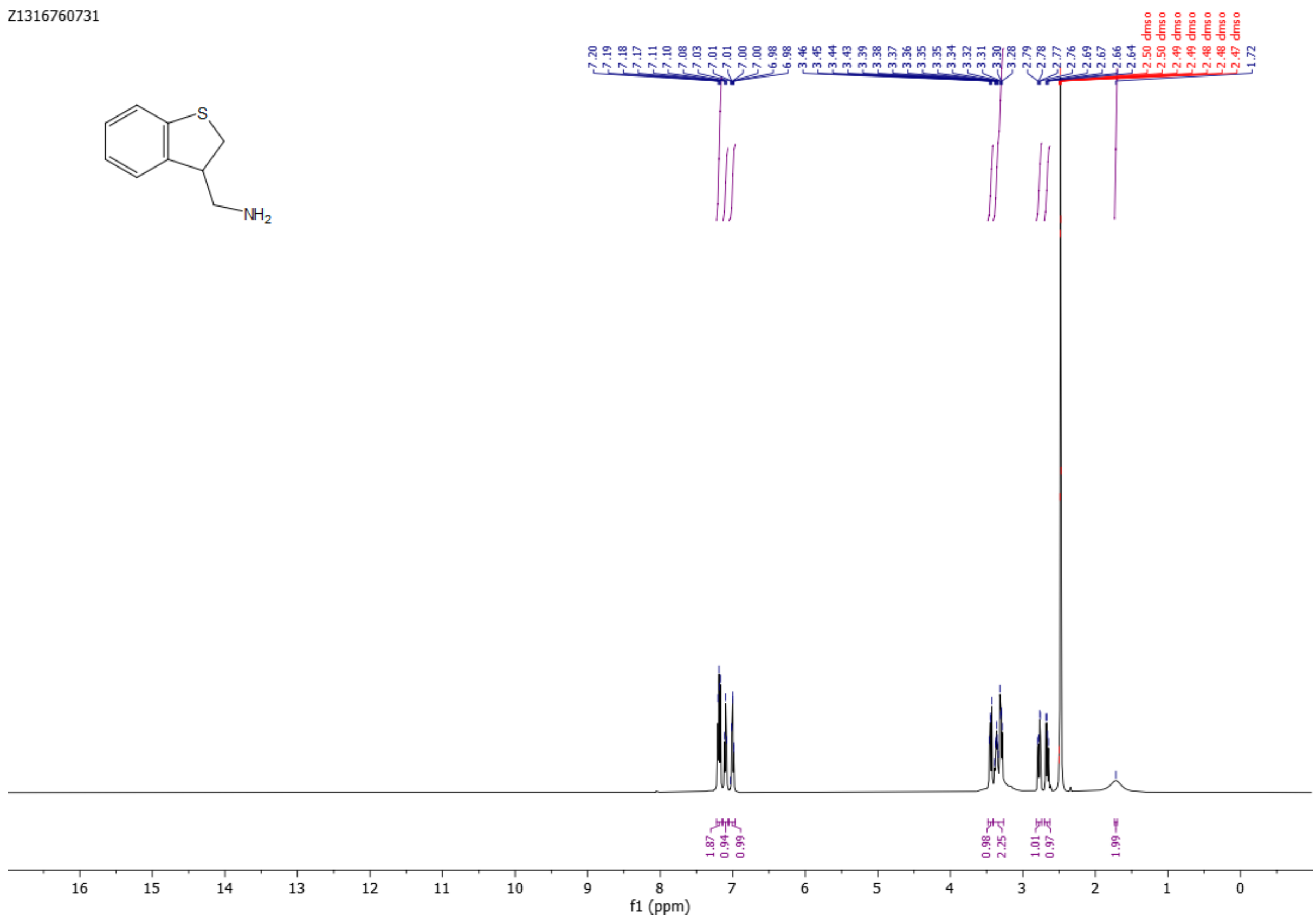

Z1316760731

MaxPeak: 100.00%
Ret_Time: 0.137 min

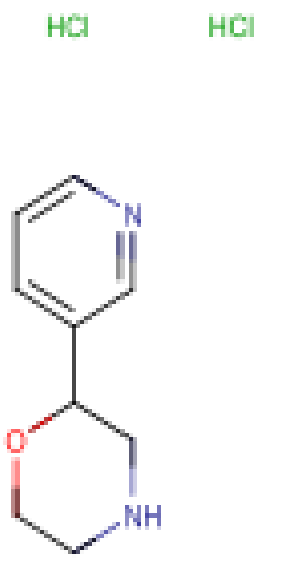

Mol Wt 237.13
Exact Mass 164.11

#	Time	Area%
1	0.137	100.00

RT 0.153

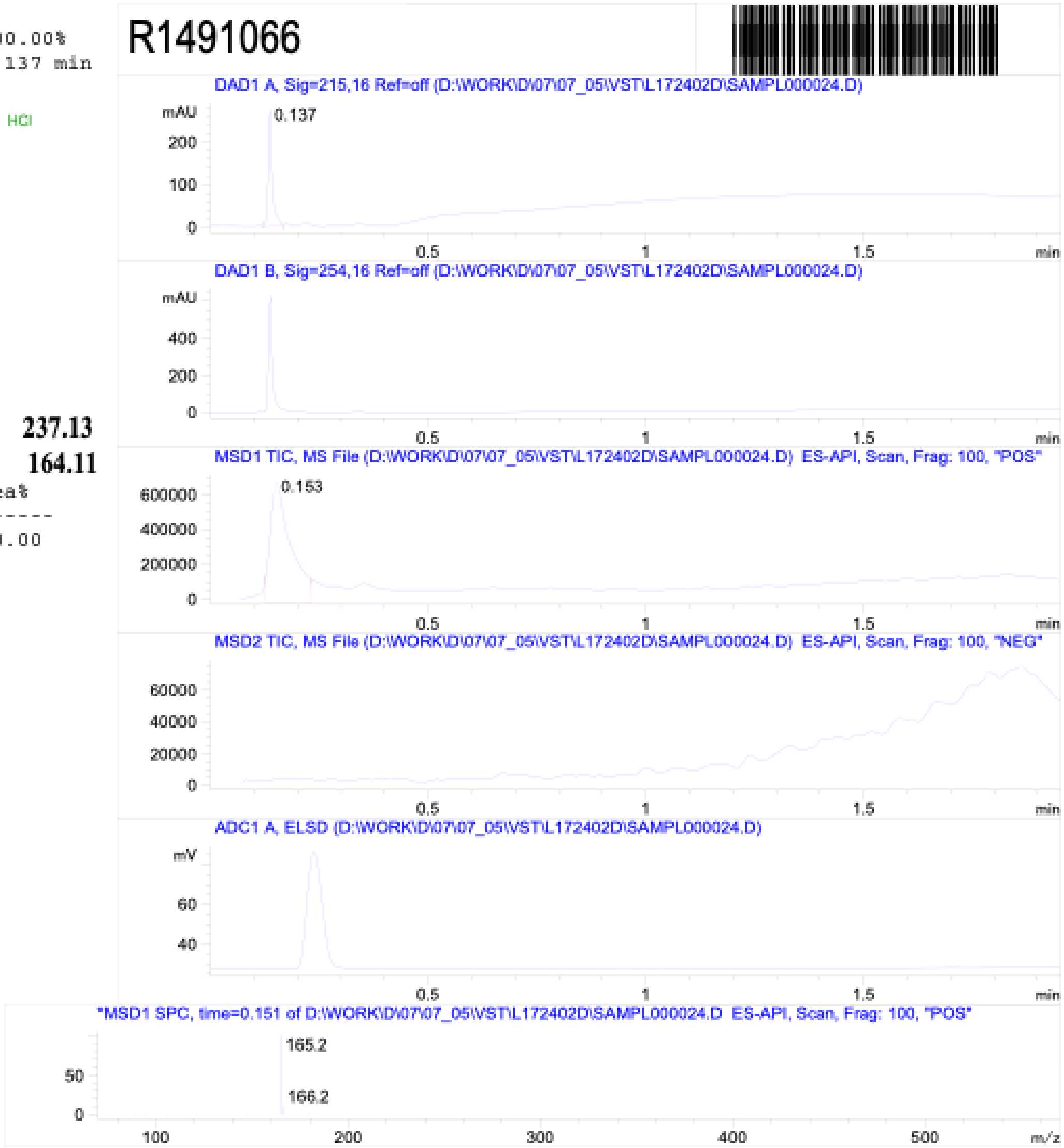

Inj.Date 7/5/2019 K P1-A-04 -6- Acq. Method C:\CHEM32\-> ->

805

806 Z3235143301

807

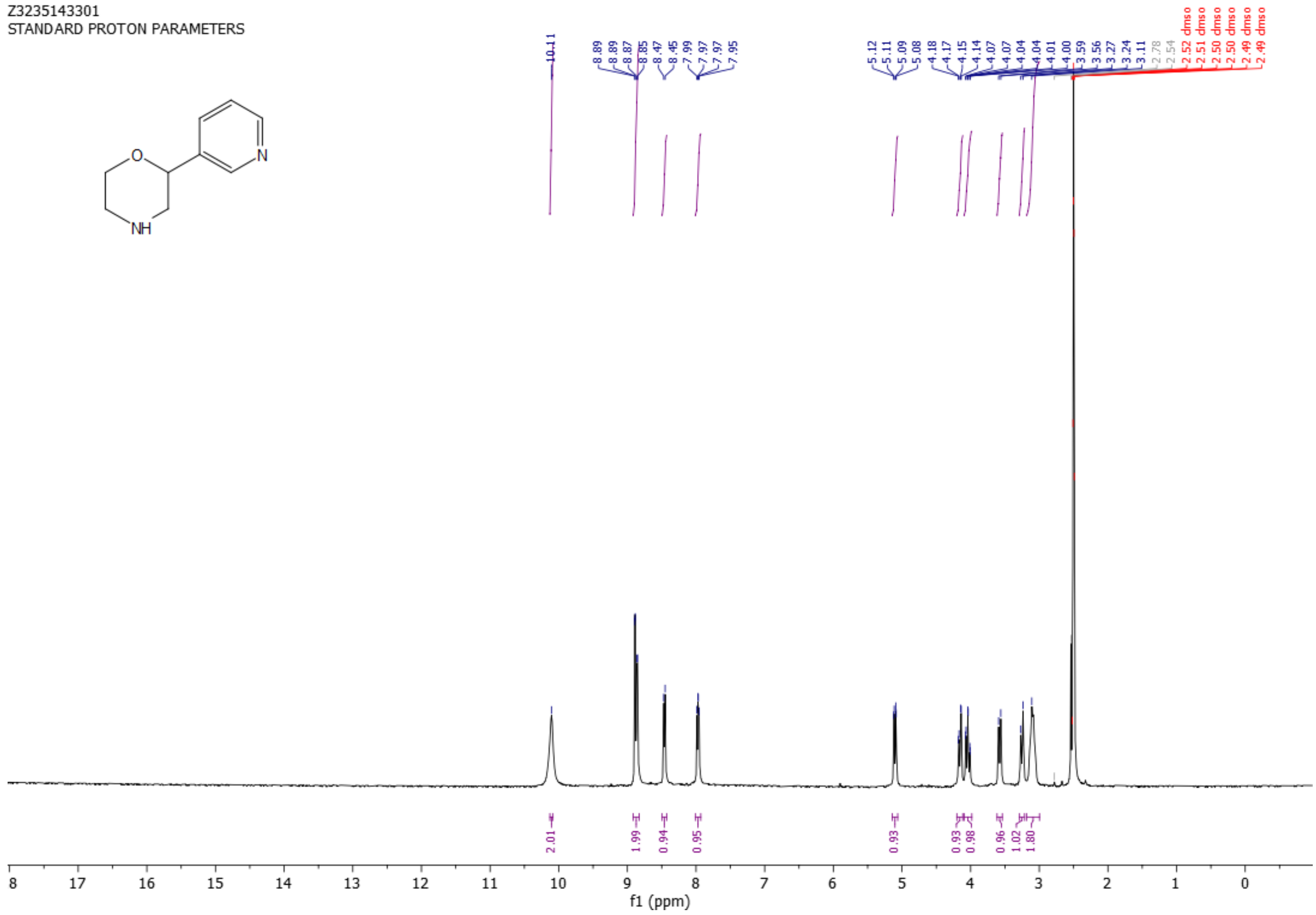

Z3235143301

MaxPeak: 97.89%
Ret_Time: 0.495 min

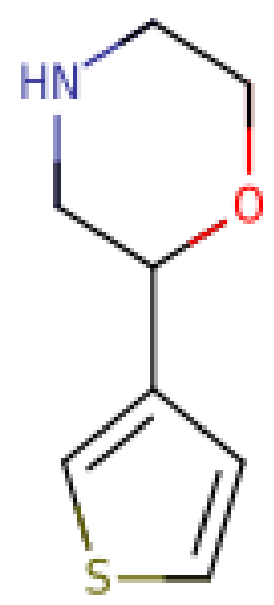

Mol Wt 169.24
Exact Mass 169.07

#	Time	Area%
1	0.495	97.89
2	0.968	2.11

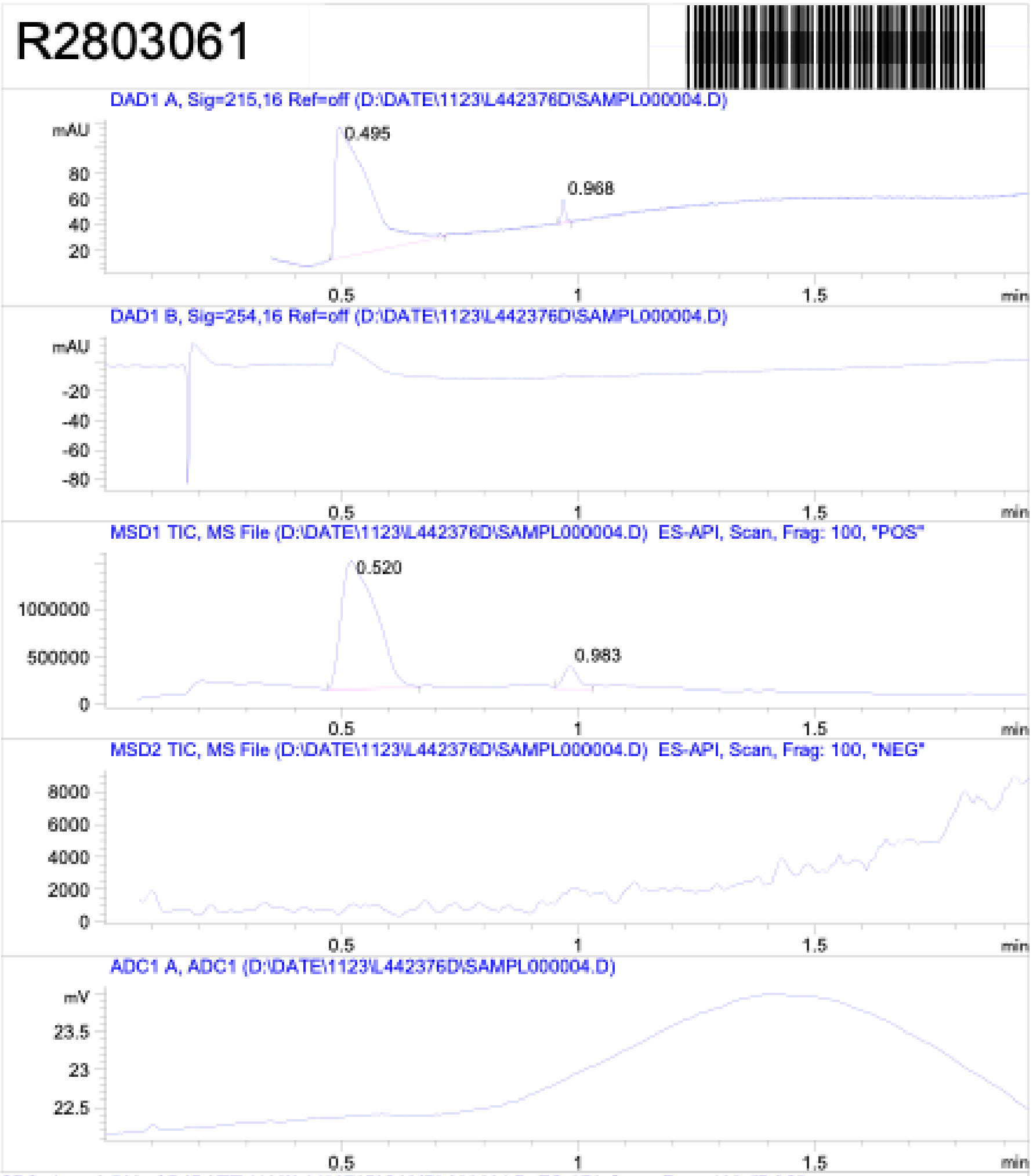

RT 0.520

RT 0.983

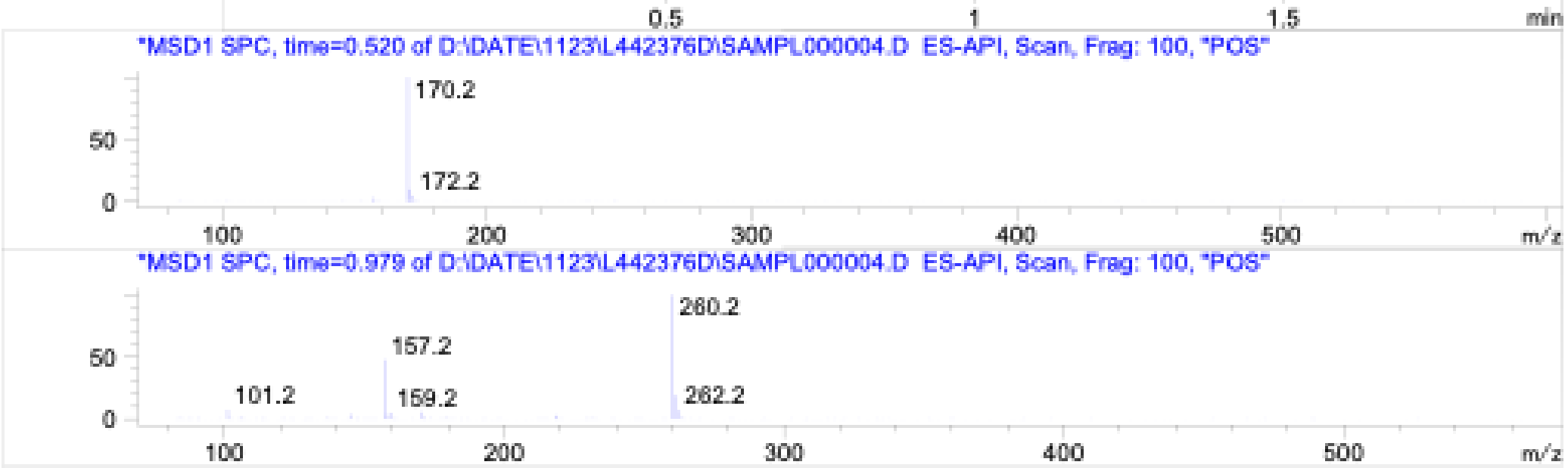

Inj.Date 11/22/2021 E - 4 - Acq. Method C:\CHEM32\-> ->

Z954552190

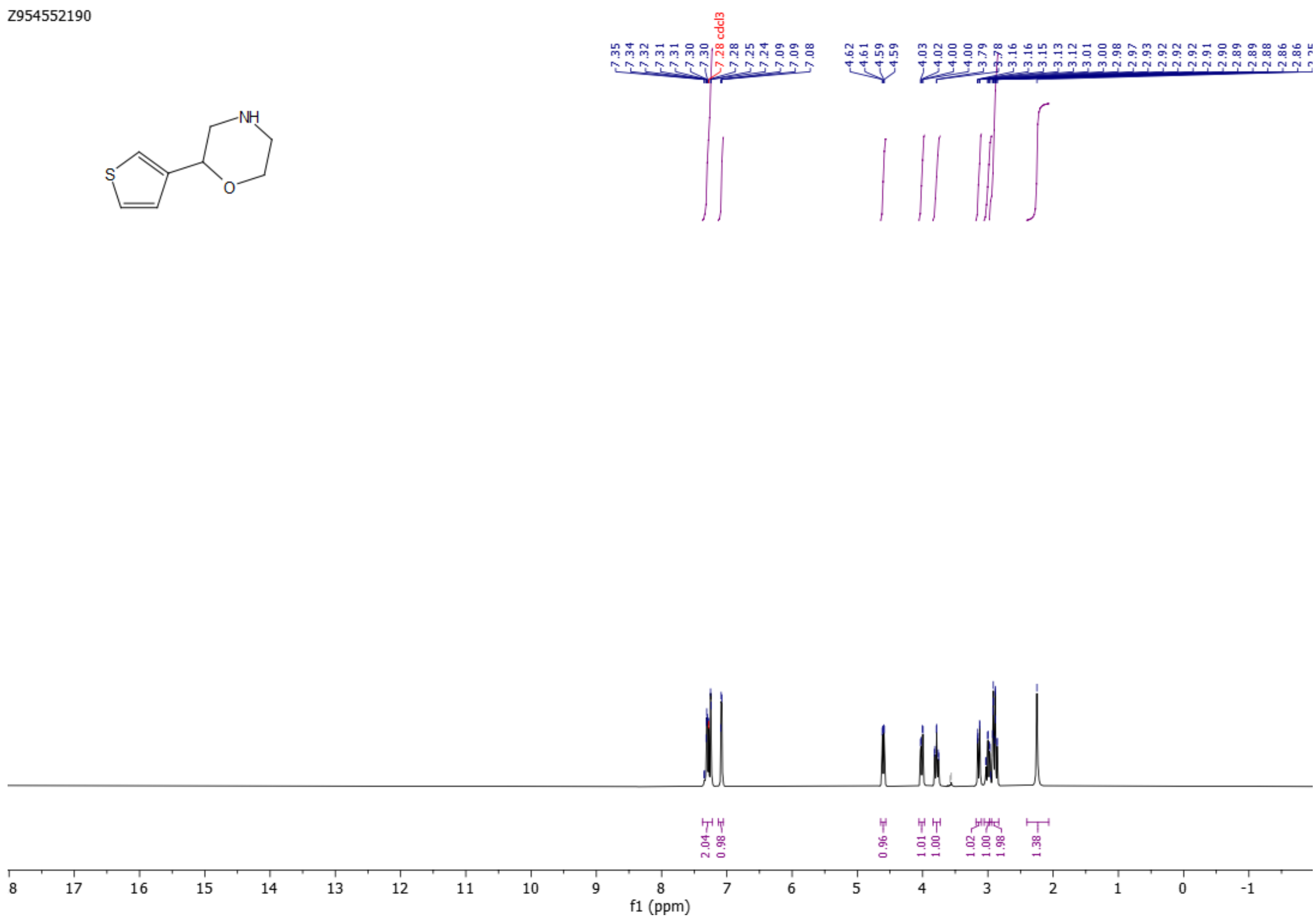

Z954552190

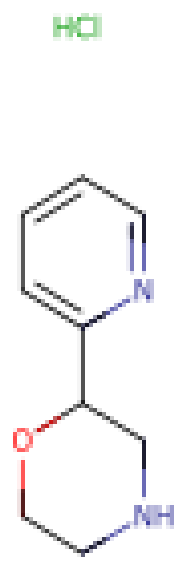

Mol Wt 237.13
Exact Mass 164.11

Error: Peaks not found!

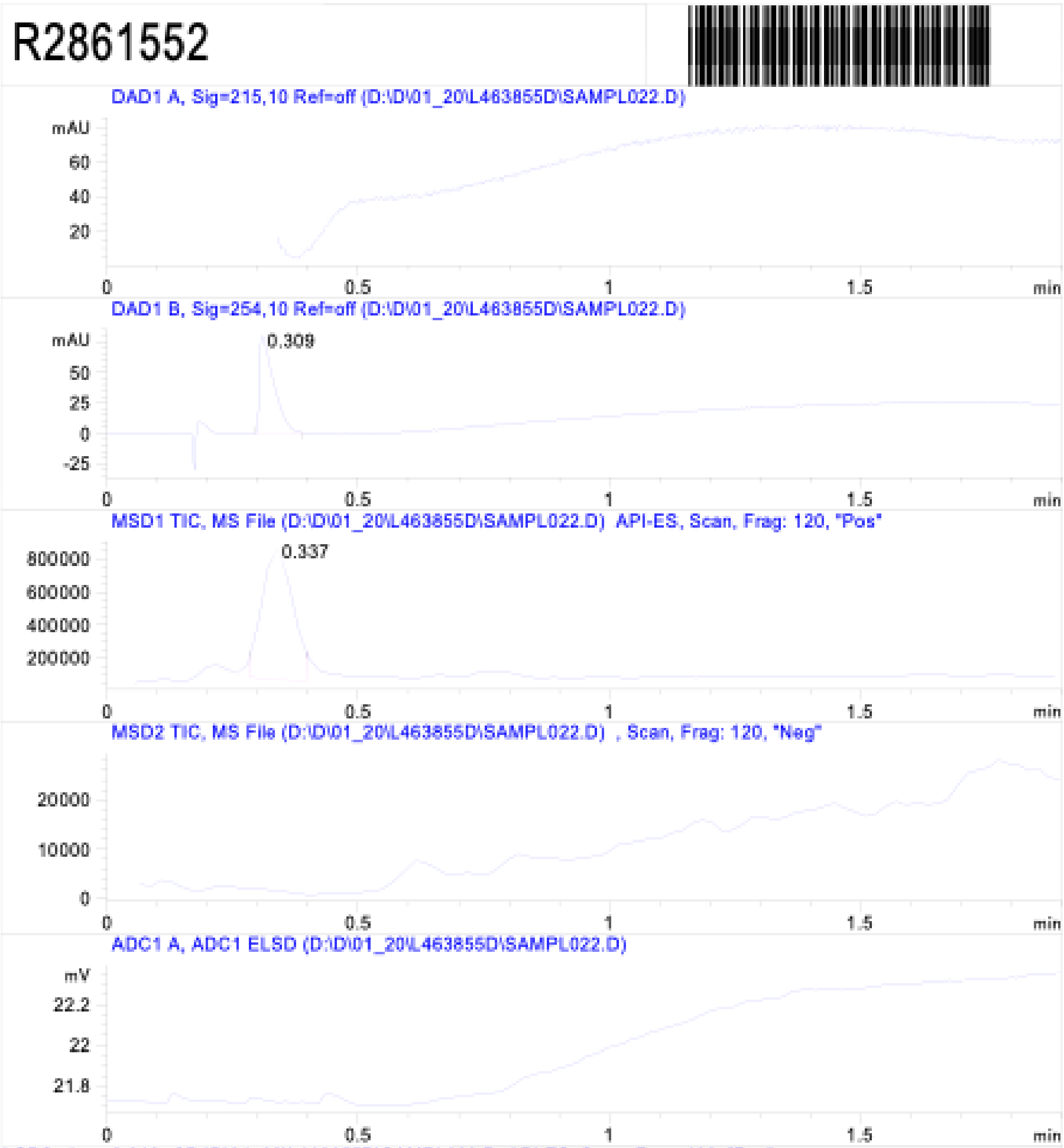

#	Time	Area%
--- DAD1 B ---		
1	0.309	100.00

RT 0.337

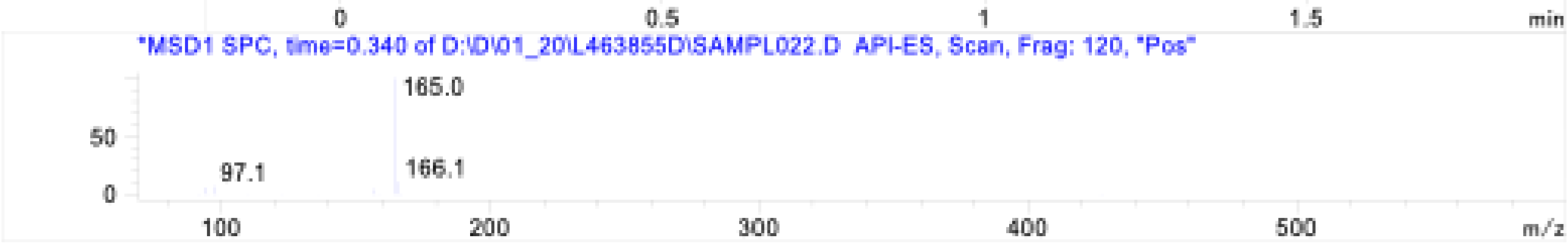

Inj.Date 1/20/2022 Y P2-C-02 -VL- Acq. Method C:\HPCHEM\-> ->

Z3235143299

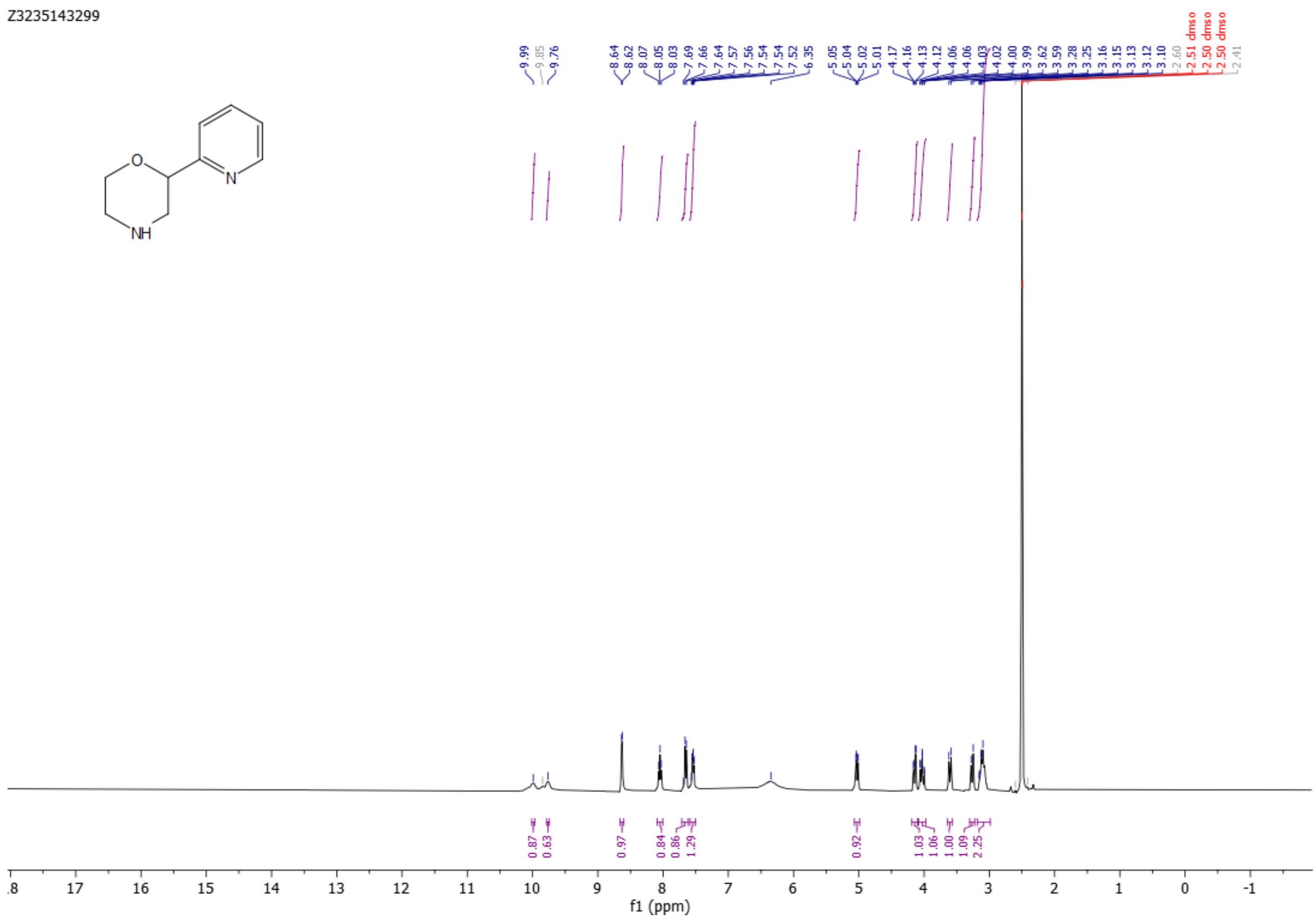

Z3235143299

MaxPeak: 36.90%
Ret_Time: 0.731 min

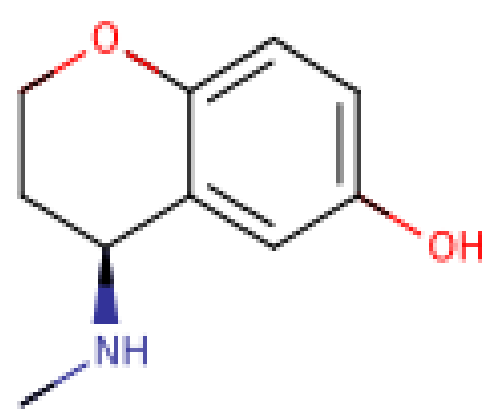

Mol Wt 179.22

Exact Mass 179.11

#	Time	Area%
1	0.604	5.66
2	0.622	18.23
3	0.654	14.14
4	0.706	15.04
5	0.731	36.90
6	0.961	10.03

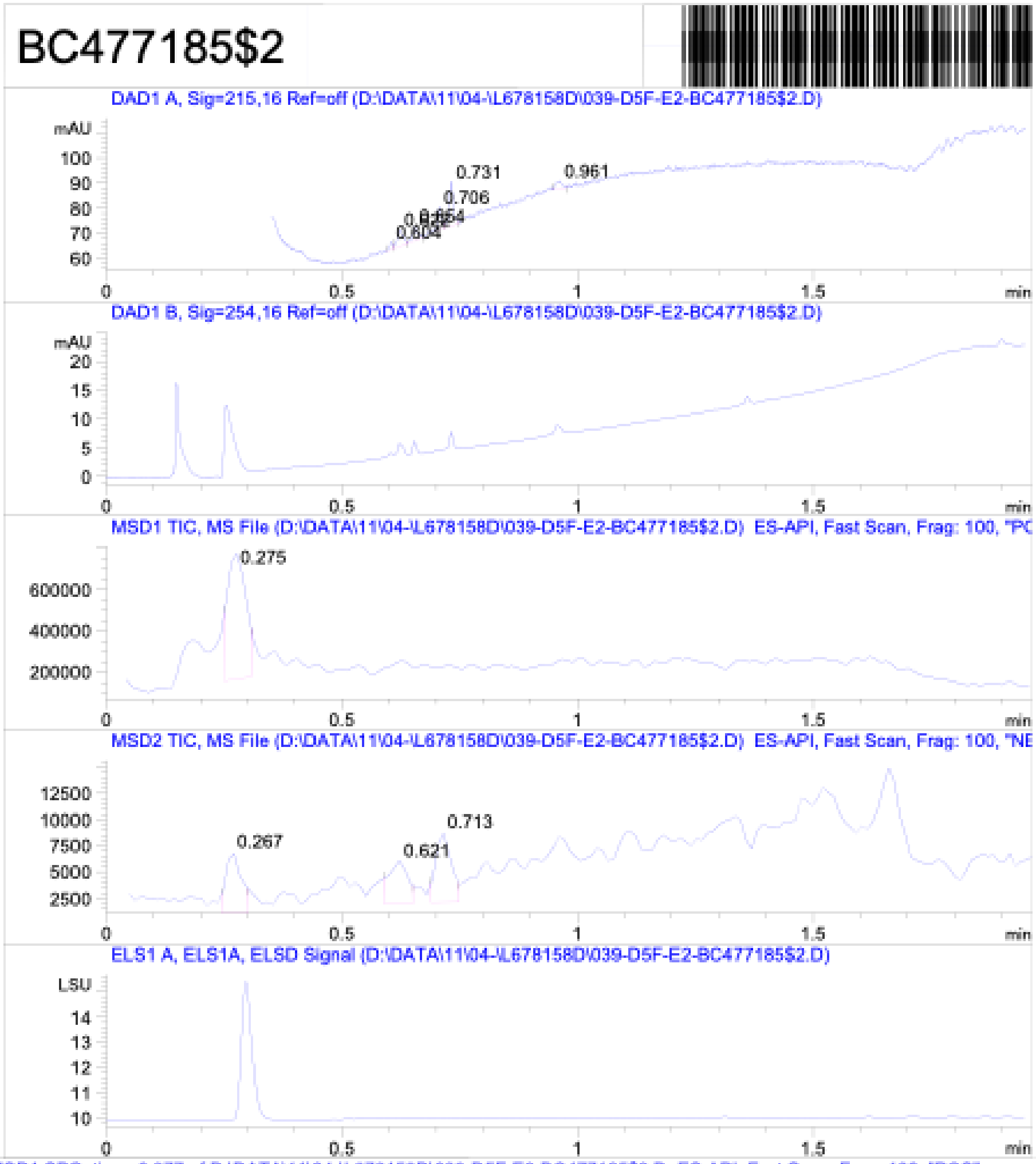

RT 0.275

RT 0.267

RT 0.621

RT 0.713

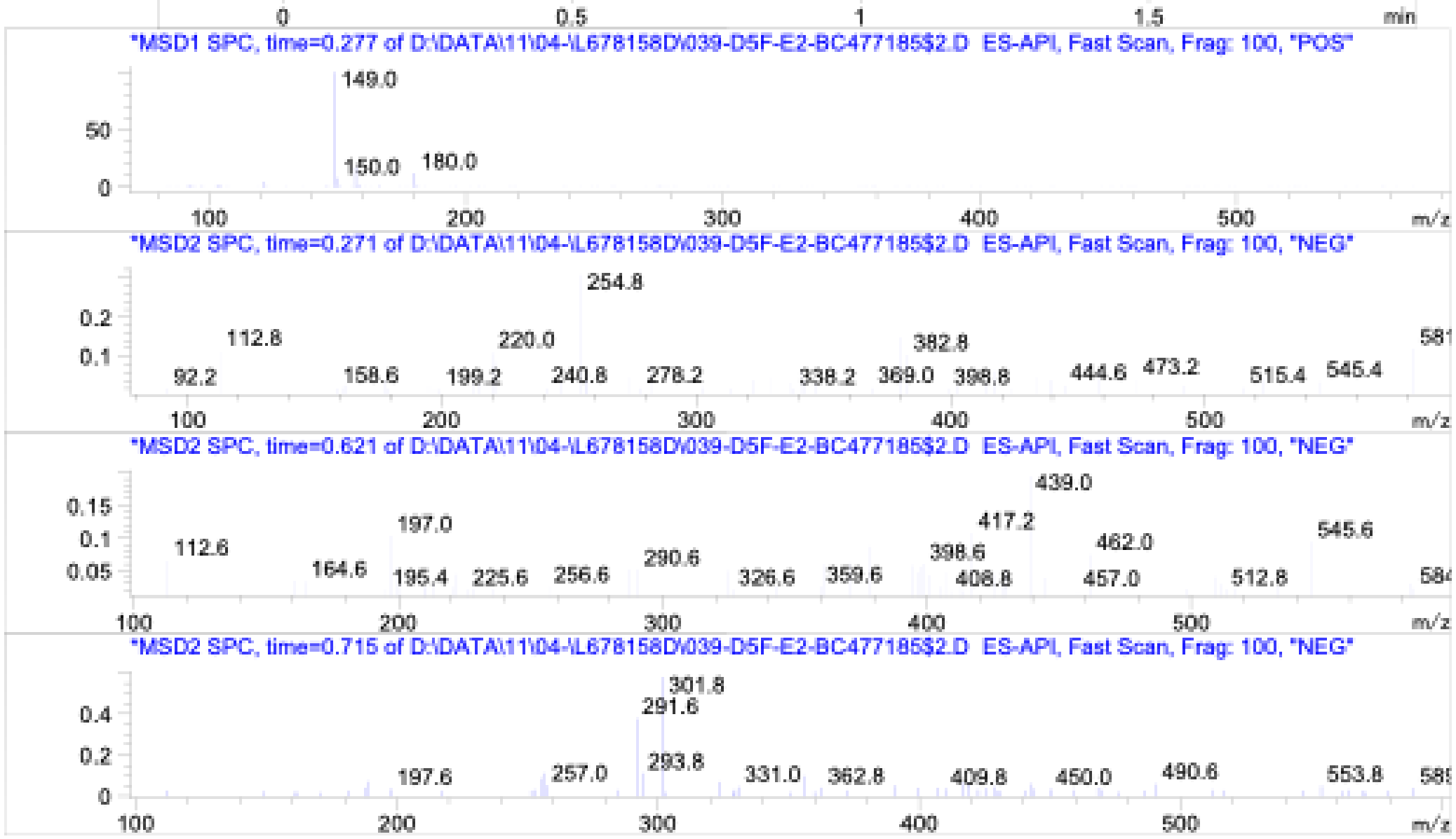

Inj.Date 11/4/2023 CH <invalid> Acq. Method C:\Users\ -> ->

Z3519067312

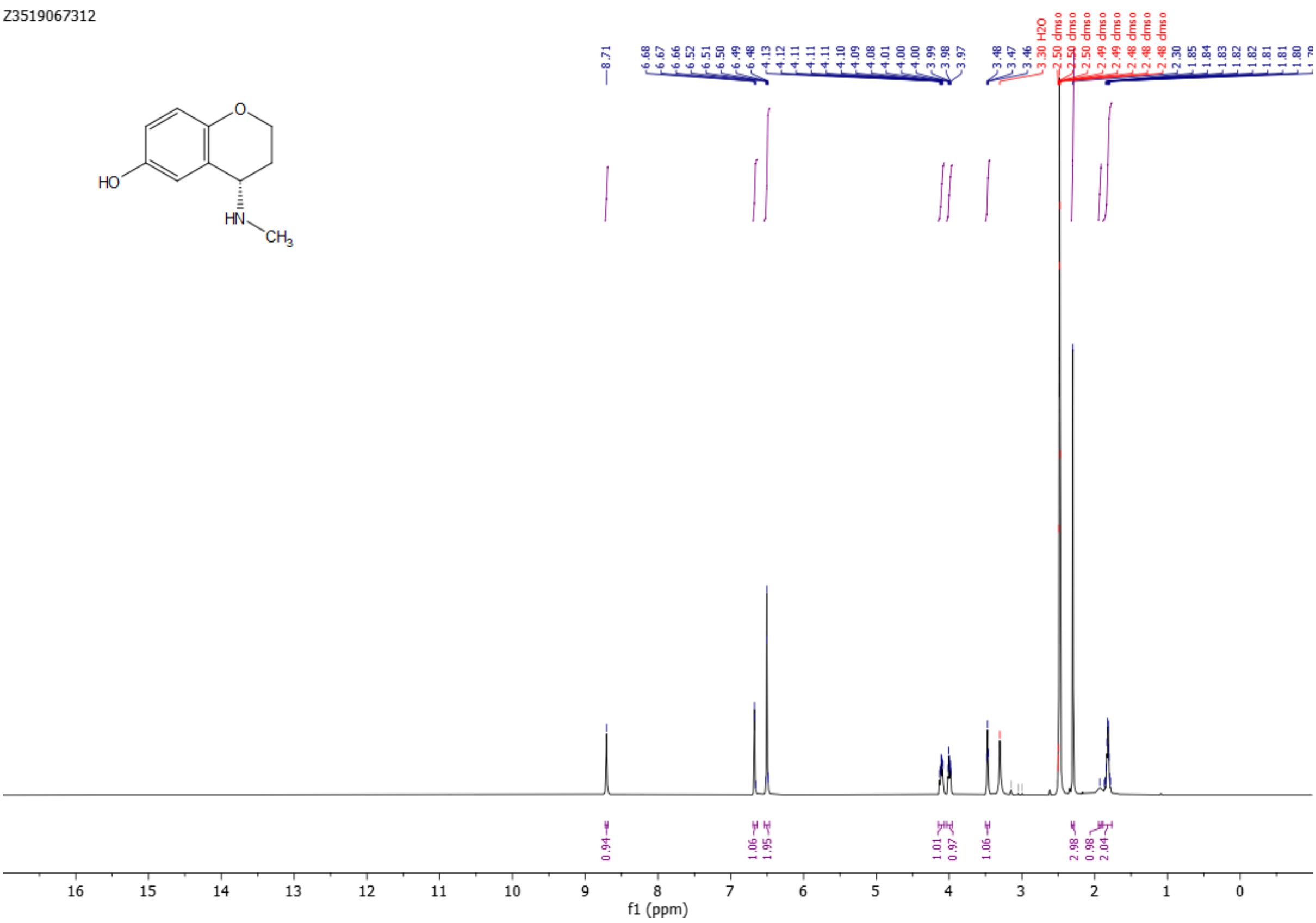

Z3519067312

MaxPeak: 100.00%
Ret_Time: 0.406 min

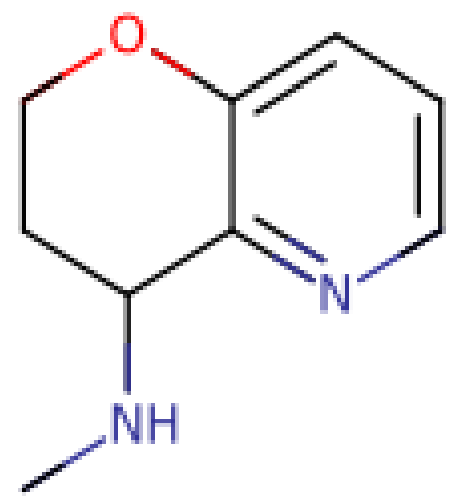

Mol Wt 164.2
Exact Mass 164.11

#	Time	Area%
1	0.406	100.00

RT 0.422

RT 0.437

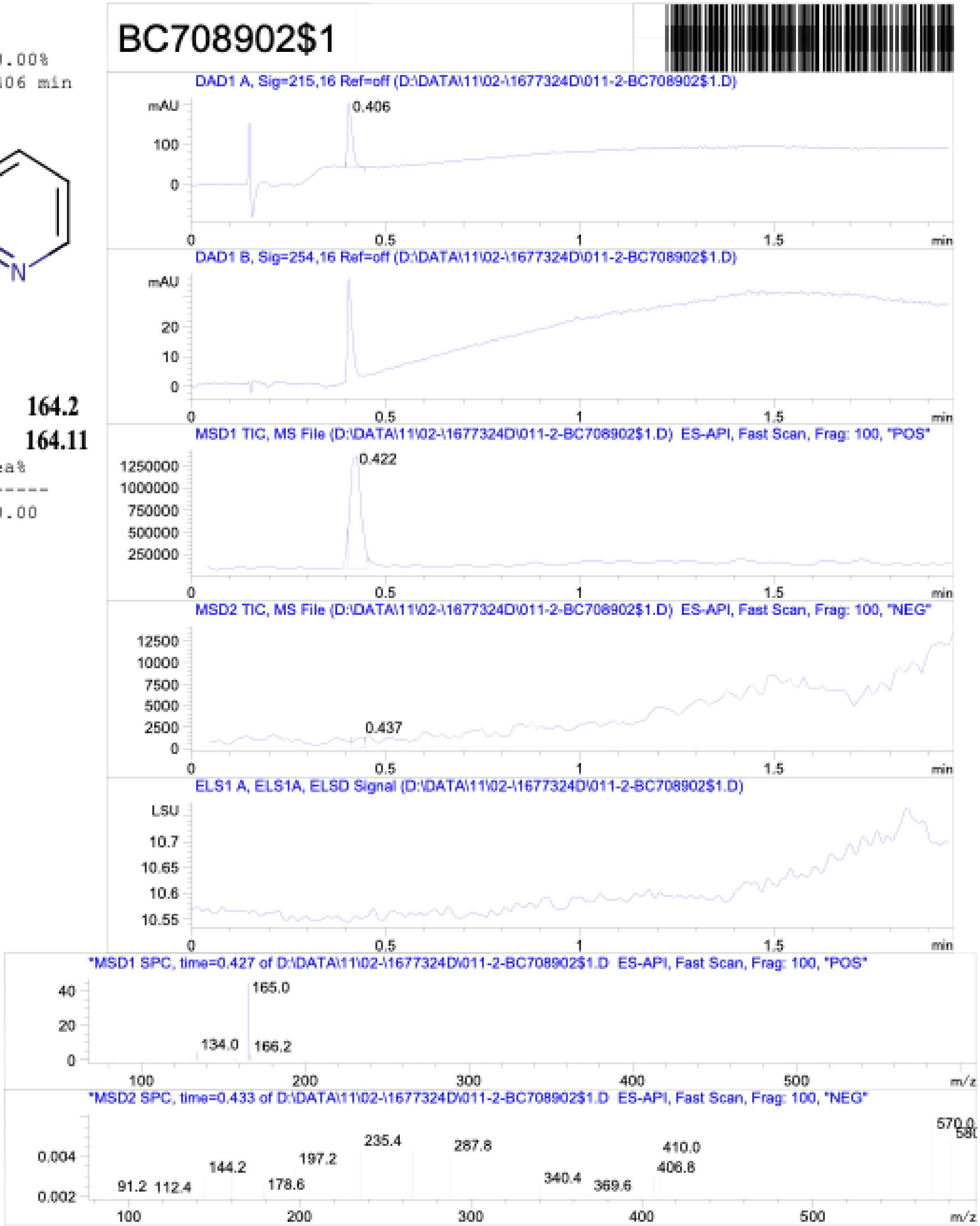

Inj.Date 11/2/2023 CH Vial 2 21 Acq. Method C:\Users\ -> ->

Z1262608488

Library Search Report

```
Data File : C:\HPCHEM\1\DATA\01_16\R1100772.D          Vial: 12
Acq On    : 16 Jan 2018   17:41                   Operator:
Sample    : R1100772                              Inst     : GC/MS Ins
Misc      : CH3CN                                 Multiplr: 1.00
                                             Sample Amount: 0.00
MS Integration Params: autoint1.e
Method    : C:\HPCHEM\1\METHODS\UNIVERS.M (Chemstation Integrator)
Title     :
```

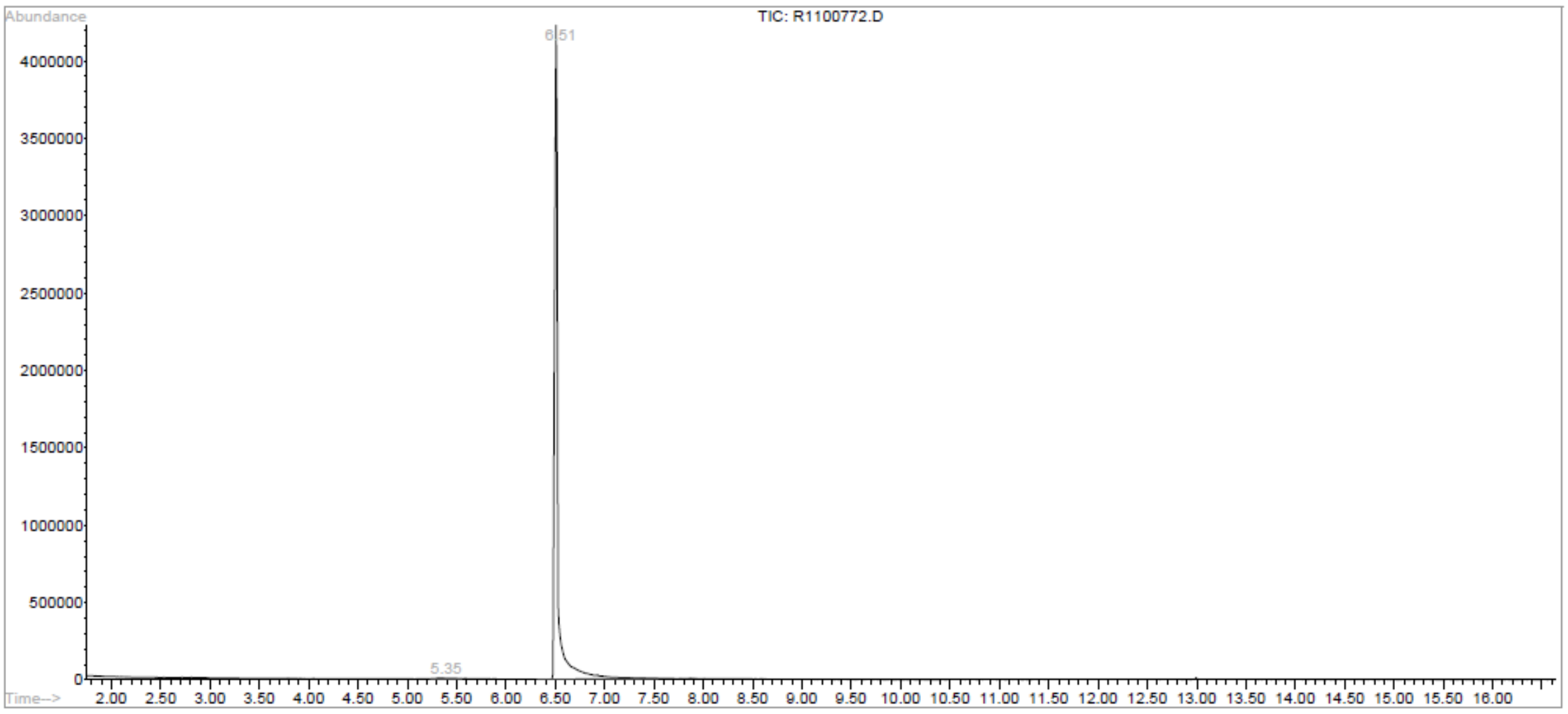

Library Search Report - Chemstation Integrator

Unknown Spectrum based on Apex

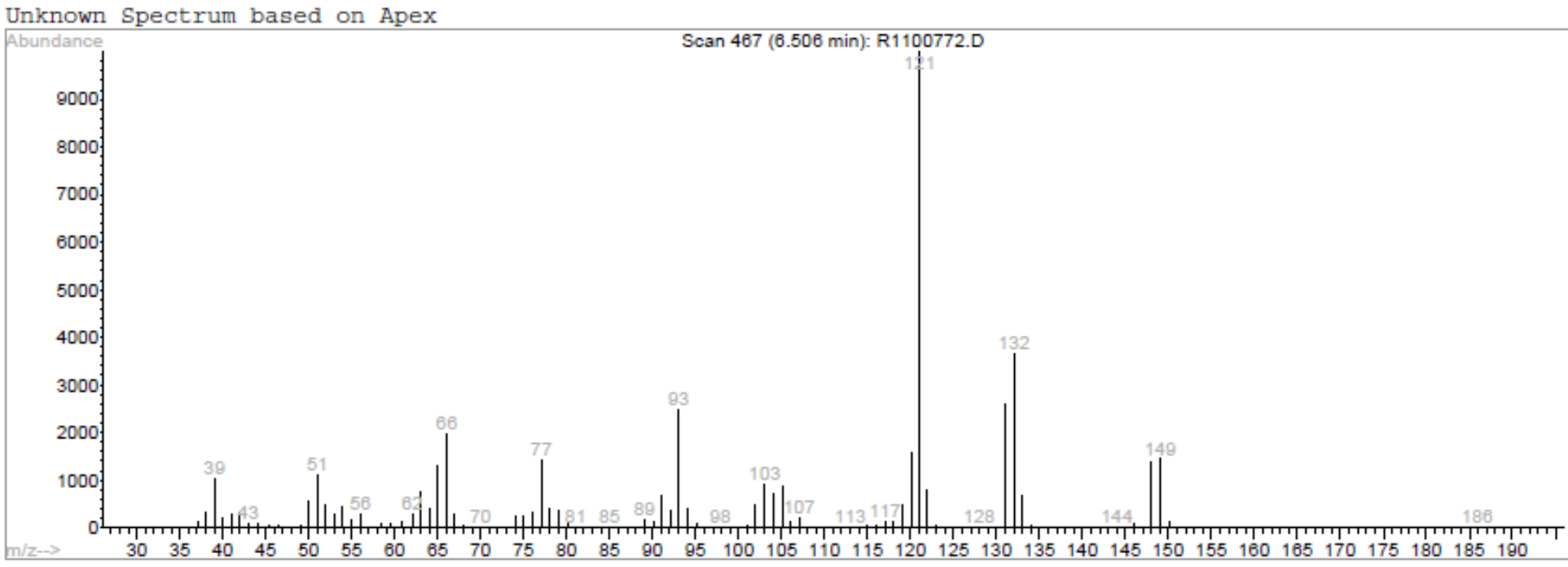

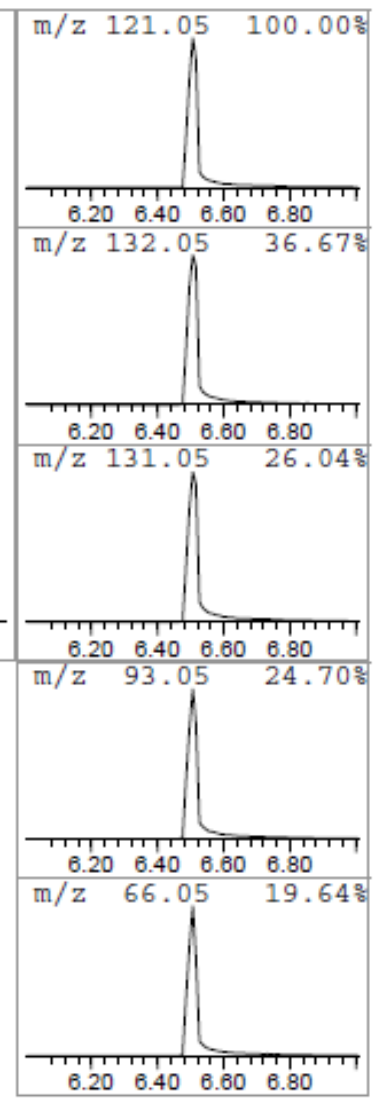


```
Peak Number:  2     at      6.51 min  Area: 100345785   Area % 99.48

 The 3 best hits from each library.          Ref#     CAS#       Qual
---------------------------------------------------------------------

C:\DATABASE\EMPTY.L     No hits were retrieved.
```

Z85923435

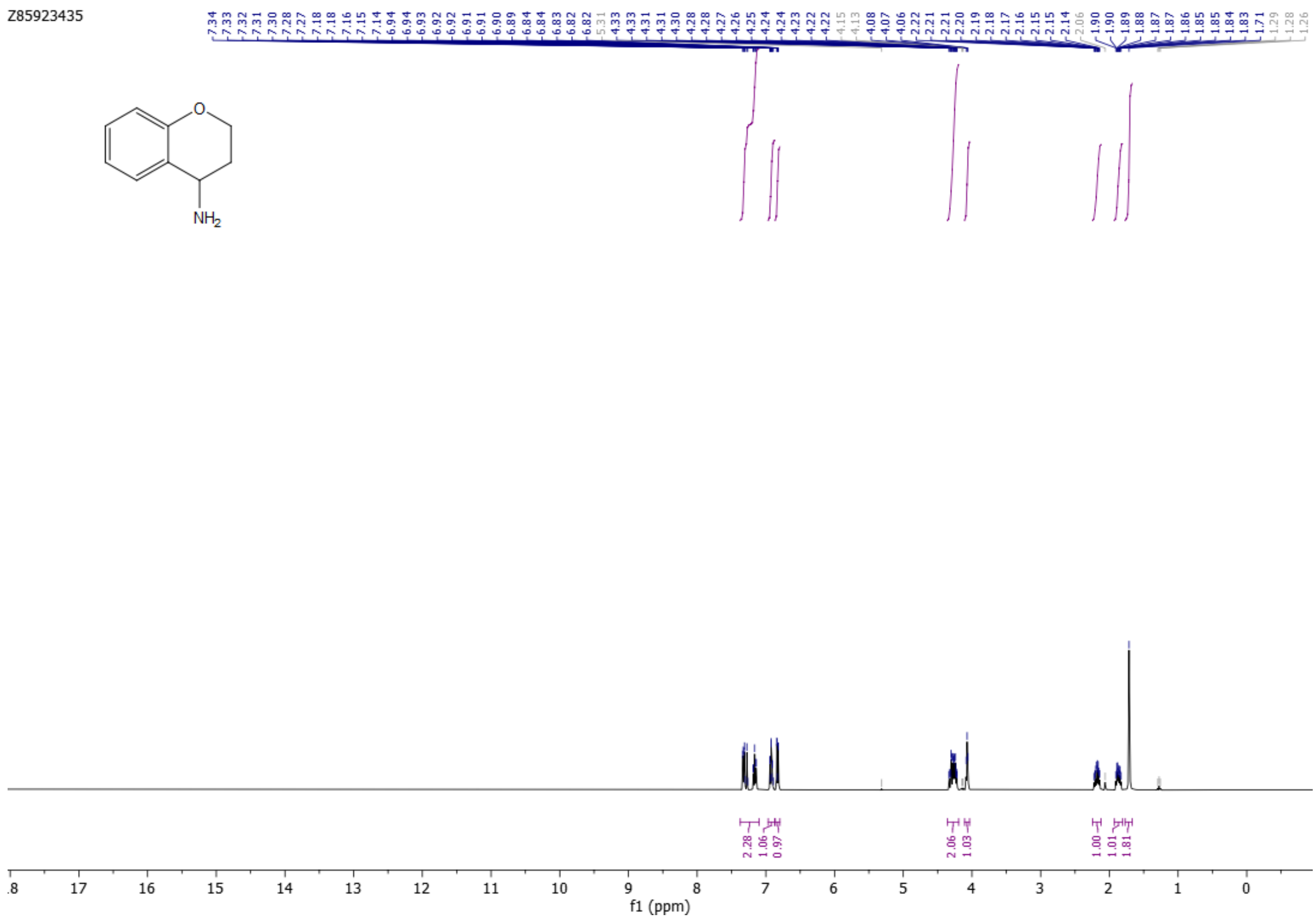

Z85923435

MaxPeak: 100.00%
Ret_Time: 0.678 min

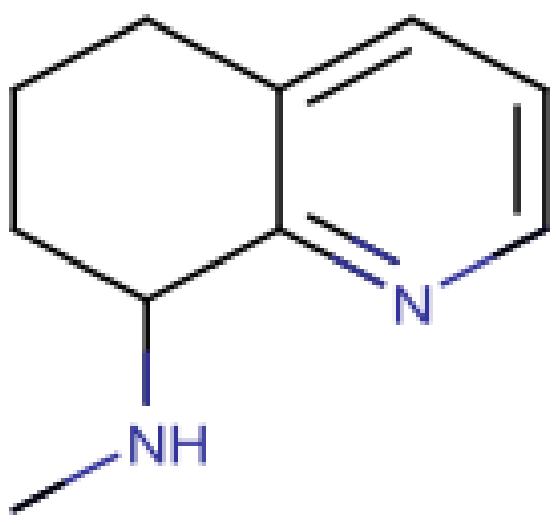

Mol Wt 162.23
Exact Mass 162.14

#	Time	Area%
1	0.678	100.00

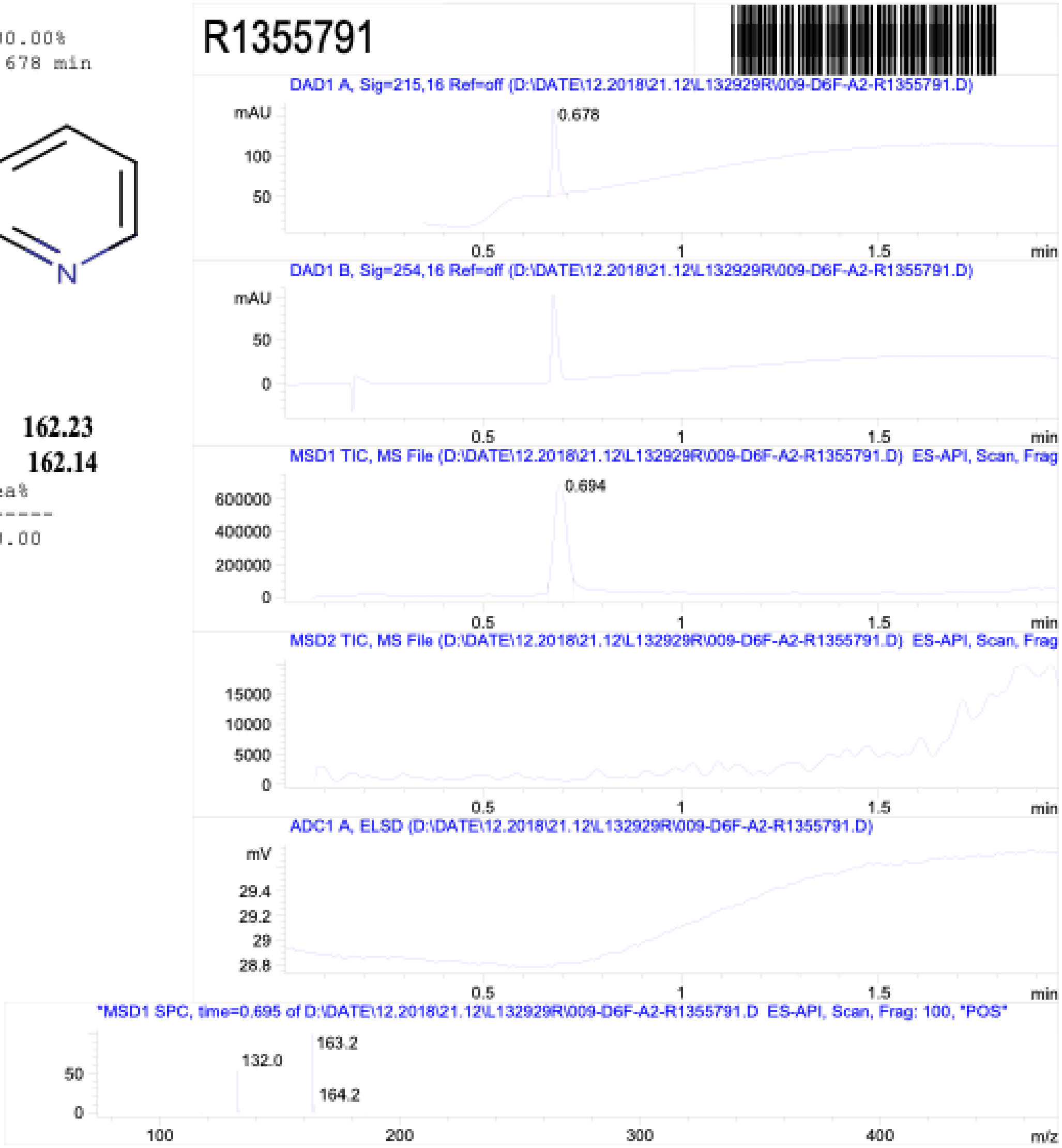

RT 0.694

Inj.Date 12/21/2018 AK <invalid> -6- Acq. Method C:\Chem32\-> ->

Z1262565741

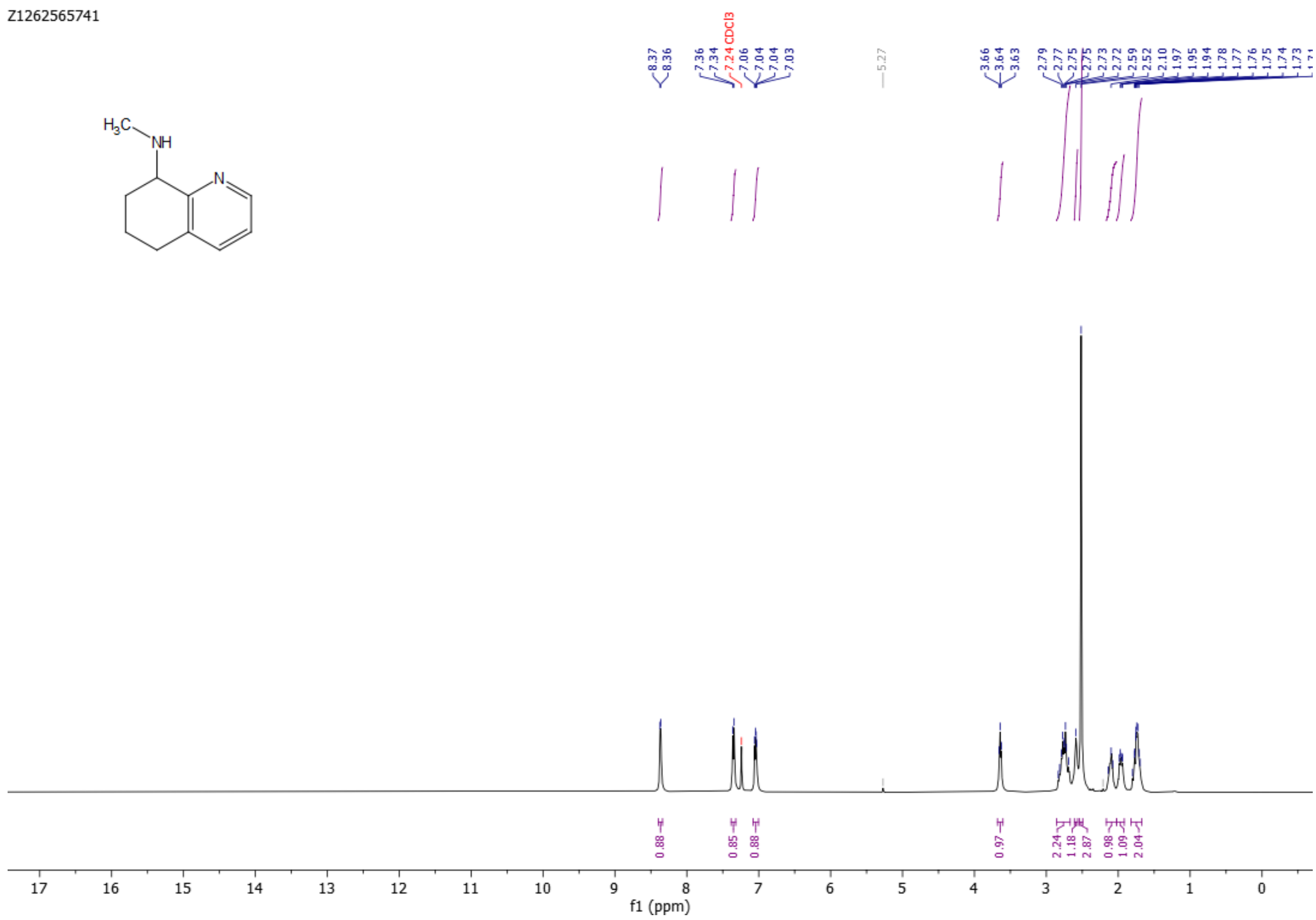

Z1262565741

Error: Peaks not found!

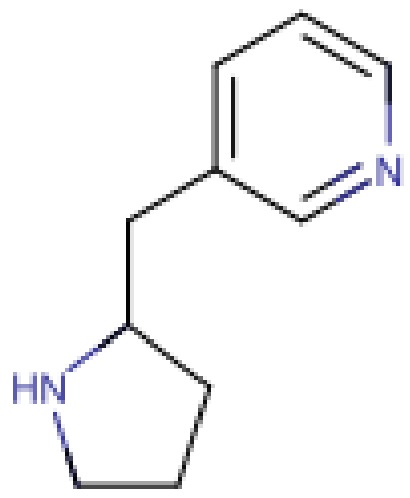

Mol Wt 162.23
Exact Mass 162.14

Error: Peaks not found!

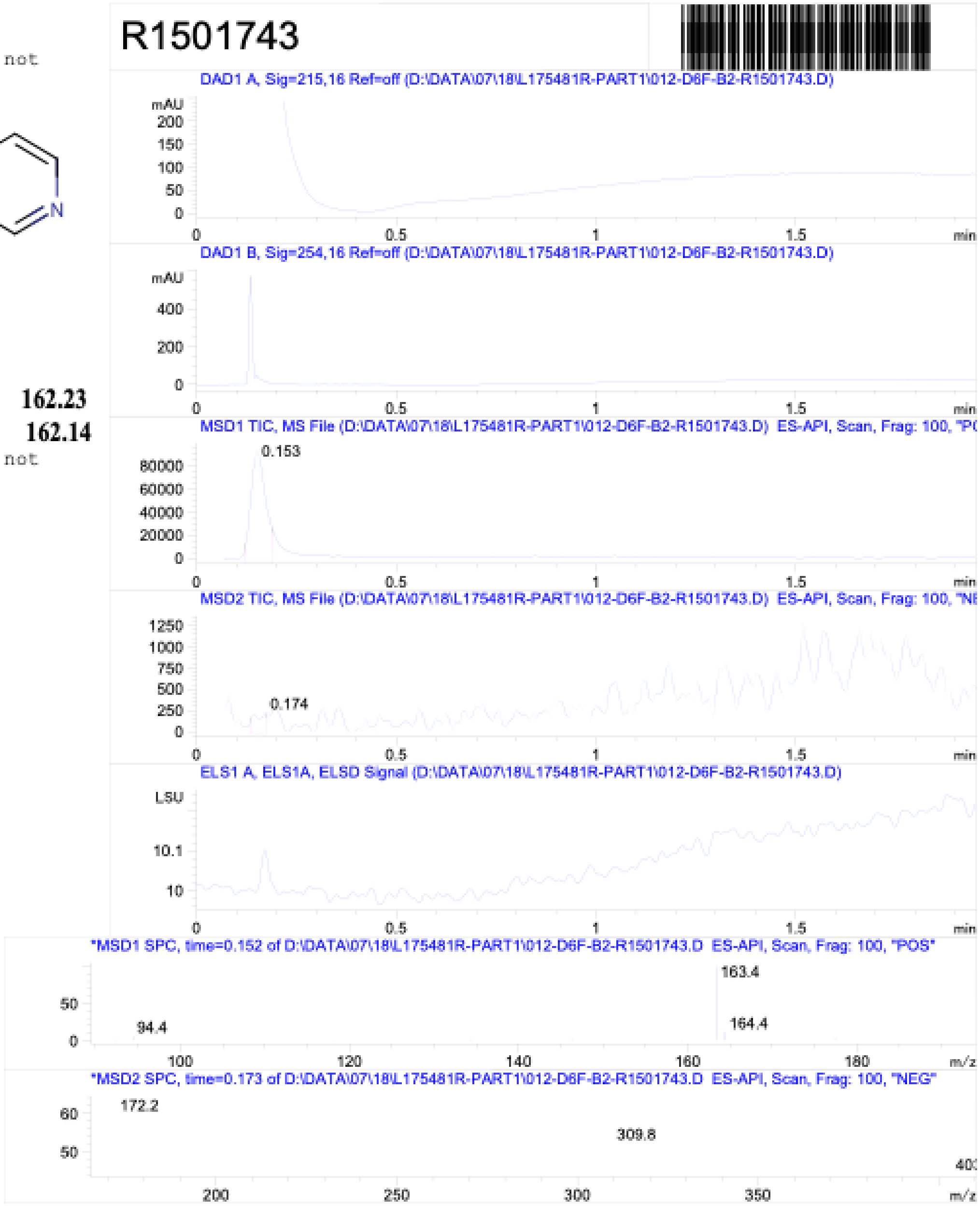

RT 0.153

RT 0.174

Inj.Date 7/18/2019 LB <invalid> -14- Acq. Method C:\Chem32\-> ->

Z1198160843

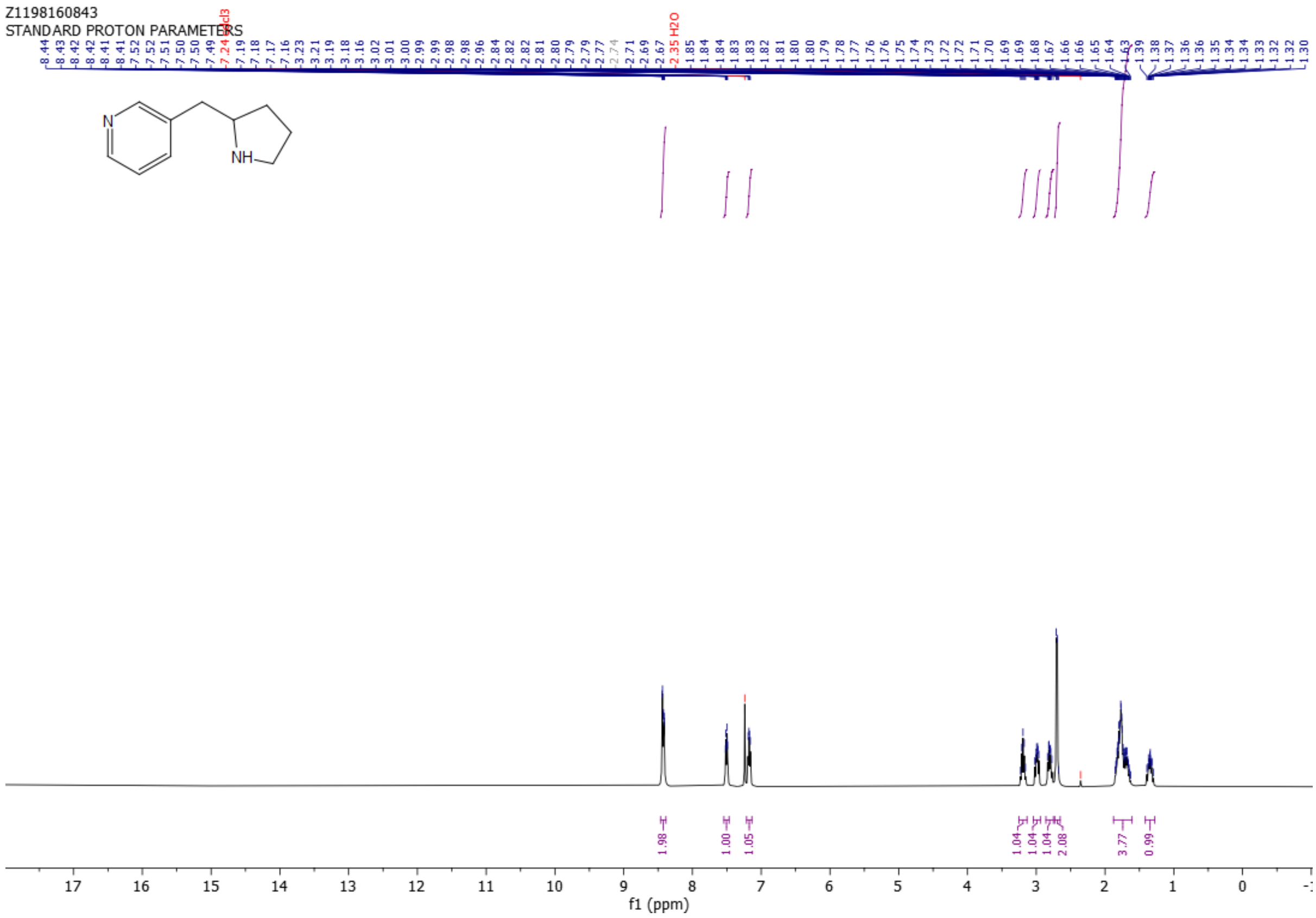

Z1198160843

MaxPeak: 100.00%
Ret_Time: 0.745 min

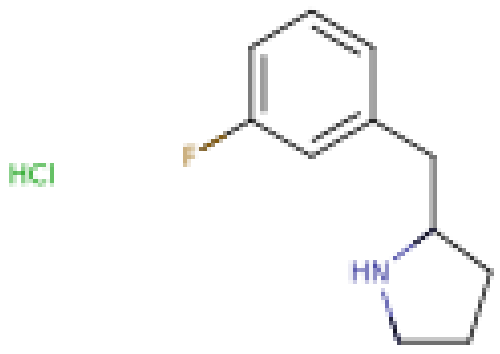

Mol Wt 215.7
Exact Mass 179.14

#	Time	Area%
1	0.745	100.00

RT 0.775

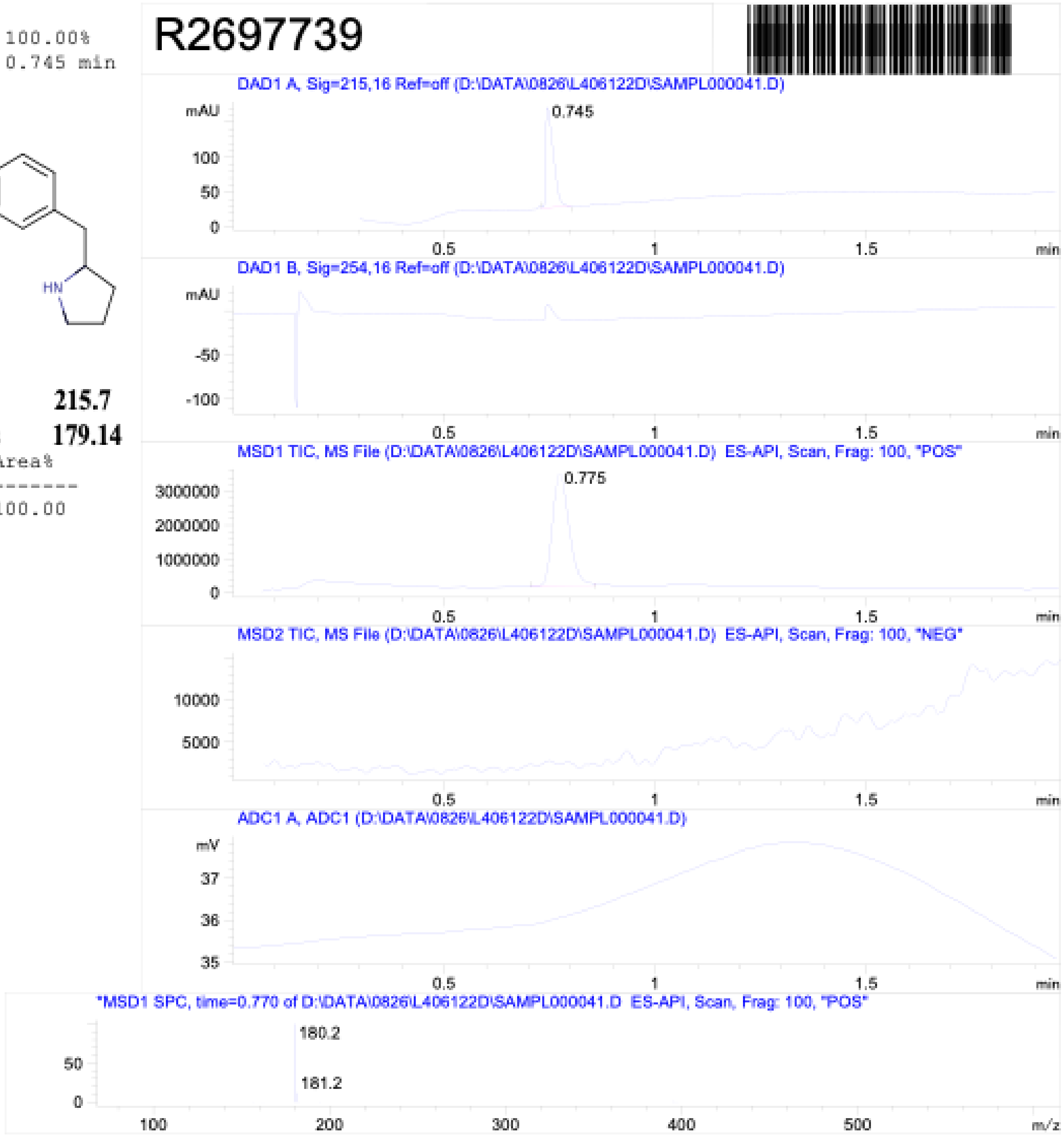

Inj.Date 8/26/2021 H - 4 - Acq. Method C:\CHEM32\-> ->

Z449369104

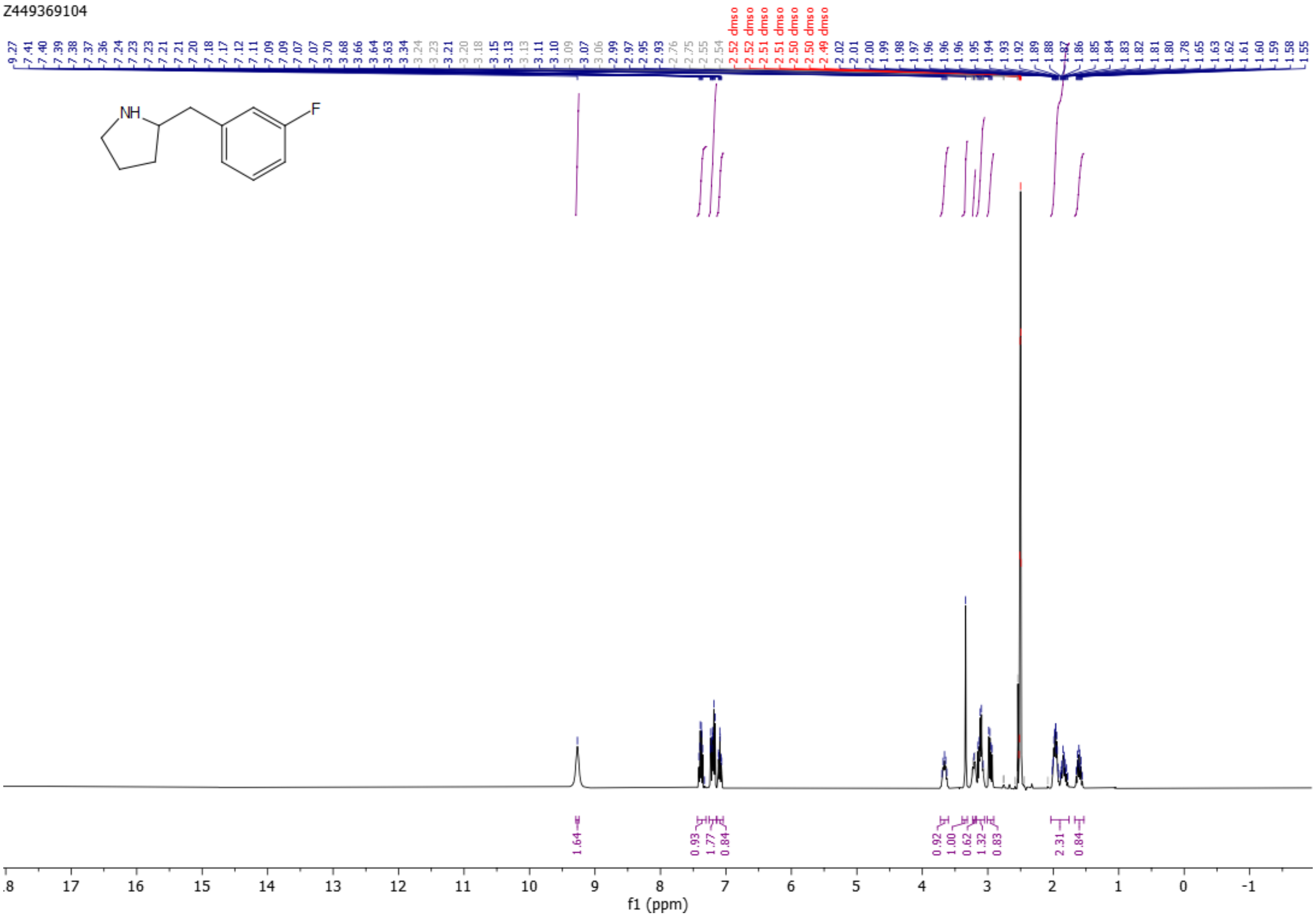

Z449369104

MaxPeak: 100.00%
Ret_Time: 0.711 min

HCl

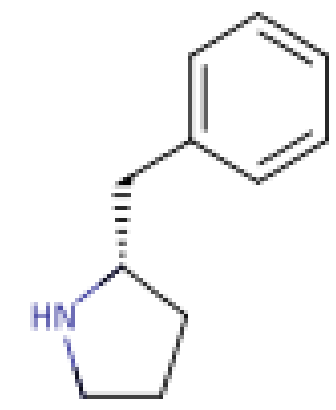

Mol Wt 197.7
Exact Mass 161.15

#	Time	Area%
1	0.711	100.00

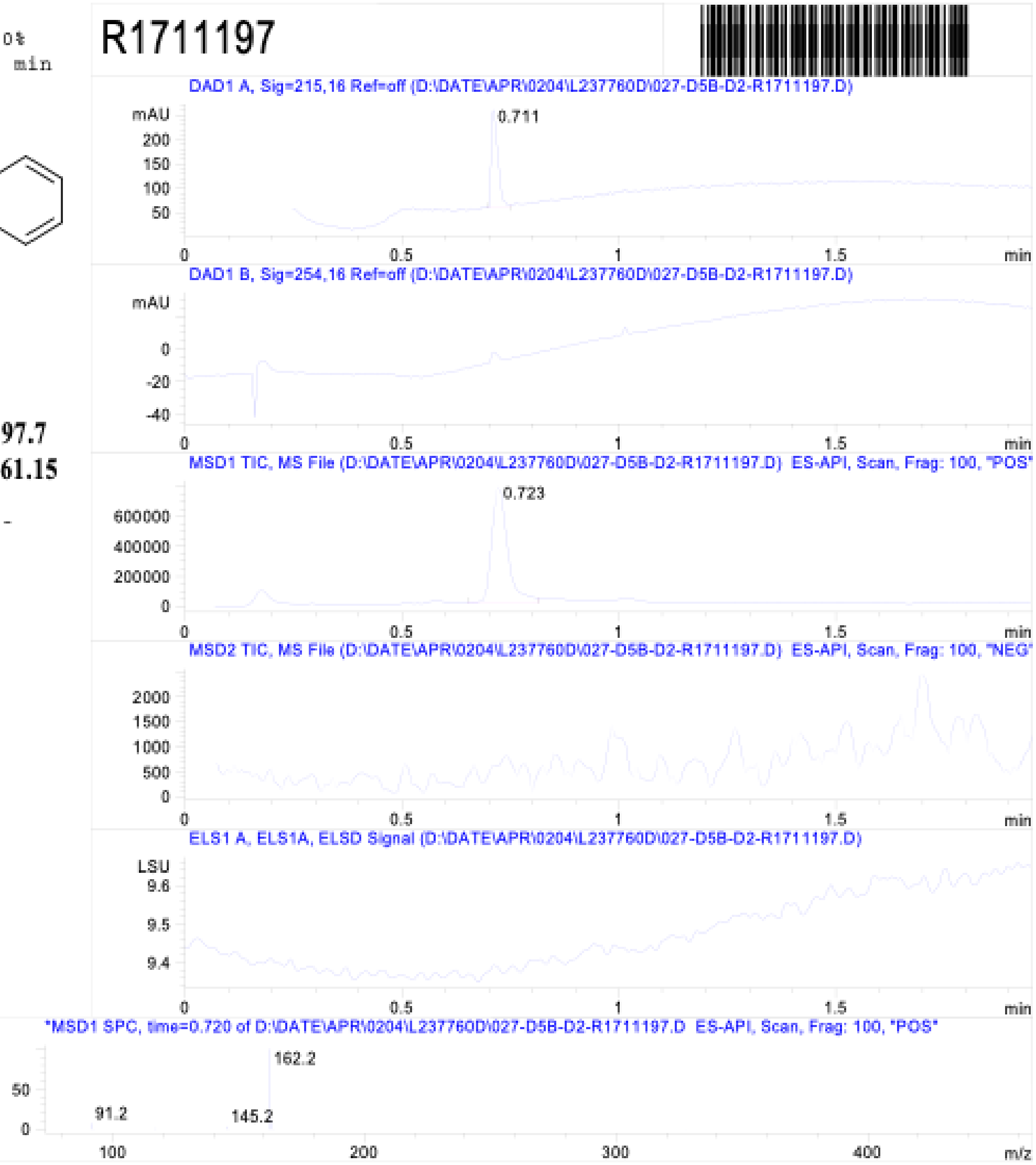

RT 0.723

Inj.Date 4/2/2020 Y -14- Acq. Method C:\Chem32\-> ->

Z3244742843

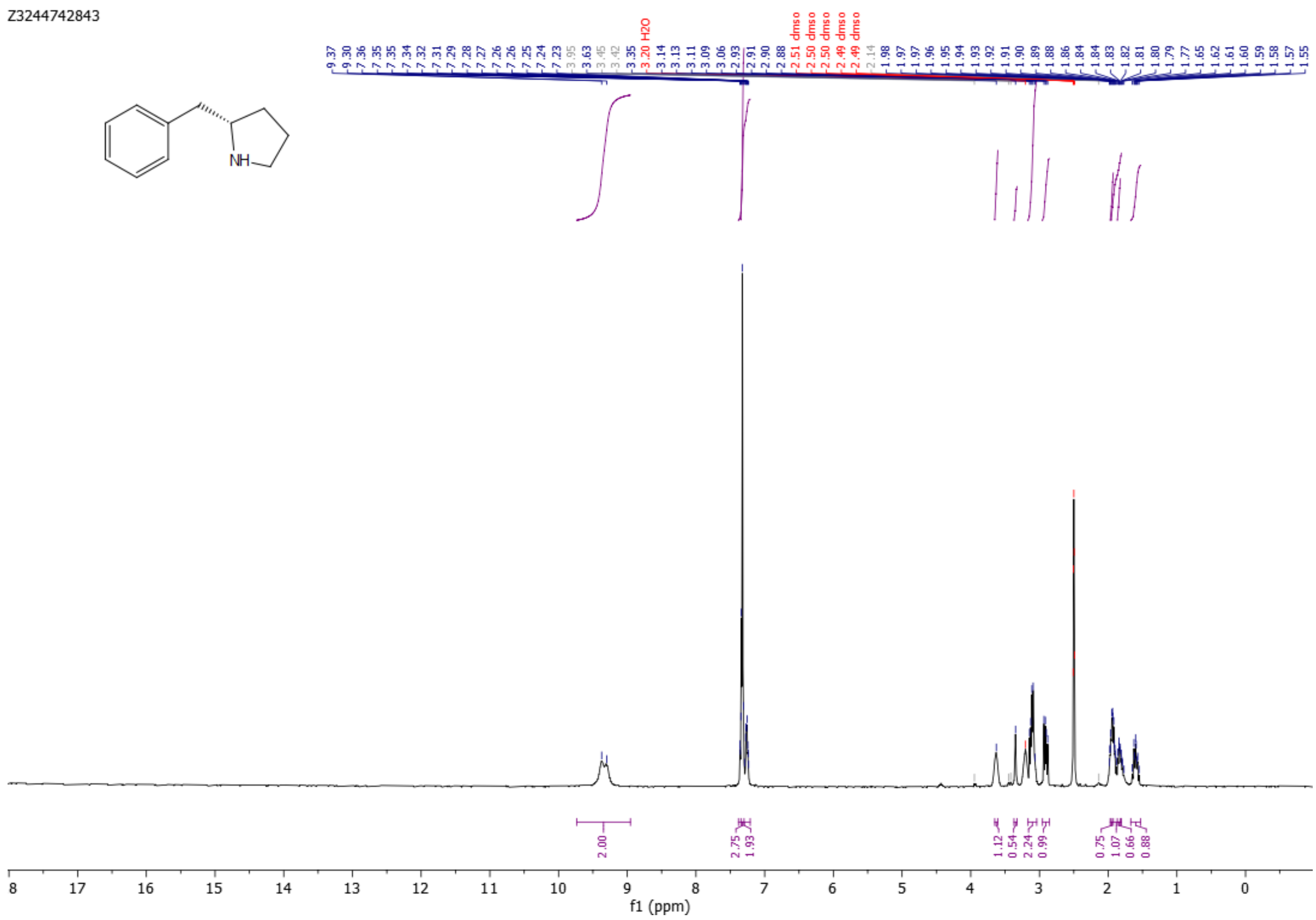

Z3244742843

MaxPeak: 100.00%
Ret_Time: 0.622 min

HCl

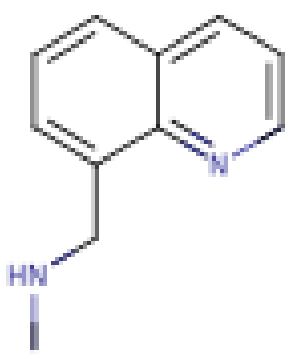

Mol Wt 208.69
Exact Mass 172.12

#	Time	Area%
1	0.622	100.00

RT 0.647

RT 0.666

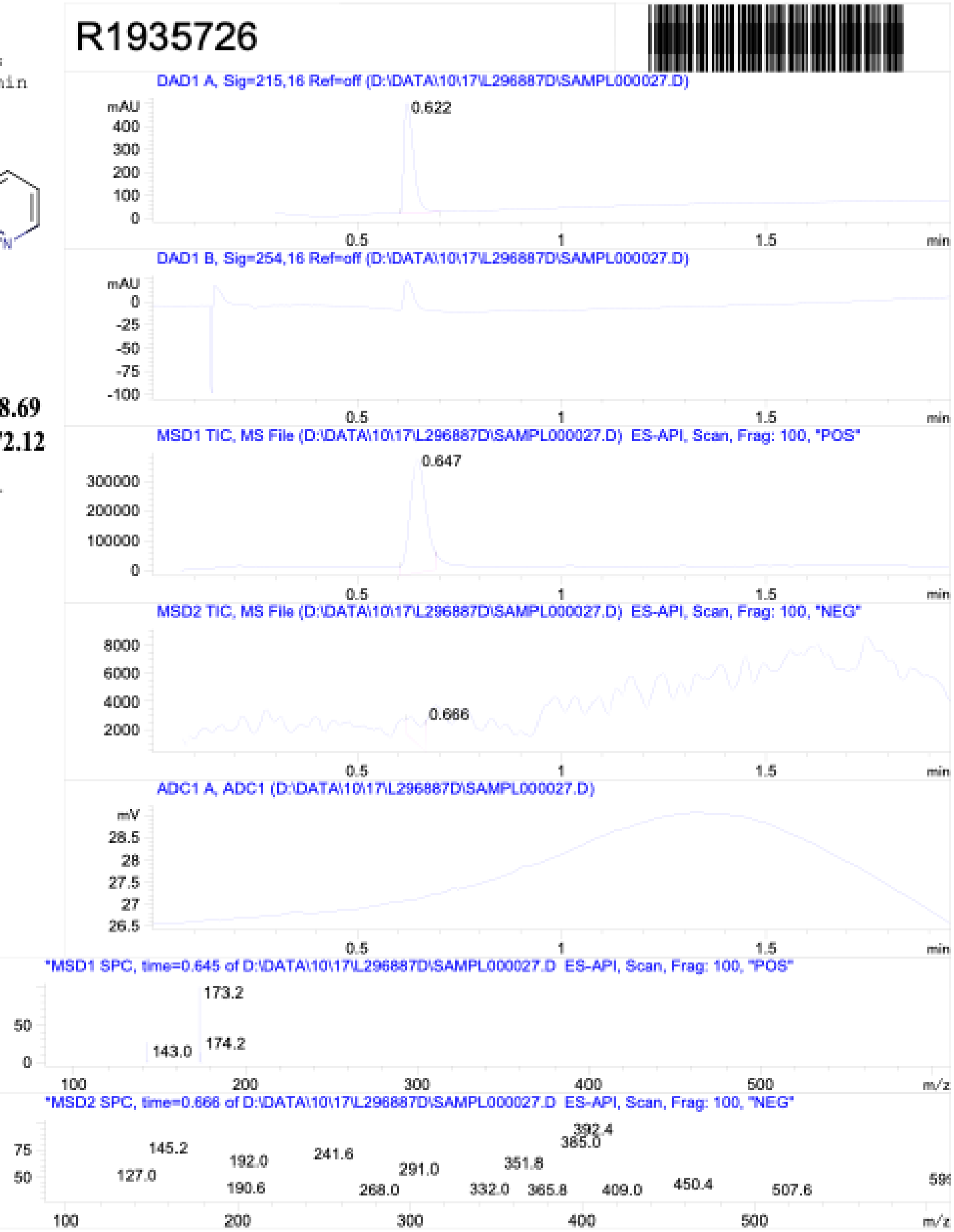

Inj.Date 10/17/2020 LB - 4 - Acq. Method C:\CHEM32\-> ->

Z4710975379

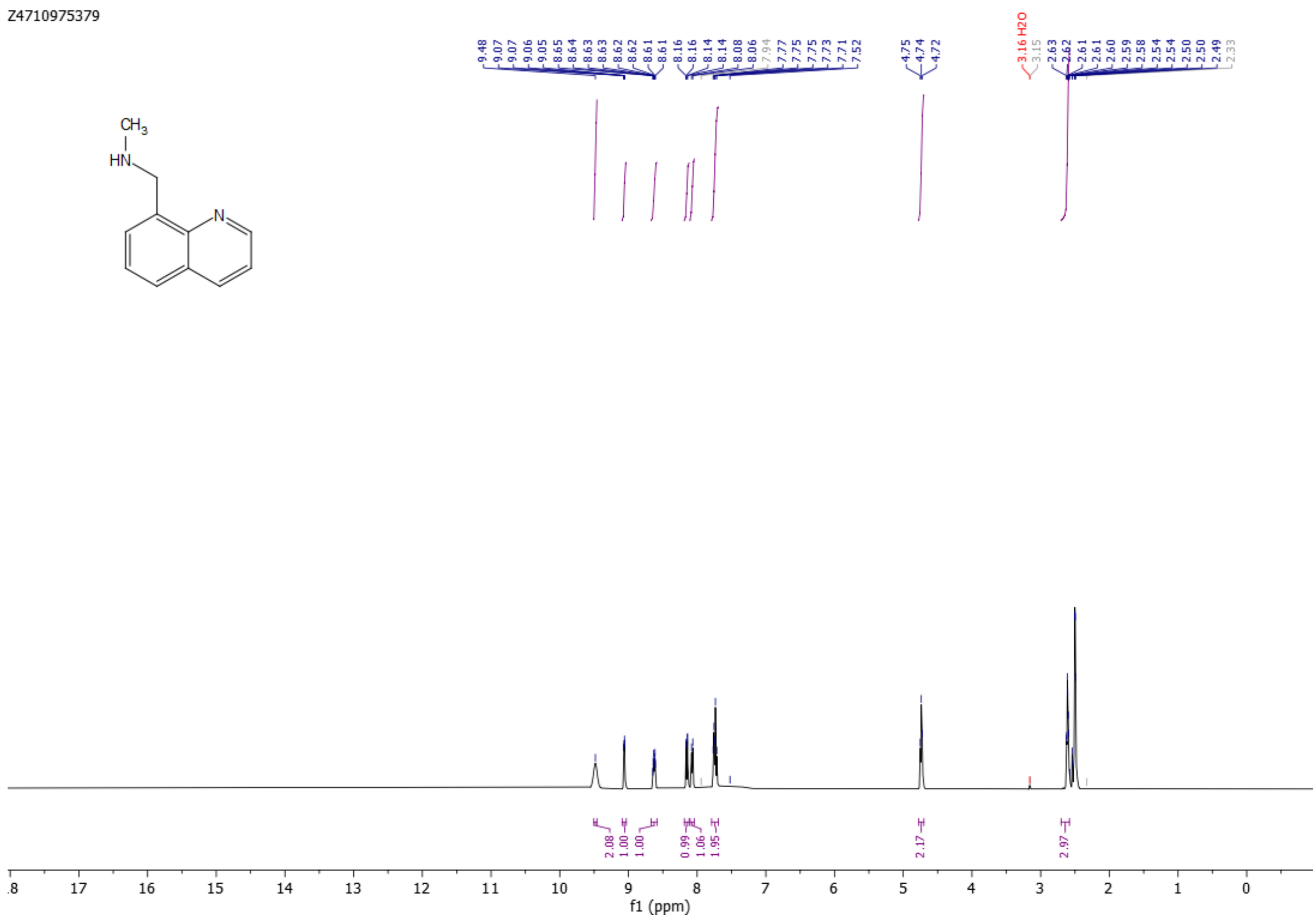

Z4710975379

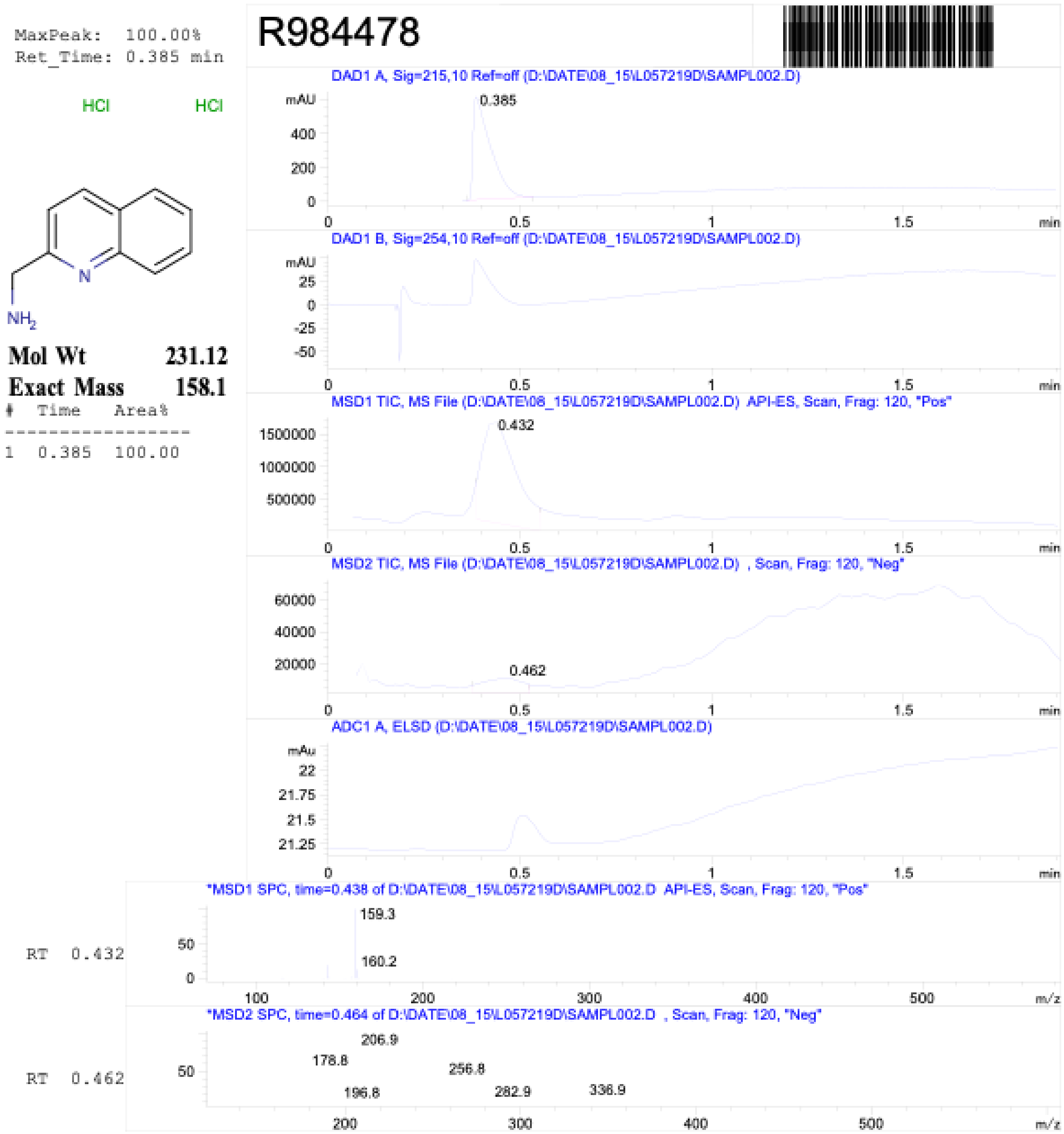

Inj.Date 8/16/2017 E P2-A-02 -SL- Acq. Method C:\HPCHEM\-> ->

Z2106596235

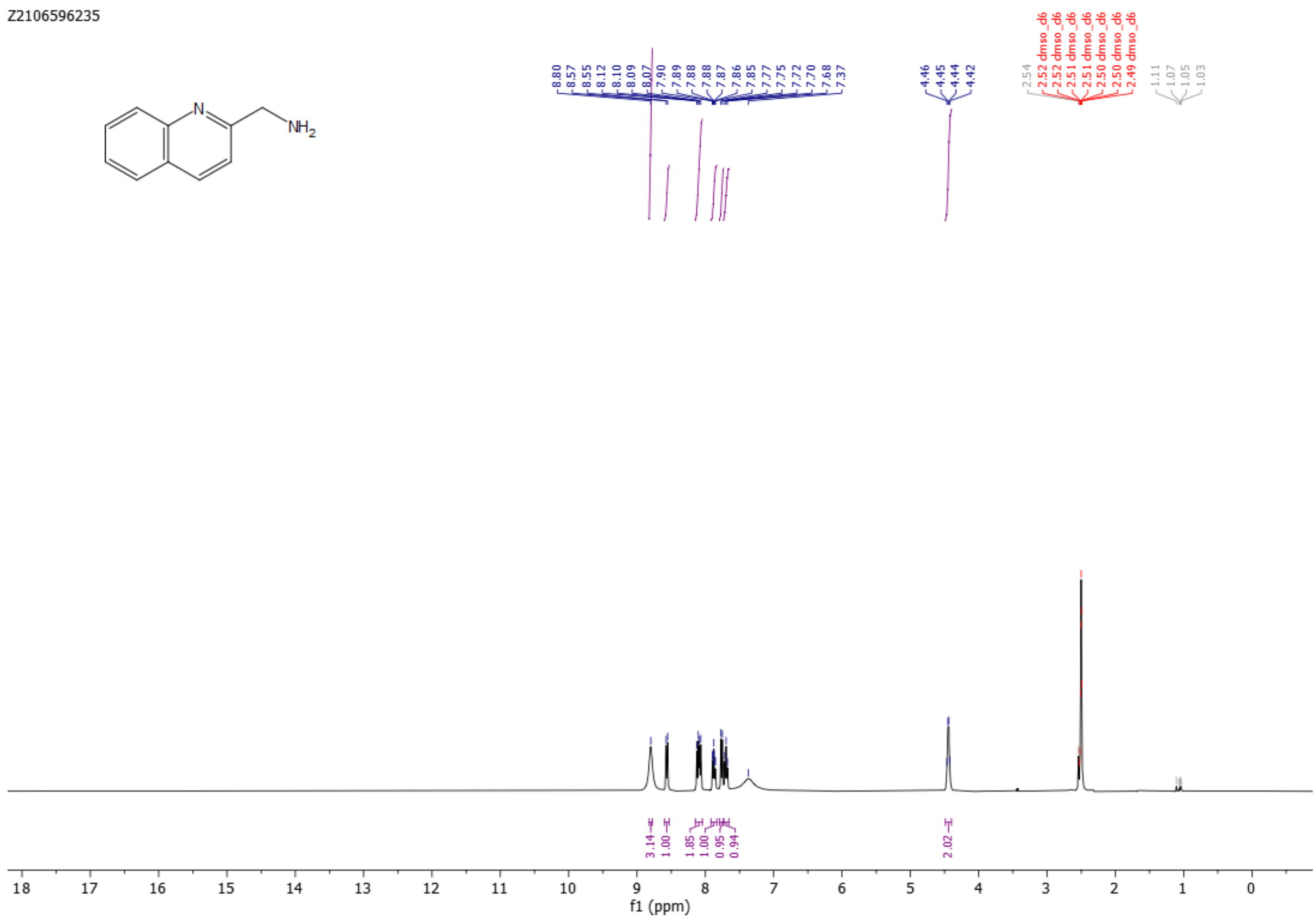

Z2106596235

MaxPeak: 98.60%
Ret_Time: 0.827 min

BC826342$1

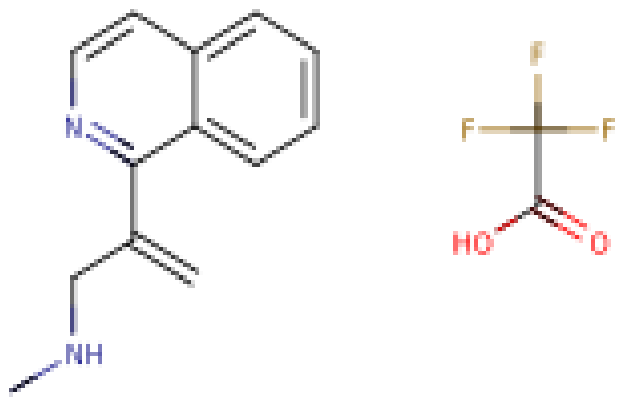

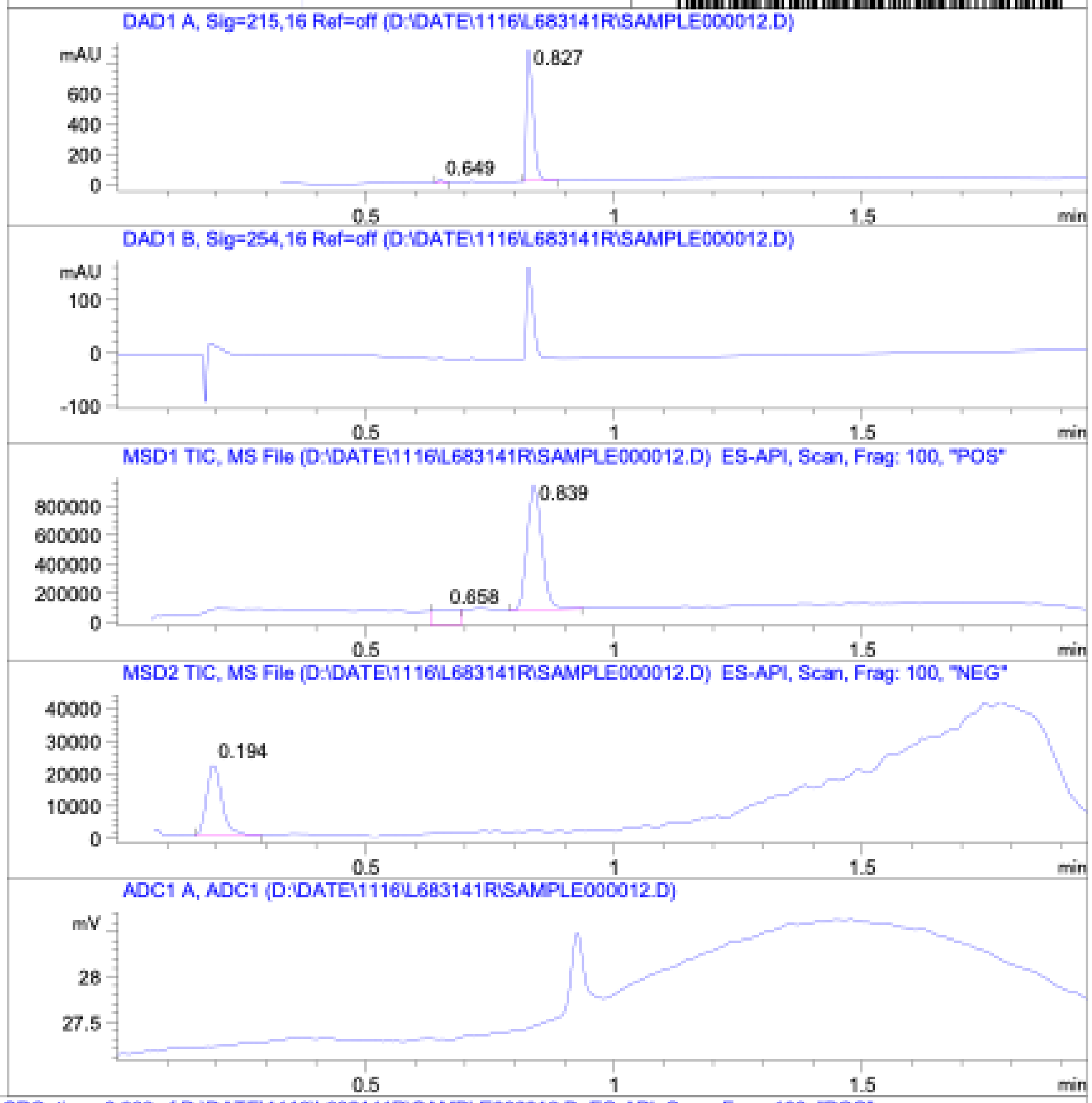

Mol Wt 312.29
Exact Mass 198.14

#	Time	Area%
1	0.649	1.40
2	0.827	98.60

RT 0.658

RT 0.839

RT 0.194

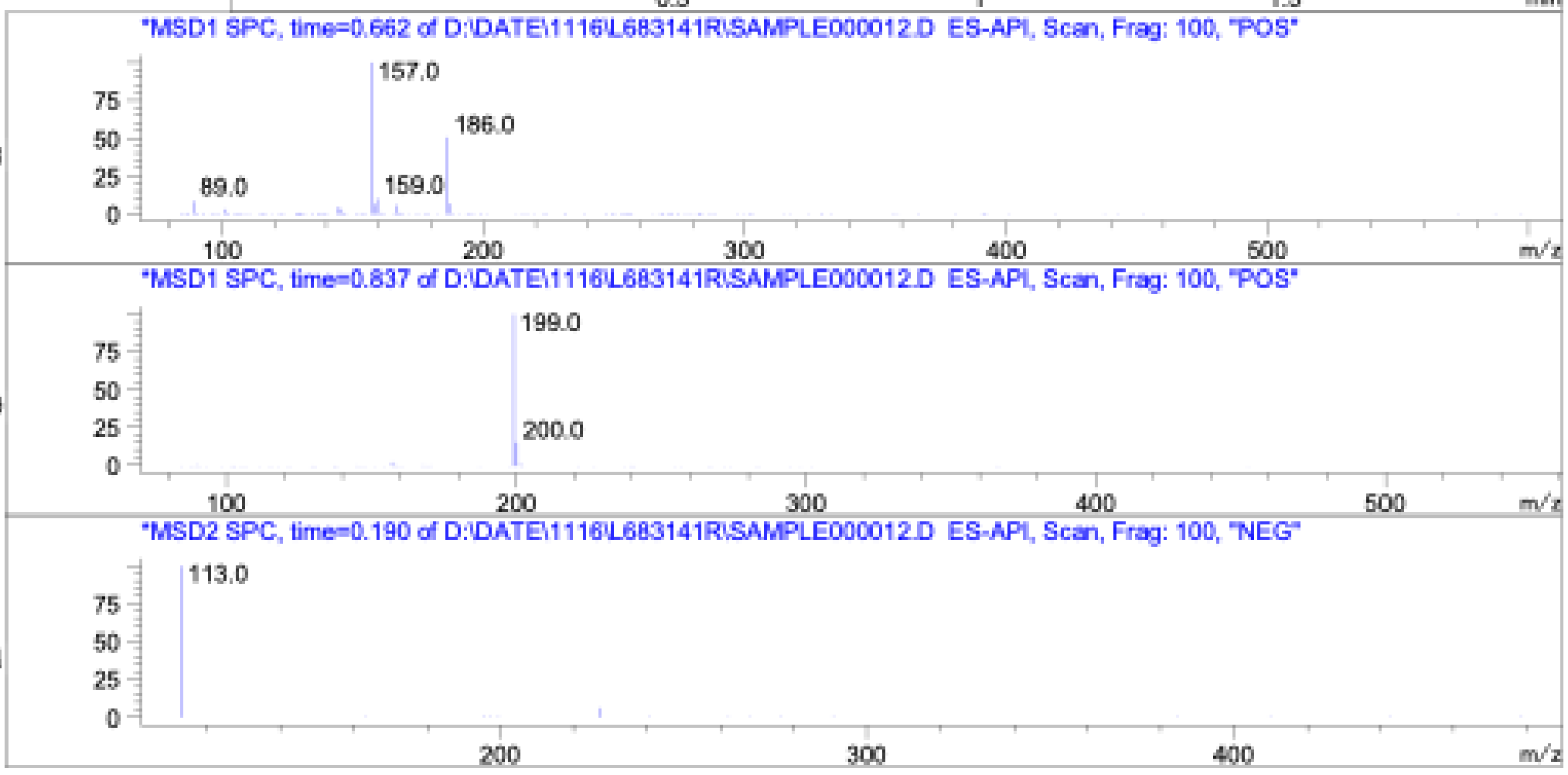

Inj.Date 11/16/2023 CH - 4 - Acq. Method C:\CHEM32\-> ->

Z8843458836

MaxPeak: 100.00%
Ret_Time: 0.739 min

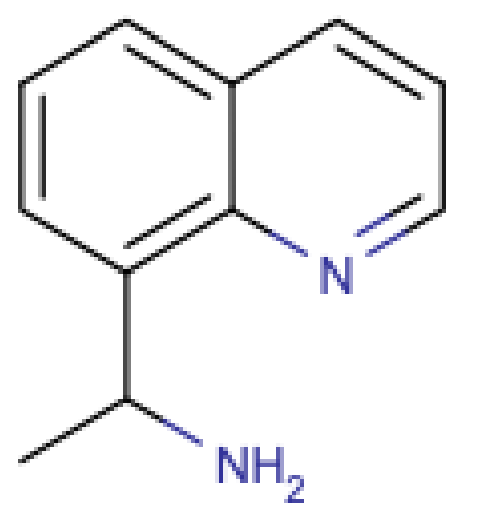

Mol Wt 172.23
Exact Mass 172.12

#	Time	Area%
1	0.739	100.00

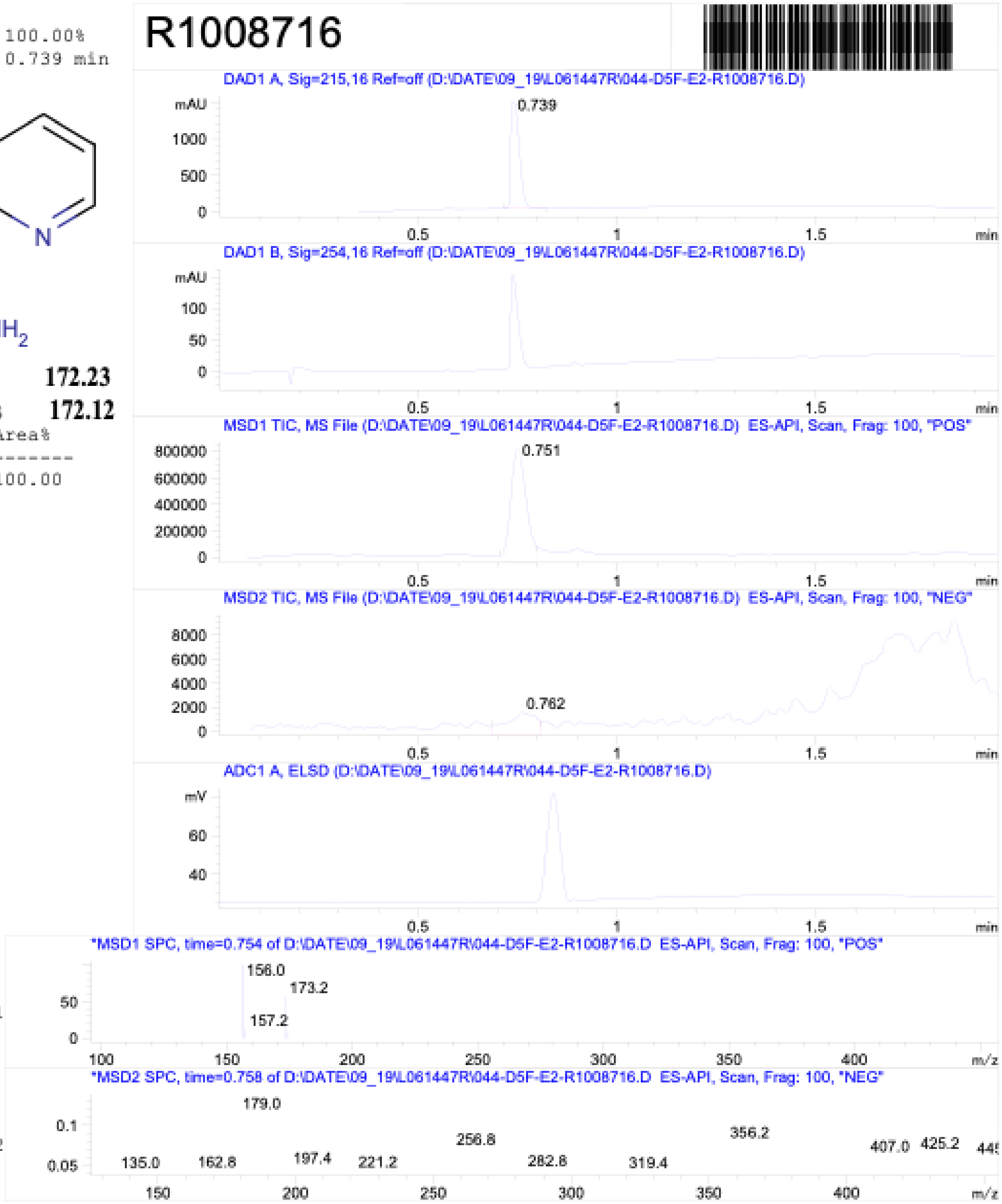

RT 0.751

RT 0.762

Inj.Date 9/19/2017 E <invalid> -8- Acq. Method C:\Chem32\-> ->

Z1198724030

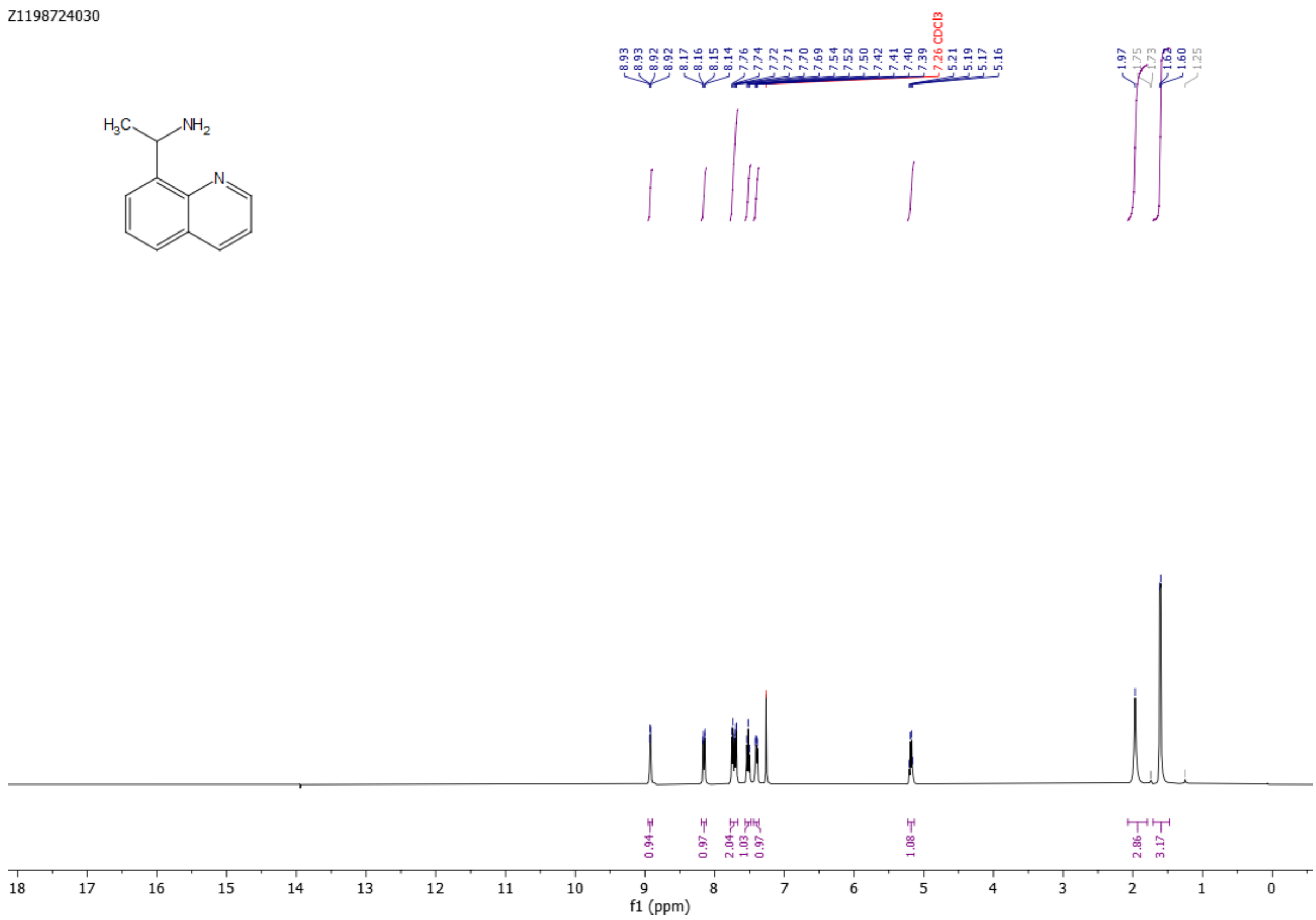

Z1198724030

MaxPeak: 100.00%
Ret_Time: 0.684 min

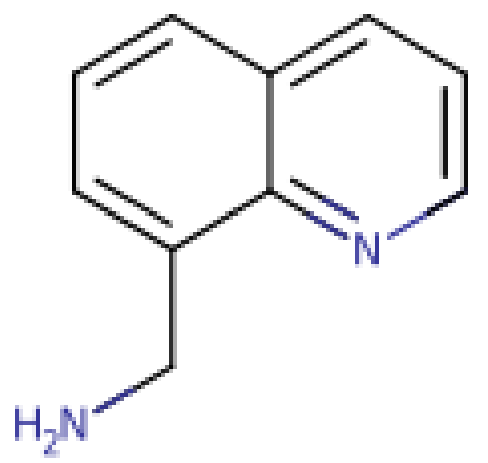

Mol Wt 158.2
Exact Mass 158.1

#	Time	Area%
1	0.684	100.00

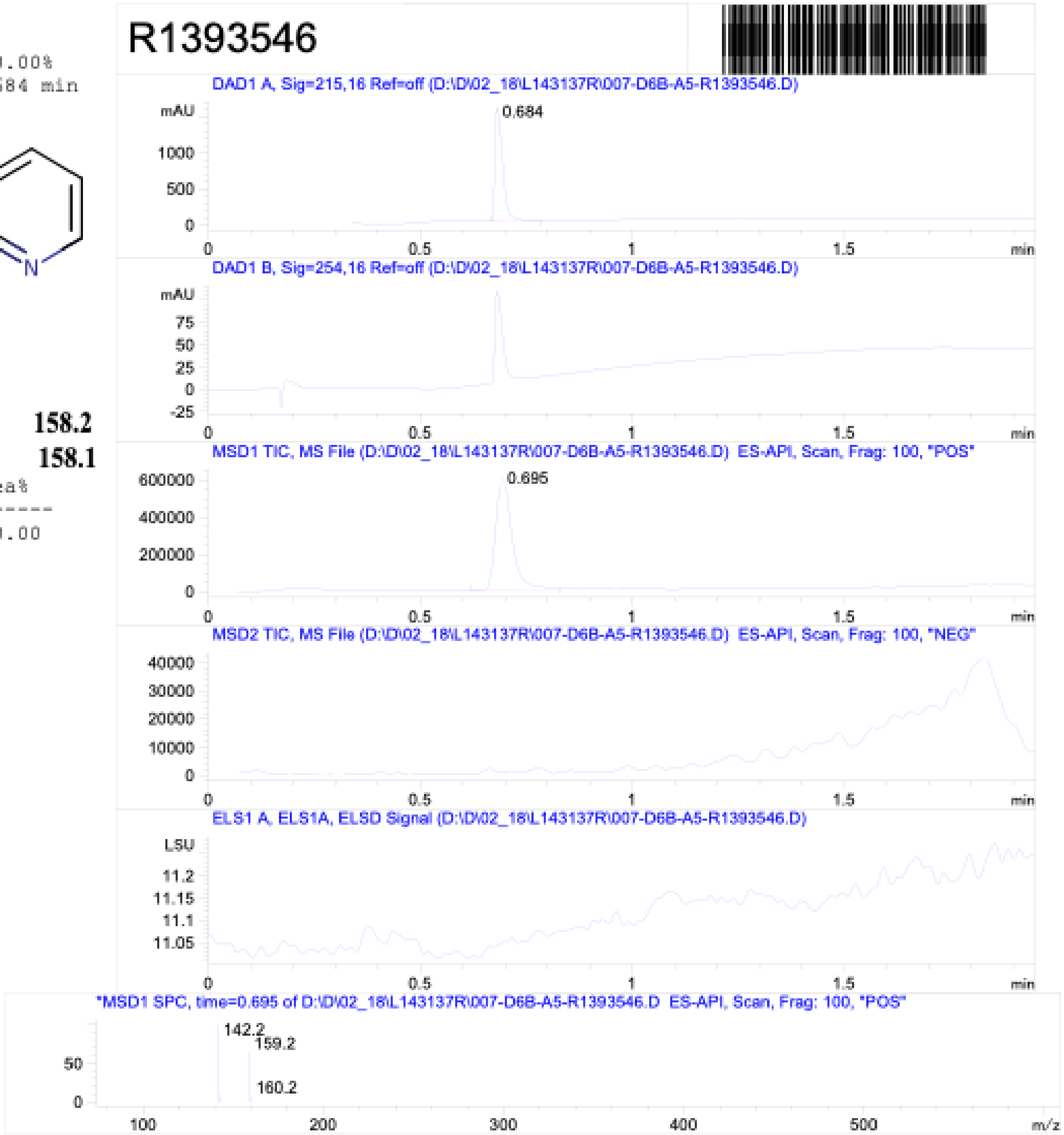

RT 0.695

Inj.Date 2/18/2019 N <invalid> -16- Acq. Method C:\Chem32\-> ->

Z234897549

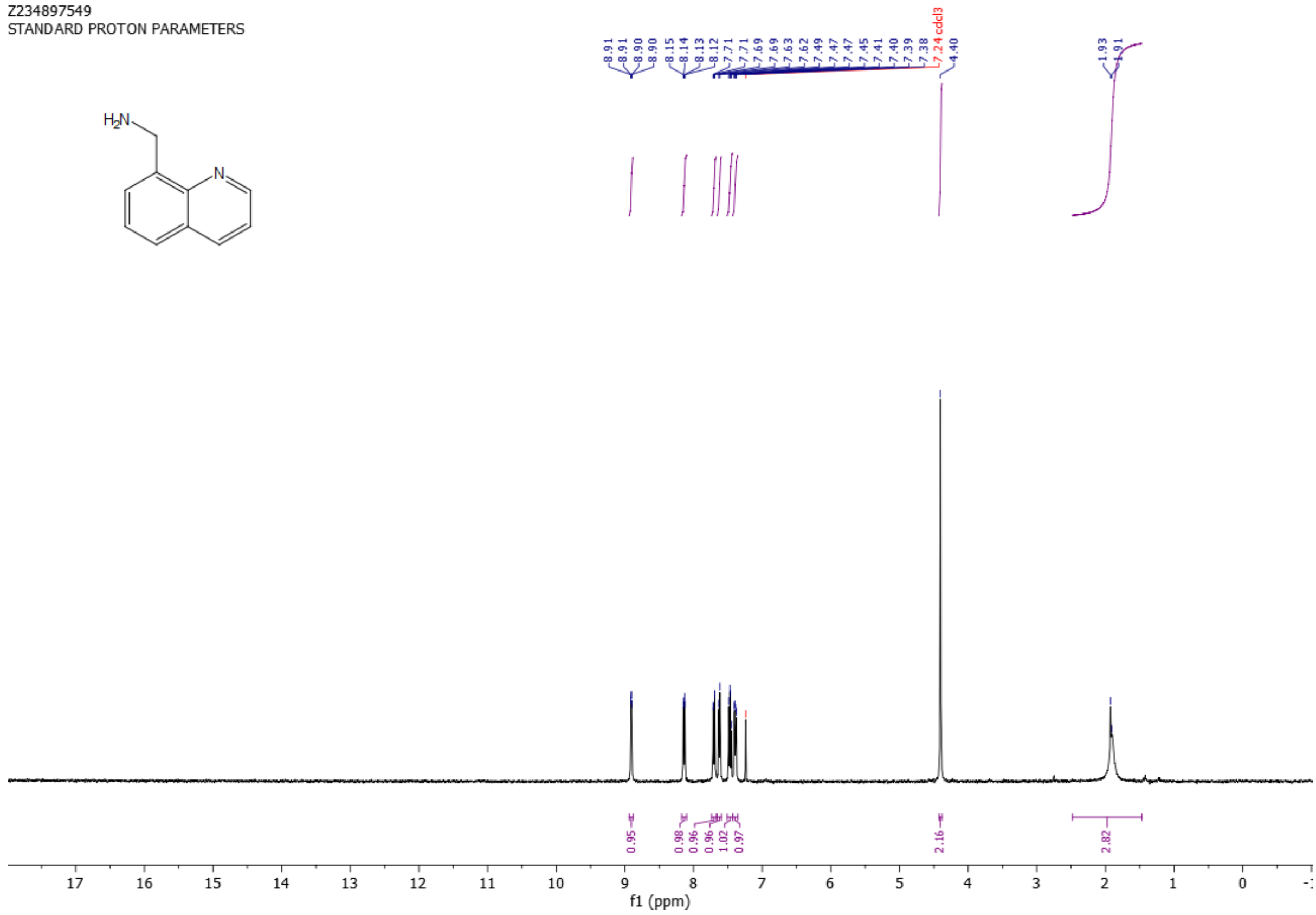
Z234897549
STANDARD PROTON PARAMETERS
H2N
N
8.91 8.91 8.90 8.90 8.15 8.14 8.13 8.12 7.71 7.71 7.69 7.69 7.63 7.62 7.49 7.47 7.47 7.45 7.41 7.40 7.39 7.38
7.24 cdcl3
4.40
1.93 1.91
0.95
0.98
0.96
0.96
1.02
0.97
2.16
2.82
17 16 15 14 13 12 11 10 9 8 7 6 5 4 3 2 1 0
f1 (ppm)

Z234897549

MaxPeak: 97.27%
Ret_Time: 0.670 min

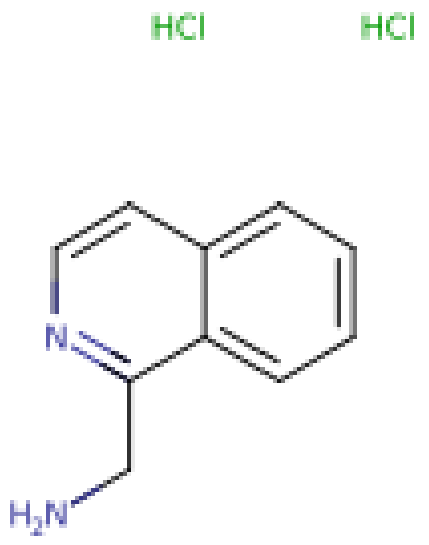

Mol Wt 231.12
Exact Mass 158.1

#	Time	Area%
1	0.670	97.27
2	0.915	2.73

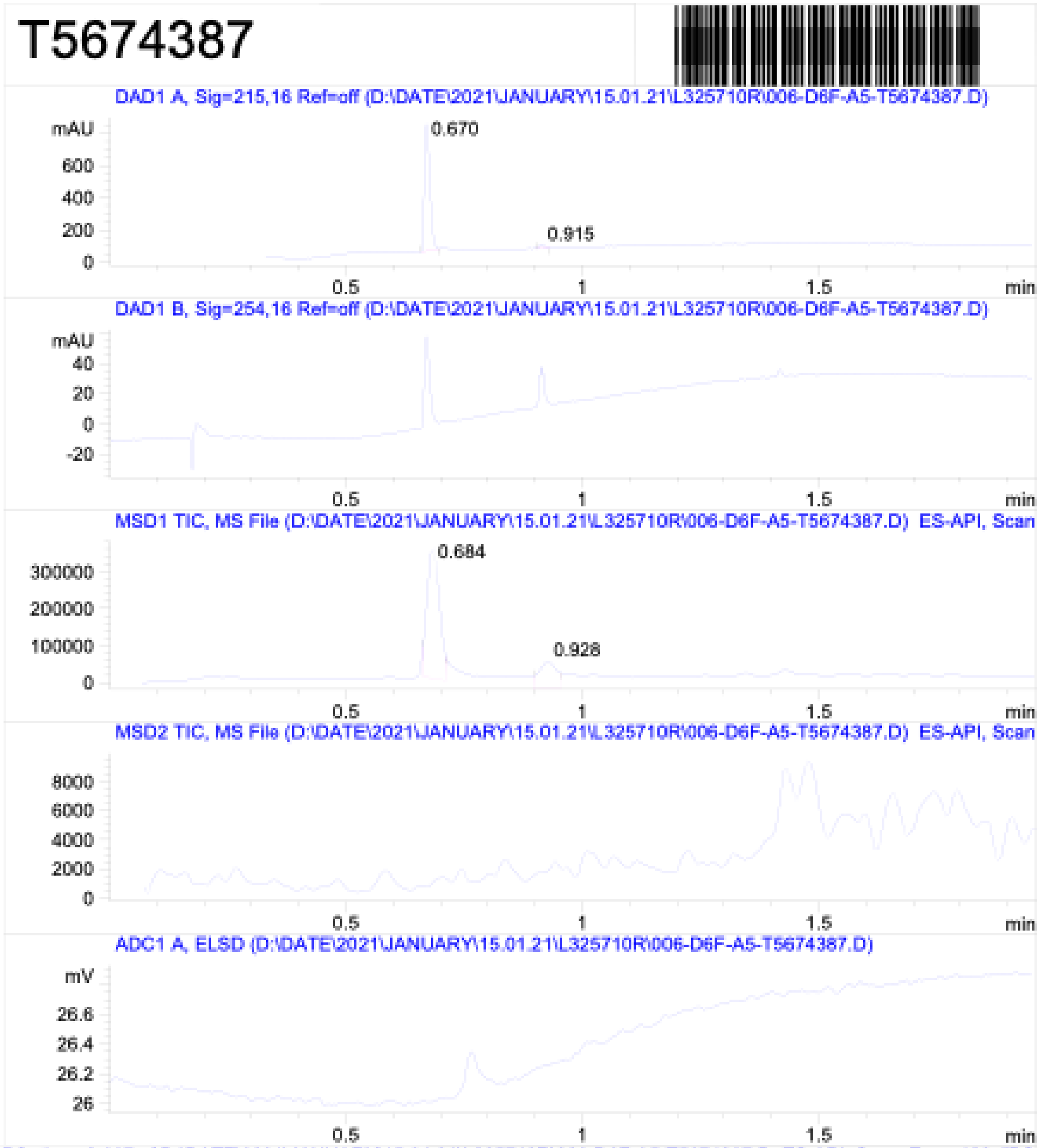

RT 0.684

RT 0.928

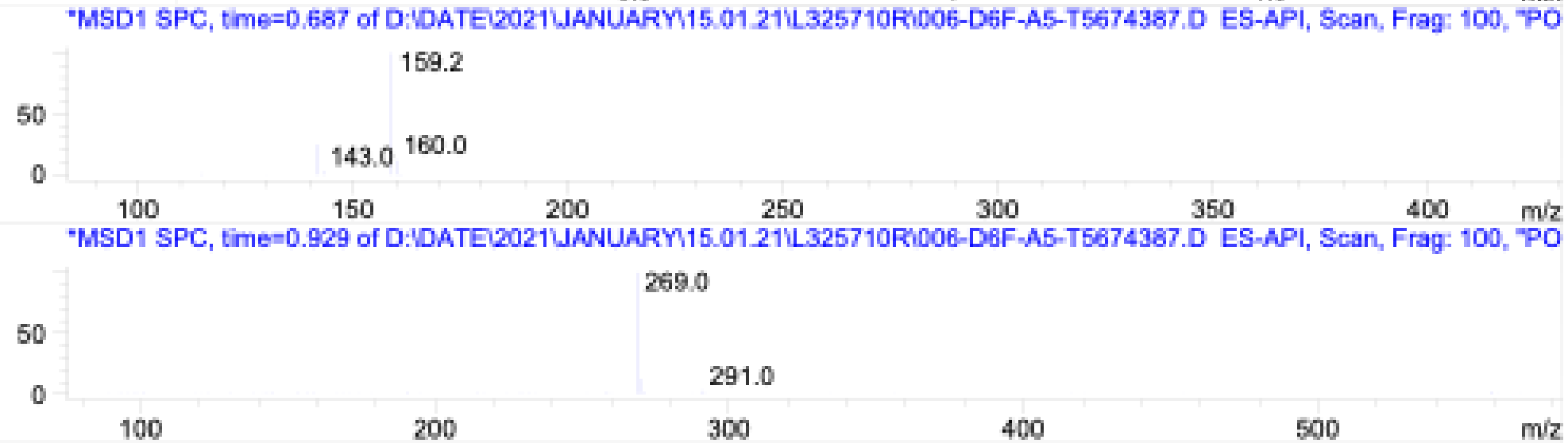

Inj.Date 1/14/2021 T <invalid> -12- Acq. Method C:\Chem32\-> ->

Z220439492

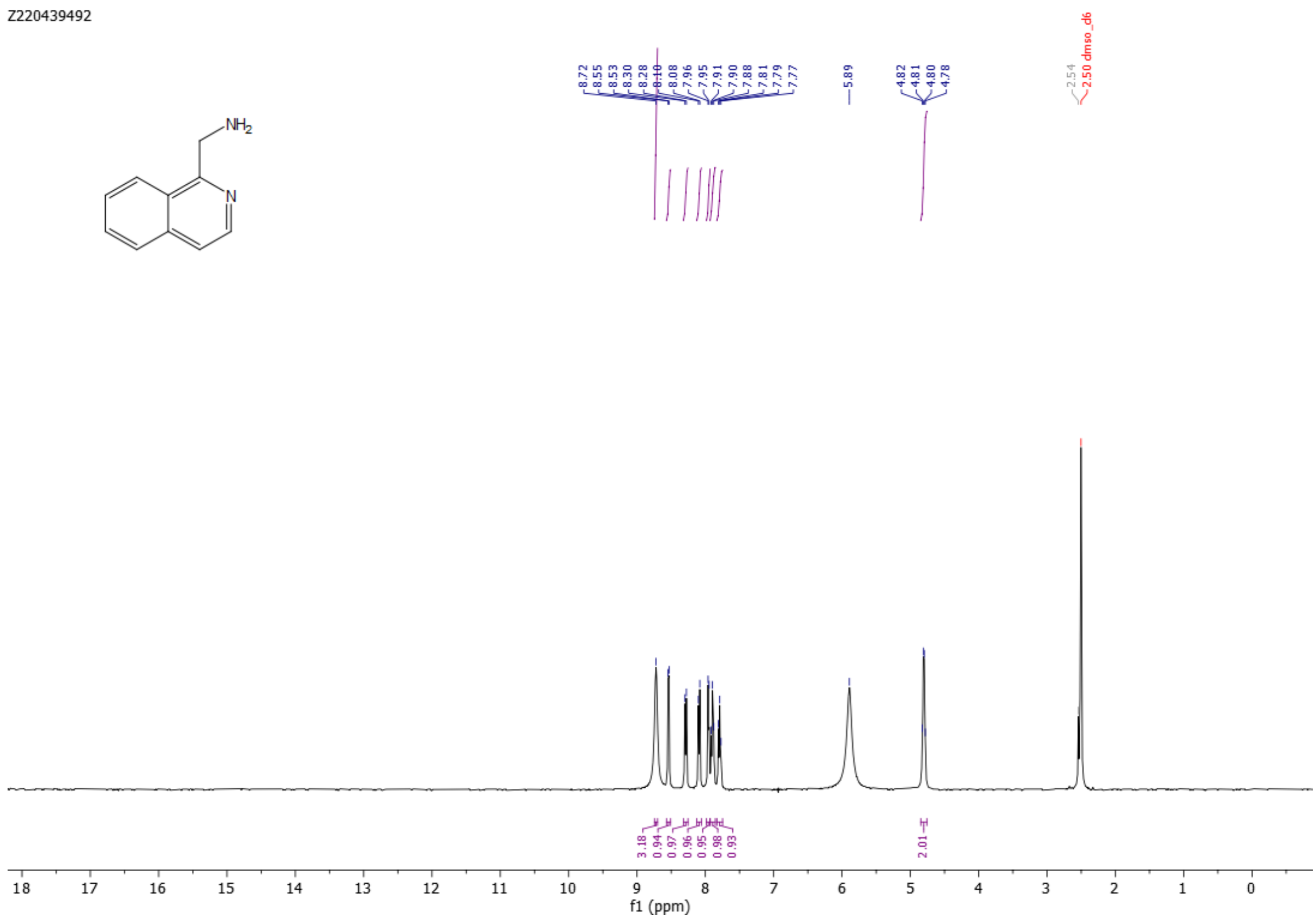

Z220439492
NH2
N
8.72
8.55
8.53
8.30
8.28
8.10
8.08
7.96
7.95
7.91
7.90
7.88
7.81
7.79
7.77
5.89
4.82
4.81
4.80
4.78
2.54
2.50 dmso_d6
3.18
0.94
0.97
0.96
0.95
0.98
0.93
2.01
f1 (ppm)

Z220439492

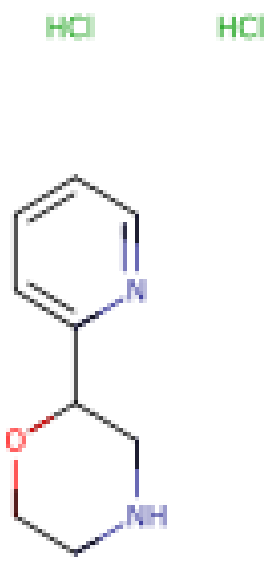

Mol Wt 237.13
Exact Mass 164.11

Error: Peaks not found!

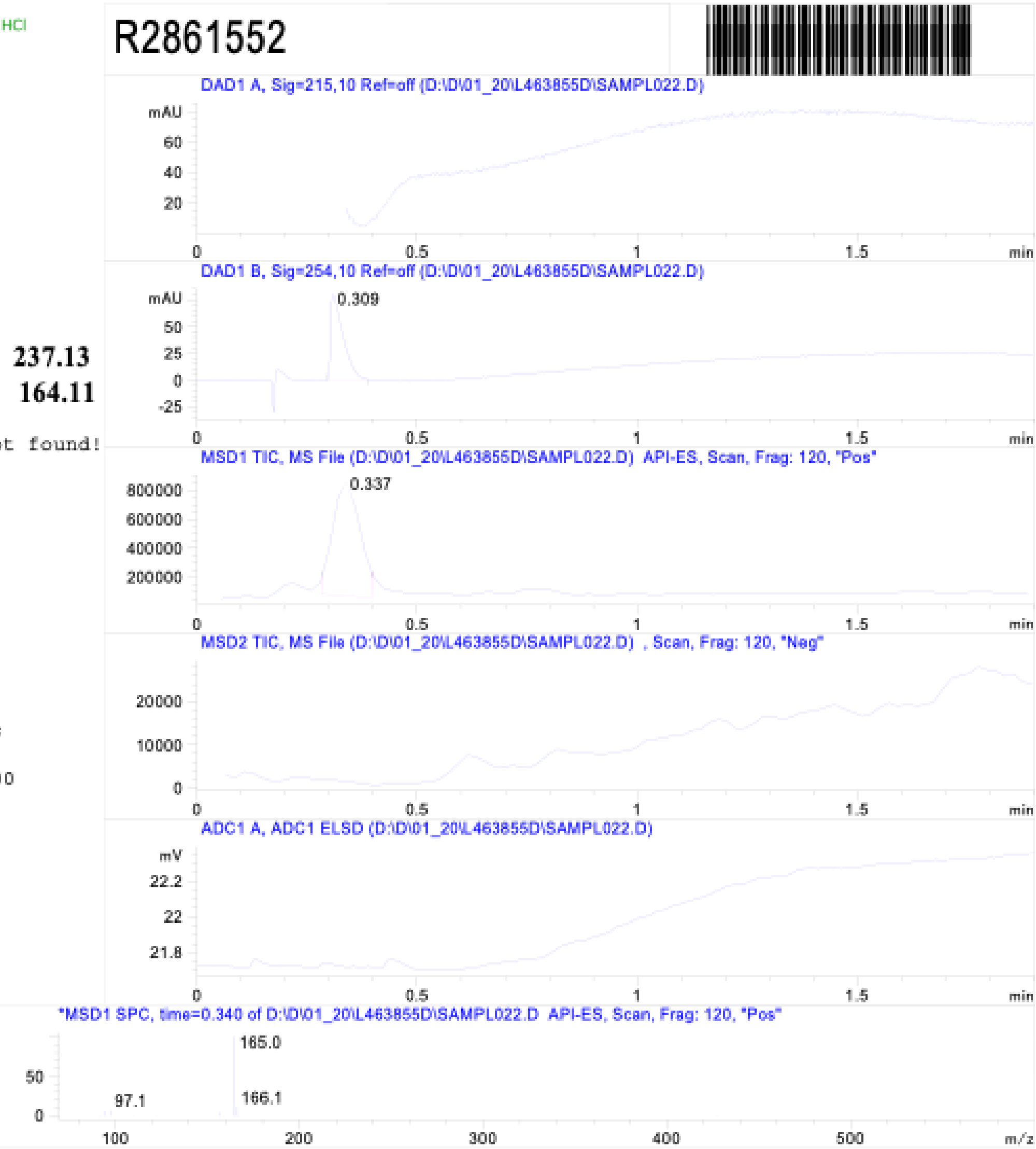

#	Time	Area%
--- DAD1 B ---		
1	0.309	100.00

RT 0.337

Inj.Date 1/20/2022 Y P2-C-02 -VL- Acq. Method C:\HPCHEM\-> ->

Z8987163207

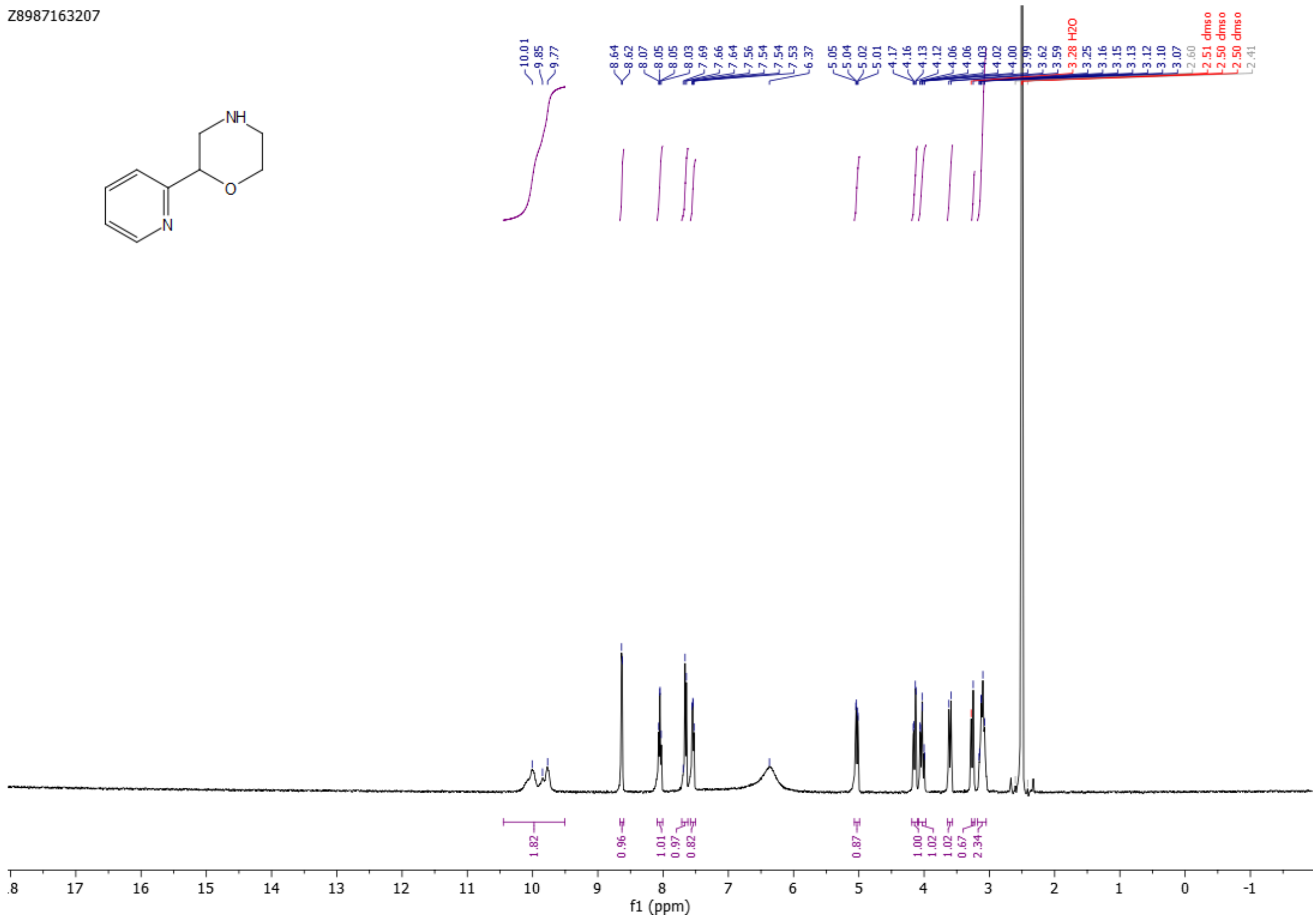

Z8987163207

MaxPeak: 100.00%
Ret_Time: 0.590 min

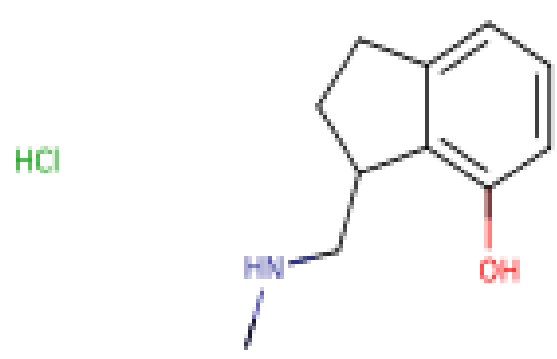

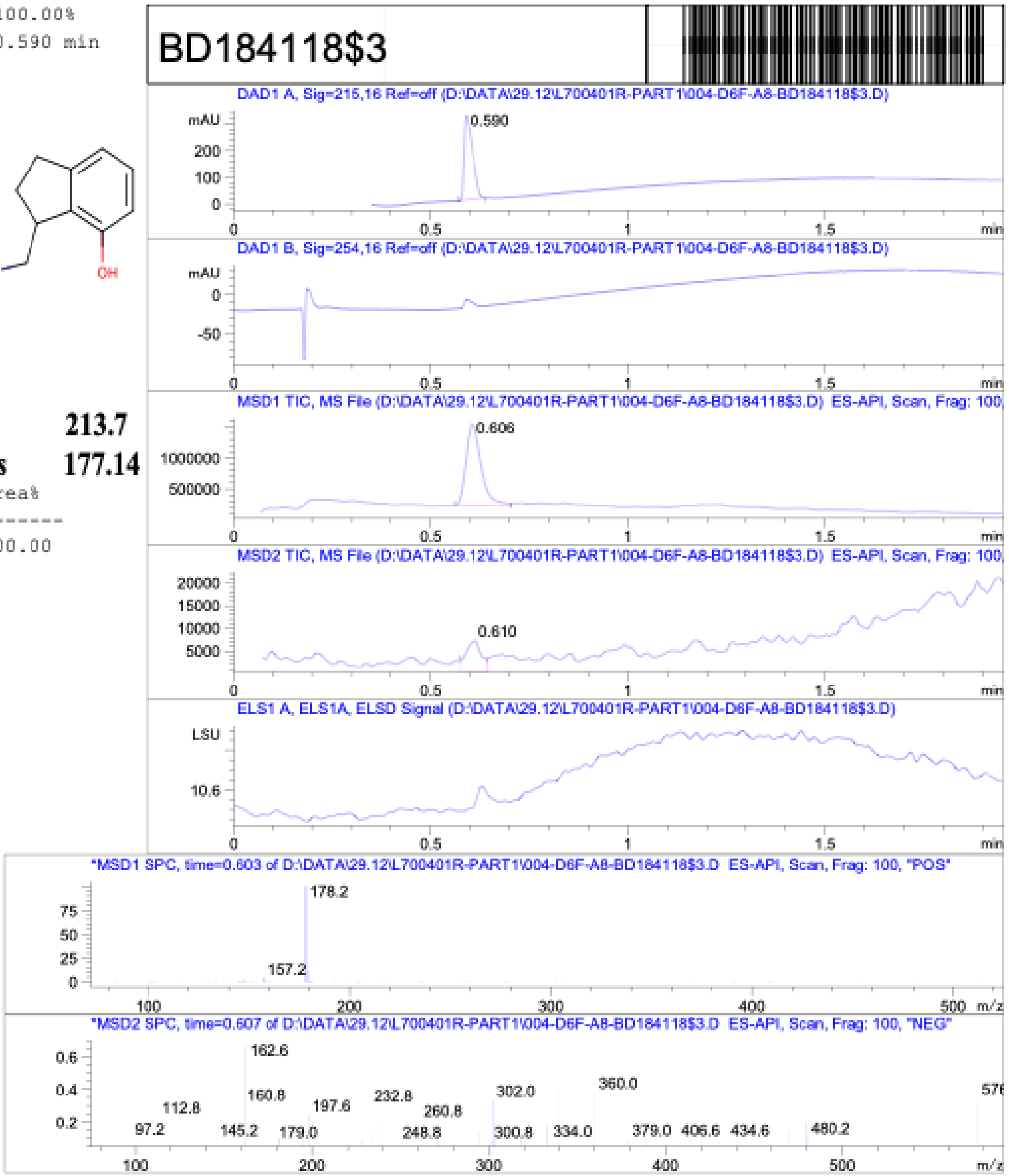

Mol Wt 213.7

Exact Mass 177.14

#	Time	Area%
1	0.590	100.00

RT 0.606

RT 0.610

Inj.Date 12/29/2023 M 13

Z2732542387

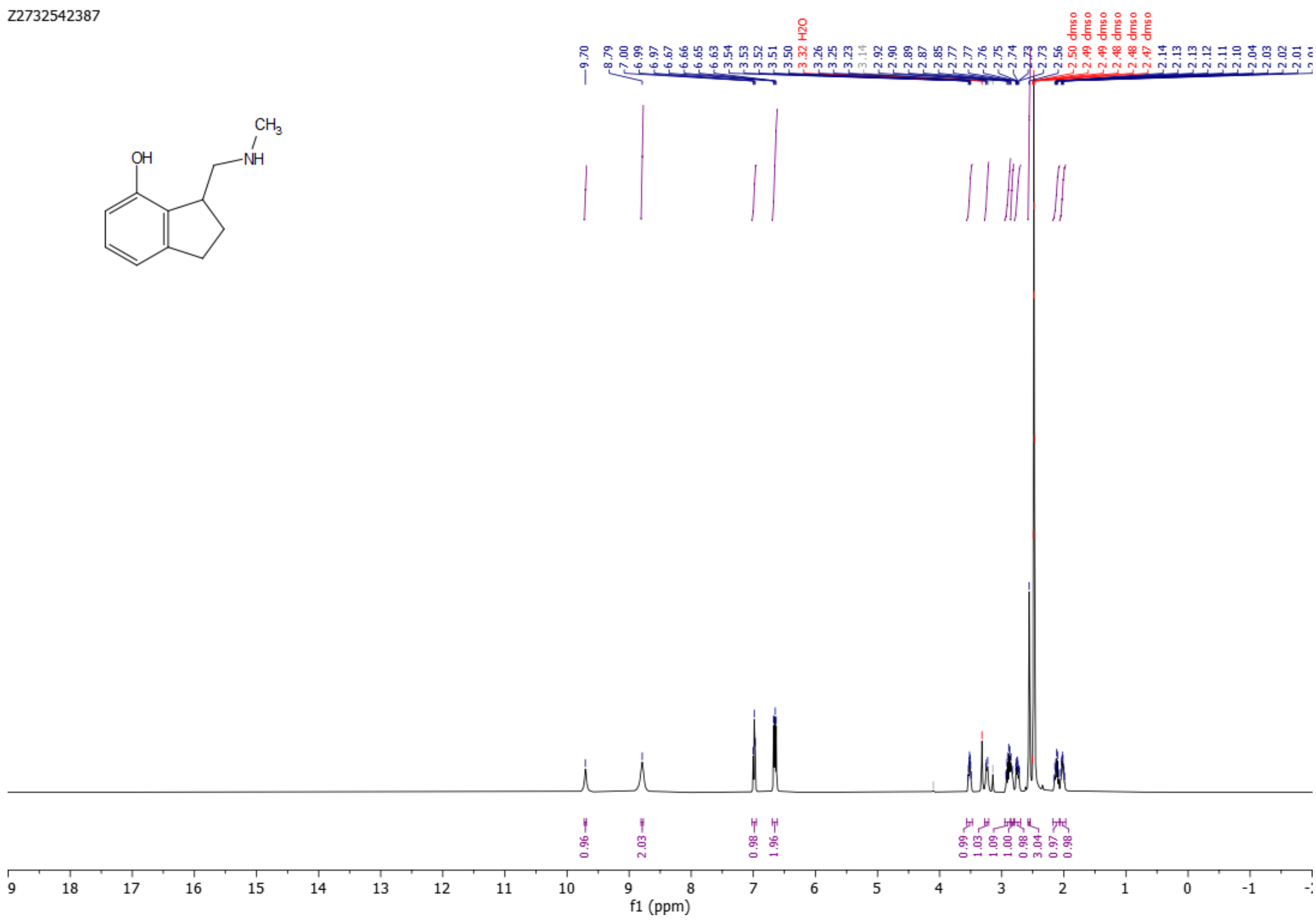

Z2732542387

MaxPeak: 98.30%
Ret_Time: 0.663 min

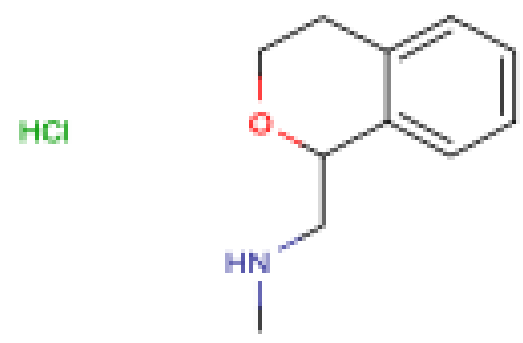

Mol Wt 213.7
Exact Mass 177.14

#	Time	Area%
1	0.611	1.70
2	0.663	98.30

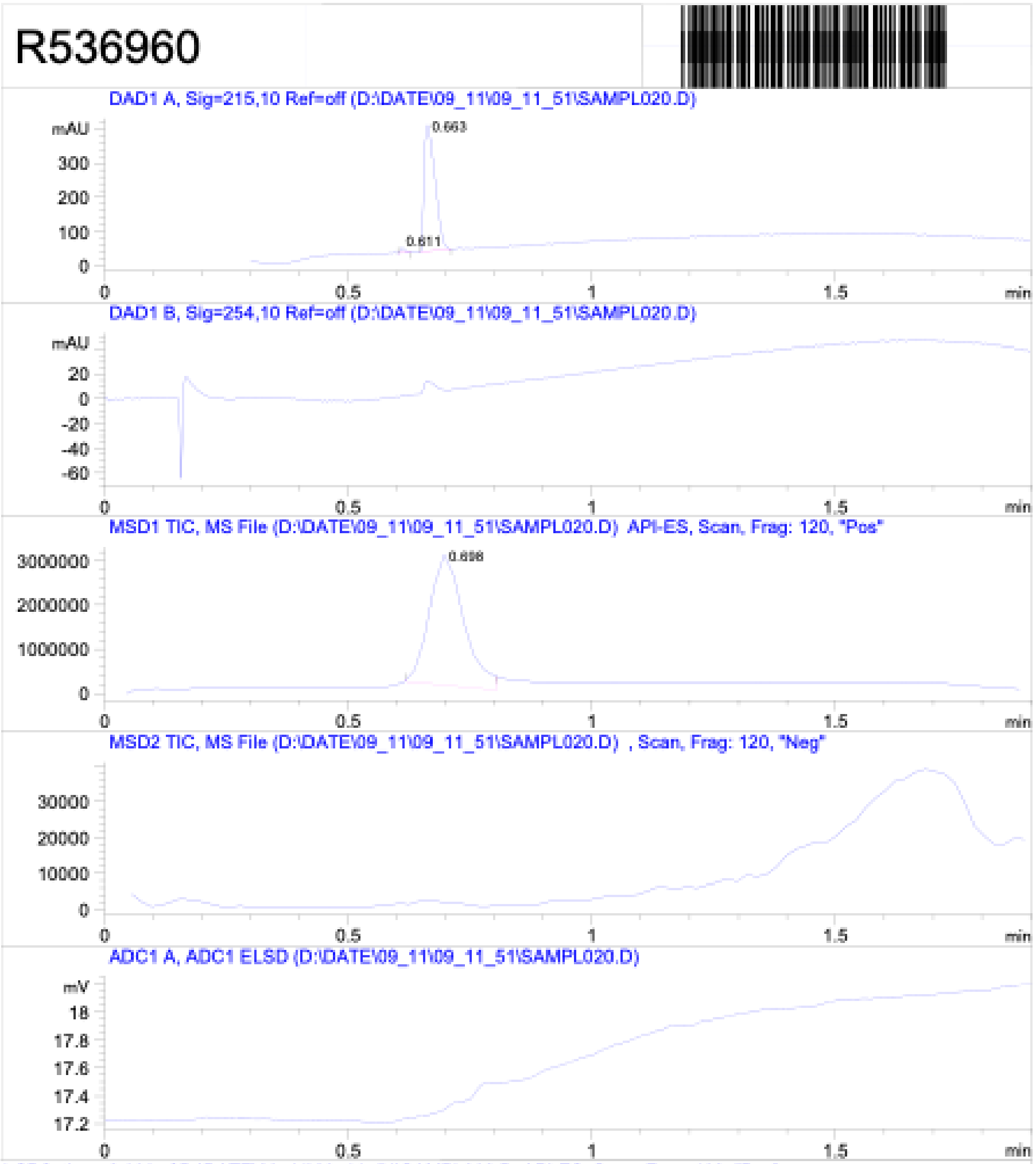

RT 0.698

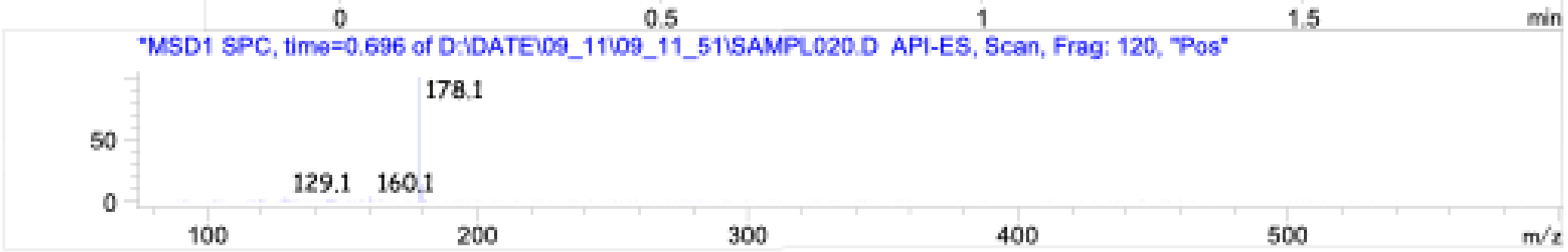

Inj.Date 9/12/2015 O P2-C-01 SL Acq. Method C:\HPCHEM\-> ->

Z337709402

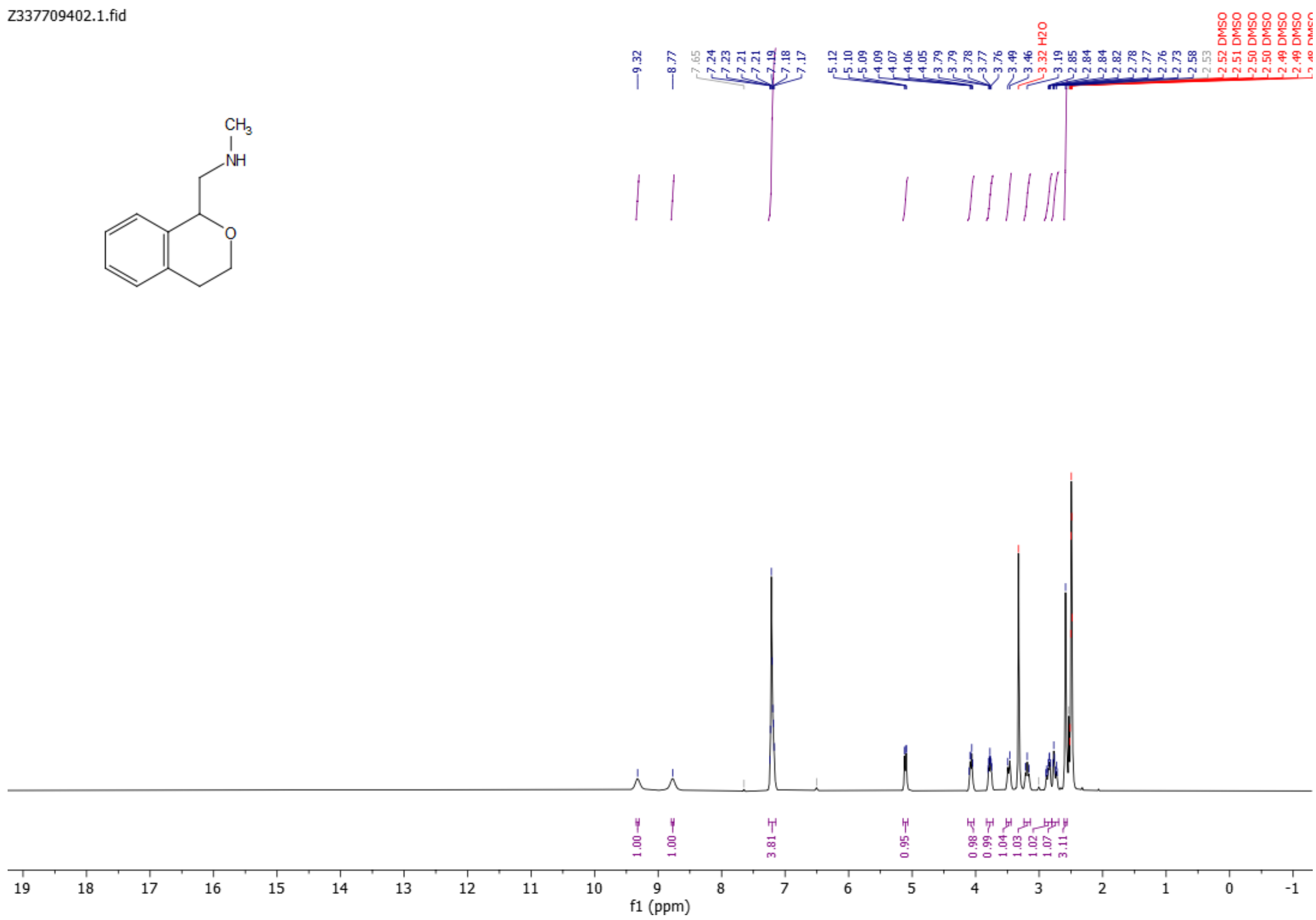
Z337709402.1.fid
CH3
NH
O
f1 (ppm)
1.00
1.00
3.81
0.95
0.98
0.99
1.04
1.03
1.02
1.07
3.11

Z337709402

MaxPeak: 100.00%
Ret_Time: 0.384 min

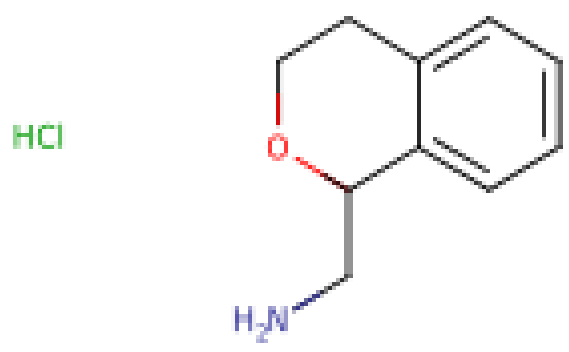

Mol Wt 199.68
Exact Mass 163.12

#	Time	Area%
1	0.384	100.00

RT 0.399

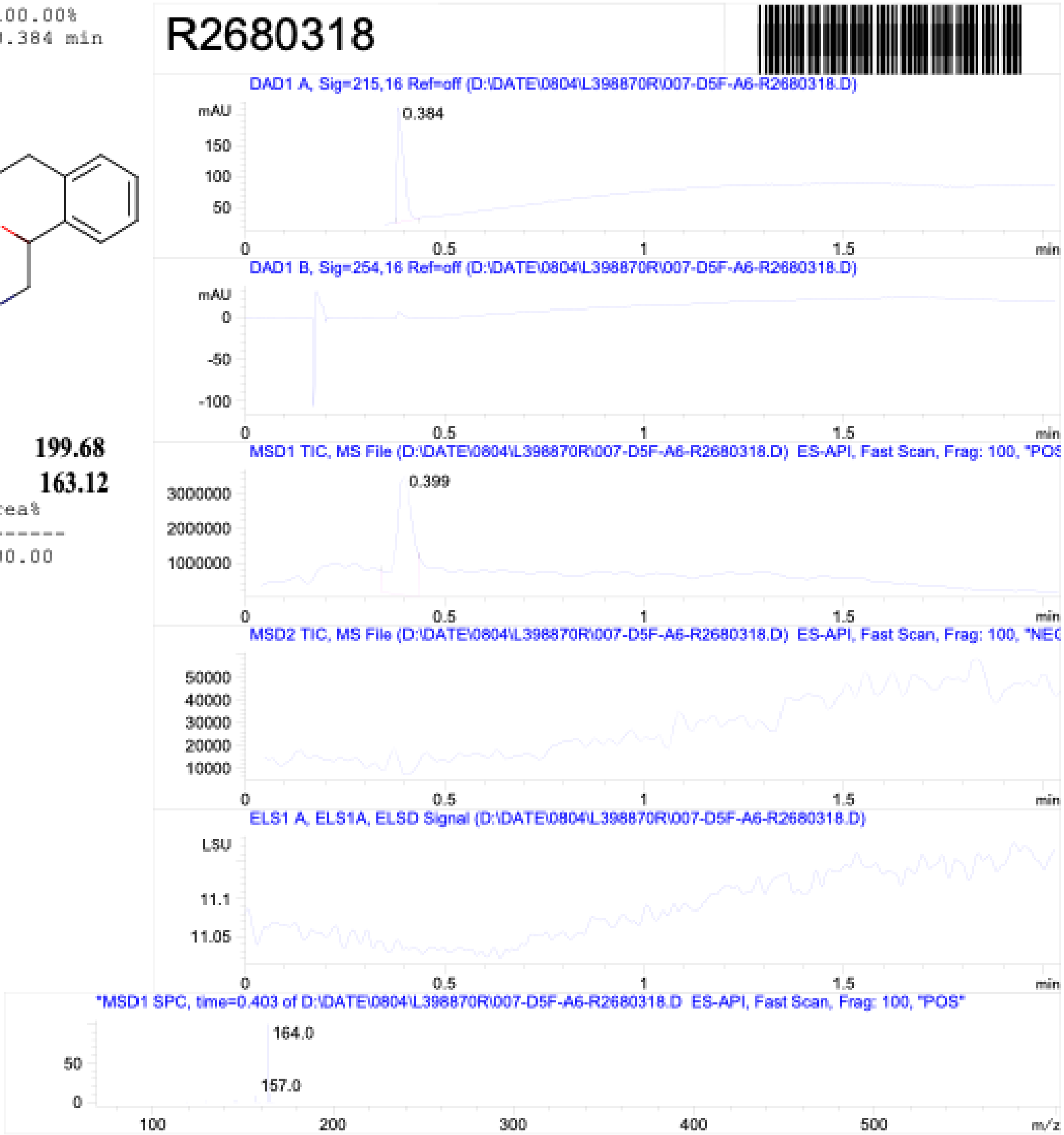

Inj.Date 8/4/2021 E Acq. Method C:\Users\ -> ->

Z337709400

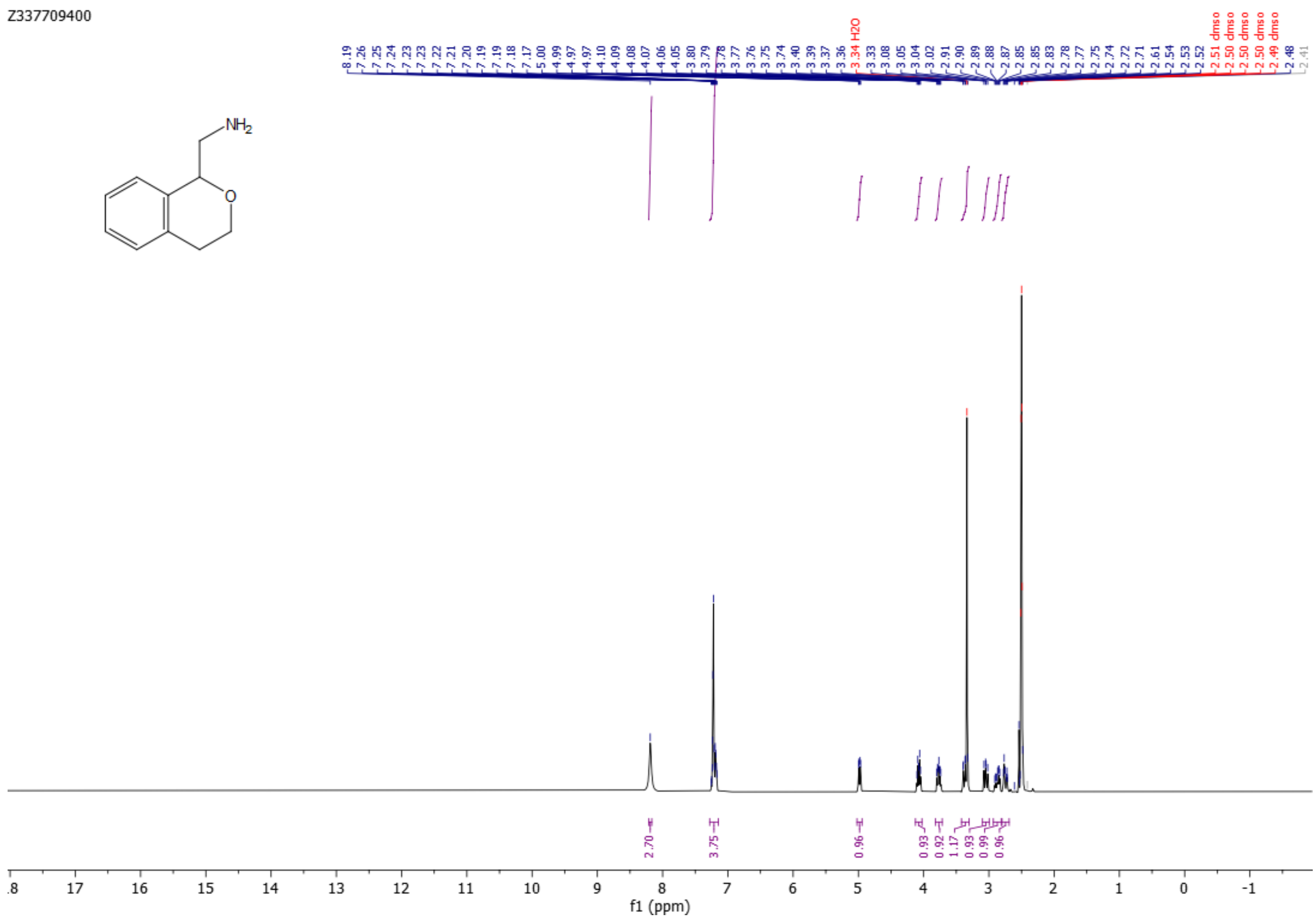

Z337709400

MaxPeak: 100.00%
Ret_Time: 0.850 min

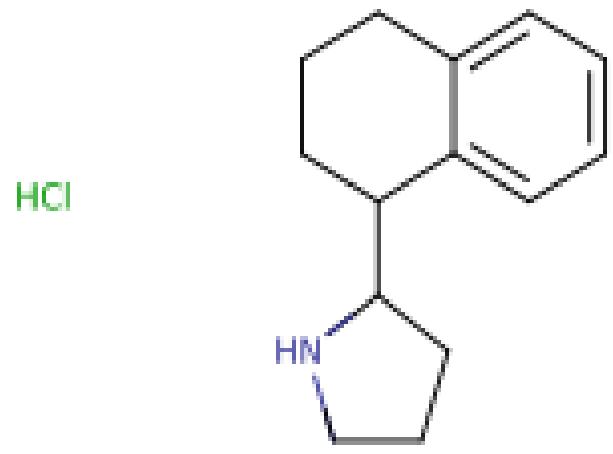

Mol Wt 237.77
Exact Mass 201.19

#	Time	Area%
1	0.850	100.00

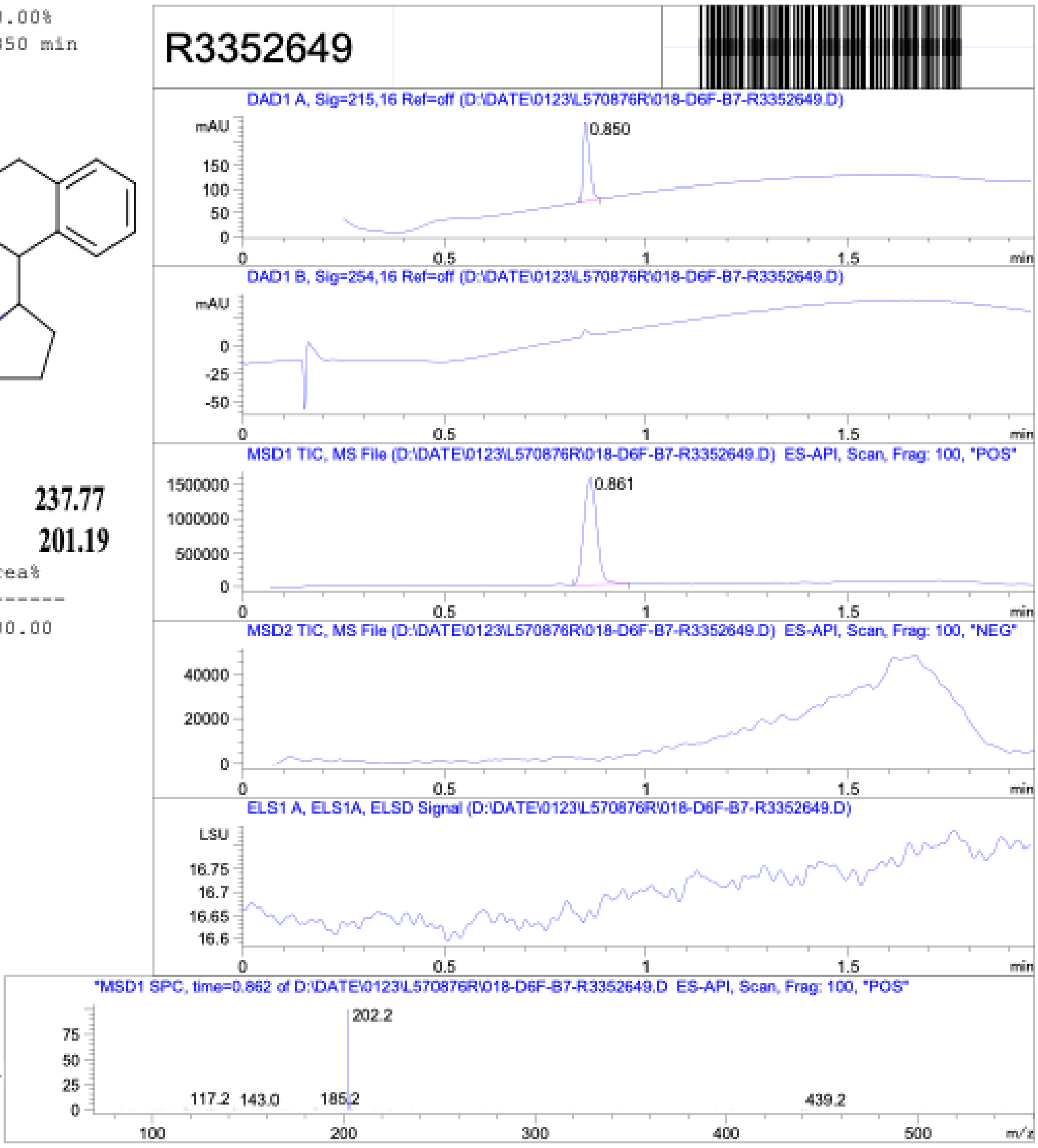

RT 0.861

Inj.Date 1/23/2023 CH -15- Acq. Method C:\Chem32\-> ->

Z995211376

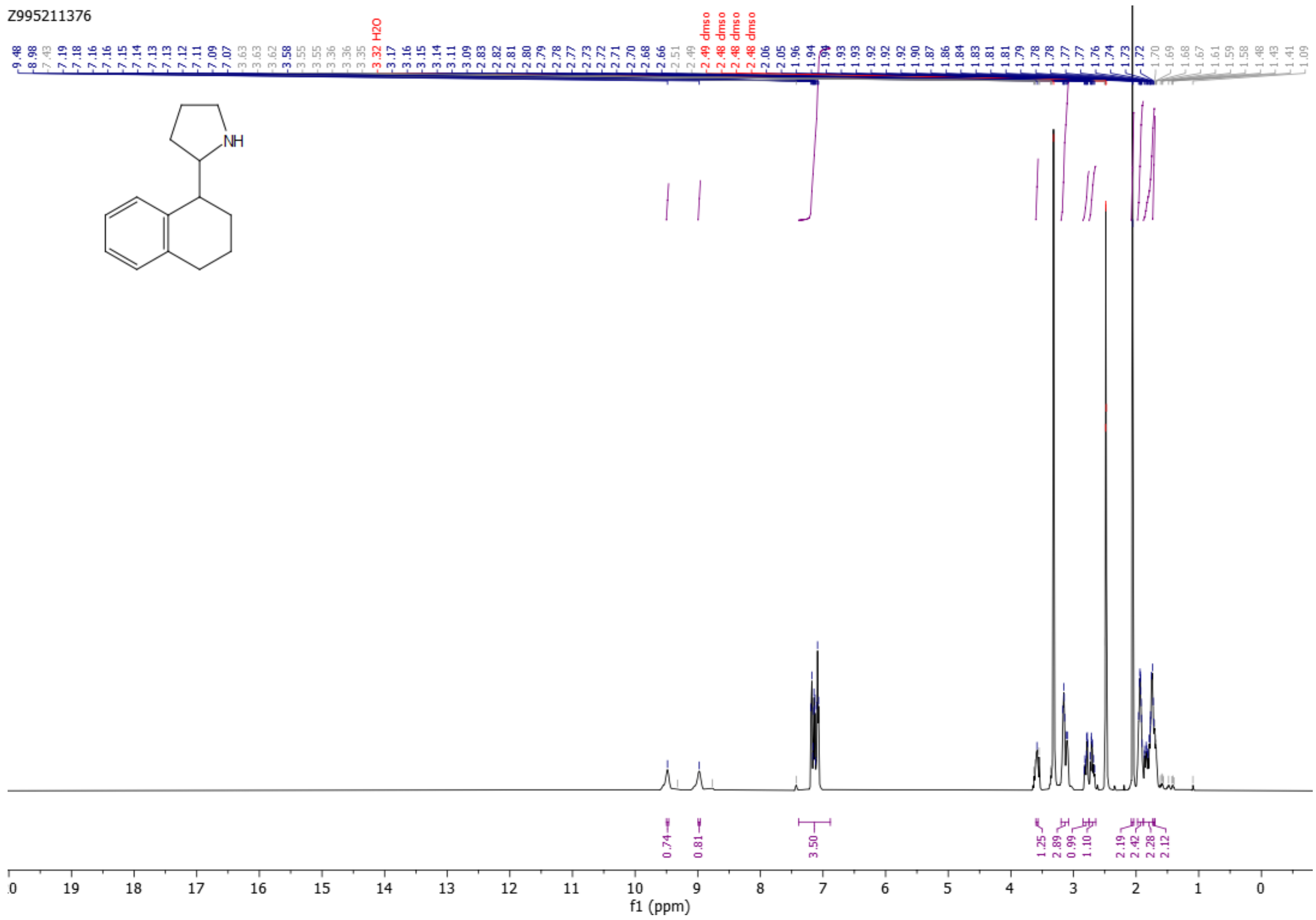
Z995211376
NH
0.74
0.81
3.50
1.25
2.89
0.99
1.10
2.19
2.42
2.28
2.12
f1 (ppm)

Z995211376

MaxPeak: 98.50%
Ret_Time: 0.558 min

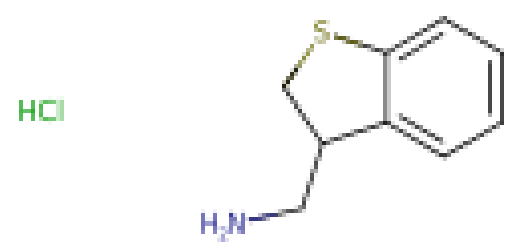

R3988191

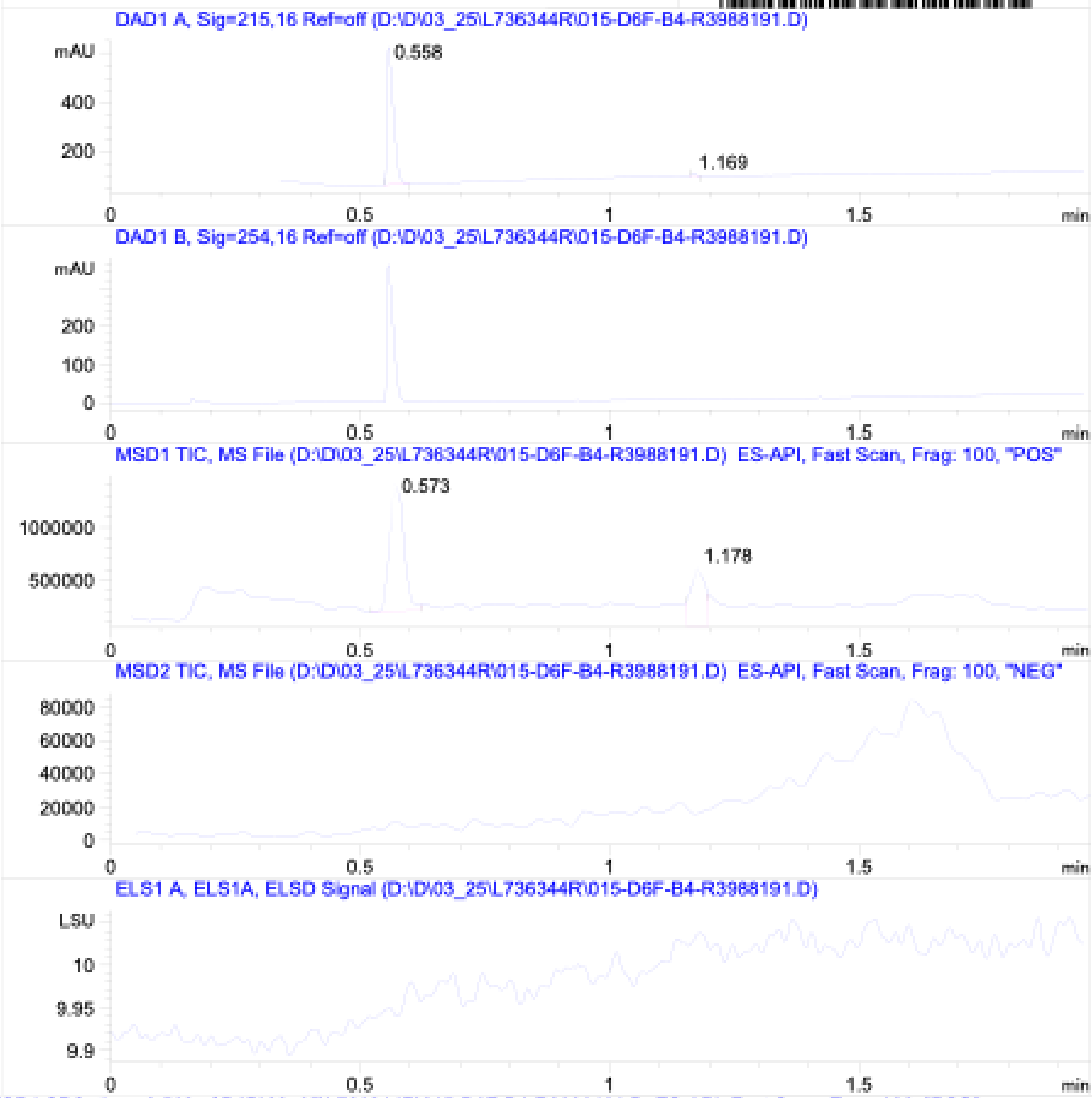

Mol Wt 201.72
Exact Mass 165.08

#	Time	Area%
1	0.558	98.50
2	1.169	1.50

RT 0.573

RT 1.178

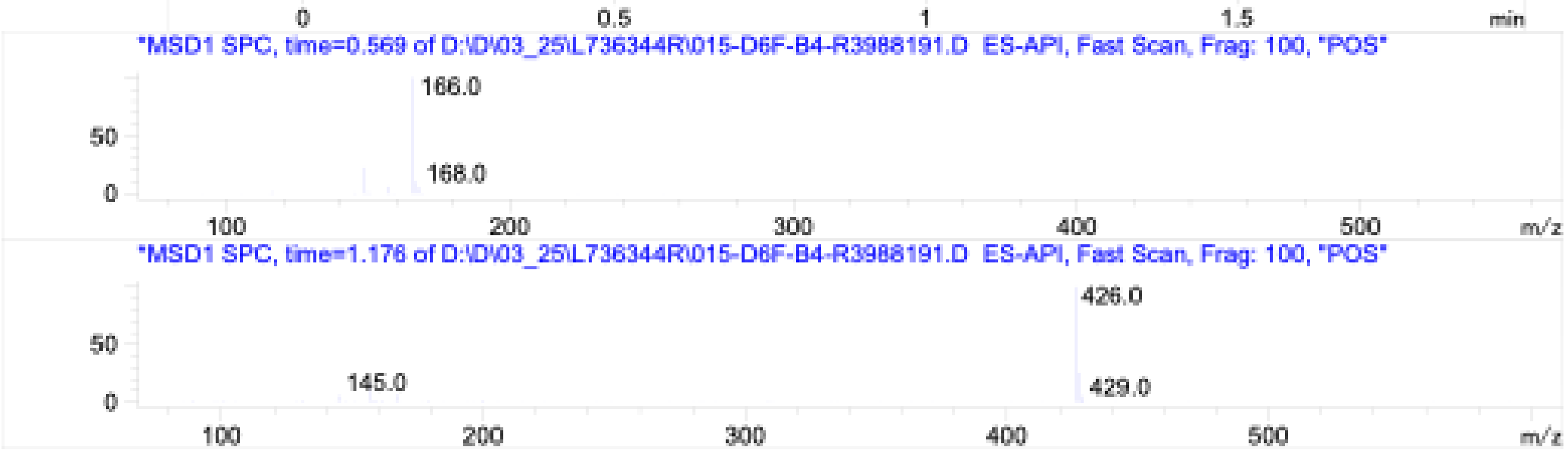

Inj.Date 3/25/2024 N
C:\Users\Public\Documents\ChemStation\1\Data\03_25\L736344R\SUPOR_30.M

EN300-47307925

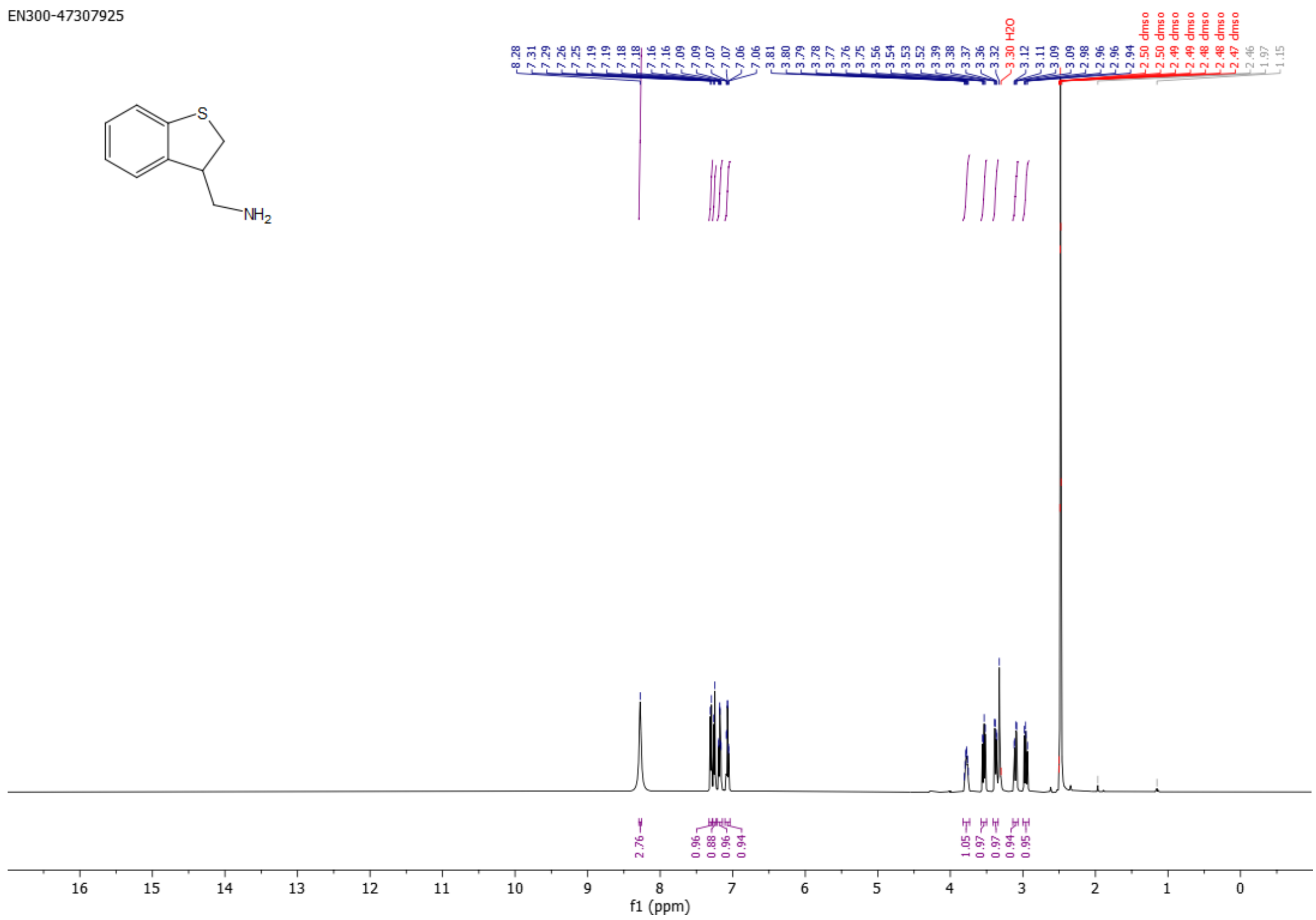

EN300-47307925

MaxPeak: 100.00%
Ret_Time: 0.669 min

HCl

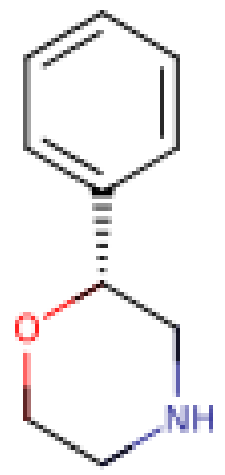

Mol Wt 199.68

Exact Mass 163.12

#	Time	Area%
1	0.669	100.00

R1634566

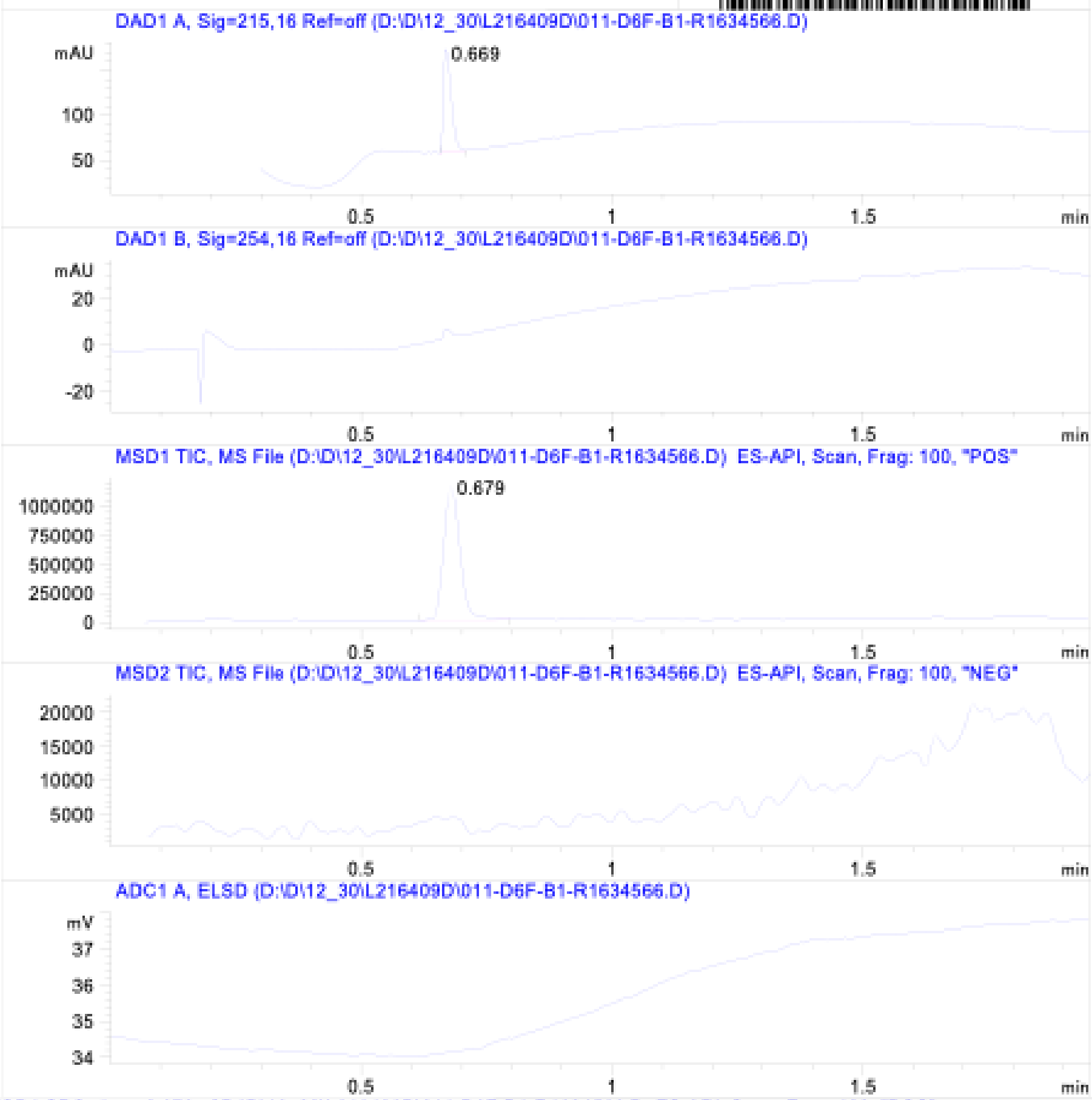

RT 0.679

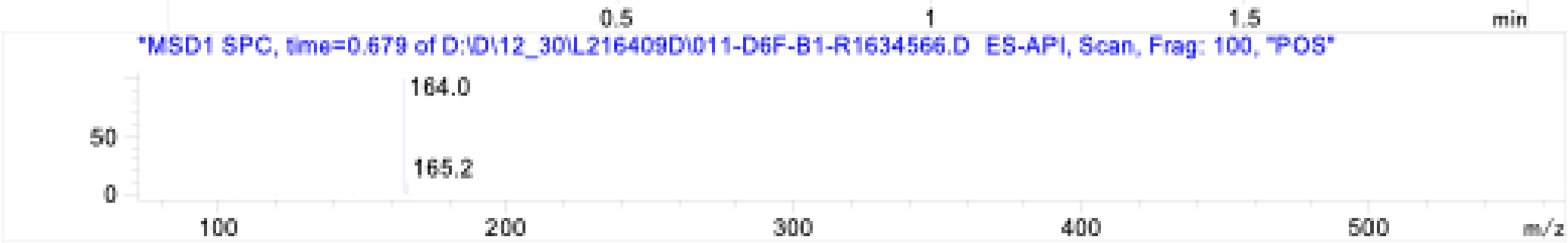

Inj.Date 12/30/2019 N <invalid> -8- Acq. Method C:\Chem32\-> ->

Z1255382255

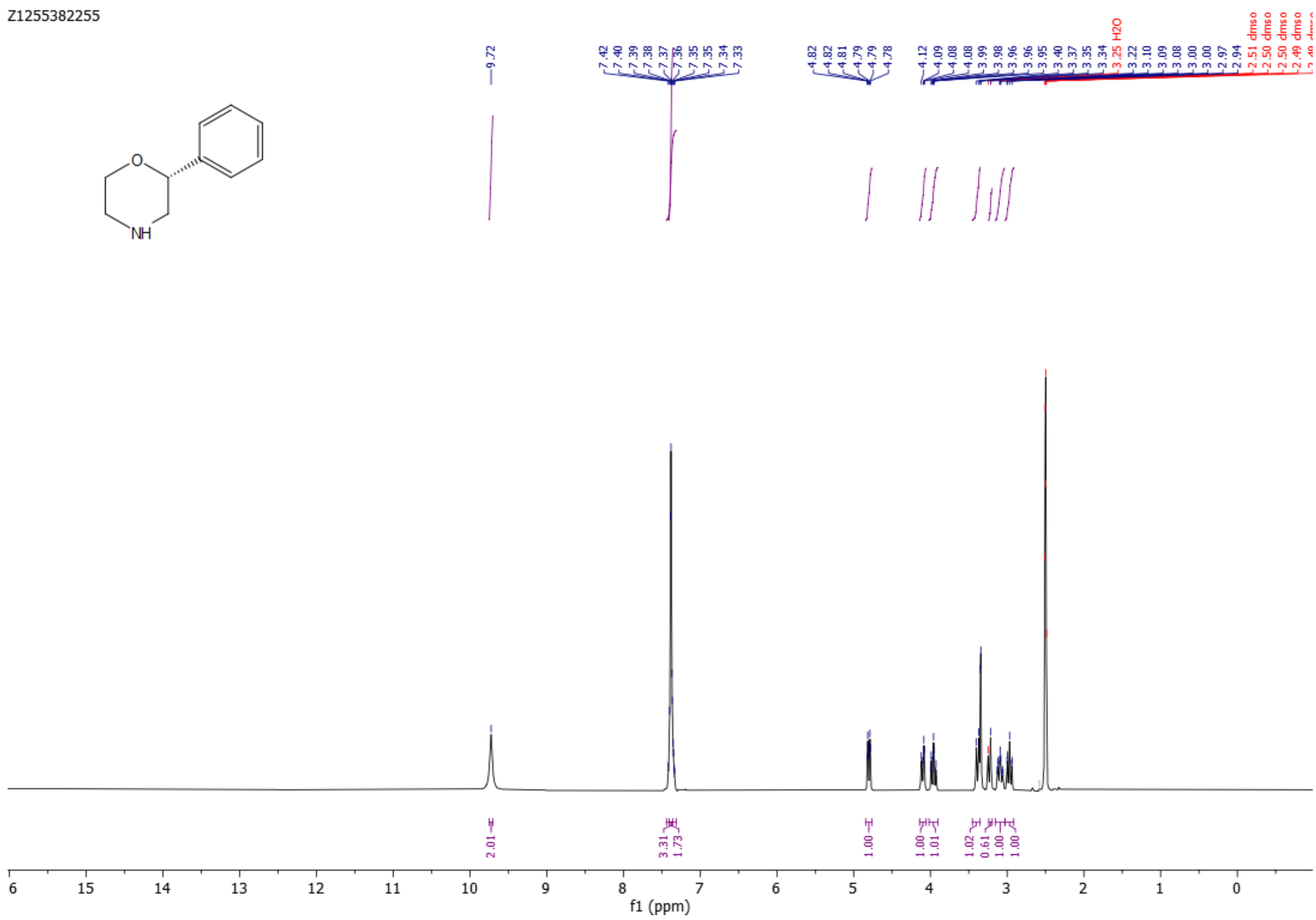

Z1255382255

MaxPeak: 100.00%
Ret_Time: 0.965 min

BF560332$1

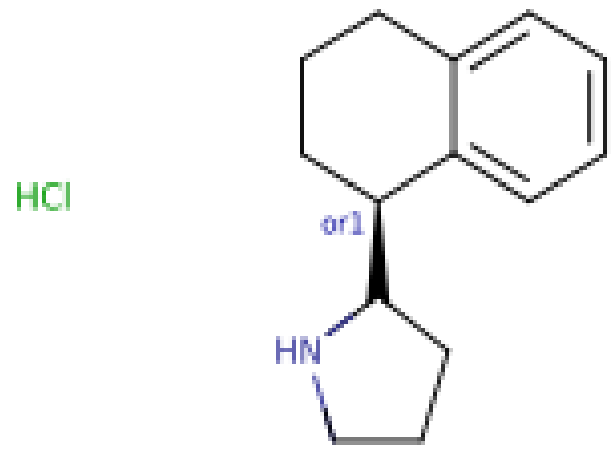

Mol Wt 237.77
Exact Mass 201.19

#	Time	Area%
1	0.965	100.00

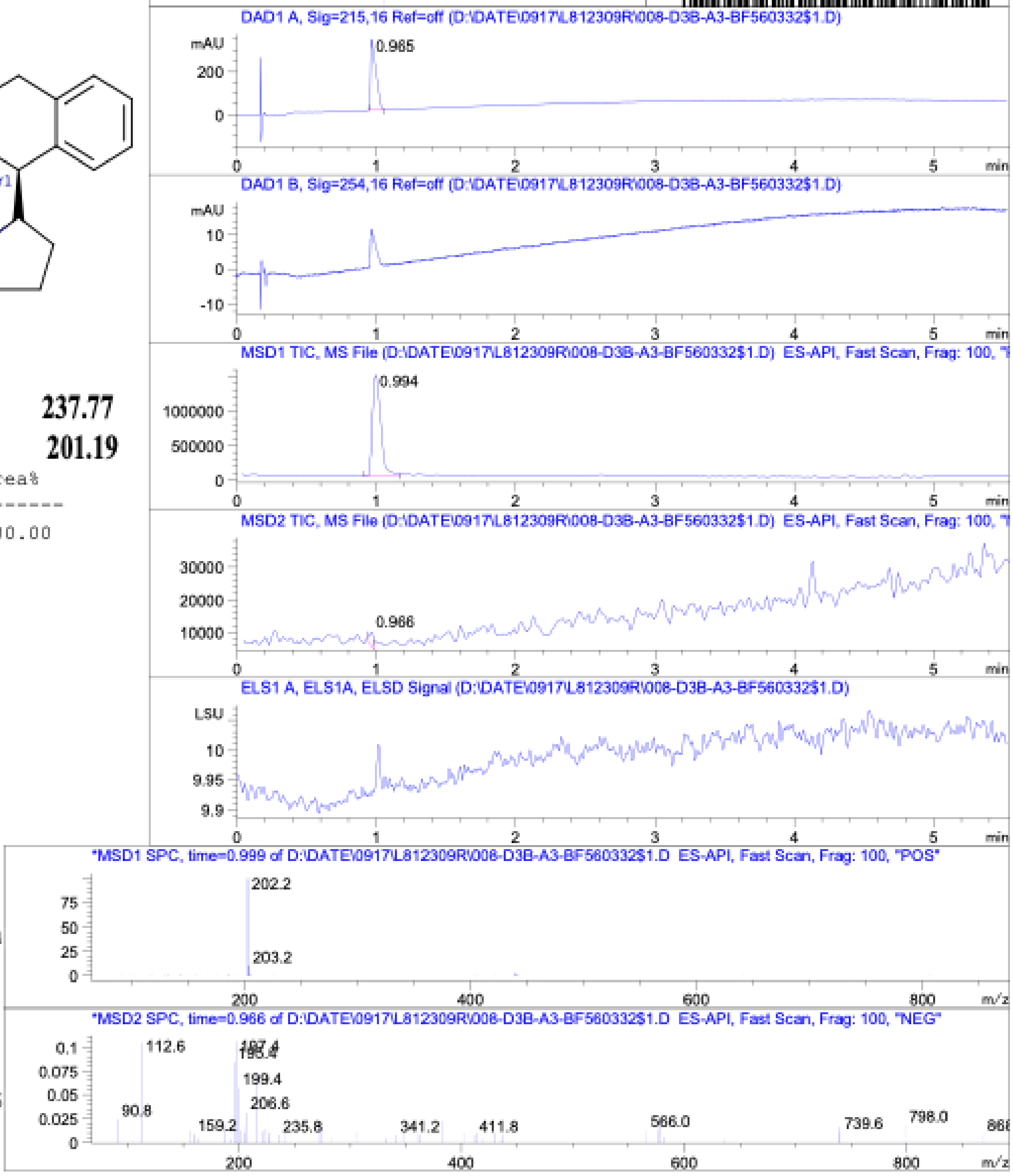

Inj.Date 9/17/2024 CH -19-
C:\Users\Public\Documents\ChemStation\1\Data\09_17\L812309R\UZRX45_3-5V.M

Z8987197482

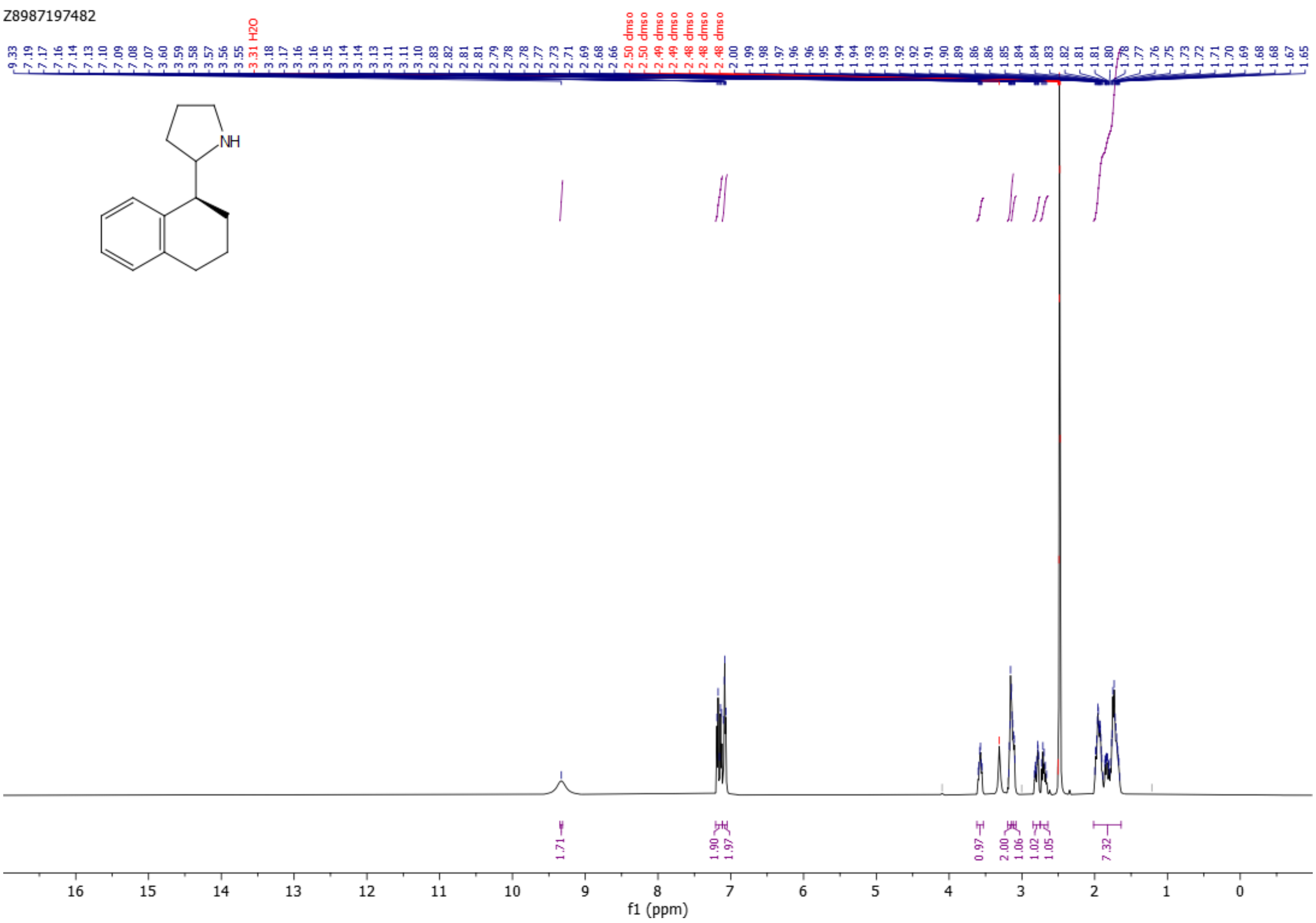

Z8987197482

MaxPeak: 98.73%
Ret_Time: 0.416 min

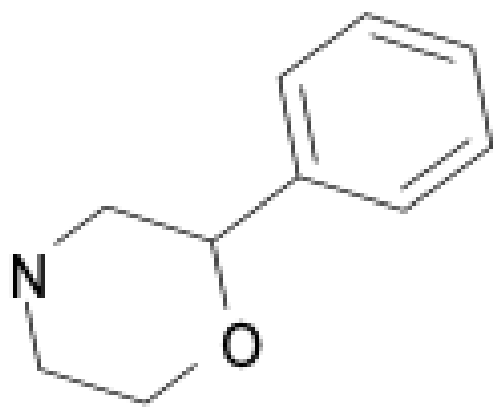

mw = 163.22

#	Time	Area%
1	0.416	98.73
2	0.994	1.27

RT 0.447

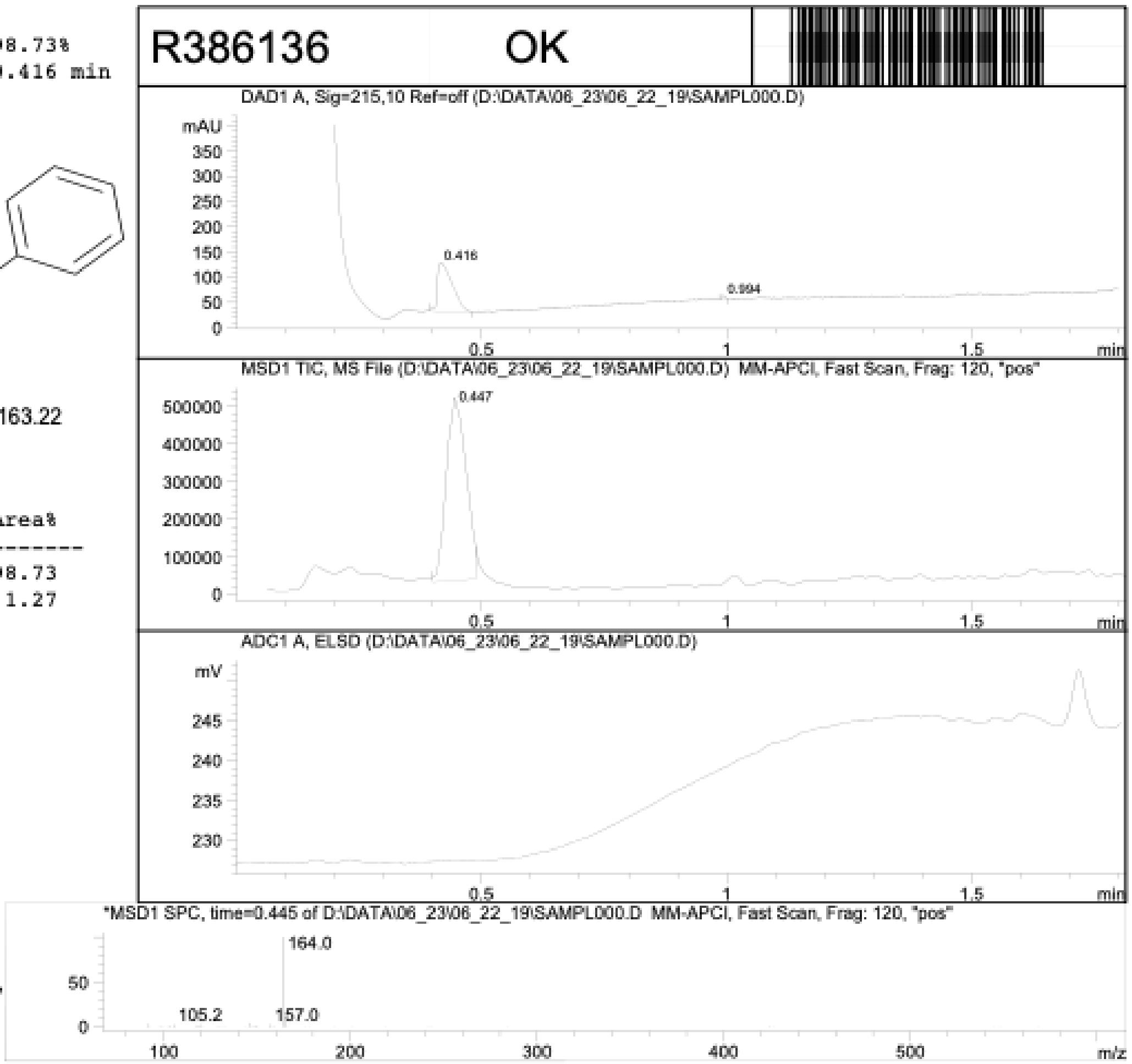

Inj.Date 6/22/2007 T P2-A-01 Acq. Method C:\Chem32\->

Z283821656

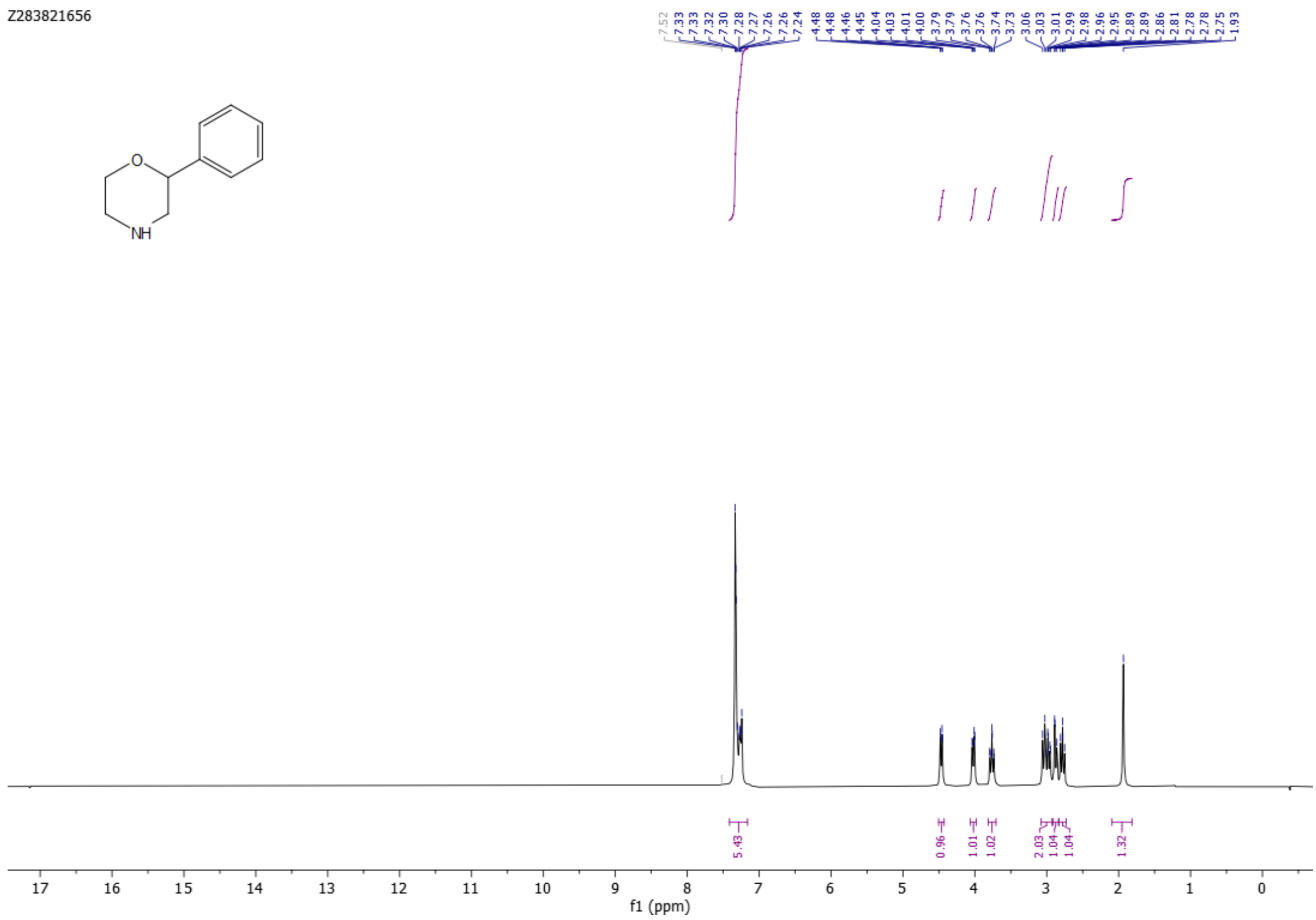

Z283821656
