# Supplementary material for: Structure based discovery of antipsychotic-like TAAR1 agonists
Source: Nat Commun. 2026 Sep 17;17:10902. doi: 10.1038/s41467-026-77484-2 (PMC13677718; doi:10.1038/s41467-026-77484-2)
Supplement: Supplementary file 2 [file 41467_2026_77484_MOESM2_ESM.pdf]

Corresponding author(s): Brian K. Shoichet, William C. Wetsel, H. Eric Xu

Last updated by author(s): Jun 15, 2026

# Reporting Summary

Nature Portfolio wishes to improve the reproducibility of the work that we publish. This form provides structure for consistency and transparency in reporting. For further information on Nature Portfolio policies, see our Editorial Policies and the Editorial Policy Checklist.

## Statistics

For all statistical analyses, confirm that the following items are present in the figure legend, table legend, main text, or Methods section.

| n/a | Confirmed | |
|---|---|---|
| ☐ | ☒ | The exact sample size (*n*) for each experimental group/condition, given as a discrete number and unit of measurement |
| ☐ | ☒ | A statement on whether measurements were taken from distinct samples or whether the same sample was measured repeatedly |
| ☐ | ☒ | The statistical test(s) used AND whether they are one- or two-sided<br>*Only common tests should be described solely by name; describe more complex techniques in the Methods section.* |
| ☒ | ☐ | A description of all covariates tested |
| ☐ | ☒ | A description of any assumptions or corrections, such as tests of normality and adjustment for multiple comparisons |
| ☐ | ☒ | A full description of the statistical parameters including central tendency (e.g. means) or other basic estimates (e.g. regression coefficient) AND variation (e.g. standard deviation) or associated estimates of uncertainty (e.g. confidence intervals) |
| ☐ | ☒ | For null hypothesis testing, the test statistic (e.g. *F*, *t*, *r*) with confidence intervals, effect sizes, degrees of freedom and *P* value noted<br>*Give P values as exact values whenever suitable.* |
| ☒ | ☐ | For Bayesian analysis, information on the choice of priors and Markov chain Monte Carlo settings |
| ☒ | ☐ | For hierarchical and complex designs, identification of the appropriate level for tests and full reporting of outcomes |
| ☒ | ☐ | Estimates of effect sizes (e.g. Cohen's *d*, Pearson's *r*), indicating how they were calculated |

*Our web collection on statistics for biologists contains articles on many of the points above.*

## Software and code

Policy information about availability of computer code

| | |
|---|---|
| Data collection | Docking was done using DOCK3.7 and DOCK3.8. CryoEM Data was collected automatically on a Titan Krios (FEI) using Falcon 4. |
| Data analysis | PyMOL v2.5.5<br>UCSF Chimera 1.14<br>GraphPad Prism 10.4.1<br>Schrodinger 2022 Maestro<br>MotionCor2<br>CryoSparc v3.2.0<br>UCSF ChimeraX v1.5<br>Coot v0.9.6<br>Phenix elbow |

For manuscripts utilizing custom algorithms or software that are central to the research but not yet described in published literature, software must be made available to editors and reviewers. We strongly encourage code deposition in a community repository (e.g. GitHub). See the Nature Portfolio guidelines for submitting code & software for further information.

## Data

Policy information about availability of data

All manuscripts must include a data availability statement. This statement should provide the following information, where applicable:

- Accession codes, unique identifiers, or web links for publicly available datasets
- A description of any restrictions on data availability
- For clinical datasets or third party data, please ensure that the statement adheres to our policy

*Provide your data availability statement here.*

## Research involving human participants, their data, or biological material

Policy information about studies with human participants or human data. See also policy information about sex, gender (identity/presentation), and sexual orientation and race, ethnicity and racism.

| | |
|---|---|
| Reporting on sex and gender | NA |
| Reporting on race, ethnicity, or other socially relevant groupings | NA |
| Population characteristics | NA |
| Recruitment | NA |
| Ethics oversight | NA |

Note that full information on the approval of the study protocol must also be provided in the manuscript.

# Field-specific reporting

Please select the one below that is the best fit for your research. If you are not sure, read the appropriate sections before making your selection.

[x] Life sciences [ ] Behavioural & social sciences [ ] Ecological, evolutionary & environmental sciences

For a reference copy of the document with all sections, see nature.com/documents/nr-reporting-summary-flat.pdf

# Life sciences study design

All studies must disclose on these points even when the disclosure is negative.

| | |
|---|---|
| Sample size | We did not perform sample-size calculations. We modeled our sample sizes for behavioral studies on previous studies using a similar approach to our own, which have been demonstrated to be capable of detecting significant changes (Pogorelov, Vladimir M., et al. 2017). Sample sizes of cryo-EM data were determined by availability of microscopetime. For functional assays, three biologically independent experiments (n=3) were performed as depicted in related Figure legends. |
| Data exclusions | No data were excluded. |
| Replication | All replications were successful at least twice. |
| Randomization | The animals were randomly assigned to the treatment group and control group. For behavioral experiments, animals were initially placed into one cage and allowed to free run for a few minutes. Next, each animal was randomly picked up, injected with the drug or vehicle control. This randomization protocol was consistently applied across all behavioral experiments. |
| Blinding | For all behavioral tests, the experimenter was always blind to treatment. |

# Reporting for specific materials, systems and methods

We require information from authors about some types of materials, experimental systems and methods used in many studies. Here, indicate whether each material, system or method listed is relevant to your study. If you are not sure if a list item applies to your research, read the appropriate section before selecting a response.

## Materials & experimental systems

| n/a | Involved in the study |
| --- | --- |
| ☐ | ☒ Antibodies |
| ☐ | ☒ Eukaryotic cell lines |
| ☒ | ☐ Palaeontology and archaeology |
| ☐ | ☒ Animals and other organisms |
| ☒ | ☐ Clinical data |
| ☒ | ☐ Dual use research of concern |
| ☒ | ☐ Plants |

## Methods

| n/a | Involved in the study |
| --- | --- |
| ☒ | ☐ ChIP-seq |
| ☒ | ☐ Flow cytometry |
| ☒ | ☐ MRI-based neuroimaging |

# Antibodies

| | |
| --- | --- |
| Antibodies used | Nb35 was used to stabilize the TAAR1-Gs complex. The sequence of Nb35 was reported in https://doi.org/10.1038/nature10361 |
| Validation | Nb35 was produced in Brian Kobilka's lab and was not used for probing purposes, therefore no validation is required. |

# Eukaryotic cell lines

Policy information about cell lines and Sex and Gender in Research

| | |
| --- | --- |
| Cell line source(s) | Hi5 insect cells (Invitrogen), Sf9 cells were obtained from Expression Systems (Cat 94-001S) and HEK293 cells were purchased from the American Type Culture Collection (ATCC, CRL-1573). |
| Authentication | Hi5 cell lines are maintained by the supplier. No additional authentication was performed by the authors of this study.<br>HEK293 cell lines are maintained by the supplier. No additional authentication was performed by the authors of this study.<br>Sf9 cells lines are maintained by the supplier. No additional authentication was performed by the authors of this study. |
| Mycoplasma contamination | Cell lines are tested by manufacturer for contamination, but not were not further tested by the authors of this study. |
| Commonly misidentified lines (See ICLAC register) | No commonly misidentified lines were used in this study. |

# Animals and other research organisms

Policy information about studies involving animals; ARRIVE guidelines recommended for reporting animal research, and Sex and Gender in Research

| | |
| --- | --- |
| Laboratory animals | Adult male and female C57BL/6J mice (90 days old; #000664; The Jackson Laboratory, Bar Harbor, ME) were used in these experiments. |
| Wild animals | No wild animals were used in this study. |
| Reporting on sex | Both male and female mice were included in the study. Sex was incorporated as a biological variable in the statistical analyses; however, the study was not specifically powered to detect sex-specific effects. Although limited sex-dependent effects were observed in some measures, no consistent sex-dependent differences were detected across PPI-related endpoints overall. Therefore, data from male and female mice were combined for presentation. |
| Field-collected samples | No field collected samples were used in this study |
| Ethics oversight | All PPI studies were conducted under protocols approved by the Duke University Institutional Animal Care and Use Committee (IACUC protocol no. A069-23-03), and all procedures were performed in accordance with ARRIVE guidelines.<br>Pharmacokinetic experiments were performed by by Bienta (Enamine Biology Services) in in accordance with Enamine pharmacokinetic study protocols and Institutional Animal Care and Use Guidelines. |

Note that full information on the approval of the study protocol must also be provided in the manuscript.

## Plants

| | |
|---|---|
| Seed stocks | NA |
| Novel plant genotypes | NA |
| Authentication | NA |
