# Supplementary material for: Structure based discovery of antipsychotic-like TAAR1 agonists
Source: Nat Commun. 2026 Sep 17;17:10902. doi: 10.1038/s41467-026-77484-2 (PMC13677718; doi:10.1038/s41467-026-77484-2)
Supplement: Supplementary file 3 [file 41467_2026_77484_MOESM3_ESM.pdf]

nature portfolio

Peer Review File

Structure based discovery of antipsychotic-like TAAR1 agonists

Corresponding Author: Professor Brian Shoichet

This file contains all reviewer reports in order by version, followed by all author rebuttals in order by version.

Version 0:

Reviewer comments:

Reviewer #1

(Remarks to the Author)
This manuscript is a strong addition to the success of virtual docking screens for aminergic GPCR small molecule drug discovery by extending to the existing literature (DOI: 10.1016/j.cell.2023.10.014 , DOI: 10.1126/sciadv.adn1524, DOI: 10.1021/acs.jmedchem.5c00432, DOI: 10.1021/acs.jmedchem.4c00195) on virtual screening to identify TAAR1 compounds. The authors use recently available TAAR1 structures for docking screens to identify novel TAAR1 agonists and optimize the compounds in accordance with their expected behavior observed with SAR. Cryo-EM was used to confirm their docking predictions. They follow up the optimization with animal work and show strong evidence of antipsychotic-like effects, but no catalepy, in mouse models. The data and interpretations are of sound quality. However, some clarifications and additions to the paper would increase its impact.

Specific comments:
(1) From my understanding of the methods, the cAMP GloSensor assay and TAAR1 construct was transfected and expressed in cells incubated for 24h in 10% FBS containing media. While the cells were later transferred to another medium to run the assay, despite a brief washout period (1h), the presence of monoamines in FBS could likely contribute to receptor sensitization and affect the screening performance and subsequent follow-up studies. The authors should clarify if that was the case or whether dialyzed FBS was used, and if not, why not.

(2) I do not see statistics related to biological or technical replicates, nor errors for the cell experiments anywhere in the figure legends. This information is essential for evaluating the reported pharmacological values. The legends for Fig 2 should be extended to indicate which receptor is being shown in the concentration-response curves for clarity.

(3) You state seven known antagonists but I’m also not aware of more than one independently validated, active antagonist at the human TAAR1, RTI. Can you please spell the identity of the seven antagonists that you are referring to? Which antagonist was most enriched in the inactive state model?

(4) A clarification as to how the inactive model was generated would be helpful here (line 144).

(5) A major point of ambiguity is the in vivo mechanism of action. The compounds used for behavioral testing were shown to be dual 5-HT1A and TAAR1 agonists, with the authors also noting the effect of 5-HT1A activation for antipsychotic efficacy. However, the paper attributes the behavioral benefits primarily to TAAR1 agonism. This presents a mixed message, and the authors should clarify whether their optimization goal is a selective TAAR1 agonist or if this specific TAAR1/5-HT1A polypharmacology profile is the intended end goal.

(6) Following this concern, it would be very reassuring to show TAAR1 dependence of the antipsychotic-like effects exerted by the compounds in vivo. A commercially available antagonist (RTI-7470-44) would be useful here. Dissection of the 5-HT1A receptor contribution would be informative.

(7) Owing to the known promiscuity of TAAR1 agonists, it would also be useful to know the potency or activity of the compounds at other relevant other aminergic receptors which could contribute to the observed in vivo behavioral effects. The probable lack of D2 occupancy due to the catalepsy test is noted.

(8) The behavioral comparison to ulotaront without a heads-on comparison should not be made.

Minor comments:
• The primary statistics in Supp. Table 3 appear to incorrectly note Fig 5 as Fig 4
• -Gs should be referred to as Gαs throughout the manuscript
• There are typos in Refs 62 and 71

Reviewer #2

(Remarks to the Author)
I co-reviewed this manuscript with one of the reviewers who provided the listed reports. This is part of the Nature Communications initiative to facilitate training in peer review and to provide appropriate recognition for Early Career Researchers who co-review manuscripts.

Reviewer #3

(Remarks to the Author)
The manuscript reports a successful structure-based discovery and optimization campaign for novel TAAR1 agonists, which is a significant contribution to the field of neuroscience and drug discovery. The work not only delivers new ligands but also provides crucial methodological and structural insights for future virtual screening campaigns targeting TAAR1. I strongly recommend the publication of the manuscript, below just minor comments/suggestions:
1. The novelty of the identified ligands based on a Tc cutoff do not provide specificity regarding the closest known active ligand - I would suggest to report the similarity the most chemically similar known TAAR1 agonist available in public databases or literature for identified ligands. Consider adding a figure (or Extended Data figure) that visually compares the molecular scaffolds of the two or three most potent leads with the structures of ulotaront and/or known ligands, allowing readers to readily appreciate the chemical differences.
2. The manuscript touches upon the critical observation that docking against the active state (Cryo-EM) yielded an exclusive set of agonists, while the use of an inactive state model may have yielded different results. Please explain the use of Boltz-2 for the inactive state modeling. Would it be possible to provide also the pdb structure of the Boltz-2 model?
3. AF2 is introduced only in comparison with the newly released cryoEM structure. Why was not used for the agonists/antagonists comparison? A comparison between the active-state structure (8W8A and new one) and the AF2 and Boltz-2 models could be helpful to summarize and better understand the differences discussed and the reasons behind methodological decisions. A side-by-side structural superimposition of the orthosteric binding pockets, showing the differences in key residue positions (especially at the extracellular gate), would be highly helpful.
4. Success rate. Previous work using an inactive AF2 model proved to have higher success rate. Can this be due to the ensemble docking procedure?

Reviewer #4

(Remarks to the Author)
The authors report docking studies against TAAR1. Optimizations of hit compounds led to low nanomolar potency agonists. Some of these agonists also show submicromolar activity at 5-HT1A. Cryo-EM studies with two analogues elucidate binding poses at TAAR1. Studies using a mouse model related to schizophrenia demonstrate brain penetration and biological activity of select compounds in vivo. Two recent, related studies cited by the authors (refs 17, 20) slightly dampen the novelty of the manuscript, however, I think the paper still could be worthy of publication if some appropriate revisions were made.

Specific comments/questions:
It would have been nice to see verification that, at least for the lead compounds, the activities are indeed specific to TAAR or 5HT receptors by running functional assays in untransfected cells (or ideally in cells transfected with empty vector).

In figures and tables, please update legends to indicate if “--" means that that value could not be determined (i.e. the compound was not active enough to determine a value) or that data was just not collected.

Are the concentration-response panels in figure 2 showing activity at TAAR1 or 5-HT1A?

Nanobody35 is modeled without its two disulfide bonds for both structures.

Supplementary table 1: Can you please explain what “frames per image” indicates? I would have expected this value to be an integer.

Line 237: should ‘3206 instead be ‘3207? It appears that, from fig 2b, it should be the latter.

Regarding the pharmacokinetics methods, what are protocols P081724a etc and how were the compounds actually measured (e.g. LC-MS)?

For Fig 5, the 4-, 8- and 12-dB markers appear indistinguishable. Overall, the statistics shown on this figure are not

particularly intuitive, and a more detailed description should be made in the legend.

For Supplementary table 3, why are there no comparisons between V-A vs 0.2 Z3-A? Or V-V vs 0.5 Z3-V? Were these not significant? If so, this should be noted in the legend. It almost appears that the compounds, in the absence of amphetamine treatment, might significantly reduce %PPI themselves.

Version 1:

Reviewer comments:

Reviewer #1

(Remarks to the Author)
The authors have adequately addressed my concerns.

Reviewer #2

(Remarks to the Author)
I co-reviewed this manuscript with one of the reviewers who provided the listed reports. This is part of the Nature Communications initiative to facilitate training in peer review and to provide appropriate recognition for Early Career Researchers who co-review manuscripts.

Reviewer #3

(Remarks to the Author)
All comments were addressed! Great work!

Reviewer #4

(Remarks to the Author)
The authors have adequately addressed my prior comments and questions.

Open Access This Peer Review File is licensed under a Creative Commons Attribution 4.0 International License, which permits use, sharing, adaptation, distribution and reproduction in any medium or format, as long as you give appropriate credit to the original author(s) and the source, provide a link to the Creative Commons license, and indicate if changes were made.
In cases where reviewers are anonymous, credit should be given to 'Anonymous Referee' and the source.
The images or other third party material in this Peer Review File are included in the article’s Creative Commons license, unless indicated otherwise in a credit line to the material. If material is not included in the article’s Creative Commons license and your intended use is not permitted by statutory regulation or exceeds the permitted use, you will need to obtain permission directly from the copyright holder.

To view a copy of this license, visit https://creativecommons.org/licenses/by/4.0/

University of California
San Francisco

UCSF

Department of Pharmaceutical Chemistry

April 6, 2026
Dear Colleagues,

Brian Shoichet
Professor & Chair

1700 4th Street
Byers Hall, BH-508D
San Francisco, CA 94143-2550

Tel (415) 514-4126
Fax (415) 514-4260
shoichet@cgl.ucsf.edu

We are responding to the reviews of our manuscript, "*Structure based discovery of antipsychotic-like TAAR1 agonists*" (NCOMMS-25-80999) by Huang et al. All three reviewers supported publication, though all had specific suggestions for improving it. We take each of critique in turn:

Reviewer 1

The reviewer was broadly supportive of the manuscript, writing, "*This manuscript is a strong addition to the success of virtual docking screens for aminergic GPCR small molecule drug discovery by extending to the existing literature*." They did cite several papers that we overlooked; we regret doing so and have cited them in the revised manuscript. They also had several other specific concerns, which we take in turn.

1.1 *… the cAMP GloSensor assay and TAAR1 construct was transfected and expressed in cells incubated for 24h in 10% FBS containing media. While the cells were later transferred to another medium to run the assay, despite a brief washout period (1h), the presence of monoamines in FBS could likely contribute to receptor sensitization and affect the screening performance and subsequent follow-up studies. The authors should clarify if that was the case or whether dialyzed FBS was used, and if not, why not*.

The Reviewer is correct that we did not perform starvation using dialized FBS for the initial agonist-mode single-point screening experiments. However, all subsequent dose–response studies, antagonist-mode single-point assays, and follow-up experiments were conducted under serum-starved conditions with dialyzed FBS to avoid potential interference from endogenous monoamines. For consistency, we have now also repeated the single-point screening experiments under serum-starved conditions using dialyzed FBS. In addition, the Methods section has been updated accordingly to describe the use of dialyzed FBS.

1.2 *I do not see statistics related to biological or technical replicates, nor errors for the cell experiments anywhere in the figure legends. … The legends for Fig 2 should be extended to indicate which receptor is being shown in the concentration-response curves for clarity*.

Well-spotted; we regret this oversight. In the figure legends we now include the numbers of biological and technical replicates and state that data are presented as means with standard errors. We also clarify in the legend of Figure 2 that the concentration–response curves shown are for TAAR1. Although the EC_{50} values for both TAAR1 and 5-HT1A are reported there, the corresponding 5-HT1A curves are provided in Extended Data Figures 2 and 3, and this is now clearly stated in the revised legend.

1.3. *You state seven known antagonists but I'm also not aware of more than one independently validated, active antagonist at the human TAAR1, RTI. Can you please spell the identity of the seven antagonists …? Which antagonist was most enriched in the inactive state model?*

The 7 antagonists to which we refer are described below; corresponding structures are in in SI Table 2. We note that these antagonists are only relevant in that they helped us with control calculations and to template, for instance, Boltz-2 structure calculations. They are:

1. RTI-7470-44, perhaps the most potent TAAR1 antagonist to date. This compound was also used to generate our Boltz2-derived inactive state structure.
2. EPPTB (see: https://www.pnas.org/doi/10.1073/pnas.0906522106).
3. Compounds 9, 16, 22, and 24, are reported in https://pubs.rsc.org/en/content/articlelanding/2015/md/c5md00400d.
4. Compound 3, was reported in https://doi.org/10.1111/cbdd.12367.

1.4 A *clarification as to how the inactive model was generated would be helpful here (line 144).*
We have added a section to the Methods that reads:

> **Boltz-2 modeled inactive structure.** Because no inactive-state experimental structure of TAAR1 state was available, we turned to Boltz-2 [57] to model the inactive-state, as bound to the antagonist, RT-7470-44[58]. MSAs were generated using the mmseqs2 server, and structure prediction was performed with Boltz-2 (v2.0.3) using the default settings (3 recycling steps, 200 sampling steps, 1 diffusion sample, step size of 1.638). The predicted structure was then used to generate docking energy grids following the same procedure as the active-state structure.

1.5 *A major point of ambiguity is the in vivo mechanism of action. The compounds used for behavioral testing were shown to be dual 5-HT1A and TAAR1 agonists, with the authors also noting the effect of 5-HT1A activation for antipsychotic efficacy. However, the paper attributes the behavioral benefits primarily to TAAR1 agonism. This presents a mixed message, and the authors should clarify whether their optimization goal is a selective TAAR1 agonist or if this specific TAAR1/5-HT1A polypharmacology profile is the intended end goal*.

We take this point, and now clarify on p.10 in the section "Initial hit optimization" that, "To the extent that design was used in optimization, it was driven by complementarity to the TAAR1 structure; activity for the 5-HT1A was investigated subsequently."

1.6 "… *it would be very reassuring to show TAAR1 dependence of the antipsychotic-like effects exerted by the compounds in vivo. A commercially available antagonist (RTI-7470-44) would be useful here. Dissection of the 5-HT1A receptor contribution would be informative*."

To address the Reviewer's concern, we used the selective TAAR1 antagonist EPPTB to see if we could reverse the restoration of pre-pulse inhibition conferred by our TAAR1 agonists. We chose EPPTB over RTI-7470-44 because while the latter low nM affinity for human TAAR1, its affinity for rodent TAAR1 is 100-fold worse, and of course we are doing our experiments in mice. Meanwhile EPPTB has sub-nM activity in mice (ACS Chem. Neurosci. 2022; PNAS 2009—now cited in the manuscript). We now show that EPPTB successfully reverses the activity of our new TAAR1 agonists (**Figure 5**, p.21). In that Figure (below), you can see the reversal of the effects of the TAAR1 agonists by EPPTB in the last column. In short, the antagonist EPPTB reversed the effect of the new agonists in vivo, as requested by the reviewer.

This experiment added to our confidence in the results, but it did demand substantial effort. Disentangling the 5-HT1A contribution would be out of scope.

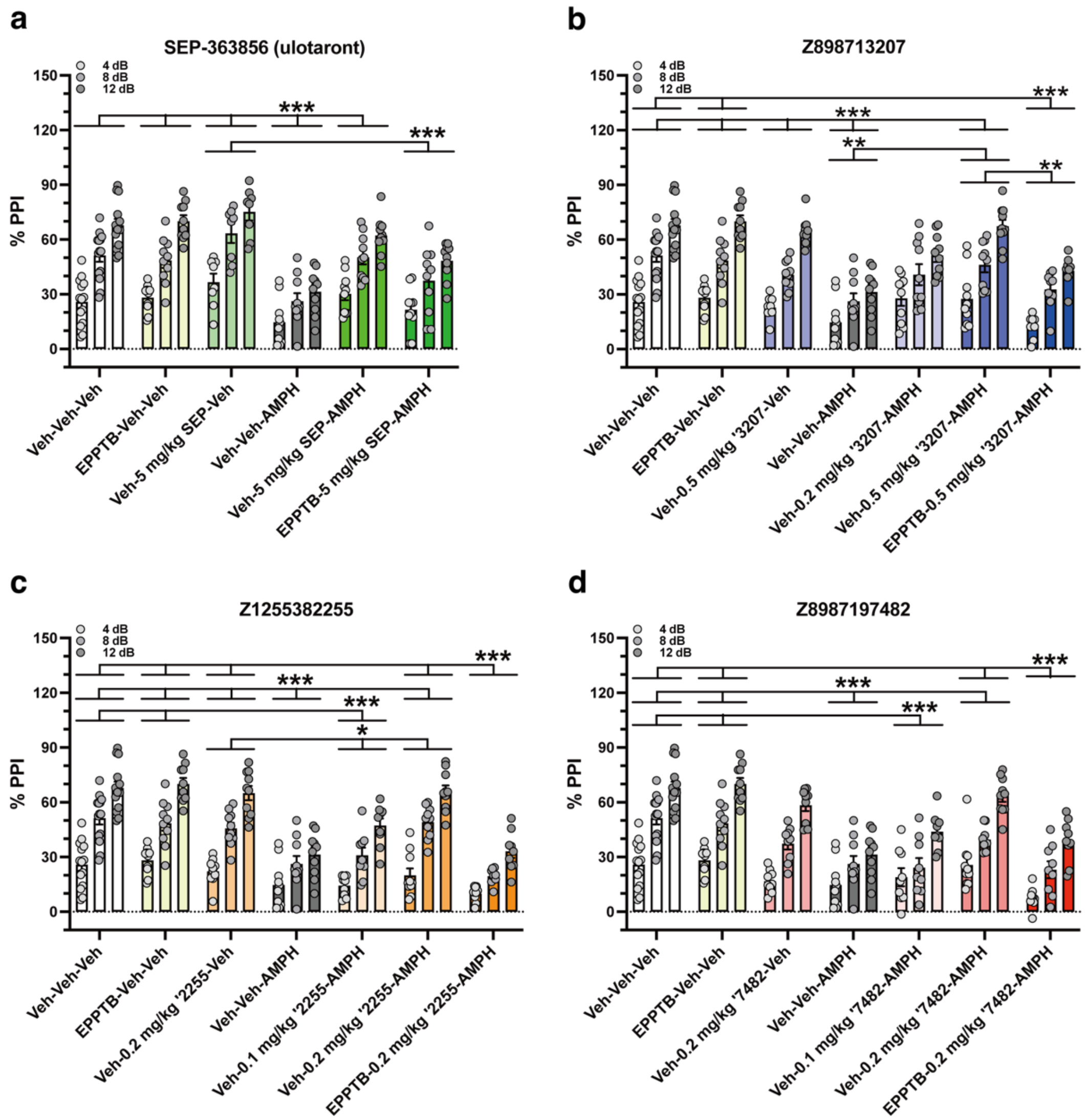

Fig. 5: Effects of SEP, '3207, '2255, and '7482 on restoring amphetamine-disrupted prepulse inhibition. a-d, Mice were administered the vehicle (Veh) or 0.5 mg/kg EPPTB, followed 10 min later with the Veh, 5 mg/kg SEP, or low or high doses of compounds. After 10 min, mice were given the Veh or 3 mg/kg amphetamine (AMPH). They were placed in the PPI apparatus and tested after 10 min of habituation. Effects on PPI on administration of **(a)** SEP, **(b)** '3207, **(c)** '2255, and **(d)** '7482. The data are presented as means and standard errors of the mean; n=8-14 for SEP and n=9-14 for the compounds; **p*<0.05, ***p*<0.01, and ****p*<0.001, *vs.* the indicated group(s). The statistics are located in Supplementary Table 5.

1.7 "… *it would also be useful to know the potency or activity of the compounds at other relevant other aminergic receptors which could contribute to the observed in vivo behavioral effects. The probable lack of D2 occupancy due to the catalepsy test is noted.*"

To answer this question, we have expanded our pharmacological profiling to assess the activity of the three new TAAR1 agonists '3207, '7482 and '2255 across a broader panel of relevant aminergic receptors (**Extended Data Fig. 5**). As shown in the new data, all three compounds retained high agonistic potency at TAAR1, while exhibiting much reduced agonist activity at 5-HT1A, DRD2 and 5-HT2A, indicating a clear preference for TAAR1 over other major monoaminergic receptors.

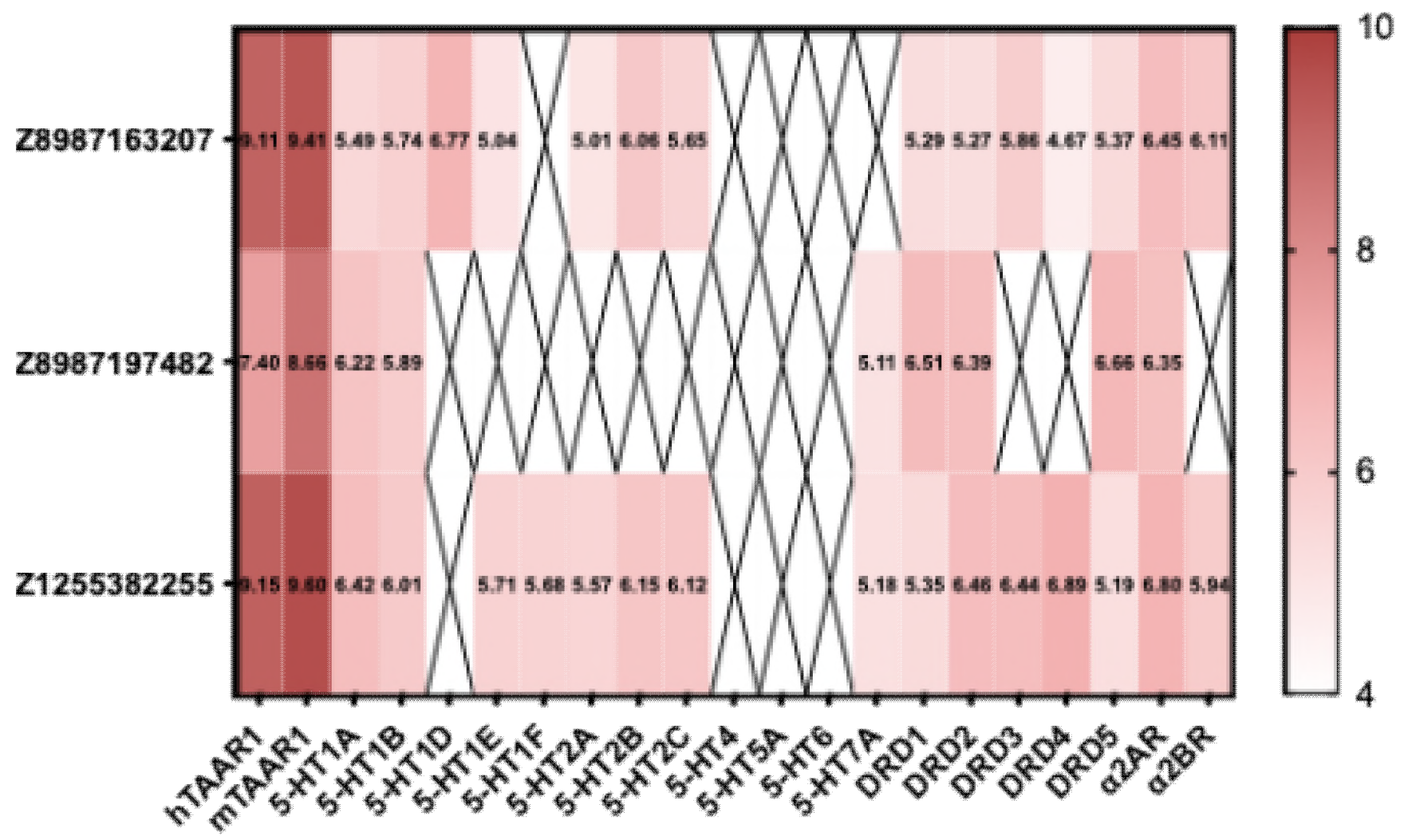

Extended Data Fig. 5. Heatmap of compound activity across selected aminergic receptors. Heatmap showing the activity of ‘3207, ‘7482, and ‘2255 across a panel of related aminergic receptors, displayed as pEC_{50} values obtained from IP1 accumulation or GloSensor cAMP functional assays. “X” denotes either no detectable response or responses for which reliable pEC_{50} values could not be determined within the tested concentration range. Data represent mean values from three independent experiments (n = 3).

1.8 “*The behavioral comparison to ulotaront without a heads-on comparison should not be made*.”

We agree. We used ulotaront (SEP) as a positive control in the experiments These results are presented in Figure 5 (PPI with SEP, ‘3207, ‘2255, and 7482), Supplementary Figure 3 (comparisons of different doses of SEP and EPPTB in a pilot study), Supplementary Figure 4 (null activities with SEP, ‘3207, ‘2255, and 7482), and Supplementary Figure 5 (startle or pulse activities with SEP, ‘3207, ‘2255, and 7482).

Minor comments: (**Sijie)**
• *The primary statistics in Supp. Table 3 appear to incorrectly note Fig 5 as Fig 4*
Well-spotted; we regret this error and the figure reference has now been corrected from Fig. 4 to Fig. 5.

• *-Gs should be referred to as Gαs throughout the manuscript*
We have clarified the distinction between Gs and Gαs in the revised manuscript. Gs refers to the heterotrimeric Gs protein complex, while Gαs specifically denotes the Gαs subunit.

• *There are typos in Refs 62 and 71.* We have corrected these.

Reviewer 3
Reviewer 3 was enthusiastic, writing: “*I strongly recommend the publication of the manuscript, below just minor comments/suggestions*.*”* We are grateful for their support and take their critiques in order:

3.1. *The novelty of the identified ligands based on a Tc cutoff do not …(identify…) the closest known active… I would … report the similarity the most chemically similar known TAAR1 agonist available in public databases …. Consider adding a figure … that visually compares the molecular scaffolds of the two or three most potent leads with the structures of ulotaront and/or known ligands, allowing readers to readily appreciate the chemical differences*.

This is well-taken. We now add ulotaront to **Figure 1E** where we first show the docking hits. For each docking hit shown in **Supplementary Table 1**, we now add the closest known TAAR1 agonists from ChEMBL to show similarity/novelty vs. knowns. For convenience, we show the first several rows of the SI Table here—the actual table extends for 2.5 pages in the SI. The closest Tc is 0.33 similarity to a known

ChEMBL ligand, which remains a scaffold-hop away for ECFP4 fingerprints.

Supplementary Table 1. Structural similarity of hits and lead compounds to reference TAAR1 ligands (full table extends for 2.5 pages**)**

ID	Structure	Tc[a]	ChEMBL ligands
Z1197877490	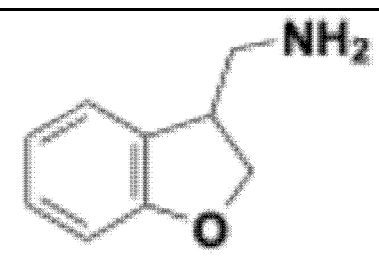	0.32	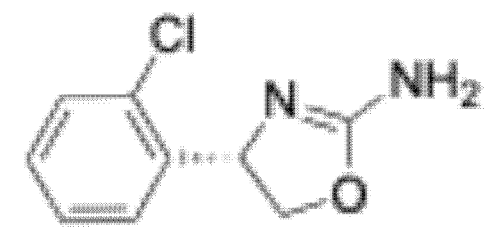
Z2489361097	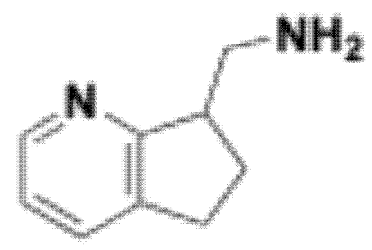	0.27	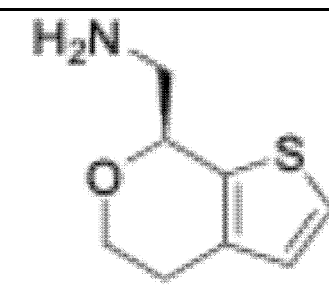
Z2211231413	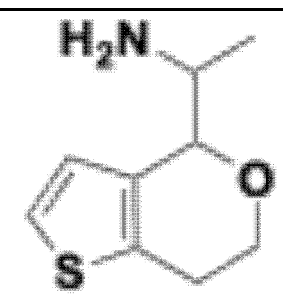	0.31	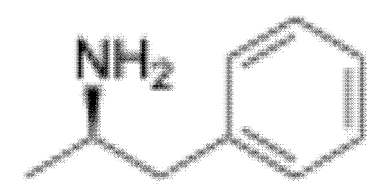
Z1255361401	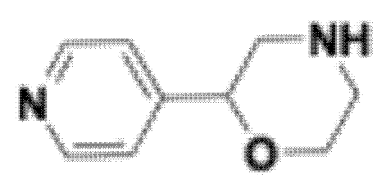	0.18	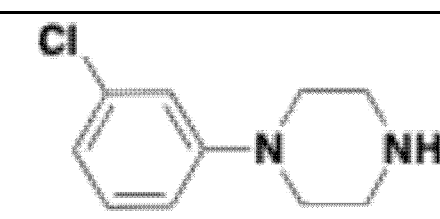

3.2. *The manuscript touches upon the critical observation that docking against the active state (Cryo-EM) yielded an exclusive set of agonists, while the use of an inactive state model may have yielded different results. Please explain the use of Boltz-2 for the inactive state modeling. Would it be possible to provide also the pdb structure of the Boltz-2 model?*

Good point. We now add the Boltz-2 structure via figshare (https://figshare.com/s/a403e4b98e4afb2999f3) and note that in the text (p.9, top). Explaining our use of Boltz-2, we now write, "To better understand this result, we modeled an inactive structure using Boltz-2[57], which is among the fastest of the co-folding methods and has been optimized for interaction energy prediction."

3.3. **a.** *AF2 is introduced only in comparison with the newly released cryoEM structure. Why was not used for the agonists/antagonists comparison?* **b.** *A comparison between the active-state structure (8W8A and new one) and the AF2 and Boltz-2 models could be helpful …. A … superimposition of the orthosteric binding pockets, showing the differences in key residue positions (especially at the extracellular gate), would be highly helpful.*

a. We probably could have used AF2, but using a co-folding method allowed us to insist on complementarity to an antagonist, and the agonist modeling in the Carlsson *Sci Adv* paper shows how AF2 can muddle the structure. For optimizing ligand-protein interaction interfaces, we believe that the co-folding methods are now considered state-of-the-art. We might have also used AF3 or Chai, but we have done a lot of work with Boltz-2, found that for ligand-protein complex modeling it has some advantages over AF3 and Chai, and it is certainly the fastest of the methods (as noted above).

b. This is well-taken. We now superpose the agonist structure used for docking, one of the new cryoEM structures determined here, the AF2 model used in Carlsson et al.,, and the Boltz-2 model of the inactive state (**SI Figure 2**). As can be seen, the conformation of the orthosteric site inf the AF2 model has over-collapsed, relative to the experimental cryoEM structure, shrinking the site even versus the agonist-bound conformation in the experimental structures. Meanwhile, the cytoplasmic side of the AF2 model is actually in the inactive state. The Boltz-2 model has an expanded orthosteric site, consistent with the larger size of

the antagonist that it was co-folded to fit and in the cytoplasmic face adopts an inactive structure (as it should and unlike the AF2 model).

We now call out these superpositions on p.9 of the revised text, writing,

> The unusual ability of the TAAR1 active state docking to prioritize agonists, and of the inactive structure to relatively prioritize antagonists, may reflect the size distinction between agonists and antagonists, and correspondingly between active and inactive receptor conformations (Supplementary Fig. 2). TAAR1 agonists are typically smaller than its antagonists, and the TAAR1 orthosteric site compacts on activation (the site contracts even more in a TAAR1 modeled structure used for docking (Supplementary Fig. 2), a point to which we will return).

3.4. *Previous work using an inactive AF2 model proved to have higher success rate. Can this be due to the ensemble docking procedure*?

We agree that it does reflect the ensemble docking and write, “One advantage of the AF2 approach is that multiple models were calculated and those that best-enriched ligands in control calculations were used prospectively.” (p.16-17). As we also note, optimizing to enrich the known agonists may have led to an overly compact orthosteric site (see **3.3**, above), reducing the optimizability of the docking hits, which typically involves enlarging them. While the larger size of the experimental orthosteric site did not distinguish true agonists from inactives as well as the AF2 docking (hit rate was lower, if still a respectable 25%), but it was more forgiving and allowed for optimization (discussed on p.17 of the revised manuscript).

Reviewer 2 & 4

These Reviewers was also supportive, though they too had specific concerns. We take these in order.

4.1. *It would have been nice to see verification that, at least for the lead compounds, the activities are indeed specific to TAAR or 5HT receptors by running functional assays in untransfected cells (or ideally in cells transfected with empty vector).*

This is well-taken. We now perform in empty-vector–transfected cell controls, including single-point assays for all initial hits and lead compounds and full dose–response curves for the lead compounds. No detectable activity was observed for any of the in the empty-vector–transfected cells (below). We now write, “In empty-vector (no transfected receptor) controls, no activity was observed for the initial active compounds nor for the optimized leads, below, in both single-point and concentration-response assays, consistent with an on-target effect of the new molecules in the cellular assays.” (p.8)

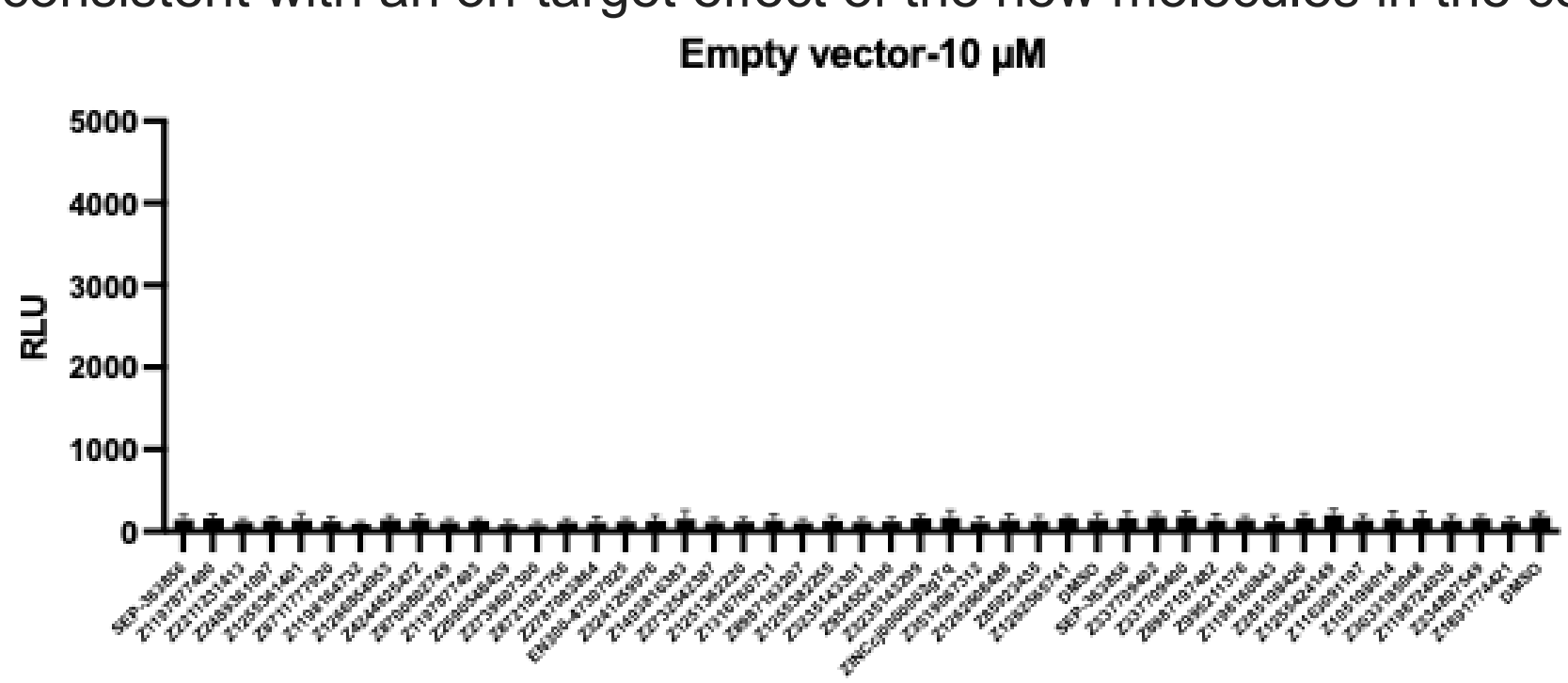

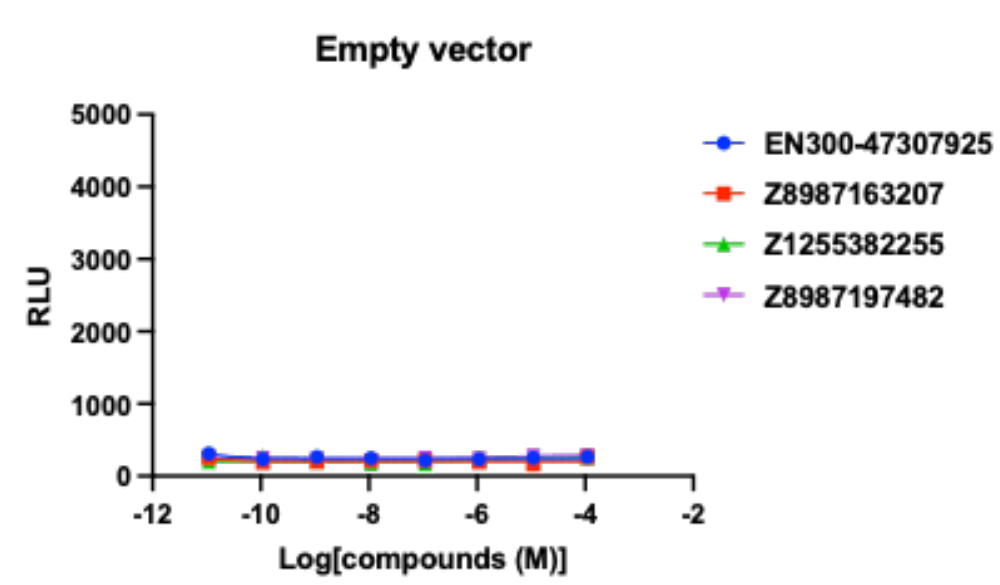

4.2. *In figures and tables, please update legends to indicate if “--" means that that value could not be determined (i.e. … not active enough to determine a value) or that data was just not collected*.

We have done so. “--” indicates that the value could not be determined due to insufficient compound activity, rather than data not being collected.

4.3. *Are the concentration-response panels in figure 2 showing activity at TAAR1 or 5-HT1A?***.**

We now make clear in the Figure 2 legend 2 that the concentration–response curves shown are for TAAR1. Although EC_{50} values for both TAAR1 and 5-HT_{1A} are reported, the corresponding 5-HT_{1A} curves are provided in Extended Data Figures 2 and 3, and this is now clearly stated in the revised legend.

4.4. *Nanobody35 is modeled without its two disulfide bonds for both structures*.

Well-spotted. We have revised both structural models to include the two disulfide bonds in Nanobody35, and the updated models have now been deposited on Figshare as PDB files (https://figshare.com/s/a403e4b98e4afb2999f3), and of course in the PDB itself.

4.5. *Supplementary table 1: Can you please explain what "frames per image" indicates? I would have expected this value to be an integer*.

Again, well-spotted; "frames per image" in SI Table 1 should be "dose per frame", the mistake has been corrected in this revision.

4.6. *Line 237: should '3206 instead be '3207? It appears that, from fig 2b, it should be the latter*. Correct; this error on our part has been fixed.

4.7. *Regarding the pharmacokinetics methods, what are protocols P081724a etc and how were the compounds actually measured (e.g. LC-MS)?*

We now add the full PK methods to our Methods section, beginning on p.37 (two pages of text added).

4.8. *For Fig 5, the 4-, 8- and 12-dB markers appear indistinguishable. Overall, the statistics shown on this figure are not particularly intuitive, and a more detailed description should be made in the legend*. **Bill.**

4.9. *For Supplementary table 3, why are there no comparisons between V-A vs 0.2 Z3-A? Or V-V vs 0.5 Z3-V? Were these not significant? If so, this should be noted in the legend. It almost appears that the compounds, in the absence of amphetamine treatment, might significantly reduce %PPI themselves*.

We agree and regret the confusion. Supplementary Table 3 (now Supplementary Table 5) incorrectly referred to Fig. 4a-c for the PPI analyses; this has been corrected to indicate that these statistics correspond to Fig. 5a-d. To simplify the presentation, the data are presented as separate panels for SEP and each of the compounds in Figure 5. We have substantially revised the legend for Figure 5 to more clearly describe the experimental design and the numbers of mice tested. We have substituted symbols (i.e., *, **, ***) denoting statistical significance rather than exact p-values to simplify the overall group differences and we state that all statistics (primary and post-hocs) can be found in Supplementary Table 5.

In summary, we have addressed each of the Reviewers' points, wherever possible by simply adopting their suggestions, strengthening the manuscript. We thank the Reviewers for the time they have lavished upon this manuscript and would be thrilled to see it appear in *Nature Communications.*

With thanks for your consideration—

Brian Shoichet, Professor, UCSF
